# Univariate- and machine learning-based plasma metabolite signature differentiates PSC-IBD from IBD and is predicted to be driven by gut microbial changes

**DOI:** 10.1007/s11306-026-02420-w

**Published:** 2026-03-28

**Authors:** Joanna C. Wolthuis, Johannes P. D. Schultheiss, Stefanía Magnúsdóttir, Edwin Stigter, Yuen Fung Tang, Judith Jans, Bas Oldenburg, Jeroen de Ridder, Saskia van Mil

**Affiliations:** 1 Center for Molecular Medicine, University Medical Center Utrecht and Utrecht University, Utrecht, The Netherlands; 2 Oncode Institute, Utrecht, The Netherlands; 3 Department of Gastroenterology and Hepatology, University Medical Center Utrecht, Utrecht, The Netherlands; 4 Department of Genetics, Section Metabolic Diagnostics, University Medical Center Utrecht, Utrecht, The Netherlands

**Keywords:** Ibd, Psc, Plasma, Mass spectrometry, Machine learning, Microbiome

## Abstract

**Introduction:**

Inflammatory bowel disease (IBD) is a group of chronic inflammatory conditions of the gastrointestinal tract comprising two major phenotypes, Crohn’s disease (CD) and ulcerative colitis (UC). Up to 8% of patients with IBD also develop primary sclerosing cholangitis (PSC), characterised by cholestasis and progressive destruction of the biliary tree, resulting in cirrhosis, end-stage liver disease and cholangiocarcinoma. Clinical outcome can currently not be improved through medication, denoting the importance of diagnosis prior to irreversible damage, which requires biomarkers of (early) disease.

**Objectives:**

We employed direct infusion mass spectrometry (DI-MS)-based metabolomics on plasma to build predictive, potentially diagnostic models for PSC-IBC and other phenotypes including IBD subtype, stricture and fistula presence and more. We used this dataset to simultaneously investigate aetiology of these phenotypes.

**Methods:**

Samples of 348 IBD patients were included for analysis. The data was analysed using our previously reported tool, *MetaboShiny*. We built predictive models using Random Forest (RF), and subsequently combined with univariate statistics to rank m/z features connected to PSC-IBD. This ranking was used to perform *mummichog* enrichment analysis connected to metabolic and metagenomic changes.

**Results:**

The highest performing predictive model differentiated PSC-IBD from PSC. The metabolic signature was enriched in changes to amino acid and vitamin metabolism, alongside changes to the metagenome suggesting decreases in anti-inflammatory microbial species and increases in pro-inflammatory species.

**Conclusion:**

These results demonstrate the potential of DI-MS-based metabolomics with machine learning to create diagnostic models and generate hypotheses on the metabolomic-metagenomic level. Sharing our dataset of patients will enrich future human IBD metabolomics research possibilities.

**Supplementary Information:**

The online version contains supplementary material available at 10.1007/s11306-026-02420-w.

## Introduction

Crohn’s disease (CD) and ulcerative colitis (UC), collectively referred to as inflammatory bowel disease (IBD), are chronic relapsing remitting immune mediated inflammatory diseases of the gastro-intestinal tract. Of patients with IBD-colitis, 2–8% have or eventually develop primary sclerosing cholangitis (PSC) and nearly three quarters of patients with PSC present with IBD (Barberio et al., 2021; Karlsen et al., 2017). Primary sclerosing cholangitis is a cholestatic liver disease characterised by chronic inflammation of the biliary tract (BT), leading to fibrosis with intra- and/or extrahepatic stricturing (Karlsen et al., 2017).

PSC-associated IBD (PSC-IBD) presents as a distinctive phenotype. It is characterised by right-sided colitis, frequently with rectal sparing, backwash ileitis and absence of transmural inflammatory lesions and fistulae (Boonstra et al., 2012). Furthermore, PSC increases the risk for end-stage liver disease and cholangiocarcinoma (Munster et al., 2024).

**Fig. 1:**
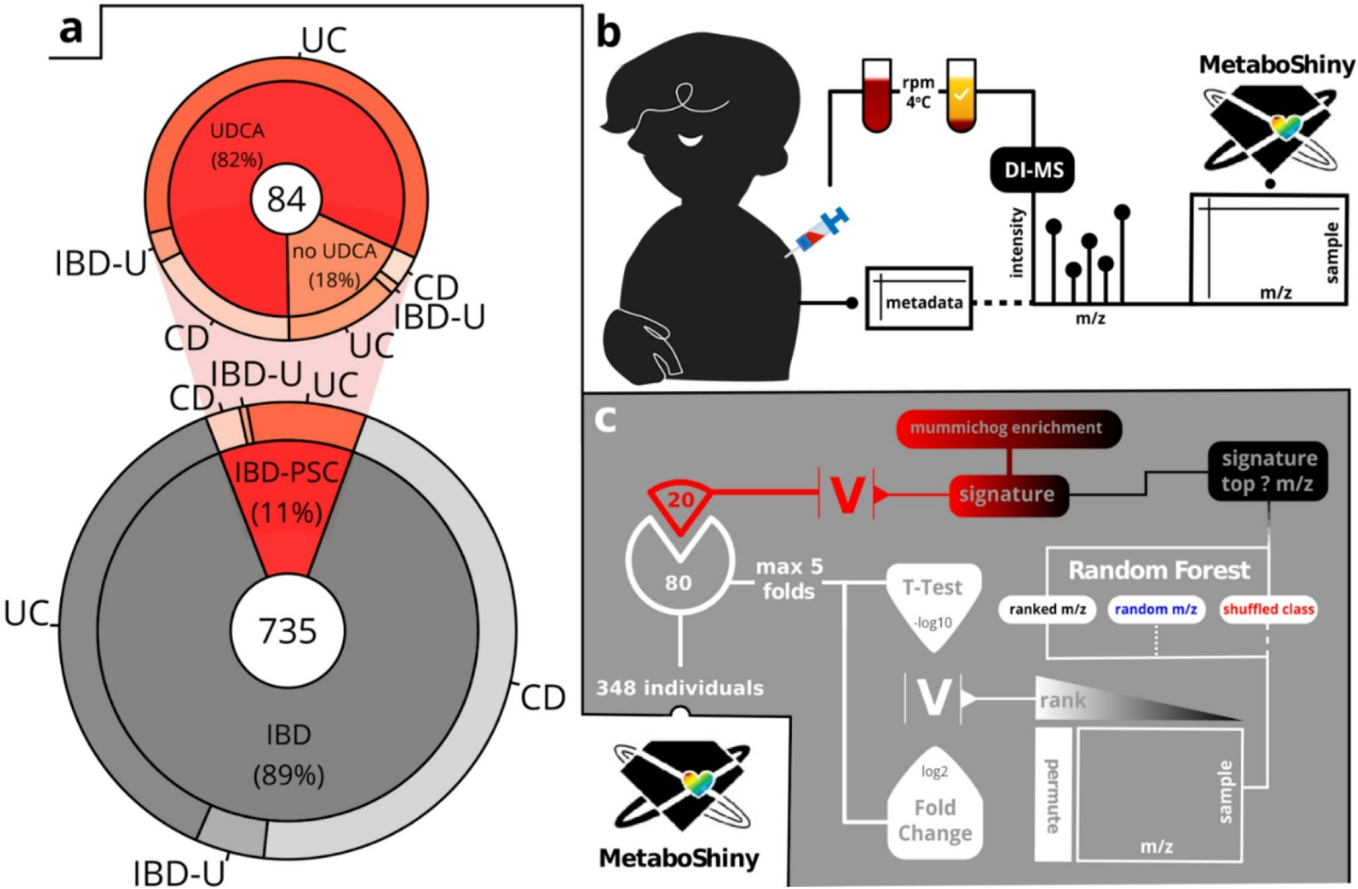
Workflow summary and cohort description (**b**) At endoscopy, plasma was taken from patients alongside metadata per time point. Through direct infusion mass spectrometry (DI-MS) metabolomics data was collected for further processing in MetaboShiny. **(c)** Machine learning and enrichment workflow within MetaboShiny. In max. 5 folds, t-test and fold-change analysis were combined into a V-score. Ranking by absolute V-score was used to create models using a decreasing amount of m/z values, first removing the highest absolute V-score ranked m/z values. Per m/z subset, this was compared to two negative controls - a model using an equivalent amount of random m/z values, and a model using randomised predictive labels differing by question (in the case of PSC-IBD vs. PSC, randomised PSC status labels). From this comparison, a m/z signature threshold containing the majority of predictive information can be extracted and used for enrichment analysis in a 20% holdout set of the data

Primary sclerosing cholangitis is mainly diagnosed through magnetic resonance cholangiopancreatography (MRCP), with endoscopic retrograde cholangiopancreatography (ERCP) being used for inconclusive cases(Morgan et al., 2023). Unfortunately, to date, no medical therapy has been shown to improve clinical outcomes (Krones et al., 2019).

It has been suggested that intervention at a very early disease stage, before irreversible bile duct fibrosis has developed, might be beneficial, but diagnostic non-invasive biomarkers of early disease have not been identified. Little is known about the aetiology of PSC and PSC-IBD, with the majority of known causes attributed to heritability, which only explains a fraction of PSC cases (Ji et al., 2017). Additionally, the transcriptome, proteome, metabolome and microbiome have all shown differences between IBD and PSC-IBD patients (Quraishi et al., 2020; Leibovitzh et al., 2024; Vessby et al., 2022; Fererberger et al., 2024). Further investigation of molecular and microbial changes in (PSC-)IBD patients is required to fully understand disease aetiology and pinpoint novel therapeutic targets. The metabolome is the closest to the biological phenotype, giving an as accurate possible readout of the disease state (Ramautar et al., 2013).

Direct-infusion mass spectrometry (DI-MS) is a fast and cheap high-throughput method to measure plasma metabolites (de Sain-van der Velden et al., 2017). Using DI-MS, we measured over 700 plasma samples in a cohort of IBD patients undergoing colorectal cancer (CRC) surveillance. The data were analysed using our previously developed application *MetaboShiny*, to build metabolic signatures and study their biological relevance using machine learning methods (Wolthuis et al., 2022). We have extended this to investigate the gut microbiome by proxy, using profiles of metabolites that have previously been connected to bacterial gut presence or absence, potentially providing insights in PSC-IBD disease mechanisms (Dekkers et al., 2022).

## Results

### Sample collection

Samples were obtained from a Dutch prospective multicentre cohort study on IBD surveillance. Because of the increased risk of CRC in patients with IBD with colonic involvement, these patients undergo surveillance through colonoscopy. Patients underwent surveillance yearly, or more frequently in accordance with the Dutch guidelines on IBD-surveillance.

Surveillance was started 8 years after symptom onset, except for PSC-IBD patients in which surveillance is initiated at diagnosis due to the extra increased risk of CRC. Surveillance intervals in these guidelines depend on several clinical and/or endoscopic risk factors such as the presence of post-inflammatory polyps, the extensiveness of colonic involvement, persistent chronic disease activity and presence of dysplasia in previous years.

### The plasma metabolome accurately predicts PSC in IBD patients

We first evaluated predictive performance of multiple questions based on the available metadata in the cohort, including PSC, stricture and dysplasia presence, IBD subtype and inflammation. Prior to doing so, we first set apart 80% of the samples for use in machine learning, and then distributed those individuals over 5 folds, except in the PSC-IBD model, where four folds were required to properly distribute the four available patients not treated with ursodeoxycholic acid (UDCA).

We then built Random Forest models without any feature selection. A model predicting sex was used as positive control, as it previously has been demonstrated that sex has a strong effect on the human plasma metabolome (Krumsiek et al., 2015). Per model, a negative control was included using the same individual-based fold distribution but with permuted class labels.

**Fig. 2:**
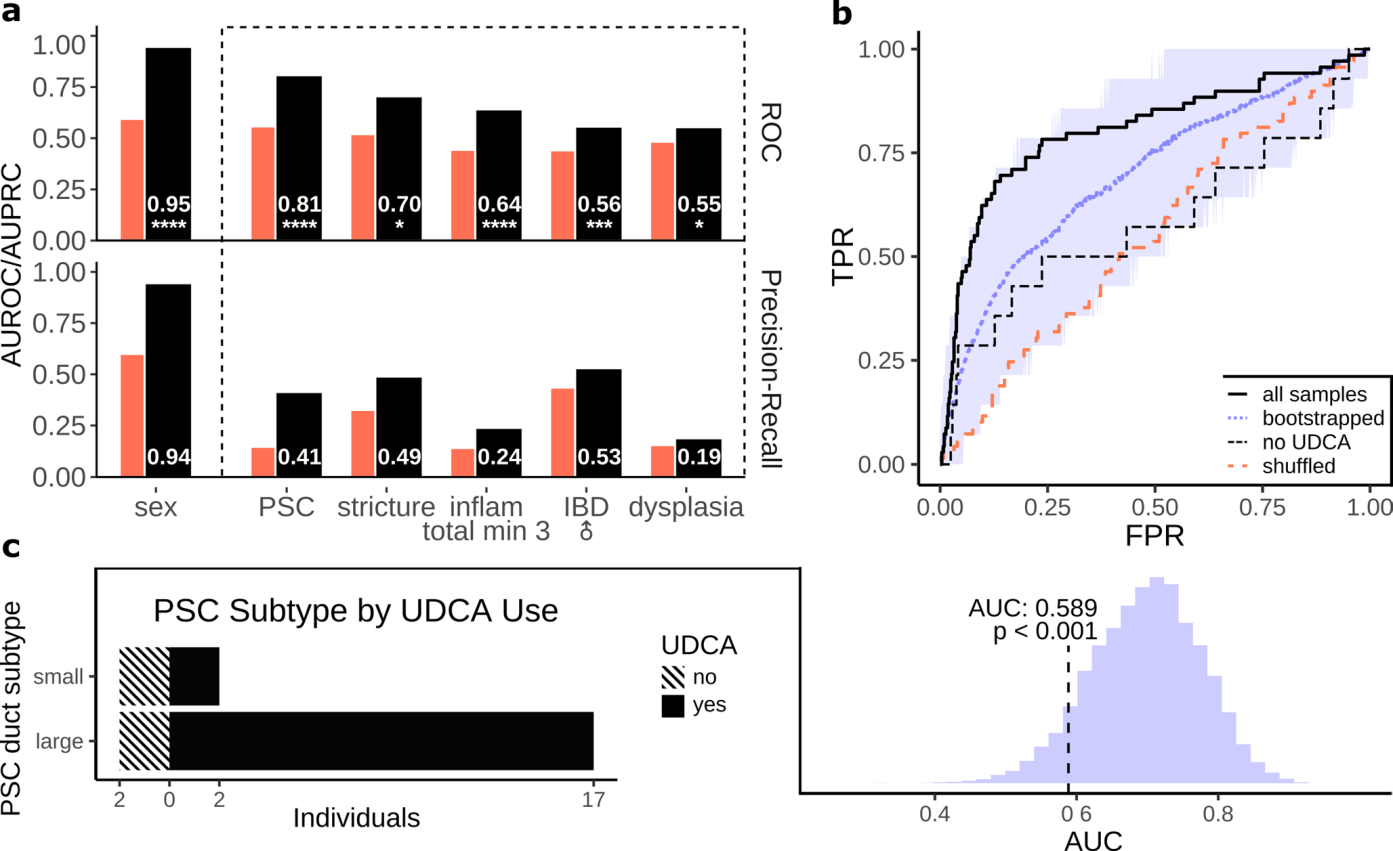
Performance of classifiers predicting sex, PSC, inflammation, dysplasia and IBD subtype. **a** Area Under the Receiver Operator Characteristic (AUROC) and Area Under the Precision-Recall curve (AUPRC) for sex, PSC, inflammation, IBD subclass and dysplasia. Red left bar of each pair represents the negative permuted label control model. **b** AUROC for PSC model prediction. Black dashed line represents patients untreated with UDCA. Blue line (with 95% margins) represents 10 000 iterations of bootstrapped performance with equal number of samples as the non-UDCA test group. Red dashed line represents the negative permuted label control model. **c** Distribution of PSC subtype and UDCA treatment in individuals in the test set (left). Distribution of bootstrapped AUROC values with t-test p-value of the difference between the non-UDCA AUC and the bootstrapped results (right)

Performance was estimated by cross validation (Fig. 1). Figure 2 demonstrates the (areas under) the cross-validated Receiver Operator Characteristic (AUROC) and Precision-Recall (AUPRC) curves for RF models predicting sex, PSC, inflammation and CD vs. UC (Fig. 2a). We also explored questions involving upper GI involvement, perianal disease, stricture development and PSC in inflamed and non-inflamed contexts.

We observed that the positive control model predicting sex performed best in terms of AUROC (AUROC: 0.95****, AUPRC: 0.94) followed by PSC (AUROC: 0.81****, AUPRC: 0.41), inflammation (AUROC: 0.64****, AUPRC: 0.24), stricture presence (AUROC: 0.70*, AUPRC: 0.49), CD vs. UC in male patients (AUROC: 0.55****, AUPRC: 0.53) and dysplasia (AUROC: 0.55*, AUPRC: 0.19). The remaining models were not significant/lacked predictive power (Figure S2). Overall, the predictive signal was strongest in the PSC-IBD model. Given the effectiveness of our model predicting the PSC-IBD phenotype, distinct clinical presentation and relatively unknown aetiology, we next prioritised investigation of the PSC-IBD model.

Part of this involved medication presence within the PSC-IBD patients, as the majority of IBD-PSC patients (82%) were undergoing ursodeoxycholic acid treatment.

To evaluate model flexibility, we tested the predictive performance of this model on only the UDCA-negative patients in each test set. The PSC-IBD predictive model underperformed on these individuals compared to the all-sample model (Fig. 2b). Of the four individuals in this test subset, 2/4 (50%) non-UDCA were diagnosed with small-duct PSC, as compared to 4/23 (17%) of the individuals in the full test set.

We next evaluated the effect of the highly reduced number of samples in the non-UDCA test set. This was done through repeatedly evaluating PSC-IBD model performance on an equal amount of samples by means of bootstrapping. The performance of the non-UDCA model (AUROC: 0.59) was significantly lower than the AUROC obtained through bootstrapping with matched sample size (*p < 0.001*, Fig. 2c). This result suggests that the reduced performance of the model in non-UDCA patients is not due to the small sample size and is potentially due to the relatively low number of small-duct PSC patients, which has little representation in the training set and may therefore be more difficult to predict.

**Fig. 3:**
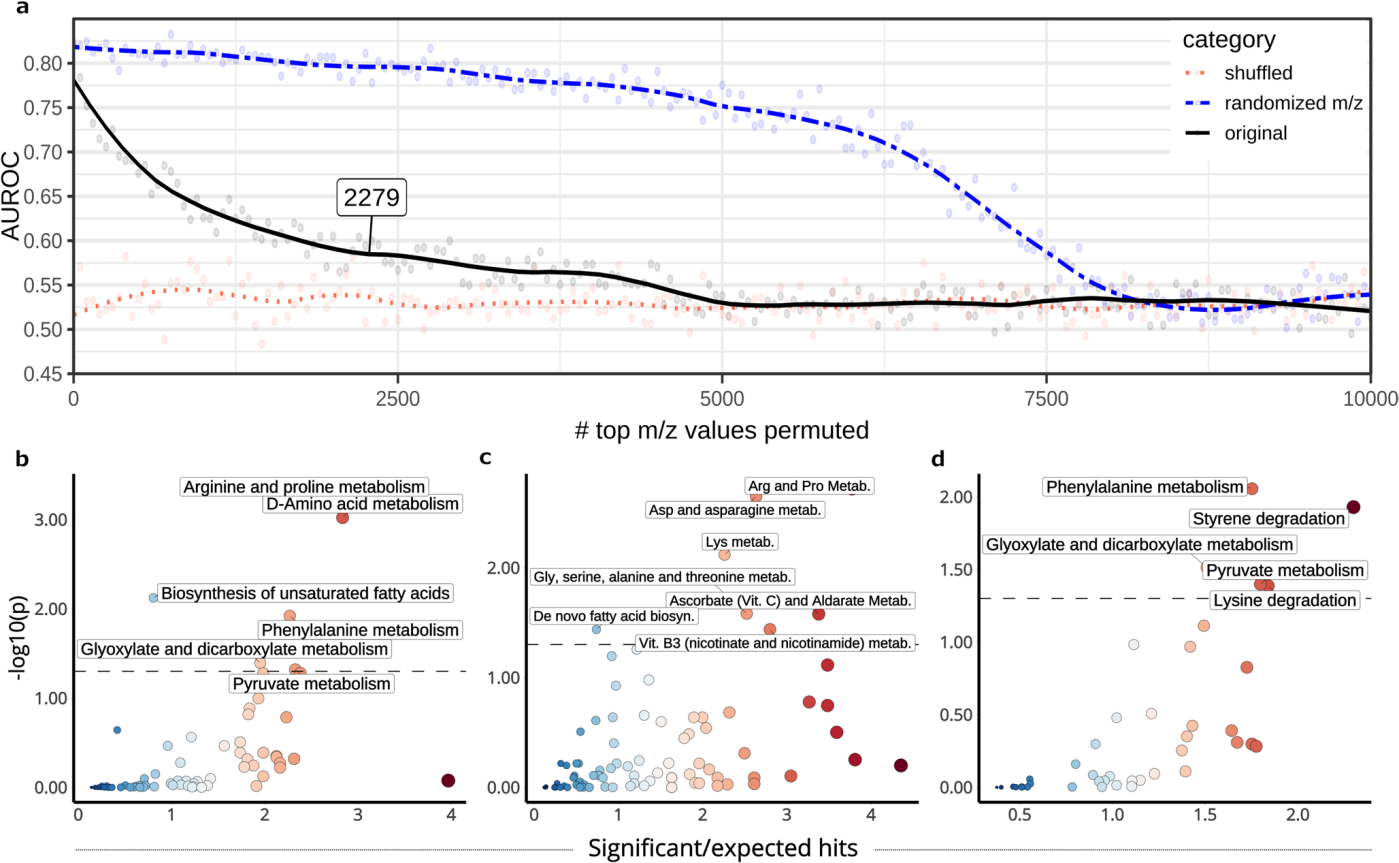
Feature selection, signature threshold determination and enrichment analysis. **a** Signature determination in PSC. Label-shuffled negative control is represented by the red dashed line. Blue dotted line represents a second negative control built by removing random features rather than ranked. Black solid line represents the models built by removing ranked m/z values, this line is used for signature determination. **b** Enrichment results for KEGG *homo sapiens* metabolism. Dotted line represents an EASE p-value of 0.05. **c** Enrichment results for MetaFishNet database. **d** Mummichog results for the metabolic signature in KEGG microbial metabolism

### Amino acid metabolism is enriched in PSC-IBD metabolic signature

To gain insights into the metabolic processes involved in PSC-IBD, we aimed to determine a metabolic signature comprising a group of m/z values representing the biological signal of interest. To determine which m/z values to include, we applied a feature elimination approach based on univariate testing. For each of the previously defined machine learning (ML) folds, we calculated the ‘V-score**’** ($V$) for each m/z value by combining t-test and fold-change analysis results through multiplication.

We then ranked all m/z values in descending absolute $V$ (|$V$|), the absolute value was used as we aimed for the direction of change between PSC-IBD and IBD without PSC to be equally important. As PSC has been previously correlated to sex, we first wished to investigate the relationship between the PSC and sex V-score rankings (Ismail et al., 2023). Correlating $\left|V\right|$_sex_ and $\left|V\right|$_PSC_ revealed a correlation of ϱ=0.144 (*p* < 0.001), signifying a weak but significant correlation between the absolute V rankings of m/z values when calculated for PSC and sex (Figure S3).

This low correlation demonstrates that PSC presence and sex are unlikely to be confounded, allowing us to further investigate the IBD-PSC model without correcting for sex.

We next repeatedly built RF models, starting with all available m/z values. We then proceeded with stepwise permutation of the m/z abundances, permuting an increasing amount of high-ranking m/z values, building and evaluating the AUROC performance of the per-fold RF models after each permutation step until the majority of signal was permuted.

The predictive performance is evaluated by comparing the result curve to two negative controls, one with permuted class labels, and one permuting a matching amount of random m/z values for each model.

If the ranking was of predictive importance and thus contains the biological signal of interest, we expected only the experimental curve to drop in performance as more and more values are permuted.

In Fig. 3 we demonstrate that permuting high-ranked m/z values results in a stronger decrease of predictive performance compared to random m/z values, suggesting that the PSC-IBD metabolic signal is stronger than random noise present in the dataset. To next define a metabolic signature based on this result, we required a cutoff point of the amount of high-ranking m/z values containing the majority of the predictive metabolic signal. To do so, we calculated the flattest point of the curve fit to the performance data, which resulted in a signature threshold of the highest 2279 |$V$|-ranked m/z values (Fig. 3a).

To investigate which metabolic processes were enriched in this metabolite signature, we performed enrichment analysis through *mummichog*. After switching to the separate 20% of data used for enrichment, we first calculated $V$ by performing and combining t-test and fold-change analyses. With this ranking, we then performed enrichment analysis using the top 2279 m/z values based on |$V$| within the separate 20%. As we wished to investigate both pathways known in human and (the results of) bacterial metabolism, we included pathways related to both.

For human pathways, we tested two pathway collections, KEGG human/*homo sapiens* and the Human MetaFishNet (MFN). Using KEGG facilitates visualisation of the pathways in an intuitive way through *pathview*, whereas both KEGG and MFN have been referenced in literature in similar studies, facilitating comparison when interpreting the results (Walker et al., 2022).

When investigating enrichment of compounds in the metabolic signature in the KEGG human database, we find enrichment of pathways *‘D-Amino acid metabolism’*, ‘*Arginine and proline metabolism’* ‘*Biosynthesis of unsaturated fatty acids’ ‘Phenylalanine metabolism’ ‘Glyoxylate and dicarboxylate metabolism’ and ‘Pyruvate metabolism’* (Fig. 3b, Tables S2-S3, Figures S4-S9)*.*

The human MFN pathway collection showed enrichment of ‘Arginine and Proline Metabolism’, ‘Aspartate and asparagine metabolism’, ‘Lysine metabolism’, ‘Glycine, serine, alanine and threonine metabolism’ and ‘De novo fatty acid biosynthesis’ alongside pathways connected to vitamin metabolism: ‘Ascorbate (Vitamin C) and Aldarate Metabolism’ and ‘Vitamin B3 (nicotinate and nicotinamide) metabolism (Fig. 3c, Tables S4-S5). To facilitate comparisons with published experiments, we also performed enrichment analysis using the MFN pathway collection on a signature of 2106 m/z values, which was determined through per-m/z logistic regression. Here, we ranked features in descending |Z| and repeated our permutation-based experiment (Figure S11, Tables S12-S13). Significantly enriched were ‘Aspartate and asparagine metabolism’, ‘Arginine and Proline Metabolism’, ‘Lysine metabolism’, ‘Glycine, serine, alanine and threonine metabolism’, ‘Carnitine shuttle’, ‘De novo fatty acid biosynthesis’, ‘Phosphatidylinositol phosphate metabolism’ and ‘Ascorbate (Vitamin C) and Alderate Metabolism’.

To investigate potential microbial metabolism changes, we next investigated enrichment in the KEGG microbial database, which also showed enrichment in amino-acid related pathways ‘*Phenylalanine metabolism’* mentioned in the human database, *‘Lysine degradation’ and ‘Styrene degradation’* (Fig. 3d, Tables S6-S7, Figures S8-S10).

### Metabolite PSC-IBD signature is enriched in gut metagenome markers

**Fig. 4:**
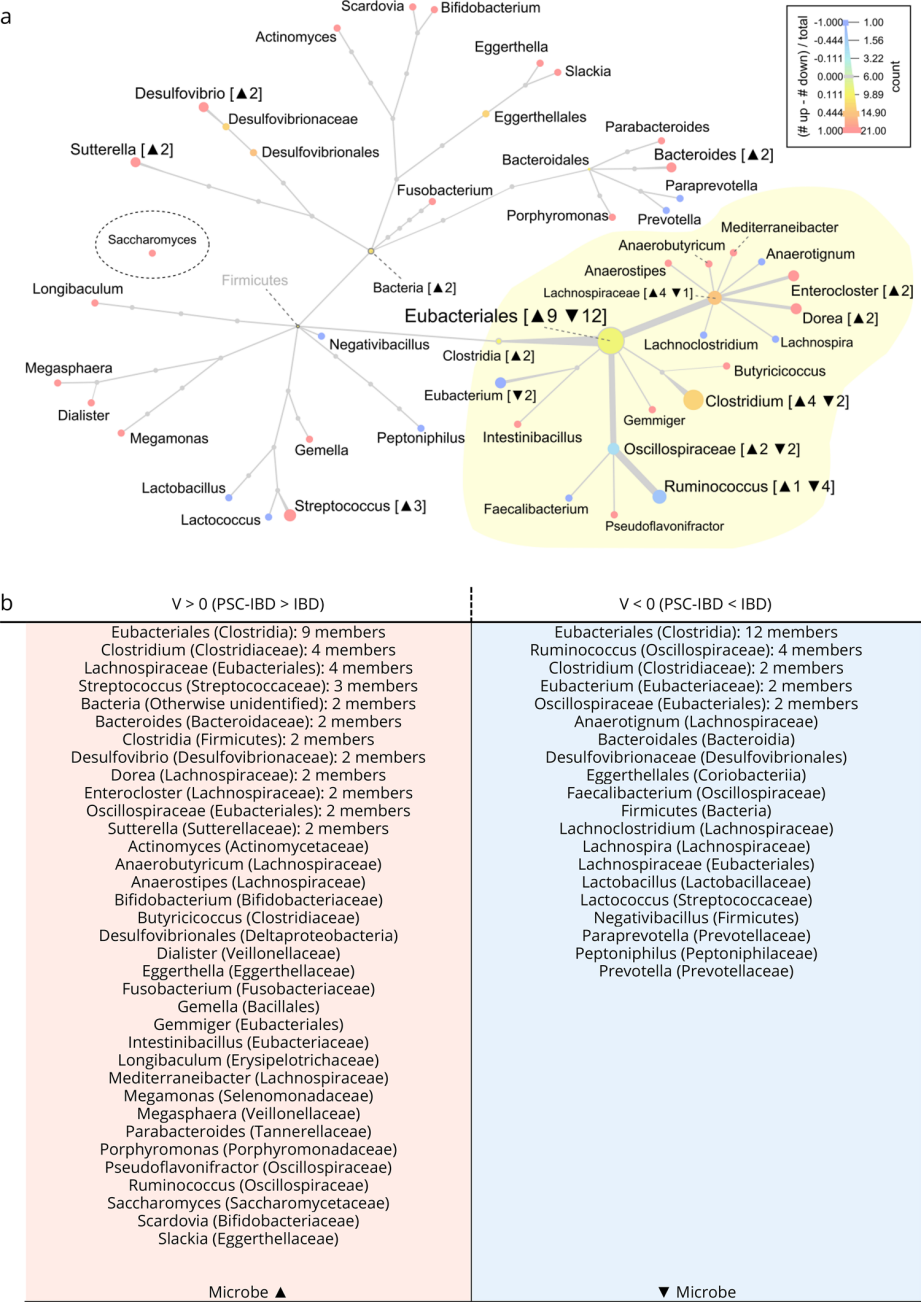
Enrichment in signature of metabolite sets associated with gut microbial species. **a** Taxonomy of microbial species connected to enriched metabolite sets. Blue branches indicate relatively more enriched metabolite sets in $\mathrm{V}<0$. Yellow highlighted area represents Eubacteriales. **b** Overview of microbiome changes in metabolites with either a positive or negative V in the signature connected to PSC-IBD

**Fig. 5:**
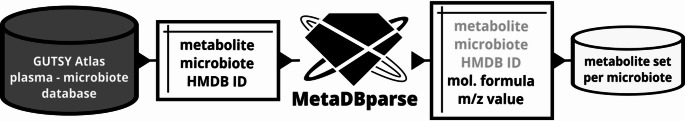
Overview of GUTSY Atlas database creation for mummichog analysis. Base data was enriched using the HMDB parsed through MetaDBparse, resulting in metabolite sets connected to individual microorganisms

To further investigate potential microbial influences on the metabolome, we leveraged the GUTSY Atlas, a database containing correlations between gut bacteria and plasma metabolite markers (Dekkers et al., 2022), using our *MetaDBparse package* to extract molecular formula and m/z values using available HMDB identifiers. We defined a new *mummichog* database for use in *MetaboShiny*, with each ‘pathway’ defined as a metagenomic species (Fig. 5). Using the 19 adducts used for the MFN and KEGG enrichment analyses, we performed another round of enrichment analyses, now using this novel database. To investigate both decreased and increased abundance of bacteria and fungi, we split the 2279-m/z signature in two separate sets, based on $V$ above or below zero, where $V>0$ indicated relative increased abundance of a metabolite in PSC-IBD as compared to PSC, and $V<0$ the opposite (Fig. 4).

The enriched metabolite sets indicated potential increases in 9 members of Eubacteriales, 4 members of *Lachnospiraceae* and *Clostridium*, 3 members of *Streptococcus*, 2 members of *Bacteria*, *Bacteroides*, *Clostridia*, *Desulfovibrio*, *Dorea*, *Enterocloster*, *Oscillospiraceae* and *Sutterella*, alongside individual members in *Actinomyces*, *Bifidobacterium*, *Butyricicoccus*, *Desulfovibrionales*, *Dialister*, *Eggerthella*, *Fusobacterium*, *Gemella*, *Gemmiger*, *Intestinibacillus*, *Longibaculum*, *Mediterraneibacter*,* Megamonas*,* Megasphaera*,* Parabacteroides*,* Porphyromonas*,* Pseudoflavonifractor*,* Ruminococcus Saccharomyces*,* Scardovia* and *Slackia* (Fig. 4, Tables S8-S11). Of these species increased in PSC-IBD relative to IBD, *Eggerthella* and *Fusobacterium* have previously been connected to increased inflammation in both gut and liver (Eicher, and Mohajeri, 2022); Sabino et al., 2016).

The reverse experiment, aiming to investigate which microorganisms could be decreased in PSC-IBD, was enriched in metabolite sets connected to 12 members of *Eubacteriales*, 4 members of *Ruminococcus*, multiple groups with 2 members including *Clostridium*,* Eubacterium* and *Oscillospiraceae* and *individuals* in *Anaerotignum*,* Bacteroidales*,* Desulfovibrionaceae*,* Eggerthellales*,* Faecalibacterium*,* Firmicutes*,* Lachnoclostridium*,* Lachnospira*,* Lachnospiraceae*,* Lactobacillus*,* Lactococcus*,* Negativibacillus*,* Paraprevotella*,* Peptoniphilus* and *Prevotella*.

Some of these species have been previously connected to maintaining gut epithelial integrity through SCFA production, including *Ruminococcus*, *Lactobacillus*, *Eubacterium*, *Faecalibacterium* and *Prevotella* (Xie et al., 2022; Fusco et al., 2023). Overall, relatively more distinct metagenomic species-related metabolite sets were found as significantly increased than decreased in the PSC-IBD patients.

Furthermore, the ratio of *Oscillospiraceae* to *Lachnospiraceae* was reversed between the two experiments, where relatively more *Oscillospiraceae*-related metabolite sets were enriched in the $V<0$ results, indicating a potential relative reduction in bacteria of that subset, and the matching increase of *Lachnospiraceae* in the $V>0$ subset suggesting increased presence of these bacteria in the gut of PSC-IBD patients. Similar relative increases were visible in *Proteobacteria*,* Coriobacteria Clostridia*,* Coriobacteria* and *Clostridiaceae*. The only non-bacterium metabolite set found as significant was *Saccharomyces*, which was increased (significant in $V>0$ subset of metabolites).

Some bacterial subsets were uniquely enriched in the signature metabolites with $V>0$. We see potential increases of metabolite sets correlated to Negativicutes (*Dialister*,* Megasphaera*,* Megamonas*), specific *Lachnospiraceae* members *(Dorea*,* Anaerostipes*,* Mediterraneibacter)*,* Eggerthellaceae (Eggerthella*,* Suterella)*,* Streptococcus*,* Bacteroidales (Parabacteroides*,* Porphyromonas*,* Bacteroides)*,* Actinomycetia (Bifidobacterium*,* Scardovia*,* Actinomyces)*,* Fusobacterium*,* Longibaculum*,* Tissierellia*,* Intestinibacillus*,* Gemmiger* and *Desulfovibrio*.

Inversely, some were only present as significantly enriched in the $V<0$ subset of metabolites, *Prevotellaceae (Prevotella*,* Paraprevotella)*,* Negativibacillus*,* Peptoniphilus*,* Lachnospira*,* Lachnoclostridium*,* Lactobacillus*,* Lactococcus*,* Eubacterium*,* Anaerotignum*,* Faecalibacterium* and unspecified members of *Desulfovibrionaceae*,* Eggerthellales* and *Bacteroidales.*

These results suggest a relative shift in the microbiome in PSC-IBD patients compared to IBD alone. The metabolite changes differentiating these groups suggest increases in pro-inflammatory bacterial species, and relative decreases in bacteria connected to gut epithelial maintenance in PSC-IBD patients as compared to patients diagnosed with IBD alone.

## Discussion

Approximately three quarters of patients with PSC also have IBD, and UC accounts for the majority (80%) of IBD cases associated with PSC (CD 15%, undetermined 5%). Yet, in both PSC-associated CD and UC clinical differences have been described compared to CD or UC without PSC. Patients with PSC often have more quiescent intestinal disease and mild inflammation endoscopically. The disease can even appear endoscopically quiescent while there is active colitis/inflammation on biopsy. Not only does PSC-IBD clinically differ from IBD without PSC, at the genetic level, PSC-IBD also seems to be a distinct disease, as the genome-wide correlation between UC and CD was found to be higher than their individual correlation to PSC, alongside specific susceptibility loci uniquely being found in PSC-IBD patients (Ji et al., 2017; Janse et al., 2011; Ellinghaus et al., 2013).

Prediction of PSC within IBD patients has rarely been done using metabolomics. Van Vorstenborch et al., 2024 utilised volatile organic compounds in breath and subsequent GC-MS to predict PSC-IBD (van Vorstenbosch et al., 2024). Although beneficial due the non-invasive nature, it offers less insight into the underlying phenotype given the limited reflection of VOCs on overall metabolism, and the potential for contamination through compounds produced by the oral microbiome (Cikach & Dweik, 2012). Furthermore, GC-MS measurements are substantially slower and less economically viable than DI-MS (Wevers et al., 2023). Furthermore, Van Vorstenborch et al. developed this predictive model based on a cohort of just 116 patients. Our model, using a cohort of three times that size, is more likely to perform well on new patients due to the increased generalisability and reduced risk of overfitting from sampling a larger part of the patient population.

We show that in our large cohort of phenotypically mostly heterogeneous patients measured through DI-MS, PSC can be predicted accurately within IBD patients by RF, which supports the hypothesis that PSC-IBD is a different entity from IBD without PSC (Fig. 2).

Furthermore, stricture presence, dysplasia, inflammation and IBD subtype, among other features (Figs. 2, S2) can also be predicted but with lower AUROC as compared to PSC.

To decrease the potential confounding effect of UDCA administration in PSC-IBD patients, we tested our model separately on non-UDCA treated patients, which revealed that the model underperforms on these patients relative to the complete test set (Fig. 2b). However, a relatively larger fraction of the patients in the non-treated set (50% as compared to 17%) were diagnosed with small-duct PSC as compared to the overall dataset (Fig. 2c).

Small-duct PSC (sdPSC) is a more benign PSC variant, which sometimes progresses to large duct PSC (ldPSC) (Nguyen et al., 2022). It has been suggested that sdPSC-IBD is a potential early stage which eventually progresses to ldPSC-IBD (Naess et al., 2014). As the sdPSC phenotype has a milder presentation, these patients may be more difficult for our model to correctly pinpoint. Furthermore, another factor which can cause the lower predictive performance in this subset is the small number of sdPSC patients in the training set, which also may reduce model performance.

Given the UDCA-PSC confounding in our dataset, we additionally investigated which pathways had been found to be altered solely in UDCA independently from PSC.

Walker et al. (2022) characterised the metabolomes of PSC, PSC-IBD and HC patients using high-resolution LC-MS, followed by logistic regression for compound prioritisation, and enrichment analysis using *mummichog*. They found a small but significant difference between the metabolomes of PSC patients treated with UDCA and patients without this treatment, and subsequently found enriched MFN pathways ‘*Bile acid biosynthesis*’ and ‘*Vitamin D3 metabolism*’ characterising that difference. These pathways were, however, not significant in our enrichment analysis, which may suggest that UDCA administration does not impact the metabolome on the pathway level in this cohort of PSC-IBD patients. Furthermore, in a cohort of Primary Biliary Cholangitis (PBC) patients, UDCA treatment did not cause significant metabolomic changes in serum (Hao et al., 2017). To verify that our V-score-based ranking was sufficiently comparable to the study by Walker et al., we additionally determined a ranking based on logistic regression (Figure S11, Table S12-S13). The resulting signature size and enriched MFN pathways were highly similar to the ones from our V-score-based experiment, and the novel pathways were not connected to UDCA administration by Walker et al.

These findings suggest that the impact of UDCA administration on the metabolomic signature is low, and our findings are thus likely medication-independent. Regardless, to improve our model quality, future follow-up studies should include more IBD-PSC patients, ideally prior to UDCA treatment. If available, PSC patients without IBD should also be included in such studies to further strengthen the association of this signature with PSC.

These predictive models that we have built are not just valuable as a first step towards an alternative diagnostic tool but also offer valuable biological information through extraction of the metabolite signature.

By using RF, we found a metabolic signature connected to PSC-IBD consisting of 2279 metabolites (Fig. 3). Our metabolomic signature, also acquired through high resolution MS, is enriched in multiple pathways matching the MFN pathways found by Walker et al. (2022), the majority of which is involved in amino acid metabolism. However, these changes are potentially a consequence of disease rather than cause, as increased methionine, as we see in our results, has been connected to liver disease varying in aetiology (Morgan et al., 1982). Interestingly, we do not see enrichment in changes to bile acid metabolism.

Shifts in the microbiome have also been implicated in PSC-IBD aetiology. Although microbial diversity was not found to significantly differ between IBD and PSC-IBD, differences were found on the genus and species level, a distinct microbiome signature differentiating PSC-IBD from IBD (UC) patients (Bajer et al., 2017). To investigate if this connection was also measurable at the plasma level, we performed enrichment analysis on the m/z signature using the GUTSY Atlas, a database of metabolite sets correlated to gut microbiota (Dekkers et al., 2022).

As mentioned previously, (Bajer et al., 2017) studied the differences in microbiome between PSC (and PSC-IBD) patients, patients with only IBD, and healthy controls. Of the species predicted via our enrichment analysis, they also detected increased abundances of *Streptococcus* and *Clostridium*. In contrast, there was an increase in *Lactobacillus*, which is the opposite of our enrichment analysis (Fig. 4). Increased abundance of *Clostridium* was also found by (Kummen et al., 2017), alongside increased members of the *Veillonella* genus.

Our enrichment analysis demonstrated enrichment of metabolite sets connected to members of *Veillonellaceae*, *Megasphaera* and *Dialister*, of which *Megasphaera* correlates to increased bilirubin and thus disease severity (Cortez et al., 2020).

Overrepresentation of *Fusobacterium*, as suggested by our analysis, has also been connected to PSC independent of IBD, and has been connected to liver cirrhosis (Sabino et al., 2016). Together with the aforementioned *Veillonella*, overrepresentation of *Fusobacterium-related* metabolite sets can differentiate PSC(-IBD) from controls and IBD-only patients (Vieira-Silva et al. 2019).

Damage to the gut epithelium (‘leaky gut’) through microbiome dysbiosis and subsequent or simultaneous inflammation of the gut, such as in IBD, may allow for bacteria or bacterial products to permeate the gut epithelium and translocate to the liver and cause liver damage (Dhillon et al., 2019). Some of the bacteria with enriched metabolite sets in our experiments have been connected to increased risk of gut permeability, such as pro-inflammatory *Eggerthella*, alongside decreases of beneficial anti-inflammatory bacteria such as *Lactobacillus*,* Eubacterium*,* Faecalibacterium* and *Prevotella* (Eicher, and Mohajeri, 2022).

To maintain homeostasis of the gut epithelium, SCFAs play an important role, and decrease of SCFA producers in the gut may harm gut homeostasis and increase the risk of epithelial damage and subsequent intestinal permeability (Hamer et al., 2008). Interestingly, some species suspected to be decreased based on our analysis are short-chain fatty acid (SCFA) producers, such as members of *Ruminococcae* (*Ruminococcus*,* Faecalibacterium)* and *Lactobacillus* that produce butyrate, and *(Para)prevotella* that produce propionate (Xie et al., 2022; Fusco et al., 2023), which suggests a potential decrease in species important to gut epithelium integrity in PSC-IBD patients as compared to IBD alone.

Reductions in *Faecalibacterium* in combination with increases in *Bacteroides* as seen in our results is connected to specific enterotype, *Bacteroides II*, which previously been connected to inflammation and is found in IBD and IBD-PSC, with many CD patients falling in this enterotype (Vieira-Silva et al., 2019).

Given the fact that the majority of our PSC-IBD patients have UC rather than CD, these results match the results by (Vieira-Silva et al., 2019), with a larger portion of PSC-IBD patients presenting with this enterotype compared to the UC group. This strengthens the hypothesis suggesting increased inflammation in PSC-IBD.

Lastly, we see enrichment of metabolite sets connected to bacteria indicated in disease of the oral cavity, such as *Dialister pneumosintes*,* Fusobacterium nucleatum*,* Scardovia wiggsiae* in connection with *Streptococcus mutans*,* Streptococcus oralis and Streptococcus gordonii* (Kressirer et al., 2017; Huh & Roh, 2020; Moore & Moore, 1994).

Potential increased presence of these bacteria in the gut may indicate translocation from the oral cavity to the gut, where they contribute to dysbiosis (Lapidot et al., 2021). Presence of these bacteria in the gut may be due to transmission through cholangiography, as used equipment such as duodenoscopes regularly test positive for bacterial contamination despite sanitation (Lapidot et al., 2021; Effenberger et al., 2023; Visrodia et al., 2014).

Altogether, the results of the pathway enrichment analyses suggest dysbiosis of the gut through increases in inflammatory bacteria alongside decreases in bacteria important to gut homeostasis, which may induce a leaky gut and consequently lead to inflammation of the biliary tract. Subsequent liver inflammation may result in the amino-acid centric changes seen in the KEGG and MFN enrichment results. However, given the nature of these experiments, and the fact that they are based on an untargeted blood metabolic signature, both targeted metabolomics and metagenomics validation experiments are required to separate cause and consequence.

Overall, using blood plasma metabolomic data collected through DI-MS, we built a functional predictive model for PSC-IBD that may form a first step towards a low-cost and non-invasive diagnostic model for PSC in IBD patients. Next to predicted changes in amino acid and vitamin metabolism, our analyses predict increases in pro-inflammatory and decreases in protective bacterial subsets. By uploading the raw data and rich metadata connected to this study to *DataverseNL* we aim to contribute to future open IBD and/or PSC research.

## Methods

### Cohort

The cohort was composed of 348 unique patients (Table 1). About half were female (173, 49.7%) and median age at inclusion was 49.1 years (IQR 39.4–57.5). Forty-five patients (12.9%) were active smokers. Twenty patients (5,7%) were diagnosed with IBD unclassified (IBD-U), 159 (45.7%) with UC, 169 (48.6%) with CD. PSC was present in 28 patients (8% of patients, 11% of samples, Tables 1 and 2).

The overall methodology is described in Fig. 1. A total of 735 endoscopies were performed with combined plasma sampling. Inflammation was scored endoscopically per colonic segment from 0 to 3. Overall, 432 (58.78%) surveillance endoscopies were without any inflammation (inflammation total score across sectors < = 3) (Table 2).

### Sample collection

Peripheral blood was drawn prior to colonoscopy in 10 mL K2 EDTA vials (*BD Vacutainer® blood collection tubes*,* SKU:366643*). Blood was spun at 3000 rpm for 10 min at 4 degrees Celsius with maximum ramp up and brake speeds. Plasma was aliquoted into vials afterwards and immediately stored in a −80 °C freezer for a maximum of 9 years until measurement.

**Table 1:** Patient cohort subdivided by IBD subtype and PSC

IBD subtype	Cohort	IBD			PSC-IBD		
	*N* = 348	UC, *N* = 138	CD, *N* = 164	IBD-U, *N* = 18	UC, *N* = 21	CD, *N* = 5	IBD-U, *N* = 2
Median age at inclusion (years)	49.1, (39.4–57.5)	50.4 (41.47–57.9)	49.0 (38.42–57.5)	47.0 (38.89–60.8)	44.7 (40.05–50.9)	42.9 (37.01–45.0.01.0)	36.7 (32.85–40.5)
Sex							
♂male	175 (50.3%)	63 (45.65%)	80 (48.78%)	11 (61.11%)	16 (76.19%)	4 (80%)	1 (50%)
♀female	173 (49.7%)	75 (54.35%)	84 (51.22%)	7 (38.89%)	5 (23.81%)	1 (20%)	1 (50%)
PSC subtype							
large	24 (6.9%)				19 (90.48%)	4 (80%)	1 (50%)
small	4 (1.1%)				2 (9.52%)	1 (20%)	1 (50%)
Smoking status							
non-smoker	178 (51.1%)	60 (43.48%)	84 (51.22%)	12 (66.67%)	16 (76.19%)	4 (80%)	2 (100%)
quit	105 (30.2%)	54 (39.13%)	45 (27.44%)	3 (16.67%)	3 (14.29%)		
unknown	20 (5.7%)	10 (7.25%)	9 (5.49%)	1 (5.56%)			
smoker	45 (12.9%)	14 (10.14%)	26 (15.85%)	2 (11.11%)	2 (9.52%)	1 (20%)	
Visit interval							
1 year	62 (17.8%)	13 (16.05%)	27 (25.23%)	2 (22.22%)	15 (100%)	4 (80%)	1 (100%)
2.5 years	71 (20.4%)	30 (37.04%)	36 (33.64%)	5 (55.56%)			
3 years	1 (0.3%)	1 (1.23%)					
5 years	83 (23.9%)	37 (45.68%)	44 (41.12%)	2 (22.22%)			
6 years	1 (0.3%)					1 (20%)	

**Table 2:** Plasma sample overview subdivided by IBD subtype and PSC

IBD subtype	Cohort	IBD			PSC-IBD		
	*N* = 735	UC, *N* = 2761	CD, *N* = 3391	IBD-U, *N* = 361	UC, *N* = 621	CD, *N* = 181	IBD-U, *N* = 41
Inflammation score	1.0 (0.00–10.0)	0.7 (0.00–6.0)	1.2 (0.00–10.0)	0.8 (0.00–3.0)	1.9 (0.00–8.0)	1.3 (0.00–8.0)	3.0 (2.00–4.0)
Inflammation score > = 3							
unknown	53 (7.21%)	17 (6.16%)	26 (7.67%)	3 (8.33%)	5 (8.06%)	2 (11.11%)	
no	432 (58.78%)	175 (63.41%)	199 (58.70%)	18 (50.00%)	30 (48.39%)	10 (55.56%)	
yes	250 (34.01%)	84 (30.43%)	114 (33.63%)	15 (41.67%)	27 (43.55%)	6 (33.33%)	4 (100.00%)
Dysplasia							
no	620 (84.35%)	223 (80.80%)	295 (87.02%)	30 (83.33%)	55 (88.71%)	13 (72.22%)	4 (100.00%)
yes	115 (15.65%)	53 (19.20%)	44 (12.98%)	6 (16.67%)	7 (11.29%)	5 (27.78%)	
Fistulae							
unknown	52 (7.07%)	17 (6.16%)	26 (7.67%)	3 (8.33%)	5 (8.06%)	1 (5.56%)	
no	677 (92.11%)	259 (93.84%)	309 (91.15%)	33 (91.67%)	55 (88.71%)	17 (94.44%)	4 (100.00%)
yes	6 (0.82%)		4 (1.18%)		2 (3.23%)		
Strictures							
unknown	48 (6.53%)	16 (5.80%)	23 (6.78%)	3 (8.33%)	5 (8.06%)	1 (5.56%)	
no	648 (88.16%)	257 (93.12%)	282 (83.19%)	33 (91.67%)	55 (88.71%)	17 (94.44%)	4 (100.00%)
yes	39 (5.31%)	3 (1.09%)	34 (10.03%)		2 (3.23%)		
UDCA use							
yes	69 (9.39%)				51 (82.26%)	15 (83.34%)	3 (75.00%)
no	666 (90.61%)	276 (100%)	339 (100%)1	36 (100%)1	11 (17.74%)	3 (16.67%)	1 (25.00%)

### Data acquisition

To acquire mass spectra for each sample, we applied an adapted version of our in-house DI-MS protocol as described by (de Sain-van der Velden et al., 2017). Rather than the use of blood spot cards, we used plasma samples stored at −80.

For each of the three technical replicates per sample, 20 µl plasma was extracted using 360 µl of methanol after adding 140 µl of Labelled Amino Acid Standard Sets A and B (Cambridge Isotope Laboratories).

After centrifugation and drying of the supernatant, the residue was solubilised and filtered (Fig. 1b). Sample order was randomised, and replicates were then injected into an Advion Triverse Nanomate connected to a Q-Exactive HF high resolution mass spectrometer (Thermo Scientific). Positive mode signal was collected for 3 min, and negative mode was collected for 1.5 min. The resulting Thermo.raw files were stored as individual replicates. Data and metadata will be available on *MetaboLights* upon publication.

### Processing

Samples were processed in accordance with the protocol set up by (Wolthuis et al., 2022). Briefly, the.raw files were converted to mzML, followed by separation of positive and negative mode scans. Alignment was performed based on internal standard m/z values, split into positive and negative mode.

Individual scans were merged into one spectrum per mode, and peak calling was performed using the *MALDIquant* R package. Peaks were then binned at 2 parts per million. The resulting peak tables were used for subsequent analysis alongside batch and injection order. The file processing pipeline for SLURM-supporting compute clusters is available in the *joannawolthuis/MassChecker* GitHub repository.

### Filtering and normalisation

Mass/charge values were excluded from further analysis if more than 20% of samples missed a signal, as recommended by (Bijlsma et al., 2006). Data was normalised using the *MetaboAnalystR* package using quantile normalisation and autoscaling. Subsequently, day-of-run batch effect was corrected using recorded batch and injection order information using the *WaveICA* package (*Figure **S1*).

Lastly, the final dataset was formed by averaging the normalised signal of the technical replicates in MetaboShiny.

### Data splitting for feature selection and machine learning

To build classifiers, a nested cross-validation scheme was set up (Fig. 1c). To avoid information leakage due to samples from a single subject being present in both training and testing folds, the *Scikit-learn* package, *StratifiedGroupKFold* (SGKF) was adapted to R. We first assigned 80% of samples to a fraction to use for machine learning. The remaining fold was used to perform enrichment analysis. Note that the number of samples per fold may vary slightly due to the fact that an individual may contribute multiple samples, which are always kept together to prevent data leakage.

The 80% used for ML training was split in 5 folds, forming the training folds for the signature determination procedure. This 80/20 split followed by 5 folds in the 80% fraction was repeated separately for each tested variable, and was done based on the class balances to ensure a representative class distribution in the ML, cross-validation and enrichment fractions.

In the case of IBD-PSC patients, we adapted this method in order to also evenly divide the ursodeoxycholic acid(UDCA)-negative patients over the various data fractions.

We assign the IBD-only patients to folds using *caret*’s *GroupKFold* function, and then use SGKF on the PSC-positive patients using UCDA treatment information as variable of interest. In this case, 4 folds were defined rather than 5, as only 4 UDCA-negative individuals were available in the ML subset.

### Model building and bootstrapping

The models without feature selection were built using the R *caret* package, specifically the *ranger* Random Forest algorithm with the *mtry* parameter set as the square root of all m/z values (Kuhn, 2008; Wright & Ziegler, 2017).

Separate models were built for each cross-validation fold and the summary Receiver Operator Characteristic (ROC), and Precision-Recall (PR) curves were built using the joined predicted scores of all folds. To further quantify the area under the ROC curve (AUROC), we used the *pROC* package *roc.test* function to determine a significant increase of the experimental model compared to a negative control model with permuted class labels (Robin et al., 2011). P-values resulting from this ROC-comparison were translated to stars as follows: a single asterisk * is used for p-values between 0.05 and 0.01. Triple asterisks *** are reserved for p-values between 0.01 and 0.001. P-values beneath 0.001 are marked by four asterisks ****.

To perform bootstrapping, matching on the amount of samples in the non-UDCA set, we sampled the full dataset 10.000 times. We then calculated the AUROC of these drawn subsets and compared the distribution of these AUC values to the AUC of the non-UDCA patients and calculated the significance through one-sample T-test.

### Feature selection using univariate analyses

To determine a metabolite signature for the PSC-IBD phenotype, we performed t-test and fold-change analyses within each fold, through *MetaboShiny* and calculated the combined V-score (denoted as $V$) for each compound.

$$V~ = ~ - \log 10\left( {p - value} \right)~ \times ~\log 2\left( {fold - change} \right)$$

Correlation of V_PSC_ and V_sex_ was calculated by taking absolute $V$ (|$V$|) for each m/z value and subsequently calculating the Spearman correlation between the resulting rankings for sex and PSC.

### Feature ranking using logistic regression

In addition, within each fold, we performed per-m/z logistic regression predicting PSC-IBD phenotype using the *glm* core function in R. We extracted the Wald statistic (z-value) per m/z value and utilised the absolute z-value (|Z|) in descending order for subsequent ranking.

### Signature determination using random forest

We proceeded with an adapted version of the analysis used in (Wolthuis et al., 2022) to determine the threshold defining the (expanded) m/z signature (Fig. 1c).

Using either V_PSC_ (or Z_PSC_ in logistic regression) we repeatedly built RF models, starting from a model containing all m/z values available in the data. Next, starting at the top 50, we permuted 50 m/z values per step using the *sample* function within R. This led to many ML models containing an increasing amount of permuted data. Alongside permutation based on ranking, we also permuted 50 random m/z values as a negative control, and built models with permuted class labels as a second negative control.

The resulting AUROC vs. permuted m/z values can be used for signature determination. To do so, the elbow point of the *geom_smooth* fit was estimated by performing gradient descent to find the flattest part of the curve. The resulting amount of permuted m/z values defined the upper threshold of the metabolic signature.

### Enrichment

To gain biological insights for each question, we used the signature threshold resulting from the analysis performed on the ~ 80% feature selection partition of the data. We then separately calculated $V$ and/or $Z$ for the ~ 20% partition and used the *mummichog* algorithm built into *MetaboAnalystR* to calculate enrichment of metabolic pathways in the top-ranking m/z values up to the threshold calculated in the feature selection step (Li et al., 2013). We downloaded the KEGG human metabolic pathway (hsa01100), MetaFishNet (MFN) human pathway collection from *MetaboAnalystR*, and KEGG ‘Microbial metabolism in diverse environments’ pathway collection using 19 adducts (Table S1). As an extra step, pathways were removed that contained synthesis of amino acids that cannot be synthesised by humans (essential amino acids).

As a result, pathways and modules involving *biosynthesis* of *histidine*,* isoleucine*,* leucine*,* lysine*,* methionine*,* phenylalanine*,* threonine*,* tryptophan* and *valine* were excluded from further analysis. Mummichog hits were considered significant if the EASE p-value was below or equal to 0.05.

For the enrichment analysis of metabolites connected to the microbiome, we utilised the GUTSY Atlas (Dekkers et al., 2022). We merged the Supplementary Tables connected to the manuscript containing annotations, species and associations, and then filtered the annotations based on the Human Metabolome Database (HMDB) identifier availability.

Metabolite-microorganism associations were filtered by significance, with diversity-adjusted p-values < 0.05 and ϱ values > 0 included in the pathway database creation. Our metabolite database package *MetaDBparse* was used to match HMDB identifiers to molecular formulas and subsequently calculate m/z values (Fig. 5). Adduct calculation was done through the *MetaboAnalystR* implementation of *mummichog*.

### Visualisation

Visualisation was done mainly through *MetaboShiny* and associated packages (Wolthuis et al., 2020). Any graphs not directly supported by MetaboShiny were generated using R, *ggplot*, *plotly* and *metacoder* using the data available through the data export functionality of *MetaboShiny.* The *pathview* package was used to project $V$ onto KEGG diagrams (Luo & Brouwer, 2013).

## Supplementary Information

Below is the link to the electronic supplementary material.

Supplementary Material 1

## Data Availability

Access to the dataset can be requested on DataverseNL (doi: 10.34894/L4EM0D, submission in progress).

## Material availability

MetaboShiny is available on GitHub: https://github.com/joannawolthuis/MetaboShiny. MetaDBparse is available on GitHub: https://github.com/joannawolthuis/MetaDBparse
