# Supplementary material for: Univariate- and machine learning-based plasma metabolite signature differentiates PSC-IBD from IBD and is predicted to be driven by gut microbial changes
Source: Metabolomics. 2026 Mar 28;22(2):44. doi: 10.1007/s11306-026-02420-w (PMC13032943; doi:10.1007/s11306-026-02420-w)
Supplement: Supplementary file 1 — Supplementary Material 1 [file 11306_2026_2420_MOESM1_ESM.docx]

*Supplementary materials for*

# Univariate- and machine learning-based plasma metabolite signature differentiates PSC-IBD from IBD and is predicted to be driven by gut microbial changes

Joanna C. Wolthuis^1,2^, Johannes P.D. Schultheiss^3^, Stefanía Magnúsdóttir^1,6^, Edwin Stigter^1^, Yuen Fung Tang^1^, Judith Jans^4^, Bas Oldenburg^3^*, Jeroen de Ridder^1,2^ *, Saskia van Mil^1^*

^1^Center for Molecular Medicine, University Medical Center Utrecht and Utrecht University, Utrecht, The Netherlands

^2^Oncode Institute, Utrecht, The Netherlands

^3^ Department of Gastroenterology and Hepatology, University Medical Center Utrecht, Utrecht, The Netherlands

*Contributed equally as senior scientists.


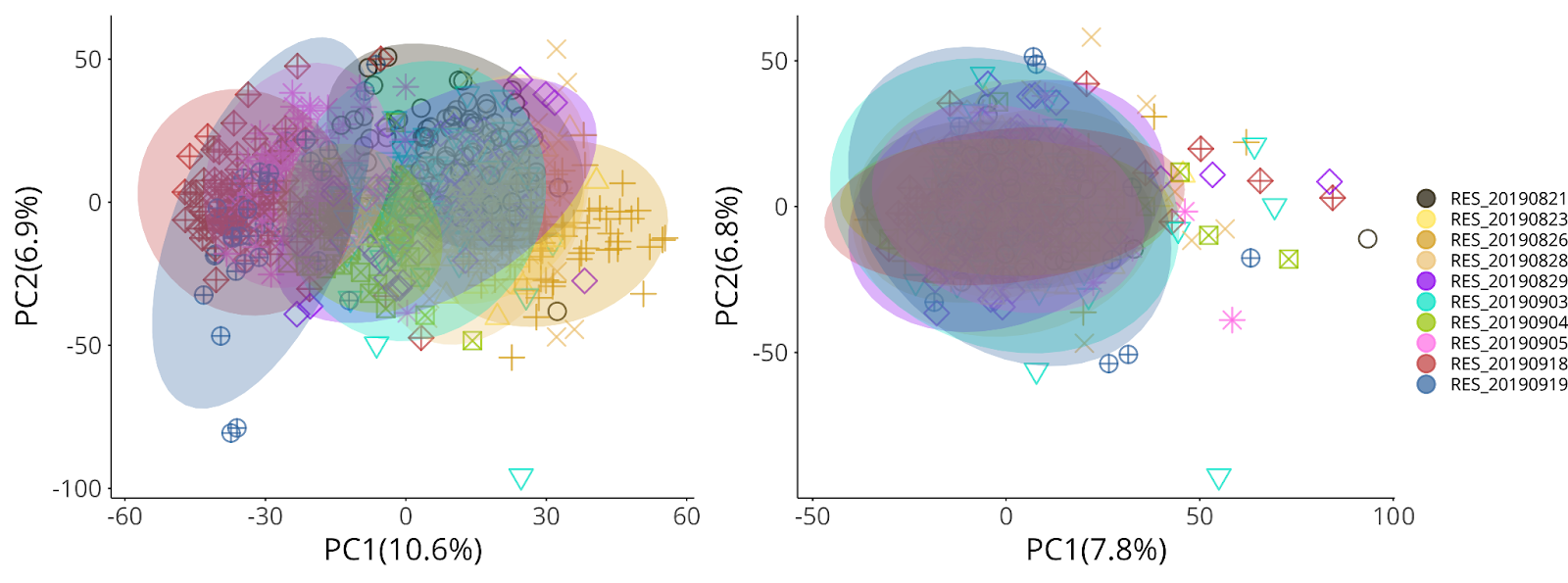


### Figure S1: PCA of batches pre- and post-batch correction using waveICA.


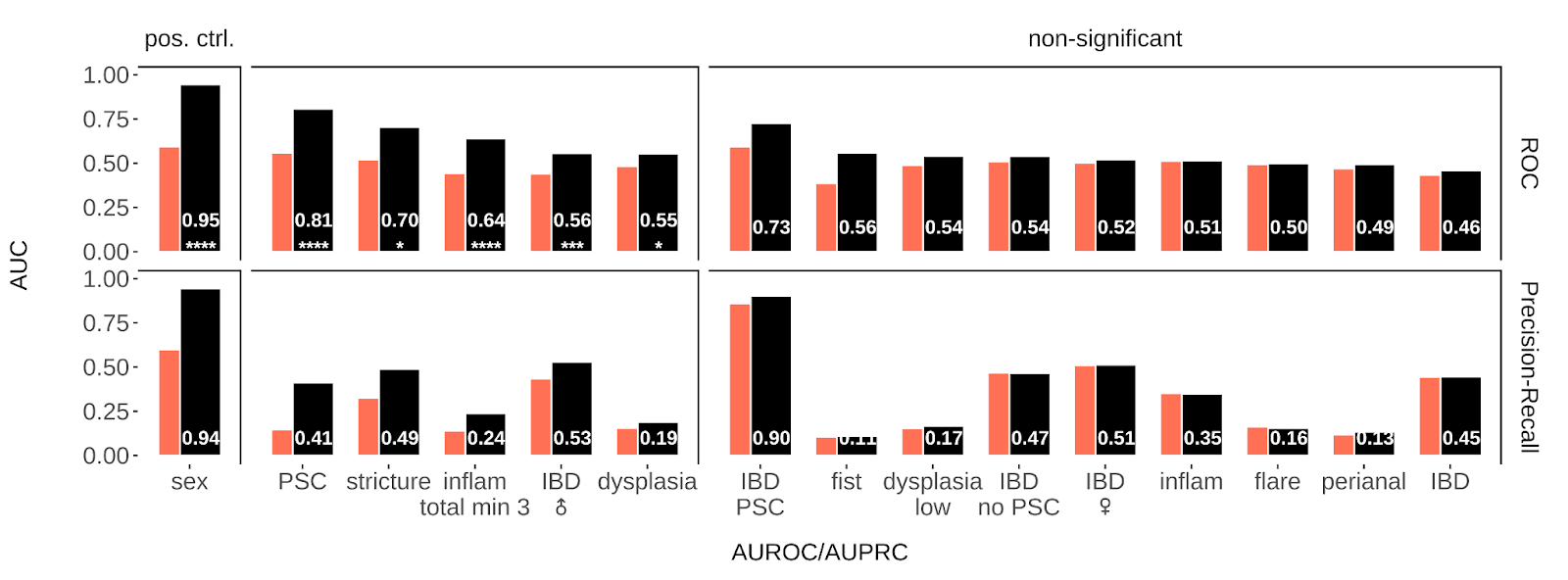


### Figure S2: Performance of ML models of all investigated questions.

Right panel question AUROC did not reach significance according to *pROC’s* significance test comparing the regular label order (right of the twin bars) to the shuffled label order (left). Performance of these models was as follows: IBD type (CD vs. UC) in PSC patients (AUROC: 0.73, AUPRC: 0.80), fistula presence (AUROC: 0.56, AUPRC: 0.11), low-grade dysplasia (AUROC: 0.56, AUPRC: 0.17), IBD type in non-PSC patients (AUROC: 0.54, AUPRC: 0.47), IBD type in female patients (AUROC: 0.54, AUPRC: 0.47), inflammation(inflammation score > 0; AUROC: 0.51, AUPRC: 0.35), flare (AUROC: 0.50, AUPRC: 0.16), perianal disease (AUROC: 0.49, AUPRC: 0.13) and IBD type in all patients (AUROC: 0.46, AUPRC: 0.45)

### Table S1 – Adduct table

| Name | Ion_mode | Charge | AddEx | RemEx | Nelec | Rule |
| --- | --- | --- | --- | --- | --- | --- |
| [M-H+Cl]2- | negative | -2 | Cl1 | H1 | 2 | Ndon>0 AND Nch=0 |
| [M-H+FA]1- | negative | -1 | H2C1O2 | H1 | 1 | Ndon>0 AND Nch=0 |
| [M+H]1+ | positive | 1 | H1 |  | -1 | Nacc>0 AND Nch=0 |
| [M+K]1+ | positive | 1 | K1 |  | -1 | Nacc>0 AND Nch=0 |
| [M+Na]1+ | positive | 1 | Na1 |  | -1 | Nacc>0 AND Nch=0 |
| [M+NH4]1+ | positive | 1 | N1H4 |  | -1 | Nacc>0 AND Nch=0 |
| [M1+.]1+ | positive | 1 |  |  | 0 | Nch=1 |
| [M-H]1- | negative | -1 |  | H1 | 1 | Ndon>0 AND Nch=0 |
| [M+Cl]1- | negative | -1 | Cl1 |  | 1 | Nacc>0 AND Nch=0 |
| [M+NaCl+H]1+ | positive | 1 | Na1Cl1H1 |  | -1 | Nacc>0 AND Nch=0 |
| [M+(NaCl)2+H]1+ | positive | 1 | Na2Cl2H1 |  | -1 | Nacc>0 AND Nch=0 |
| [M+(NaCl)3+H]1+ | positive | 1 | Na3Cl3H1 |  | -1 | Ndon>0 AND Nch=0 |
| [M+(NaCl)4+H]1+ | positive | 1 | Na4Cl4H1 |  | -1 | Nacc>0 AND Nch=0 |
| [M+(NaCl)5+H]1+ | positive | 1 | Na5Cl5H1 |  | -1 | Nacc>0 AND Nch=0 |
| [M+NaCl-H]1- | negative | -1 | Na1Cl1 | H1 | 1 | Ndon>0 AND Nch=0 |
| [M+(NaCl)2-H]1- | negative | -1 | Na2Cl2 | H1 | 1 | Ndon>0 AND Nch=0 |
| [M+(NaCl)3-H]1- | negative | -1 | Na3Cl3 | H1 | 1 | Ndon>0 AND Nch=0 |
| [M+(NaCl)4-H]1- | negative | -1 | Na4Cl4 | H1 | 1 | Ndon>0 AND Nch=0 |
| [M+(NaCl)5-H]1- | negative | -1 | Na5Cl5 | H1 | 1 | Ndon>0 AND Nch=0 |

*Addex = Added atoms

*RemEx = removed atoms

*Nelec = electron amount

### Figure S3

###
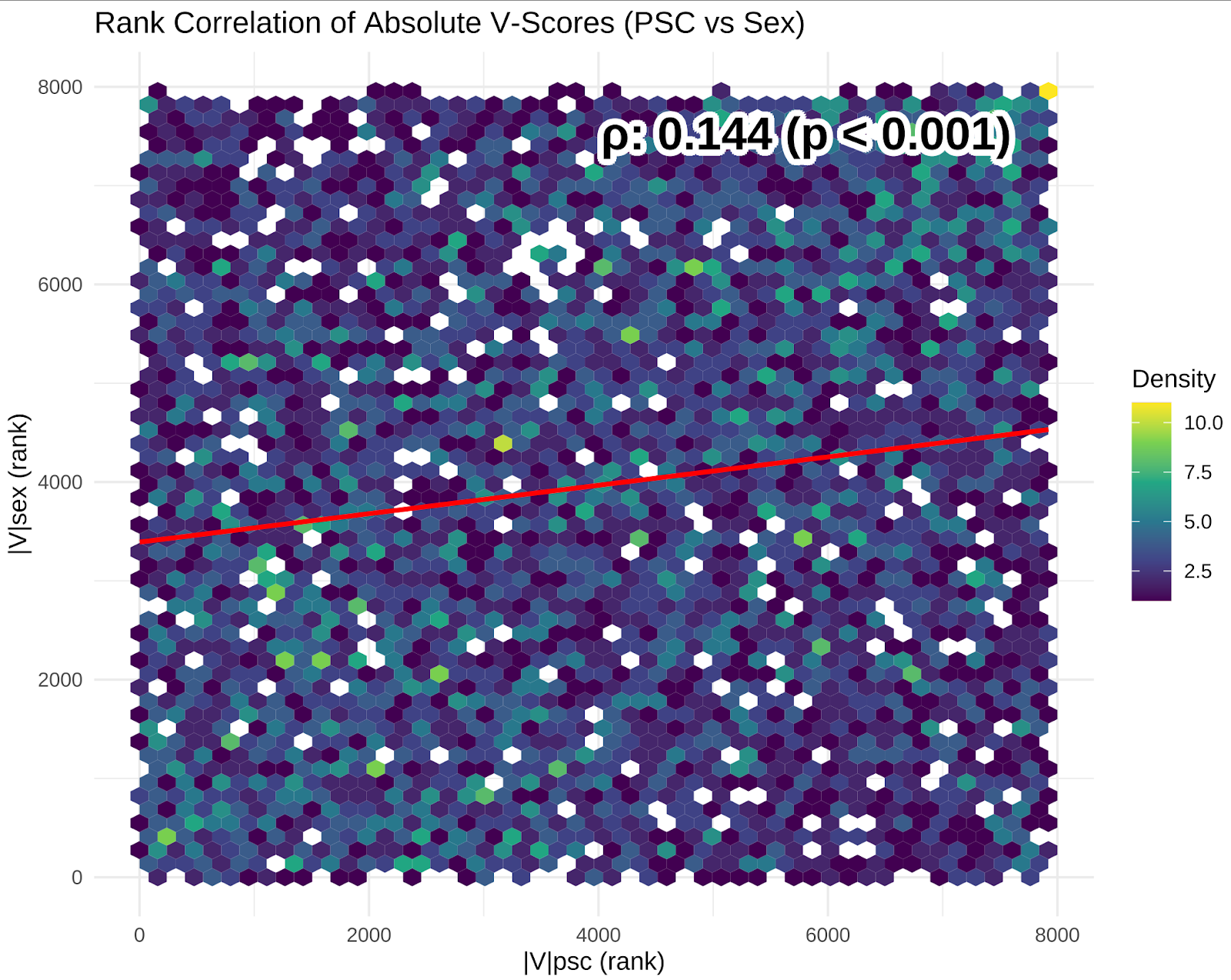


### Table S2 – KEGG Homo sapiens mummichog enrichment results

|  | Pathway total | Hits.total | Hits.sig | Expected | FET | EASE | Gamma | Emp., Hits |
| --- | --- | --- | --- | --- | --- | --- | --- | --- |
| Arginine and proline metabolism | 68 | 40 | 35 | 17.139 | 0.00010924 | 0.00045072 | 0.0030031 | 0 |
| D-Amino acid metabolism | 63 | 55 | 45 | 15.879 | 0.00032906 | 0.00094905 | 0.003007 | 0 |
| Biosynthesis of unsaturated fatty acids | 69 | 14 | 14 | 17.391 | 0.00072802 | 0.0075394 | 0.0030593 | 1 |
| Phenylalanine metabolism | 49 | 34 | 28 | 12.35 | 0.0041325 | 0.011986 | 0.0030951 | 0 |
| Glyoxylate and dicarboxylate metabolism | 61 | 39 | 30 | 15.375 | 0.018317 | 0.040288 | 0.003334 | 0 |
| Pyruvate metabolism | 29 | 20 | 17 | 7.3094 | 0.014986 | 0.047901 | 0.0034017 | 0 |

###

### Table S3 – KEGG Homo sapiens mummichog enrichment matches

|  |  |  |
| --- | --- | --- |
| KEGG ID | Pathway name | Matched m/z [adduct] |
| C00022 | Arginine and proline metabolism, D-Amino acid metabolism, Phenylalanine metabolism, Glyoxylate and dicarboxylate metabolism, Pyruvate metabolism | 111.00526[M+Na]1+, 106.04987[M+NH4]1+, 133.01427[M-H+FA]1-, 144.96737[M+NaCl-H]1-, 202.92616[M+(NaCl)2-H]1-, 87.0088[M-H]1-, 122.98551[M+Cl]1- |
| C00025 | Arginine and proline metabolism, D-Amino acid metabolism, Glyoxylate and dicarboxylate metabolism | 170.04238[M+Na]1+, 186.01634[M+K]1+, 148.06044[M+H]1+, 204.00448[M+NaCl-H]1-, 319.9221[M+(NaCl)3-H]1-, 182.02254[M+Cl]1-, 261.96322[M+(NaCl)2-H]1-, 146.0459[M-H]1- |
| C00026 | D-Amino acid metabolism, Glyoxylate and dicarboxylate metabolism | 262.94591[M+(NaCl)2+H]1+, 320.90454[M+(NaCl)3+H]1+, 378.86309[M+(NaCl)4+H]1+, 436.82163[M+(NaCl)5+H]1+, 145.01427[M-H]1-, 191.01981[M-H+FA]1- |
| C00033 | Glyoxylate and dicarboxylate metabolism, Pyruvate metabolism | 98.98417[M+K]1+, 83.01034[M+Na]1+, 105.01929[M-H+FA]1-, 94.99038[M+Cl]1-, 116.97251[M+NaCl-H]1- |
| C00036 | D-Amino acid metabolism, Glyoxylate and dicarboxylate metabolism, Pyruvate metabolism | 177.00406[M-H+FA]1- |
| C00041 | D-Amino acid metabolism | 128.01083[M+K]1+, 112.03688[M+Na]1+, 90.05497[M+H]1+, 134.04592[M-H+FA]1-, 124.01703[M+Cl]1-, 88.04043[M-H]1-, 145.99895[M+NaCl-H]1-, 203.95768[M+(NaCl)2-H]1- |
| C00042 | Phenylalanine metabolism, Glyoxylate and dicarboxylate metabolism, Pyruvate metabolism | 141.01584[M+Na]1+, 156.98982[M+K]1+, 176.99247[M+NaCl+H]1+, 163.02485[M-H+FA]1-, 117.01937[M-H]1-, 174.97791[M+NaCl-H]1-, 152.99597[M+Cl]1- |
| C00047 | D-Amino acid metabolism | 169.09475[M+Na]1+, 147.11281[M+H]1+, 185.06872[M+K]1+, 181.07497[M+Cl]1-, 145.0983[M-H]1- |
| C00048 | Arginine and proline metabolism, Glyoxylate and dicarboxylate metabolism | 118.9986[M-H+FA]1-, 72.9931[M-H]1- |
| C00058 | Glyoxylate and dicarboxylate metabolism, Pyruvate metabolism | 218.87403[M+(NaCl)3-H]1-, 334.79174[M+(NaCl)5-H]1-, 276.83281[M+(NaCl)4-H]1-, 160.91548[M+(NaCl)2-H]1-, 91.00374[M-H+FA]1-, 102.9569[M+NaCl-H]1-, 80.97492[M+Cl]1- |
| C00062 | Arginine and proline metabolism, D-Amino acid metabolism | 197.10093[M+Na]1+, 175.11895[M+H]1+, 173.10437[M-H]1-, 209.08114[M+Cl]1- |
| C00064 | D-Amino acid metabolism, Glyoxylate and dicarboxylate metabolism | 169.05837[M+Na]1+, 147.07644[M+H]1+, 185.03233[M+K]1+, 376.89704[M+(NaCl)4-H]1-, 434.85492[M+(NaCl)5-H]1-, 181.03859[M+Cl]1-, 203.02058[M+NaCl-H]1-, 145.06183[M-H]1-, 318.9382[M+(NaCl)3-H]1- |
| C00065 | D-Amino acid metabolism, Glyoxylate and dicarboxylate metabolism | 164.00833[M+NaCl+H]1+, 128.03181[M+Na]1+, 144.00582[M+K]1+, 106.04987[M+H]1+, 277.91141[M+(NaCl)3-H]1-, 219.95258[M+(NaCl)2-H]1-, 161.99397[M+NaCl-H]1-, 104.03534[M-H]1-, 140.012[M+Cl]1- |
| C00073 | D-Amino acid metabolism | 172.04022[M+Na]1+, 150.05837[M+H]1+, 188.01428[M+K]1+, 184.02024[M+Cl]1-, 148.04382[M-H]1-, 321.91935[M+(NaCl)3-H]1- |
| C00077 | Arginine and proline metabolism, D-Amino acid metabolism | 171.05306[M+K]1+, 249.01483[M+(NaCl)2+H]1+, 133.09717[M+H]1+, 155.07905[M+Na]1+, 131.08261[M-H]1-, 167.05927[M+Cl]1- |
| C00079 | D-Amino acid metabolism, Phenylalanine metabolism | 204.04223[M+K]1+, 188.06823[M+Na]1+, 166.08624[M+H]1+, 164.0716[M-H]1-, 200.04843[M+Cl]1-, 279.98909[M+(NaCl)2-H]1-, 222.03025[M+NaCl-H]1- |
| C00082 | Phenylalanine metabolism | 204.06309[M+Na]1+, 220.03699[M+K]1+, 182.08121[M+H]1+, 226.07223[M-H+FA]1-, 180.06664[M-H]1-, 295.98419[M+(NaCl)2-H]1-, 216.04299[M+Cl]1-, 238.02531[M+NaCl-H]1- |
| C00086 | Arginine and proline metabolism | 98.99551[M+K]1+, 83.02155[M+Na]1+, 78.06619[M+NH4]1+, 95.00173[M+Cl]1- |
| C00122 | Phenylalanine metabolism, Pyruvate metabolism | 134.0448[M+NH4]1+, 232.9354[M+(NaCl)2+H]1+, 115.00368[M-H]1-, 161.00918[M-H+FA]1- |
| C00133 | D-Amino acid metabolism | 128.01083[M+K]1+, 112.03688[M+Na]1+, 90.05497[M+H]1+, 134.04592[M-H+FA]1-, 124.01703[M+Cl]1-, 88.04043[M-H]1-, 145.99895[M+NaCl-H]1-, 203.95768[M+(NaCl)2-H]1- |
| C00134 | Arginine and proline metabolism, D-Amino acid metabolism | 378.90016[M+(NaCl)5+H]1+, 89.10728[M+H]1+ |
| C00135 | D-Amino acid metabolism | 173.10342[M+NH4]1+, 156.07679[M+H]1+, 178.05867[M+Na]1+, 194.03269[M+K]1+, 190.03891[M+Cl]1-, 212.02085[M+NaCl-H]1-, 154.0622[M-H]1- |
| C00148 | Arginine and proline metabolism, D-Amino acid metabolism | 138.0526[M+Na]1+, 116.0706[M+H]1+, 154.02647[M+K]1+, 133.09717[M+NH4]1+, 172.01473[M+NaCl-H]1-, 150.03268[M+Cl]1-, 114.05603[M-H]1-, 160.06156[M-H+FA]1- |
| C00149 | Glyoxylate and dicarboxylate metabolism, Pyruvate metabolism | 157.0108[M+Na]1+, 134.0212[M1+.]1+, 133.01427[M-H]1-, 190.97292[M+NaCl-H]1- |
| C00158 | Glyoxylate and dicarboxylate metabolism | 230.99018[M+K]1+, 215.01617[M+Na]1+, 191.01981[M-H]1-, 248.97839[M+NaCl-H]1-, 364.89598[M+(NaCl)3-H]1-, 306.93734[M+(NaCl)2-H]1- |
| C00160 | Glyoxylate and dicarboxylate metabolism | 99.00527[M+Na]1+, 75.00874[M-H]1-, 110.98544[M+Cl]1-, 132.96739[M+NaCl-H]1- |
| C00166 | D-Amino acid metabolism, Phenylalanine metabolism | 182.08121[M+NH4]1+, 165.05462[M+H]1+, 203.01014[M+K]1+, 187.03667[M+Na]1+, 163.04012[M-H]1- |
| C00168 | Glyoxylate and dicarboxylate metabolism | 127.00045[M+Na]1+, 162.97674[M+NaCl+H]1+, 103.00369[M-H]1- |
| C00186 | Pyruvate metabolism | 128.99486[M+K]1+, 91.03893[M+H]1+, 113.02087[M+Na]1+, 125.00114[M+Cl]1-, 146.98312[M+NaCl-H]1-, 89.0245[M-H]1-, 204.94161[M+(NaCl)2-H]1-, 320.8594[M+(NaCl)4-H]1-, 378.81825[M+(NaCl)5-H]1-, 135.02995[M-H+FA]1-, 262.90035[M+(NaCl)3-H]1- |
| C00188 | D-Amino acid metabolism | 235.9827[M+(NaCl)2+H]1+, 293.94133[M+(NaCl)3+H]1+, 178.02408[M+NaCl+H]1+, 142.0475[M+Na]1+, 120.06552[M+H]1+, 158.02142[M+K]1+, 291.92654[M+(NaCl)3-H]1-, 233.96828[M+(NaCl)2-H]1-, 176.00959[M+NaCl-H]1-, 154.02766[M+Cl]1-, 118.05102[M-H]1- |
| C00209 | Glyoxylate and dicarboxylate metabolism | 206.91985[M+(NaCl)2+H]1+, 148.96118[M+NaCl+H]1+, 146.94667[M+NaCl-H]1-, 88.98811[M-H]1- |
| C00213 | Arginine and proline metabolism | 128.01083[M+K]1+, 112.03688[M+Na]1+, 90.05497[M+H]1+, 134.04592[M-H+FA]1-, 124.01703[M+Cl]1-, 88.04043[M-H]1-, 145.99895[M+NaCl-H]1-, 203.95768[M+(NaCl)2-H]1- |
| C00217 | D-Amino acid metabolism | 170.04238[M+Na]1+, 186.01634[M+K]1+, 148.06044[M+H]1+, 204.00448[M+NaCl-H]1-, 319.9221[M+(NaCl)3-H]1-, 182.02254[M+Cl]1-, 261.96322[M+(NaCl)2-H]1-, 146.0459[M-H]1- |
| C00219 | Biosynthesis of unsaturated fatty acids | 303.23333[M-H]1-, 339.20992[M+Cl]1- |
| C00249 | Biosynthesis of unsaturated fatty acids | 279.22936[M+Na]1+, 295.20328[M+K]1+, 257.24743[M+H]1+, 291.20978[M+Cl]1-, 301.23867[M-H+FA]1-, 255.23309[M-H]1- |
| C00256 | Pyruvate metabolism | 128.99486[M+K]1+, 91.03893[M+H]1+, 113.02087[M+Na]1+, 125.00114[M+Cl]1-, 146.98312[M+NaCl-H]1-, 89.0245[M-H]1-, 204.94161[M+(NaCl)2-H]1-, 320.8594[M+(NaCl)4-H]1-, 378.81825[M+(NaCl)5-H]1-, 135.02995[M-H+FA]1-, 262.90035[M+(NaCl)3-H]1- |
| C00258 | Glyoxylate and dicarboxylate metabolism | 129.01583[M+Na]1+, 164.99248[M+NaCl+H]1+, 280.90964[M+(NaCl)3+H]1+, 222.95103[M+(NaCl)2+H]1+, 105.01929[M-H]1-, 162.97802[M+NaCl-H]1-, 140.99606[M+Cl]1- |
| C00266 | Glyoxylate and dicarboxylate metabolism | 98.98417[M+K]1+, 83.01034[M+Na]1+, 105.01929[M-H+FA]1-, 94.99038[M+Cl]1-, 116.97251[M+NaCl-H]1- |
| C00300 | Arginine and proline metabolism | 154.05869[M+Na]1+, 132.07679[M+H]1+, 170.03266[M+K]1+, 130.06214[M-H]1-, 166.03884[M+Cl]1- |
| C00311 | Glyoxylate and dicarboxylate metabolism | 230.99018[M+K]1+, 215.01617[M+Na]1+, 191.01981[M-H]1-, 248.97839[M+NaCl-H]1-, 364.89598[M+(NaCl)3-H]1-, 306.93734[M+(NaCl)2-H]1- |
| C00315 | Arginine and proline metabolism | 146.16522[M+H]1+ |
| C00417 | Glyoxylate and dicarboxylate metabolism | 212.97947[M+K]1+, 173.0091[M-H]1- |
| C00424 | Pyruvate metabolism | 133.00251[M+NaCl+H]1+, 75.04406[M+H]1+, 97.02601[M+Na]1+, 119.03496[M-H+FA]1-, 73.02949[M-H]1- |
| C00431 | Arginine and proline metabolism, D-Amino acid metabolism | 156.04211[M+K]1+, 140.0682[M+Na]1+, 118.0862[M+H]1+, 231.989[M+(NaCl)2-H]1-, 162.07723[M-H+FA]1-, 152.04834[M+Cl]1-, 174.0303[M+NaCl-H]1-, 116.07172[M-H]1- |
| C00441 | Arginine and proline metabolism | 156.00574[M+K]1+, 140.03182[M+Na]1+, 176.00847[M+NaCl+H]1+, 118.0499[M+H]1+, 116.03533[M-H]1-, 152.01196[M+Cl]1- |
| C00515 | D-Amino acid metabolism | 171.05306[M+K]1+, 249.01483[M+(NaCl)2+H]1+, 133.09717[M+H]1+, 155.07905[M+Na]1+, 131.08261[M-H]1-, 167.05927[M+Cl]1- |
| C00546 | Pyruvate metabolism | 73.02841[M+H]1+, 90.05497[M+NH4]1+, 117.01937[M-H+FA]1-, 71.01379[M-H]1- |
| C00555 | Arginine and proline metabolism | 88.07567[M+H]1+, 126.0315[M+K]1+, 110.05765[M+Na]1+ |
| C00581 | Arginine and proline metabolism | 140.04307[M+Na]1+, 118.06109[M+H]1+ |
| C00596 | Phenylalanine metabolism | 132.06556[M+NH4]1+, 115.03889[M+H]1+, 137.02098[M+Na]1+, 159.02987[M-H+FA]1-, 113.02445[M-H]1- |
| C00601 | Phenylalanine metabolism | 138.09137[M+NH4]1+, 143.04681[M+Na]1+, 121.06477[M+H]1+, 165.05567[M-H+FA]1- |
| C00642 | Phenylalanine metabolism | 153.05463[M+H]1+, 175.03661[M+Na]1+, 151.04002[M-H]1- |
| C00666 | D-Amino acid metabolism | 307.01972[M+(NaCl)2+H]1+, 249.061[M+NaCl+H]1+, 213.08454[M+Na]1+, 189.08815[M-H]1- |
| C00680 | D-Amino acid metabolism | 307.01972[M+(NaCl)2+H]1+, 249.061[M+NaCl+H]1+, 213.08454[M+Na]1+, 189.08815[M-H]1- |
| C00712 | Biosynthesis of unsaturated fatty acids | 321.21899[M+K]1+, 305.24509[M+Na]1+, 283.26312[M+H]1+, 327.25453[M-H+FA]1-, 317.22566[M+Cl]1-, 397.1663[M+(NaCl)2-H]1-, 281.24876[M-H]1- |
| C00739 | D-Amino acid metabolism | 169.09475[M+Na]1+, 147.11281[M+H]1+, 185.06872[M+K]1+, 181.07497[M+Cl]1-, 145.0983[M-H]1- |
| C00740 | D-Amino acid metabolism | 164.00833[M+NaCl+H]1+, 128.03181[M+Na]1+, 144.00582[M+K]1+, 106.04987[M+H]1+, 277.91141[M+(NaCl)3-H]1-, 219.95258[M+(NaCl)2-H]1-, 161.99397[M+NaCl-H]1-, 104.03534[M-H]1-, 140.012[M+Cl]1- |
| C00750 | Arginine and proline metabolism | 203.22305[M+H]1+ |
| C00763 | Arginine and proline metabolism, D-Amino acid metabolism | 138.0526[M+Na]1+, 116.0706[M+H]1+, 154.02647[M+K]1+, 133.09717[M+NH4]1+, 172.01473[M+NaCl-H]1-, 150.03268[M+Cl]1-, 114.05603[M-H]1-, 160.06156[M-H+FA]1- |
| C00791 | Arginine and proline metabolism | 114.06622[M+H]1+, 152.02209[M+K]1+, 136.04819[M+Na]1+, 148.02829[M+Cl]1-, 112.05166[M-H]1- |
| C00792 | D-Amino acid metabolism | 197.10093[M+Na]1+, 175.11895[M+H]1+, 173.10437[M-H]1-, 209.08114[M+Cl]1- |
| C00819 | D-Amino acid metabolism | 169.05837[M+Na]1+, 147.07644[M+H]1+, 185.03233[M+K]1+, 376.89704[M+(NaCl)4-H]1-, 434.85492[M+(NaCl)5-H]1-, 181.03859[M+Cl]1-, 203.02058[M+NaCl-H]1-, 145.06183[M-H]1-, 318.9382[M+(NaCl)3-H]1- |
| C00820 | D-Amino acid metabolism | 235.9827[M+(NaCl)2+H]1+, 293.94133[M+(NaCl)3+H]1+, 178.02408[M+NaCl+H]1+, 142.0475[M+Na]1+, 120.06552[M+H]1+, 158.02142[M+K]1+, 291.92654[M+(NaCl)3-H]1-, 233.96828[M+(NaCl)2-H]1-, 176.00959[M+NaCl-H]1-, 154.02766[M+Cl]1-, 118.05102[M-H]1- |
| C00855 | D-Amino acid metabolism | 172.04022[M+Na]1+, 150.05837[M+H]1+, 188.01428[M+K]1+, 184.02024[M+Cl]1-, 148.04382[M-H]1-, 321.91935[M+(NaCl)3-H]1- |
| C00937 | Pyruvate metabolism | 133.00251[M+NaCl+H]1+, 75.04406[M+H]1+, 97.02601[M+Na]1+, 119.03496[M-H+FA]1-, 73.02949[M-H]1- |
| C00975 | Glyoxylate and dicarboxylate metabolism | 206.96664[M+NaCl+H]1+ |
| C00988 | Glyoxylate and dicarboxylate metabolism | 178.97173[M+Na]1+, 156.98982[M+H]1+ |
| C01035 | Arginine and proline metabolism | 146.09244[M+H]1+, 163.11865[M+NH4]1+, 168.07437[M+Na]1+ |
| C01043 | Arginine and proline metabolism | 132.05294[M1+.]1+, 155.0427[M+Na]1+, 171.01662[M+K]1+, 133.0608[M+H]1+, 150.08738[M+NH4]1+, 246.96343[M+(NaCl)2-H]1-, 131.04622[M-H]1-, 189.00492[M+NaCl-H]1- |
| C01110 | Arginine and proline metabolism, D-Amino acid metabolism | 154.04746[M+Na]1+, 170.02135[M+K]1+, 132.06556[M+H]1+, 166.02763[M+Cl]1-, 130.05096[M-H]1-, 176.05641[M-H+FA]1- |
| C01127 | Glyoxylate and dicarboxylate metabolism | 207.01466[M-H+FA]1-, 161.00918[M-H]1- |
| C01146 | Glyoxylate and dicarboxylate metabolism | 127.00045[M+Na]1+, 162.97674[M+NaCl+H]1+, 103.00369[M-H]1- |
| C01157 | Arginine and proline metabolism, D-Amino acid metabolism | 154.04746[M+Na]1+, 170.02135[M+K]1+, 132.06556[M+H]1+, 166.02763[M+Cl]1-, 130.05096[M-H]1-, 176.05641[M-H+FA]1- |
| C01165 | Arginine and proline metabolism | 154.04746[M+Na]1+, 170.02135[M+K]1+, 132.06556[M+H]1+, 166.02763[M+Cl]1-, 130.05096[M-H]1-, 176.05641[M-H+FA]1- |
| C01198 | Phenylalanine metabolism | 456.86413[M+(NaCl)5+H]1+, 167.07032[M+H]1+, 189.05231[M+Na]1+, 165.05567[M-H]1- |
| C01251 | Pyruvate metabolism | 265.00907[M+NaCl+H]1+, 229.0319[M+Na]1+, 224.07639[M+NH4]1+, 241.01235[M+Cl]1-, 205.03544[M-H]1- |
| C01530 | Biosynthesis of unsaturated fatty acids | 307.26075[M+Na]1+, 517.1131[M+(NaCl)4+H]1+, 323.23457[M+K]1+, 515.0987[M+(NaCl)4-H]1-, 283.26443[M-H]1-, 329.27014[M-H+FA]1-, 319.24133[M+Cl]1- |
| C01595 | Biosynthesis of unsaturated fatty acids | 319.20337[M+K]1+, 303.22943[M+Na]1+, 315.21001[M+Cl]1-, 325.23887[M-H+FA]1-, 279.23312[M-H]1- |
| C01772 | Phenylalanine metabolism | 182.08121[M+NH4]1+, 165.05462[M+H]1+, 203.01014[M+K]1+, 187.03667[M+Na]1+, 163.04012[M-H]1- |
| C01989 | Glyoxylate and dicarboxylate metabolism | 185.04207[M+Na]1+, 162.05248[M1+.]1+, 201.01601[M+K]1+, 180.08656[M+NH4]1+, 163.06011[M+H]1+, 197.0223[M+Cl]1-, 207.0511[M-H+FA]1-, 161.04555[M-H]1- |
| C01990 | Glyoxylate and dicarboxylate metabolism | 380.89004[M+(NaCl)3+H]1+, 322.93145[M+(NaCl)2+H]1+ |
| C02123 | Glyoxylate and dicarboxylate metabolism | 215.03157[M+K]1+, 176.06819[M1+.]1+, 199.05778[M+Na]1+, 221.0666[M-H+FA]1-, 175.06119[M-H]1- |
| C02237 | D-Amino acid metabolism | 152.03183[M+Na]1+, 168.00575[M+K]1+, 130.04993[M+H]1+, 147.07644[M+NH4]1+, 128.03537[M-H]1-, 185.99394[M+NaCl-H]1-, 174.04077[M-H+FA]1-, 243.95258[M+(NaCl)2-H]1- |
| C02265 | D-Amino acid metabolism, Phenylalanine metabolism | 204.04223[M+K]1+, 188.06823[M+Na]1+, 166.08624[M+H]1+, 164.0716[M-H]1-, 200.04843[M+Cl]1-, 279.98909[M+(NaCl)2-H]1-, 222.03025[M+NaCl-H]1- |
| C02488 | Pyruvate metabolism | 185.04207[M+Na]1+, 162.05248[M1+.]1+, 201.01601[M+K]1+, 180.08656[M+NH4]1+, 163.06011[M+H]1+, 197.0223[M+Cl]1-, 207.0511[M-H+FA]1-, 161.04555[M-H]1- |
| C02504 | Pyruvate metabolism | 215.03157[M+K]1+, 176.06819[M1+.]1+, 199.05778[M+Na]1+, 221.0666[M-H+FA]1-, 175.06119[M-H]1- |
| C02505 | Phenylalanine metabolism | 136.07563[M+H]1+, 158.05766[M+Na]1+, 174.03156[M+K]1+, 180.06664[M-H+FA]1- |
| C02565 | Arginine and proline metabolism | 132.07679[M+NH4]1+, 115.05021[M+H]1+, 137.03217[M+Na]1+, 113.03569[M-H]1- |
| C02647 | Arginine and proline metabolism | 130.09752[M+H]1+, 174.08838[M-H+FA]1- |
| C02763 | Phenylalanine metabolism | 182.08121[M+NH4]1+, 165.05462[M+H]1+, 203.01014[M+K]1+, 187.03667[M+Na]1+, 163.04012[M-H]1- |
| C02946 | Arginine and proline metabolism | 146.08117[M+H]1+, 168.06313[M+Na]1+, 184.03708[M+K]1+, 144.06662[M-H]1- |
| C03217 | Glyoxylate and dicarboxylate metabolism | 176.03177[M1+.]1+, 194.06598[M+NH4]1+, 199.02149[M+Na]1+, 175.0248[M-H]1- |
| C03239 | D-Amino acid metabolism | 146.08117[M+H]1+, 168.06313[M+Na]1+, 184.03708[M+K]1+, 144.06662[M-H]1- |
| C03242 | Biosynthesis of unsaturated fatty acids | 305.24902[M-H]1- |
| C03341 | D-Amino acid metabolism | 154.04746[M+Na]1+, 170.02135[M+K]1+, 132.06556[M+H]1+, 166.02763[M+Cl]1-, 130.05096[M-H]1-, 176.05641[M-H+FA]1- |
| C03440 | D-Amino acid metabolism | 154.04746[M+Na]1+, 170.02135[M+K]1+, 132.06556[M+H]1+, 166.02763[M+Cl]1-, 130.05096[M-H]1-, 176.05641[M-H+FA]1- |
| C03459 | Glyoxylate and dicarboxylate metabolism | 206.96664[M+NaCl+H]1+ |
| C03548 | Glyoxylate and dicarboxylate metabolism | 177.00406[M-H+FA]1- |
| C03564 | Arginine and proline metabolism, D-Amino acid metabolism | 136.03701[M+Na]1+, 158.04586[M-H+FA]1-, 112.04042[M-H]1- |
| C03589 | Phenylalanine metabolism | 191.00811[M+NaCl+H]1+, 248.96668[M+(NaCl)2+H]1+, 171.00542[M+K]1+, 150.07609[M+NH4]1+, 155.03145[M+Na]1+, 131.03498[M-H]1-, 177.04047[M-H+FA]1- |

###
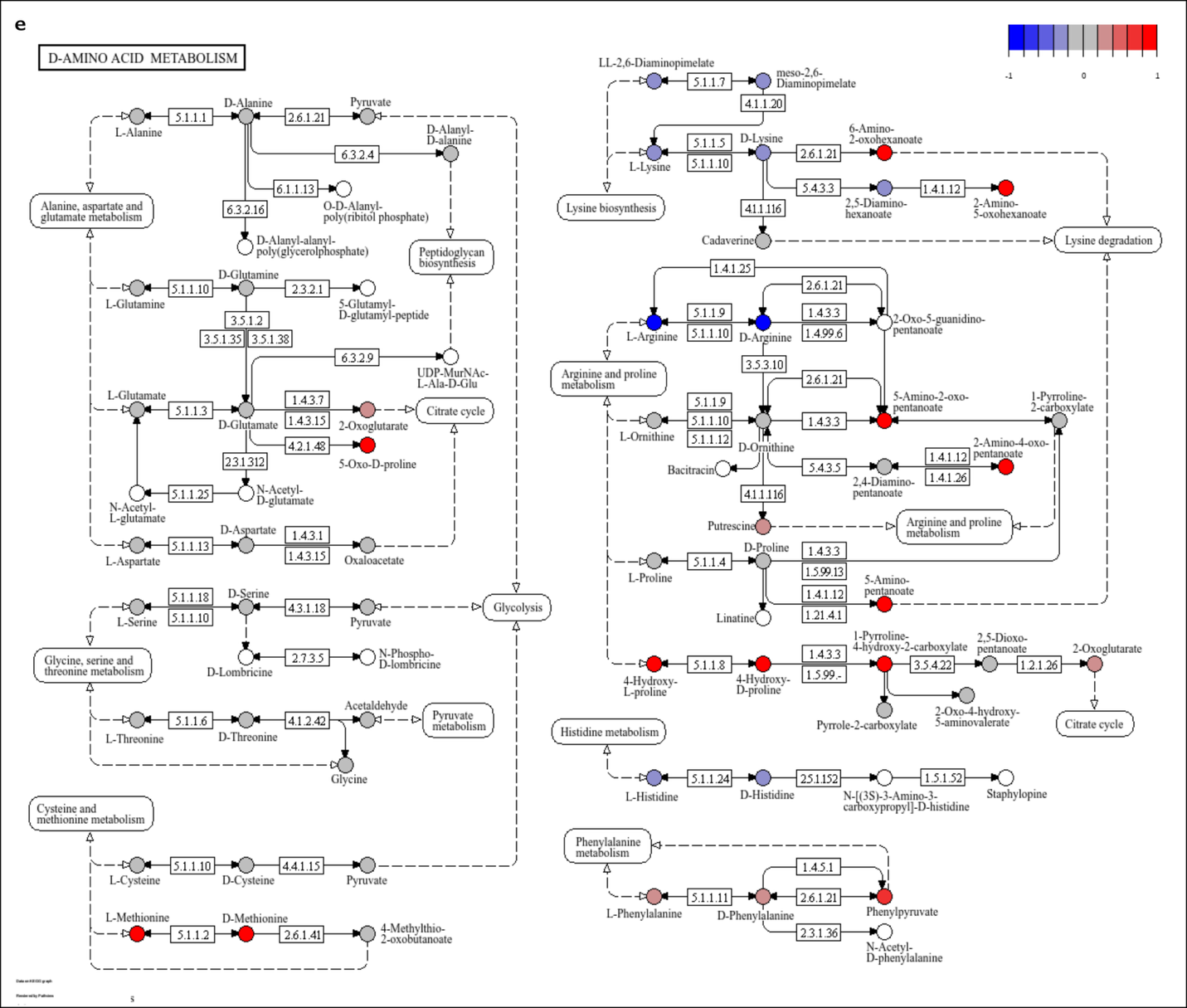


**Figure S4:** V-score projection *homo sapiens* onto KEGG pathway. Red indicates increased relative V-score in PSC-IBD patients relative to IBD-only. Blue is a decrease relative to IBD-only. Grey nodes were found in the dataset but not in m/z signature.

###
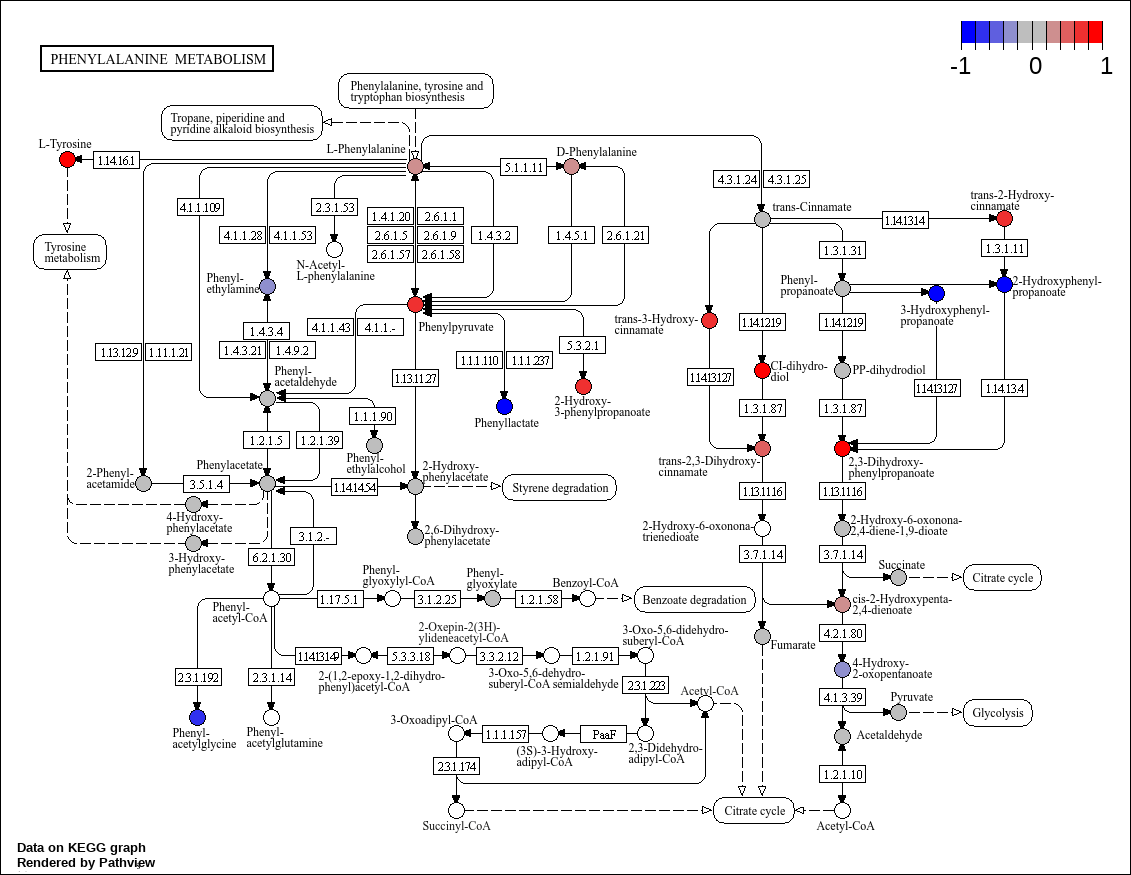


**Figure S5:** V-score projection *homo sapiens* onto KEGG pathway. Red indicates increased relative V-score in PSC-IBD patients relative to IBD-only. Blue is a decrease relative to IBD-only. Grey nodes were found in the dataset but not in m/z signature.

###
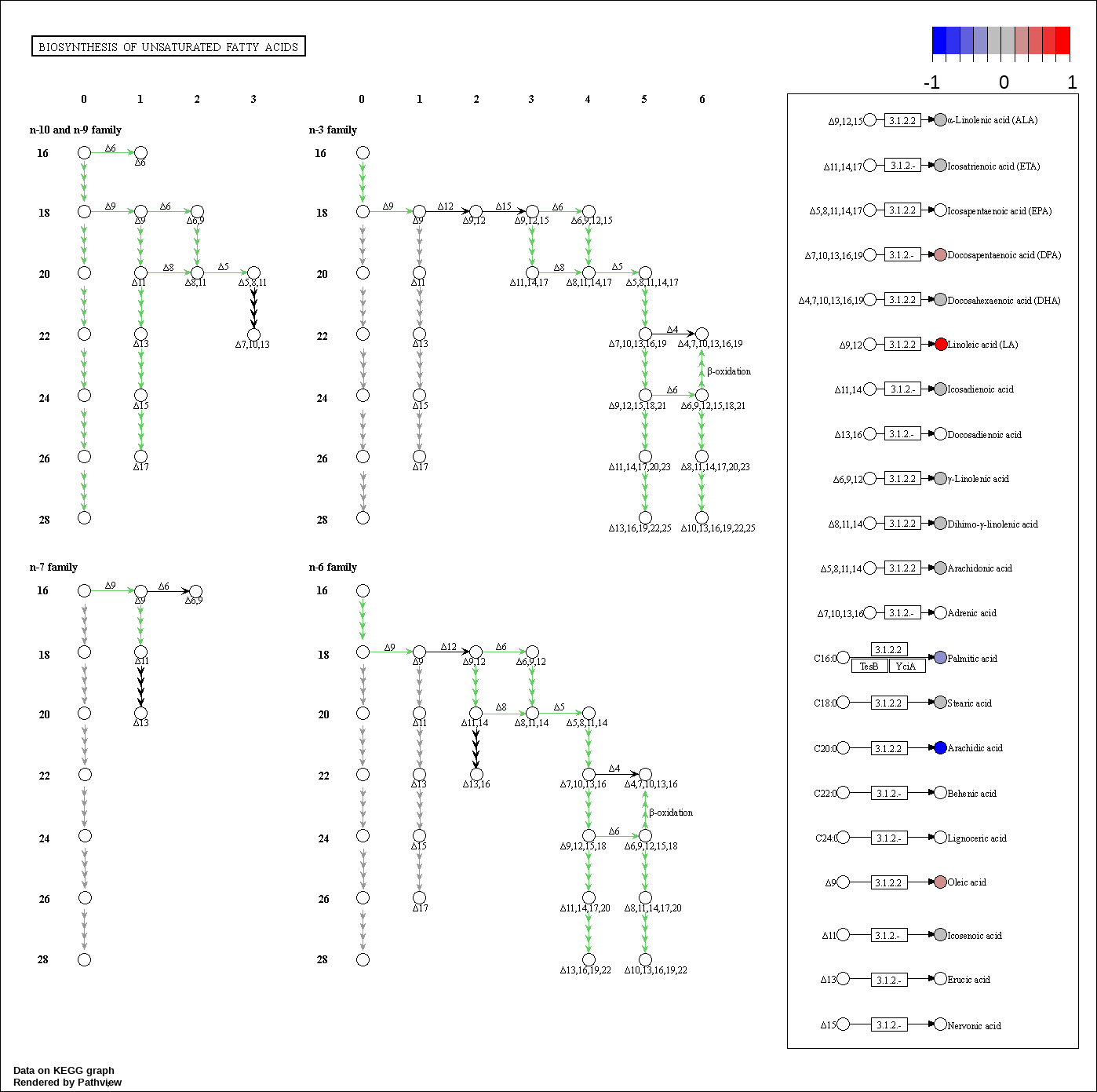


**Figure S6:** V-score projection *homo sapiens* onto KEGG pathway. Red indicates increased relative V-score in PSC-IBD patients relative to IBD-only. Blue is a decrease relative to IBD-only. Grey nodes were found in the dataset but not in m/z signature.

###
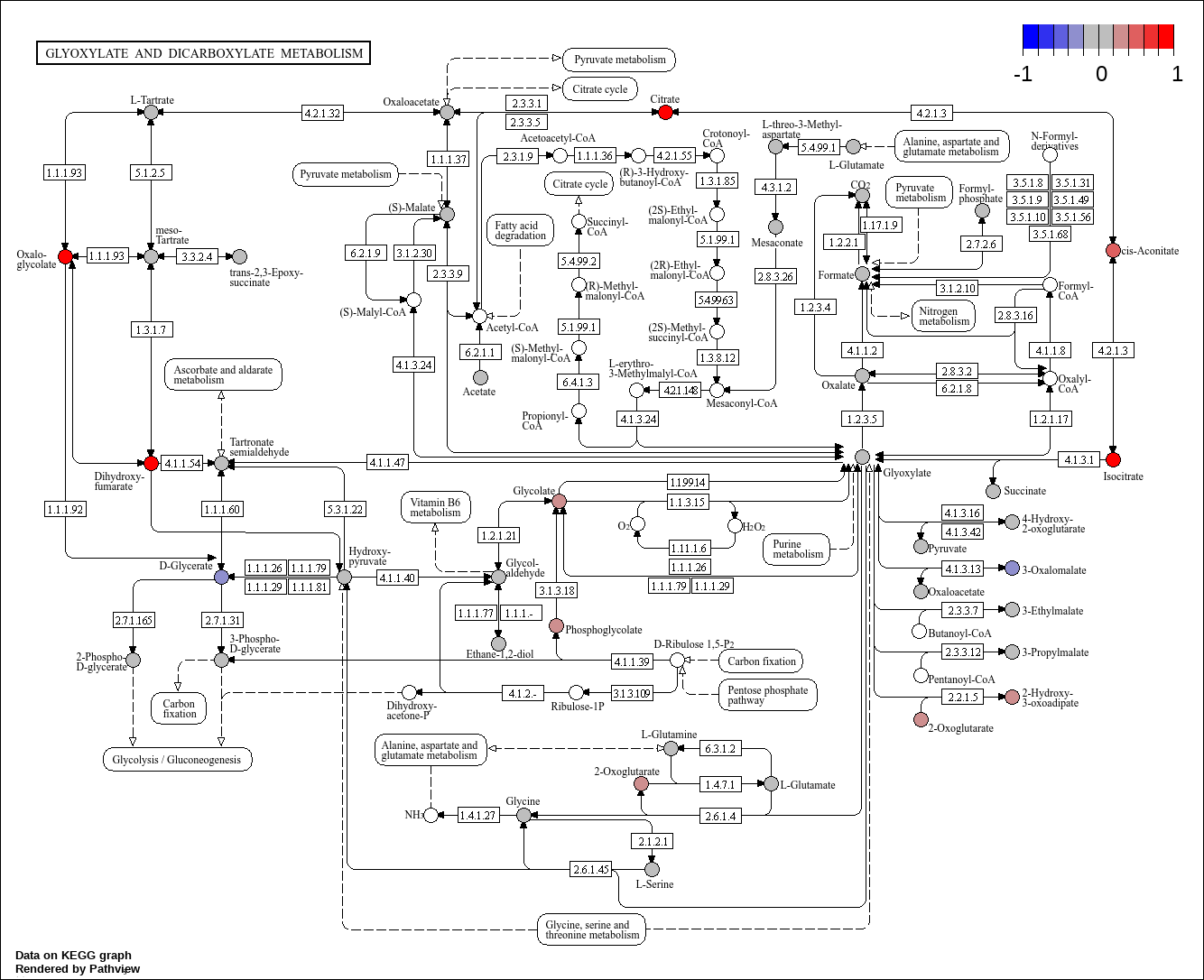


**Figure S8:** V-score projection onto KEGG *homo sapiens* pathway. Red indicates increased relative V-score in PSC-IBD patients relative to IBD-only. Blue indicates a decrease relative to IBD-only. Grey nodes were found in the dataset but not in the PSC m/z signature.

###
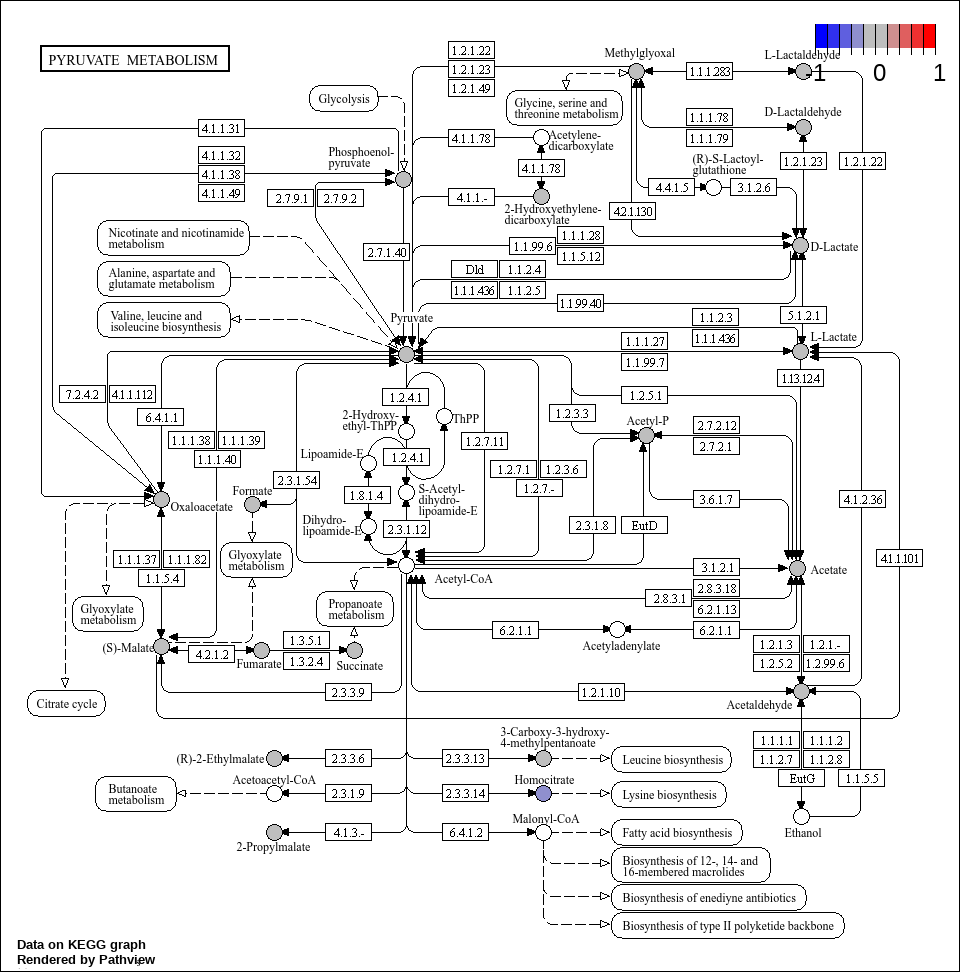


**Figure S8:** V-score projection onto KEGG *homo sapiens* pathway. Red indicates increased relative V-score in PSC-IBD patients relative to IBD-only. Blue indicates a decrease relative to IBD-only. Grey nodes were found in the dataset but not in the PSC m/z signature.

###

###

###

### Table S4 – MFN mummichog enrichment results

|  | Pathway total | Hits.total | Hits.sig | Expected | FET | EASE | Gamma | Emp.Hits |
| --- | --- | --- | --- | --- | --- | --- | --- | --- |
| Arginine and Proline Metabolism | 45 | 30 | 26 | 6.8908 | 0.00045794 | 0.0019048 | 0.0083673 | 0 |
| Aspartate and asparagine metabolism | 114 | 60 | 46 | 17.457 | 0.00092429 | 0.0022366 | 0.0083724 | 0 |
| Lysine metabolism | 52 | 20 | 18 | 7.9627 | 0.0015518 | 0.007601 | 0.008455 | 0 |
| Glycine, serine, alanine and threonine metabolism | 88 | 46 | 34 | 13.475 | 0.012344 | 0.026126 | 0.0087474 | 0 |
| Ascorbate (Vitamin C) and Aldarate Metabolism | 29 | 17 | 15 | 4.4407 | 0.0063246 | 0.026343 | 0.0087509 | 0 |
| De novo fatty acid biosynthesis | 106 | 13 | 12 | 16.232 | 0.007182 | 0.036388 | 0.0089145 | 1 |
| Vitamin B3 (nicotinate and nicotinamide) metabolism | 28 | 13 | 12 | 4.2876 | 0.007182 | 0.036388 | 0.0089145 | 0 |

### Table S5 – MFN mummichog enrichment matches

| Compound identifier | Pathway name | Matches [adduct] |
| --- | --- | --- |
| 1mncam | Vitamin B3 (nicotinate and nicotinamide) metabolism | 160.06041[M+Na]1+, 137.07099[M1+.]1+ |
| C00022 | Arginine and Proline Metabolism, Glycine, serine, alanine and threonine metabolism | 111.00526[M+Na]1+, 106.04987[M+NH4]1+, 133.01427[M-H+FA]1-, 144.96737[M+NaCl-H]1-, 202.92616[M+(NaCl)2-H]1-, 87.0088[M-H]1-, 122.98551[M+Cl]1- |
| C00025 | Arginine and Proline Metabolism, Aspartate and asparagine metabolism, Lysine metabolism, Glycine, serine, alanine and threonine metabolism, Vitamin B3 (nicotinate and nicotinamide) metabolism | 170.04238[M+Na]1+, 186.01634[M+K]1+, 148.06044[M+H]1+, 204.00448[M+NaCl-H]1-, 319.9221[M+(NaCl)3-H]1-, 182.02254[M+Cl]1-, 261.96322[M+(NaCl)2-H]1-, 146.0459[M-H]1- |
| C00026 | Arginine and Proline Metabolism, Aspartate and asparagine metabolism, Lysine metabolism, Glycine, serine, alanine and threonine metabolism | 262.94591[M+(NaCl)2+H]1+, 320.90454[M+(NaCl)3+H]1+, 378.86309[M+(NaCl)4+H]1+, 436.82163[M+(NaCl)5+H]1+, 145.01427[M-H]1-, 191.01981[M-H+FA]1- |
| C00033 | Aspartate and asparagine metabolism, Glycine, serine, alanine and threonine metabolism | 98.98417[M+K]1+, 83.01034[M+Na]1+, 105.01929[M-H+FA]1-, 94.99038[M+Cl]1-, 116.97251[M+NaCl-H]1- |
| C00036 | Arginine and Proline Metabolism, Aspartate and asparagine metabolism | 177.00406[M-H+FA]1- |
| C00041 | Glycine, serine, alanine and threonine metabolism | 128.01083[M+K]1+, 112.03688[M+Na]1+, 90.05497[M+H]1+, 134.04592[M-H+FA]1-, 124.01703[M+Cl]1-, 88.04043[M-H]1-, 145.99895[M+NaCl-H]1-, 203.95768[M+(NaCl)2-H]1- |
| C00042 | Arginine and Proline Metabolism, Lysine metabolism | 141.01584[M+Na]1+, 156.98982[M+K]1+, 176.99247[M+NaCl+H]1+, 163.02485[M-H+FA]1-, 117.01937[M-H]1-, 174.97791[M+NaCl-H]1-, 152.99597[M+Cl]1- |
| C00047 | Aspartate and asparagine metabolism, Lysine metabolism | 169.09475[M+Na]1+, 147.11281[M+H]1+, 185.06872[M+K]1+, 181.07497[M+Cl]1-, 145.0983[M-H]1- |
| C00048 | Arginine and Proline Metabolism, Glycine, serine, alanine and threonine metabolism | 118.9986[M-H+FA]1-, 72.9931[M-H]1- |
| C00058 | Arginine and Proline Metabolism, Aspartate and asparagine metabolism | 218.87403[M+(NaCl)3-H]1-, 334.79174[M+(NaCl)5-H]1-, 276.83281[M+(NaCl)4-H]1-, 160.91548[M+(NaCl)2-H]1-, 91.00374[M-H+FA]1-, 102.9569[M+NaCl-H]1-, 80.97492[M+Cl]1- |
| C00062 | Arginine and Proline Metabolism, Aspartate and asparagine metabolism, Glycine, serine, alanine and threonine metabolism, Vitamin B3 (nicotinate and nicotinamide) metabolism | 197.10093[M+Na]1+, 175.11895[M+H]1+, 173.10437[M-H]1-, 209.08114[M+Cl]1- |
| C00064 | Arginine and Proline Metabolism, Aspartate and asparagine metabolism, Vitamin B3 (nicotinate and nicotinamide) metabolism | 169.05837[M+Na]1+, 147.07644[M+H]1+, 185.03233[M+K]1+, 376.89704[M+(NaCl)4-H]1-, 434.85492[M+(NaCl)5-H]1-, 181.03859[M+Cl]1-, 203.02058[M+NaCl-H]1-, 145.06183[M-H]1-, 318.9382[M+(NaCl)3-H]1- |
| C00065 | Glycine, serine, alanine and threonine metabolism | 164.00833[M+NaCl+H]1+, 128.03181[M+Na]1+, 144.00582[M+K]1+, 106.04987[M+H]1+, 277.91141[M+(NaCl)3-H]1-, 219.95258[M+(NaCl)2-H]1-, 161.99397[M+NaCl-H]1-, 104.03534[M-H]1-, 140.012[M+Cl]1- |
| C00072 | Ascorbate (Vitamin C) and Aldarate Metabolism | 176.03177[M1+.]1+, 194.06598[M+NH4]1+, 199.02149[M+Na]1+, 175.0248[M-H]1- |
| C00073 | Arginine and Proline Metabolism, Glycine, serine, alanine and threonine metabolism | 172.04022[M+Na]1+, 150.05837[M+H]1+, 188.01428[M+K]1+, 184.02024[M+Cl]1-, 148.04382[M-H]1-, 321.91935[M+(NaCl)3-H]1- |
| C00077 | Aspartate and asparagine metabolism, Glycine, serine, alanine and threonine metabolism | 171.05306[M+K]1+, 249.01483[M+(NaCl)2+H]1+, 133.09717[M+H]1+, 155.07905[M+Na]1+, 131.08261[M-H]1-, 167.05927[M+Cl]1- |
| C00086 | Arginine and Proline Metabolism, Aspartate and asparagine metabolism | 98.99551[M+K]1+, 83.02155[M+Na]1+, 78.06619[M+NH4]1+, 95.00173[M+Cl]1- |
| C00109 | Aspartate and asparagine metabolism, Glycine, serine, alanine and threonine metabolism | 103.03896[M+H]1+, 140.99485[M+K]1+, 125.02096[M+Na]1+, 120.06552[M+NH4]1+, 137.00098[M+Cl]1-, 147.02988[M-H+FA]1-, 158.98303[M+NaCl-H]1-, 101.02439[M-H]1- |
| C00122 | Arginine and Proline Metabolism | 134.0448[M+NH4]1+, 232.9354[M+(NaCl)2+H]1+, 115.00368[M-H]1-, 161.00918[M-H+FA]1- |
| C00133 | Glycine, serine, alanine and threonine metabolism | 128.01083[M+K]1+, 112.03688[M+Na]1+, 90.05497[M+H]1+, 134.04592[M-H+FA]1-, 124.01703[M+Cl]1-, 88.04043[M-H]1-, 145.99895[M+NaCl-H]1-, 203.95768[M+(NaCl)2-H]1- |
| C00134 | Arginine and Proline Metabolism, Aspartate and asparagine metabolism | 378.90016[M+(NaCl)5+H]1+, 89.10728[M+H]1+ |
| C00148 | Arginine and Proline Metabolism, Aspartate and asparagine metabolism | 138.0526[M+Na]1+, 116.0706[M+H]1+, 154.02647[M+K]1+, 133.09717[M+NH4]1+, 172.01473[M+NaCl-H]1-, 150.03268[M+Cl]1-, 114.05603[M-H]1-, 160.06156[M-H+FA]1- |
| C00152 | Aspartate and asparagine metabolism | 132.05294[M1+.]1+, 155.0427[M+Na]1+, 171.01662[M+K]1+, 133.0608[M+H]1+, 150.08738[M+NH4]1+, 246.96343[M+(NaCl)2-H]1-, 131.04622[M-H]1-, 189.00492[M+NaCl-H]1- |
| C00153 | Vitamin B3 (nicotinate and nicotinamide) metabolism | 145.03729[M+Na]1+, 123.05524[M+H]1+ |
| C00155 | Glycine, serine, alanine and threonine metabolism | 158.02466[M+Na]1+, 173.9985[M+K]1+ |
| C00160 | Glycine, serine, alanine and threonine metabolism | 99.00527[M+Na]1+, 75.00874[M-H]1-, 110.98544[M+Cl]1-, 132.96739[M+NaCl-H]1- |
| C00168 | Glycine, serine, alanine and threonine metabolism | 127.00045[M+Na]1+, 162.97674[M+NaCl+H]1+, 103.00369[M-H]1- |
| C00188 | Glycine, serine, alanine and threonine metabolism | 235.9827[M+(NaCl)2+H]1+, 293.94133[M+(NaCl)3+H]1+, 178.02408[M+NaCl+H]1+, 142.0475[M+Na]1+, 120.06552[M+H]1+, 158.02142[M+K]1+, 291.92654[M+(NaCl)3-H]1-, 233.96828[M+(NaCl)2-H]1-, 176.00959[M+NaCl-H]1-, 154.02766[M+Cl]1-, 118.05102[M-H]1- |
| C00191 | Ascorbate (Vitamin C) and Aldarate Metabolism | 217.03196[M+Na]1+, 212.07639[M+NH4]1+, 193.03537[M-H]1-, 229.012[M+Cl]1- |
| C00209 | Ascorbate (Vitamin C) and Aldarate Metabolism | 206.91985[M+(NaCl)2+H]1+, 148.96118[M+NaCl+H]1+, 146.94667[M+NaCl-H]1-, 88.98811[M-H]1- |
| C00213 | Glycine, serine, alanine and threonine metabolism | 128.01083[M+K]1+, 112.03688[M+Na]1+, 90.05497[M+H]1+, 134.04592[M-H+FA]1-, 124.01703[M+Cl]1-, 88.04043[M-H]1-, 145.99895[M+NaCl-H]1-, 203.95768[M+(NaCl)2-H]1- |
| C00219 | De novo fatty acid biosynthesis | 303.23333[M-H]1-, 339.20992[M+Cl]1- |
| C00232 | Aspartate and asparagine metabolism | 103.03896[M+H]1+, 140.99485[M+K]1+, 125.02096[M+Na]1+, 120.06552[M+NH4]1+, 137.00098[M+Cl]1-, 147.02988[M-H+FA]1-, 158.98303[M+NaCl-H]1-, 101.02439[M-H]1- |
| C00249 | De novo fatty acid biosynthesis | 279.22936[M+Na]1+, 295.20328[M+K]1+, 257.24743[M+H]1+, 291.20978[M+Cl]1-, 301.23867[M-H+FA]1-, 255.23309[M-H]1- |
| C00253 | Vitamin B3 (nicotinate and nicotinamide) metabolism | 122.0248[M-H]1-, 168.03019[M-H+FA]1- |
| C00256 | Glycine, serine, alanine and threonine metabolism | 128.99486[M+K]1+, 91.03893[M+H]1+, 113.02087[M+Na]1+, 125.00114[M+Cl]1-, 146.98312[M+NaCl-H]1-, 89.0245[M-H]1-, 204.94161[M+(NaCl)2-H]1-, 320.8594[M+(NaCl)4-H]1-, 378.81825[M+(NaCl)5-H]1-, 135.02995[M-H+FA]1-, 262.90035[M+(NaCl)3-H]1- |
| C00258 | Glycine, serine, alanine and threonine metabolism | 129.01583[M+Na]1+, 164.99248[M+NaCl+H]1+, 280.90964[M+(NaCl)3+H]1+, 222.95103[M+(NaCl)2+H]1+, 105.01929[M-H]1-, 162.97802[M+NaCl-H]1-, 140.99606[M+Cl]1- |
| C00300 | Glycine, serine, alanine and threonine metabolism | 154.05869[M+Na]1+, 132.07679[M+H]1+, 170.03266[M+K]1+, 130.06214[M-H]1-, 166.03884[M+Cl]1- |
| C00315 | Aspartate and asparagine metabolism | 146.16522[M+H]1+ |
| C00318 | Lysine metabolism | 162.11244[M+H]1+, 200.06838[M+K]1+, 184.09445[M+Na]1+, 196.07462[M+Cl]1- |
| C00322 | Lysine metabolism | 183.02646[M+Na]1+, 276.96158[M+(NaCl)2+H]1+, 160.03687[M1+.]1+, 159.02987[M-H]1-, 205.03544[M-H+FA]1- |
| C00327 | Arginine and Proline Metabolism, Aspartate and asparagine metabolism | 198.08497[M+Na]1+, 349.9795[M+(NaCl)3+H]1+, 176.10295[M+H]1+, 292.02074[M+(NaCl)2+H]1+, 214.05884[M+K]1+, 174.08838[M-H]1-, 290.0059[M+(NaCl)2-H]1-, 232.04696[M+NaCl-H]1-, 210.06513[M+Cl]1- |
| C00408 | Lysine metabolism | 152.06821[M+Na]1+, 147.11281[M+NH4]1+, 168.04216[M+K]1+, 130.08631[M+H]1+ |
| C00425 | Ascorbate (Vitamin C) and Aldarate Metabolism | 212.97947[M+K]1+, 173.0091[M-H]1- |
| C00430 | Glycine, serine, alanine and threonine metabolism | 154.04746[M+Na]1+, 170.02135[M+K]1+, 132.06556[M+H]1+, 166.02763[M+Cl]1-, 130.05096[M-H]1-, 176.05641[M-H+FA]1- |
| C00437 | Aspartate and asparagine metabolism | 192.13422[M+NH4]1+, 197.08973[M+Na]1+, 173.09315[M-H]1- |
| C00442 | Vitamin B3 (nicotinate and nicotinamide) metabolism | 229.012[M-H]1-, 286.97071[M+NaCl-H]1- |
| C00449 | Lysine metabolism | 299.12136[M+Na]1+ |
| C00450 | Lysine metabolism | 128.07058[M+H]1+, 145.09715[M+NH4]1+, 150.05259[M+Na]1+ |
| C00487 | Aspartate and asparagine metabolism, Lysine metabolism | 162.11244[M1+.]1+ |
| C00546 | Glycine, serine, alanine and threonine metabolism | 73.02841[M+H]1+, 90.05497[M+NH4]1+, 117.01937[M-H+FA]1-, 71.01379[M-H]1- |
| C00555 | Arginine and Proline Metabolism, Aspartate and asparagine metabolism | 88.07567[M+H]1+, 126.0315[M+K]1+, 110.05765[M+Na]1+ |
| C00581 | Glycine, serine, alanine and threonine metabolism | 140.04307[M+Na]1+, 118.06109[M+H]1+ |
| C00601 | Ascorbate (Vitamin C) and Aldarate Metabolism | 138.09137[M+NH4]1+, 143.04681[M+Na]1+, 121.06477[M+H]1+, 165.05567[M-H+FA]1- |
| C00612 | Aspartate and asparagine metabolism | 188.17576[M+H]1+ |
| C00712 | De novo fatty acid biosynthesis | 321.21899[M+K]1+, 305.24509[M+Na]1+, 283.26312[M+H]1+, 327.25453[M-H+FA]1-, 317.22566[M+Cl]1-, 397.1663[M+(NaCl)2-H]1-, 281.24876[M-H]1- |
| C00719 | Glycine, serine, alanine and threonine metabolism | 156.04211[M+K]1+, 140.0682[M+Na]1+, 118.0862[M+H]1+, 231.989[M+(NaCl)2-H]1-, 162.07723[M-H+FA]1-, 152.04834[M+Cl]1-, 174.0303[M+NaCl-H]1-, 116.07172[M-H]1- |
| C00725 | Ascorbate (Vitamin C) and Aldarate Metabolism | 265.00907[M+NaCl+H]1+, 207.05068[M+H]1+ |
| C00750 | Aspartate and asparagine metabolism | 203.22305[M+H]1+ |
| C00763 | Arginine and Proline Metabolism | 138.0526[M+Na]1+, 116.0706[M+H]1+, 154.02647[M+K]1+, 133.09717[M+NH4]1+, 172.01473[M+NaCl-H]1-, 150.03268[M+Cl]1-, 114.05603[M-H]1-, 160.06156[M-H+FA]1- |
| C00800 | Ascorbate (Vitamin C) and Aldarate Metabolism | 219.04749[M+Na]1+, 196.0581[M1+.]1+, 241.0565[M-H+FA]1-, 195.05102[M-H]1- |
| C00937 | Glycine, serine, alanine and threonine metabolism | 133.00251[M+NaCl+H]1+, 75.04406[M+H]1+, 97.02601[M+Na]1+, 119.03496[M-H+FA]1-, 73.02949[M-H]1- |
| C00956 | Lysine metabolism | 184.05806[M+Na]1+, 200.03206[M+K]1+, 162.07609[M+H]1+, 160.06156[M-H]1- |
| C00988 | Glycine, serine, alanine and threonine metabolism | 178.97173[M+Na]1+, 156.98982[M+H]1+ |
| C01029 | Aspartate and asparagine metabolism | 188.17576[M+H]1+ |
| C01035 | Aspartate and asparagine metabolism | 146.09244[M+H]1+, 163.11865[M+NH4]1+, 168.07437[M+Na]1+ |
| C01041 | Ascorbate (Vitamin C) and Aldarate Metabolism | 193.05838[M+NH4]1+, 176.03177[M+H]1+ |
| C01127 | Arginine and Proline Metabolism | 207.01466[M-H+FA]1-, 161.00918[M-H]1- |
| C01146 | Glycine, serine, alanine and threonine metabolism | 127.00045[M+Na]1+, 162.97674[M+NaCl+H]1+, 103.00369[M-H]1- |
| C01157 | Arginine and Proline Metabolism, Aspartate and asparagine metabolism | 154.04746[M+Na]1+, 170.02135[M+K]1+, 132.06556[M+H]1+, 166.02763[M+Cl]1-, 130.05096[M-H]1-, 176.05641[M-H+FA]1- |
| C01165 | Arginine and Proline Metabolism, Aspartate and asparagine metabolism | 154.04746[M+Na]1+, 170.02135[M+K]1+, 132.06556[M+H]1+, 166.02763[M+Cl]1-, 130.05096[M-H]1-, 176.05641[M-H+FA]1- |
| C01181 | Lysine metabolism | 146.11758[M1+.]1+ |
| C01530 | De novo fatty acid biosynthesis | 307.26075[M+Na]1+, 517.1131[M+(NaCl)4+H]1+, 323.23457[M+K]1+, 515.0987[M+(NaCl)4-H]1-, 283.26443[M-H]1-, 329.27014[M-H+FA]1-, 319.24133[M+Cl]1- |
| C01595 | De novo fatty acid biosynthesis | 319.20337[M+K]1+, 303.22943[M+Na]1+, 315.21001[M+Cl]1-, 325.23887[M-H+FA]1-, 279.23312[M-H]1- |
| C01620 | Ascorbate (Vitamin C) and Aldarate Metabolism | 195.00309[M+NaCl+H]1+, 192.98853[M+NaCl-H]1-, 135.02995[M-H]1- |
| C01879 | Aspartate and asparagine metabolism | 152.03183[M+Na]1+, 168.00575[M+K]1+, 130.04993[M+H]1+, 147.07644[M+NH4]1+, 128.03537[M-H]1-, 185.99394[M+NaCl-H]1-, 174.04077[M-H+FA]1-, 243.95258[M+(NaCl)2-H]1- |
| C01888 | Glycine, serine, alanine and threonine metabolism | 112.01589[M+K]1+, 96.04195[M+Na]1+, 74.06007[M+H]1+, 118.05102[M-H+FA]1- |
| C02045 | Ascorbate (Vitamin C) and Aldarate Metabolism | 143.03153[M+Na]1+, 159.00544[M+K]1+, 119.03496[M-H]1-, 155.01164[M+Cl]1-, 165.04037[M-H+FA]1-, 176.99351[M+NaCl-H]1- |
| C02238 | Aspartate and asparagine metabolism | 152.03183[M+Na]1+, 168.00575[M+K]1+, 130.04993[M+H]1+, 147.07644[M+NH4]1+, 128.03537[M-H]1-, 185.99394[M+NaCl-H]1-, 174.04077[M-H+FA]1-, 243.95258[M+(NaCl)2-H]1- |
| C02571 | Aspartate and asparagine metabolism | 263.08899[M+NaCl+H]1+, 204.12306[M1+.]1+, 239.09254[M+Cl]1- |
| C02630 | Aspartate and asparagine metabolism | 171.02645[M+Na]1+, 148.03692[M1+.]1+, 193.03537[M-H+FA]1-, 147.02988[M-H]1- |
| C02918 | Vitamin B3 (nicotinate and nicotinamide) metabolism | 160.06041[M+Na]1+, 137.07099[M1+.]1+ |
| C02946 | Arginine and Proline Metabolism, Aspartate and asparagine metabolism | 146.08117[M+H]1+, 168.06313[M+Na]1+, 184.03708[M+K]1+, 144.06662[M-H]1- |
| C03232 | Glycine, serine, alanine and threonine metabolism | 206.96664[M+Na]1+, 240.92884[M+NaCl-H]1-, 298.88754[M+(NaCl)2-H]1-, 414.80506[M+(NaCl)4-H]1-, 356.8466[M+(NaCl)3-H]1- |
| C03239 | Lysine metabolism | 146.08117[M+H]1+, 168.06313[M+Na]1+, 184.03708[M+K]1+, 144.06662[M-H]1- |
| C03242 | De novo fatty acid biosynthesis | 305.24902[M-H]1- |
| C03440 | Aspartate and asparagine metabolism | 154.04746[M+Na]1+, 170.02135[M+K]1+, 132.06556[M+H]1+, 166.02763[M+Cl]1-, 130.05096[M-H]1-, 176.05641[M-H+FA]1- |
| C03508 | Glycine, serine, alanine and threonine metabolism | 156.00574[M+K]1+, 140.03182[M+Na]1+, 176.00847[M+NaCl+H]1+, 118.0499[M+H]1+, 116.03533[M-H]1-, 152.01196[M+Cl]1- |
| C03564 | Arginine and Proline Metabolism | 136.03701[M+Na]1+, 158.04586[M-H+FA]1-, 112.04042[M-H]1- |
| C03722 | Vitamin B3 (nicotinate and nicotinamide) metabolism | 166.01454[M-H]1- |
| C03740 | Aspartate and asparagine metabolism | 241.07951[M+Na]1+, 218.08997[M1+.]1+, 217.08281[M-H]1- |
| C03793 | Lysine metabolism | 189.15974[M+H]1+, 211.14166[M+Na]1+ |
| C03912 | Arginine and Proline Metabolism | 136.03701[M+Na]1+, 158.04586[M-H+FA]1-, 112.04042[M-H]1- |
| C04076 | Lysine metabolism | 146.08117[M+H]1+, 168.06313[M+Na]1+, 184.03708[M+K]1+, 144.06662[M-H]1- |
| C04092 | Lysine metabolism | 128.07058[M+H]1+, 145.09715[M+NH4]1+, 150.05259[M+Na]1+ |
| C04281 | Arginine and Proline Metabolism, Aspartate and asparagine metabolism | 152.03183[M+Na]1+, 168.00575[M+K]1+, 130.04993[M+H]1+, 147.07644[M+NH4]1+, 128.03537[M-H]1-, 185.99394[M+NaCl-H]1-, 174.04077[M-H+FA]1-, 243.95258[M+(NaCl)2-H]1- |
| C04282 | Aspartate and asparagine metabolism | 152.03183[M+Na]1+, 168.00575[M+K]1+, 130.04993[M+H]1+, 147.07644[M+NH4]1+, 128.03537[M-H]1-, 185.99394[M+NaCl-H]1-, 174.04077[M-H+FA]1-, 243.95258[M+(NaCl)2-H]1- |
| C04575 | Ascorbate (Vitamin C) and Aldarate Metabolism | 230.99018[M+K]1+, 215.01617[M+Na]1+, 191.01981[M-H]1-, 248.97839[M+NaCl-H]1-, 364.89598[M+(NaCl)3-H]1-, 306.93734[M+(NaCl)2-H]1- |
| C05411 | Ascorbate (Vitamin C) and Aldarate Metabolism | 184.08167[M+NH4]1+, 166.04748[M1+.]1+, 165.04037[M-H]1- |
| C05412 | Ascorbate (Vitamin C) and Aldarate Metabolism | 184.08167[M+NH4]1+, 166.04748[M1+.]1+, 165.04037[M-H]1- |
| C05519 | Glycine, serine, alanine and threonine metabolism | 235.9827[M+(NaCl)2+H]1+, 293.94133[M+(NaCl)3+H]1+, 178.02408[M+NaCl+H]1+, 142.0475[M+Na]1+, 120.06552[M+H]1+, 158.02142[M+K]1+, 291.92654[M+(NaCl)3-H]1-, 233.96828[M+(NaCl)2-H]1-, 176.00959[M+NaCl-H]1-, 154.02766[M+Cl]1-, 118.05102[M-H]1- |
| C05543 | Lysine metabolism | 146.11758[M+H]1+, 168.09951[M+Na]1+, 184.07337[M+K]1+ |
| C05545 | Lysine metabolism | 175.14409[M+H]1+, 197.12616[M+Na]1+ |
| C05572 | Aspartate and asparagine metabolism | 144.03026[M-H]1-, 190.03578[M-H+FA]1- |
| C05829 | Aspartate and asparagine metabolism | 225.0281[M+Cl]1- |
| C05840 | Vitamin B3 (nicotinate and nicotinamide) metabolism | 247.94626[M+(NaCl)2+H]1+, 189.98769[M+NaCl+H]1+ |
| C05842 | Vitamin B3 (nicotinate and nicotinamide) metabolism | 153.06581[M+H]1+, 170.0924[M+NH4]1+, 175.04782[M+Na]1+, 151.05123[M-H]1-, 187.02799[M+Cl]1-, 197.05676[M-H+FA]1- |
| C05843 | Vitamin B3 (nicotinate and nicotinamide) metabolism | 153.06581[M+H]1+, 170.0924[M+NH4]1+, 175.04782[M+Na]1+, 151.05123[M-H]1-, 187.02799[M+Cl]1-, 197.05676[M-H+FA]1- |
| C05932 | Aspartate and asparagine metabolism | 231.07398[M1+.]1+, 254.0634[M+Na]1+ |
| C05936 | Arginine and Proline Metabolism, Aspartate and asparagine metabolism | 152.06821[M+Na]1+, 147.11281[M+NH4]1+, 168.04216[M+K]1+, 130.08631[M+H]1+ |
| C05938 | Arginine and Proline Metabolism, Aspartate and asparagine metabolism | 170.04238[M+Na]1+, 186.01634[M+K]1+, 148.06044[M+H]1+, 204.00448[M+NaCl-H]1-, 319.9221[M+(NaCl)3-H]1-, 182.02254[M+Cl]1-, 261.96322[M+(NaCl)2-H]1-, 146.0459[M-H]1- |
| C05946 | Aspartate and asparagine metabolism | 207.01466[M-H+FA]1-, 161.00918[M-H]1- |
| C06424 | De novo fatty acid biosynthesis | 287.17478[M+NaCl+H]1+, 251.19805[M+Na]1+, 267.17192[M+K]1+, 273.20725[M-H+FA]1-, 263.17842[M+Cl]1-, 227.20173[M-H]1- |
| C06425 | De novo fatty acid biosynthesis | 335.29197[M+Na]1+ |
| C06426 | De novo fatty acid biosynthesis | 323.22309[M-H+FA]1-, 313.1943[M+Cl]1-, 277.21739[M-H]1- |
| C06427 | De novo fatty acid biosynthesis | 323.22309[M-H+FA]1-, 313.1943[M+Cl]1-, 277.21739[M-H]1- |
| C06429 | De novo fatty acid biosynthesis | 351.2294[M+Na]1+, 367.20321[M+K]1+, 363.21028[M+Cl]1-, 327.23343[M-H]1- |
| CE1059 | Aspartate and asparagine metabolism | 132.07471[M-H+Cl]2- |
| CE1938 | Aspartate and asparagine metabolism | 255.13159[M+Na]1+ |
| CE1943 | Aspartate and asparagine metabolism | 144.10193[M1+.]1+, 201.05359[M+NaCl-H]1- |
| CE2026 | Glycine, serine, alanine and threonine metabolism | 215.03157[M+NaCl+H]1+, 195.0289[M+K]1+, 213.01728[M+NaCl-H]1- |
| CE2510 | De novo fatty acid biosynthesis | 542.1209[M+(NaCl)4+H]1+ |
| CE4788 | Aspartate and asparagine metabolism | 138.0526[M+Na]1+, 116.0706[M+H]1+, 154.02647[M+K]1+, 133.09717[M+NH4]1+, 172.01473[M+NaCl-H]1-, 150.03268[M+Cl]1-, 114.05603[M-H]1-, 160.06156[M-H+FA]1- |
| CE4968 | Glycine, serine, alanine and threonine metabolism | 159.08931[M+H]1+, 158.08108[M1+.]1+, 217.04722[M+NaCl+H]1+, 272.99154[M+(NaCl)2-H]1-, 330.95048[M+(NaCl)3-H]1-, 215.03291[M+NaCl-H]1-, 388.9094[M+(NaCl)4-H]1-, 446.86732[M+(NaCl)5-H]1- |
| CE4970 | Glycine, serine, alanine and threonine metabolism | 159.08931[M+H]1+, 158.08108[M1+.]1+, 217.04722[M+NaCl+H]1+, 272.99154[M+(NaCl)2-H]1-, 330.95048[M+(NaCl)3-H]1-, 215.03291[M+NaCl-H]1-, 388.9094[M+(NaCl)4-H]1-, 446.86732[M+(NaCl)5-H]1- |
| CE5586 | Aspartate and asparagine metabolism | 146.16522[M+H]1+ |
| CE5985 | Ascorbate (Vitamin C) and Aldarate Metabolism | 143.03153[M+Na]1+, 159.00544[M+K]1+, 119.03496[M-H]1-, 155.01164[M+Cl]1-, 165.04037[M-H+FA]1-, 176.99351[M+NaCl-H]1- |
| glac | Ascorbate (Vitamin C) and Aldarate Metabolism | 176.03177[M1+.]1+, 194.06598[M+NH4]1+, 199.02149[M+Na]1+, 175.0248[M-H]1- |
|  |  |  |

###

### Table S6 – KEGG ‘microbial metabolism in diverse environments’ mummichog enrichment results

|  | Pathway total | Hits.total | Hits.sig | Expected | FET | EASE | Gamma | Emp.Hits |
| --- | --- | --- | --- | --- | --- | --- | --- | --- |
| Phenylalanine metabolism | 49 | 34 | 28 | 15.981 | 0.0029346 | 0.0087911 | 0.0025647 | 0 |
| Styrene degradation | 24 | 20 | 18 | 7.8275 | 0.0025728 | 0.011764 | 0.0025859 | 0 |
| Glyoxylate and dicarboxylate metabolism | 61 | 39 | 30 | 19.895 | 0.01347 | 0.030565 | 0.0027247 | 1 |
| Pyruvate metabolism | 29 | 20 | 17 | 9.4583 | 0.01211 | 0.039938 | 0.0027968 | 0 |
| Lysine degradation | 45 | 35 | 27 | 14.677 | 0.017924 | 0.040991 | 0.002805 | 2 |

| Table S7 – KEGG ‘microbial metabolism in diverse environments’ mummichog enrichment matches | | |
| --- | --- | --- |
| Compound identifier | Pathway name | Matched m/z [adduct] |
| C00022 | Phenylalanine metabolism, Glyoxylate and dicarboxylate metabolism, Pyruvate metabolism | 111.00526[M+Na]1+, 106.04987[M+NH4]1+, 133.01427[M-H+FA]1-, 144.96737[M+NaCl-H]1-, 202.92616[M+(NaCl)2-H]1-, 87.0088[M-H]1-, 122.98551[M+Cl]1- |
| C00025 | Glyoxylate and dicarboxylate metabolism | 170.04238[M+Na]1+, 186.01634[M+K]1+, 148.06044[M+H]1+, 204.00448[M+NaCl-H]1-, 319.9221[M+(NaCl)3-H]1-, 182.02254[M+Cl]1-, 261.96322[M+(NaCl)2-H]1-, 146.0459[M-H]1- |
| C00026 | Glyoxylate and dicarboxylate metabolism, Lysine degradation | 262.94591[M+(NaCl)2+H]1+, 320.90454[M+(NaCl)3+H]1+, 378.86309[M+(NaCl)4+H]1+, 436.82163[M+(NaCl)5+H]1+, 145.01427[M-H]1-, 191.01981[M-H+FA]1- |
| C00033 | Glyoxylate and dicarboxylate metabolism, Pyruvate metabolism | 98.98417[M+K]1+, 83.01034[M+Na]1+, 105.01929[M-H+FA]1-, 94.99038[M+Cl]1-, 116.97251[M+NaCl-H]1- |
| C00036 | Glyoxylate and dicarboxylate metabolism, Pyruvate metabolism | 177.00406[M-H+FA]1- |
| C00042 | Phenylalanine metabolism, Glyoxylate and dicarboxylate metabolism, Pyruvate metabolism, Lysine degradation | 141.01584[M+Na]1+, 156.98982[M+K]1+, 176.99247[M+NaCl+H]1+, 163.02485[M-H+FA]1-, 117.01937[M-H]1-, 174.97791[M+NaCl-H]1-, 152.99597[M+Cl]1- |
| C00047 | Lysine degradation | 169.09475[M+Na]1+, 147.11281[M+H]1+, 185.06872[M+K]1+, 181.07497[M+Cl]1-, 145.0983[M-H]1- |
| C00048 | Glyoxylate and dicarboxylate metabolism | 118.9986[M-H+FA]1-, 72.9931[M-H]1- |
| C00058 | Glyoxylate and dicarboxylate metabolism, Pyruvate metabolism | 218.87403[M+(NaCl)3-H]1-, 334.79174[M+(NaCl)5-H]1-, 276.83281[M+(NaCl)4-H]1-, 160.91548[M+(NaCl)2-H]1-, 91.00374[M-H+FA]1-, 102.9569[M+NaCl-H]1-, 80.97492[M+Cl]1- |
| C00064 | Glyoxylate and dicarboxylate metabolism | 169.05837[M+Na]1+, 147.07644[M+H]1+, 185.03233[M+K]1+, 376.89704[M+(NaCl)4-H]1-, 434.85492[M+(NaCl)5-H]1-, 181.03859[M+Cl]1-, 203.02058[M+NaCl-H]1-, 145.06183[M-H]1-, 318.9382[M+(NaCl)3-H]1- |
| C00065 | Glyoxylate and dicarboxylate metabolism | 164.00833[M+NaCl+H]1+, 128.03181[M+Na]1+, 144.00582[M+K]1+, 106.04987[M+H]1+, 277.91141[M+(NaCl)3-H]1-, 219.95258[M+(NaCl)2-H]1-, 161.99397[M+NaCl-H]1-, 104.03534[M-H]1-, 140.012[M+Cl]1- |
| C00079 | Phenylalanine metabolism | 204.04223[M+K]1+, 188.06823[M+Na]1+, 166.08624[M+H]1+, 164.0716[M-H]1-, 200.04843[M+Cl]1-, 279.98909[M+(NaCl)2-H]1-, 222.03025[M+NaCl-H]1- |
| C00082 | Phenylalanine metabolism | 204.06309[M+Na]1+, 220.03699[M+K]1+, 182.08121[M+H]1+, 226.07223[M-H+FA]1-, 180.06664[M-H]1-, 295.98419[M+(NaCl)2-H]1-, 216.04299[M+Cl]1-, 238.02531[M+NaCl-H]1- |
| C00122 | Phenylalanine metabolism, Styrene degradation, Pyruvate metabolism | 134.0448[M+NH4]1+, 232.9354[M+(NaCl)2+H]1+, 115.00368[M-H]1-, 161.00918[M-H+FA]1- |
| C00149 | Glyoxylate and dicarboxylate metabolism, Pyruvate metabolism | 157.0108[M+Na]1+, 134.0212[M1+.]1+, 133.01427[M-H]1-, 190.97292[M+NaCl-H]1- |
| C00158 | Glyoxylate and dicarboxylate metabolism | 230.99018[M+K]1+, 215.01617[M+Na]1+, 191.01981[M-H]1-, 248.97839[M+NaCl-H]1-, 364.89598[M+(NaCl)3-H]1-, 306.93734[M+(NaCl)2-H]1- |
| C00160 | Glyoxylate and dicarboxylate metabolism | 99.00527[M+Na]1+, 75.00874[M-H]1-, 110.98544[M+Cl]1-, 132.96739[M+NaCl-H]1- |
| C00164 | Styrene degradation, Lysine degradation | 103.03896[M+H]1+, 140.99485[M+K]1+, 125.02096[M+Na]1+, 120.06552[M+NH4]1+, 137.00098[M+Cl]1-, 147.02988[M-H+FA]1-, 158.98303[M+NaCl-H]1-, 101.02439[M-H]1- |
| C00166 | Phenylalanine metabolism | 182.08121[M+NH4]1+, 165.05462[M+H]1+, 203.01014[M+K]1+, 187.03667[M+Na]1+, 163.04012[M-H]1- |
| C00168 | Glyoxylate and dicarboxylate metabolism | 127.00045[M+Na]1+, 162.97674[M+NaCl+H]1+, 103.00369[M-H]1- |
| C00186 | Styrene degradation, Pyruvate metabolism | 128.99486[M+K]1+, 91.03893[M+H]1+, 113.02087[M+Na]1+, 125.00114[M+Cl]1-, 146.98312[M+NaCl-H]1-, 89.0245[M-H]1-, 204.94161[M+(NaCl)2-H]1-, 320.8594[M+(NaCl)4-H]1-, 378.81825[M+(NaCl)5-H]1-, 135.02995[M-H+FA]1-, 262.90035[M+(NaCl)3-H]1- |
| C00209 | Glyoxylate and dicarboxylate metabolism | 206.91985[M+(NaCl)2+H]1+, 148.96118[M+NaCl+H]1+, 146.94667[M+NaCl-H]1-, 88.98811[M-H]1- |
| C00256 | Pyruvate metabolism | 128.99486[M+K]1+, 91.03893[M+H]1+, 113.02087[M+Na]1+, 125.00114[M+Cl]1-, 146.98312[M+NaCl-H]1-, 89.0245[M-H]1-, 204.94161[M+(NaCl)2-H]1-, 320.8594[M+(NaCl)4-H]1-, 378.81825[M+(NaCl)5-H]1-, 135.02995[M-H+FA]1-, 262.90035[M+(NaCl)3-H]1- |
| C00258 | Glyoxylate and dicarboxylate metabolism | 129.01583[M+Na]1+, 164.99248[M+NaCl+H]1+, 280.90964[M+(NaCl)3+H]1+, 222.95103[M+(NaCl)2+H]1+, 105.01929[M-H]1-, 162.97802[M+NaCl-H]1-, 140.99606[M+Cl]1- |
| C00266 | Glyoxylate and dicarboxylate metabolism | 98.98417[M+K]1+, 83.01034[M+Na]1+, 105.01929[M-H+FA]1-, 94.99038[M+Cl]1-, 116.97251[M+NaCl-H]1- |
| C00311 | Glyoxylate and dicarboxylate metabolism | 230.99018[M+K]1+, 215.01617[M+Na]1+, 191.01981[M-H]1-, 248.97839[M+NaCl-H]1-, 364.89598[M+(NaCl)3-H]1-, 306.93734[M+(NaCl)2-H]1- |
| C00322 | Lysine degradation | 183.02646[M+Na]1+, 276.96158[M+(NaCl)2+H]1+, 160.03687[M1+.]1+, 159.02987[M-H]1-, 205.03544[M-H+FA]1- |
| C00408 | Lysine degradation | 152.06821[M+Na]1+, 147.11281[M+NH4]1+, 168.04216[M+K]1+, 130.08631[M+H]1+ |
| C00417 | Glyoxylate and dicarboxylate metabolism | 212.97947[M+K]1+, 173.0091[M-H]1- |
| C00424 | Pyruvate metabolism | 133.00251[M+NaCl+H]1+, 75.04406[M+H]1+, 97.02601[M+Na]1+, 119.03496[M-H+FA]1-, 73.02949[M-H]1- |
| C00431 | Lysine degradation | 156.04211[M+K]1+, 140.0682[M+Na]1+, 118.0862[M+H]1+, 231.989[M+(NaCl)2-H]1-, 162.07723[M-H+FA]1-, 152.04834[M+Cl]1-, 174.0303[M+NaCl-H]1-, 116.07172[M-H]1- |
| C00449 | Lysine degradation | 299.12136[M+Na]1+ |
| C00450 | Lysine degradation | 128.07058[M+H]1+, 145.09715[M+NH4]1+, 150.05259[M+Na]1+ |
| C00487 | Lysine degradation | 162.11244[M1+.]1+ |
| C00489 | Lysine degradation | 191.00811[M+NaCl+H]1+, 248.96668[M+(NaCl)2+H]1+, 171.00542[M+K]1+, 150.07609[M+NH4]1+, 155.03145[M+Na]1+, 131.03498[M-H]1-, 177.04047[M-H+FA]1- |
| C00511 | Styrene degradation | 73.02841[M+H]1+, 90.05497[M+NH4]1+, 117.01937[M-H+FA]1-, 71.01379[M-H]1- |
| C00544 | Styrene degradation | 167.03489[M-H]1- |
| C00546 | Pyruvate metabolism | 73.02841[M+H]1+, 90.05497[M+NH4]1+, 117.01937[M-H+FA]1-, 71.01379[M-H]1- |
| C00596 | Phenylalanine metabolism, Styrene degradation | 132.06556[M+NH4]1+, 115.03889[M+H]1+, 137.02098[M+Na]1+, 159.02987[M-H+FA]1-, 113.02445[M-H]1- |
| C00601 | Phenylalanine metabolism, Styrene degradation | 138.09137[M+NH4]1+, 143.04681[M+Na]1+, 121.06477[M+H]1+, 165.05567[M-H+FA]1- |
| C00642 | Phenylalanine metabolism | 153.05463[M+H]1+, 175.03661[M+Na]1+, 151.04002[M-H]1- |
| C00739 | Lysine degradation | 169.09475[M+Na]1+, 147.11281[M+H]1+, 185.06872[M+K]1+, 181.07497[M+Cl]1-, 145.0983[M-H]1- |
| C00937 | Pyruvate metabolism | 133.00251[M+NaCl+H]1+, 75.04406[M+H]1+, 97.02601[M+Na]1+, 119.03496[M-H+FA]1-, 73.02949[M-H]1- |
| C00956 | Lysine degradation | 184.05806[M+Na]1+, 200.03206[M+K]1+, 162.07609[M+H]1+, 160.06156[M-H]1- |
| C00975 | Glyoxylate and dicarboxylate metabolism | 206.96664[M+NaCl+H]1+ |
| C00988 | Glyoxylate and dicarboxylate metabolism | 178.97173[M+Na]1+, 156.98982[M+H]1+ |
| C01087 | Lysine degradation | 171.02645[M+Na]1+, 148.03692[M1+.]1+, 193.03537[M-H+FA]1-, 147.02988[M-H]1- |
| C01127 | Glyoxylate and dicarboxylate metabolism | 207.01466[M-H+FA]1-, 161.00918[M-H]1- |
| C01142 | Lysine degradation | 169.09475[M+Na]1+, 147.11281[M+H]1+, 185.06872[M+K]1+, 181.07497[M+Cl]1-, 145.0983[M-H]1- |
| C01146 | Glyoxylate and dicarboxylate metabolism | 127.00045[M+Na]1+, 162.97674[M+NaCl+H]1+, 103.00369[M-H]1- |
| C01181 | Lysine degradation | 146.11758[M1+.]1+ |
| C01186 | Lysine degradation | 169.09475[M+Na]1+, 147.11281[M+H]1+, 185.06872[M+K]1+, 181.07497[M+Cl]1-, 145.0983[M-H]1- |
| C01198 | Phenylalanine metabolism | 456.86413[M+(NaCl)5+H]1+, 167.07032[M+H]1+, 189.05231[M+Na]1+, 165.05567[M-H]1- |
| C01251 | Pyruvate metabolism | 265.00907[M+NaCl+H]1+, 229.0319[M+Na]1+, 224.07639[M+NH4]1+, 241.01235[M+Cl]1-, 205.03544[M-H]1- |
| C01659 | Styrene degradation | 72.04445[M+H]1+, 89.07091[M+NH4]1+, 94.02632[M+Na]1+, 116.03533[M-H+FA]1- |
| C01772 | Phenylalanine metabolism | 182.08121[M+NH4]1+, 165.05462[M+H]1+, 203.01014[M+K]1+, 187.03667[M+Na]1+, 163.04012[M-H]1- |
| C01989 | Glyoxylate and dicarboxylate metabolism | 185.04207[M+Na]1+, 162.05248[M1+.]1+, 201.01601[M+K]1+, 180.08656[M+NH4]1+, 163.06011[M+H]1+, 197.0223[M+Cl]1-, 207.0511[M-H+FA]1-, 161.04555[M-H]1- |
| C01990 | Glyoxylate and dicarboxylate metabolism | 380.89004[M+(NaCl)3+H]1+, 322.93145[M+(NaCl)2+H]1+ |
| C02123 | Glyoxylate and dicarboxylate metabolism | 215.03157[M+K]1+, 176.06819[M1+.]1+, 199.05778[M+Na]1+, 221.0666[M-H+FA]1-, 175.06119[M-H]1- |
| C02265 | Phenylalanine metabolism | 204.04223[M+K]1+, 188.06823[M+Na]1+, 166.08624[M+H]1+, 164.0716[M-H]1-, 200.04843[M+Cl]1-, 279.98909[M+(NaCl)2-H]1-, 222.03025[M+NaCl-H]1- |
| C02488 | Pyruvate metabolism | 185.04207[M+Na]1+, 162.05248[M1+.]1+, 201.01601[M+K]1+, 180.08656[M+NH4]1+, 163.06011[M+H]1+, 197.0223[M+Cl]1-, 207.0511[M-H+FA]1-, 161.04555[M-H]1- |
| C02504 | Pyruvate metabolism | 215.03157[M+K]1+, 176.06819[M1+.]1+, 199.05778[M+Na]1+, 221.0666[M-H+FA]1-, 175.06119[M-H]1- |
| C02505 | Phenylalanine metabolism, Styrene degradation | 136.07563[M+H]1+, 158.05766[M+Na]1+, 174.03156[M+K]1+, 180.06664[M-H+FA]1- |
| C02763 | Phenylalanine metabolism | 182.08121[M+NH4]1+, 165.05462[M+H]1+, 203.01014[M+K]1+, 187.03667[M+Na]1+, 163.04012[M-H]1- |
| C03196 | Lysine degradation | 171.02645[M+Na]1+, 148.03692[M1+.]1+, 193.03537[M-H+FA]1-, 147.02988[M-H]1- |
| C03217 | Glyoxylate and dicarboxylate metabolism | 176.03177[M1+.]1+, 194.06598[M+NH4]1+, 199.02149[M+Na]1+, 175.0248[M-H]1- |
| C03239 | Lysine degradation | 146.08117[M+H]1+, 168.06313[M+Na]1+, 184.03708[M+K]1+, 144.06662[M-H]1- |
| C03273 | Lysine degradation | 134.08116[M+NH4]1+, 175.01313[M+NaCl+H]1+, 155.01048[M+K]1+, 117.05462[M+H]1+, 139.03657[M+Na]1+, 151.01669[M+Cl]1-, 115.0401[M-H]1-, 172.99862[M+NaCl-H]1-, 161.04555[M-H+FA]1- |
| C03459 | Glyoxylate and dicarboxylate metabolism | 206.96664[M+NaCl+H]1+ |
| C03548 | Glyoxylate and dicarboxylate metabolism | 177.00406[M-H+FA]1- |
| C03589 | Phenylalanine metabolism | 191.00811[M+NaCl+H]1+, 248.96668[M+(NaCl)2+H]1+, 171.00542[M+K]1+, 150.07609[M+NH4]1+, 155.03145[M+Na]1+, 131.03498[M-H]1-, 177.04047[M-H+FA]1- |
| C03618 | Glyoxylate and dicarboxylate metabolism | 170.04238[M+Na]1+, 186.01634[M+K]1+, 148.06044[M+H]1+, 204.00448[M+NaCl-H]1-, 319.9221[M+(NaCl)3-H]1-, 182.02254[M+Cl]1-, 261.96322[M+(NaCl)2-H]1-, 146.0459[M-H]1- |
| C03656 | Lysine degradation | 146.08117[M+H]1+, 168.06313[M+Na]1+, 184.03708[M+K]1+, 144.06662[M-H]1- |
| C03793 | Lysine degradation | 189.15974[M+H]1+, 211.14166[M+Na]1+ |
| C03981 | Pyruvate metabolism | 177.00406[M-H+FA]1- |
| C04044 | Phenylalanine metabolism | 205.04725[M+Na]1+, 181.05069[M-H]1- |
| C04076 | Lysine degradation | 146.08117[M+H]1+, 168.06313[M+Na]1+, 184.03708[M+K]1+, 144.06662[M-H]1- |
| C04092 | Lysine degradation | 128.07058[M+H]1+, 145.09715[M+NH4]1+, 150.05259[M+Na]1+ |
| C05161 | Lysine degradation | 169.09475[M+Na]1+, 147.11281[M+H]1+, 185.06872[M+K]1+, 181.07497[M+Cl]1-, 145.0983[M-H]1- |
| C05332 | Phenylalanine metabolism | 122.09641[M+H]1+ |
| C05593 | Phenylalanine metabolism, Styrene degradation | 153.05463[M+H]1+, 175.03661[M+Na]1+, 151.04002[M-H]1- |
| C05598 | Phenylalanine metabolism | 216.06323[M+Na]1+ |
| C05607 | Phenylalanine metabolism | 456.86413[M+(NaCl)5+H]1+, 167.07032[M+H]1+, 189.05231[M+Na]1+, 165.05567[M-H]1- |
| C05825 | Lysine degradation | 146.08117[M+H]1+, 168.06313[M+Na]1+, 184.03708[M+K]1+, 144.06662[M-H]1- |
| C05852 | Phenylalanine metabolism, Styrene degradation | 153.05463[M+H]1+, 175.03661[M+Na]1+, 151.04002[M-H]1- |
| C05853 | Phenylalanine metabolism | 123.08042[M+H]1+, 145.06241[M+Na]1+ |
| C05994 | Pyruvate metabolism | 215.03157[M+K]1+, 176.06819[M1+.]1+, 199.05778[M+Na]1+, 221.0666[M-H+FA]1-, 175.06119[M-H]1- |
| C06207 | Phenylalanine metabolism | 167.03489[M-H]1- |
| C07083 | Styrene degradation | 122.09641[M+NH4]1+, 105.06989[M+H]1+ |
| C07085 | Styrene degradation | 159.04174[M+Na]1+, 181.05069[M-H+FA]1-, 135.04523[M-H]1- |
| C07086 | Phenylalanine metabolism, Styrene degradation | 159.04174[M+Na]1+, 181.05069[M-H+FA]1-, 135.04523[M-H]1- |
| C07087 | Styrene degradation | 167.03489[M-H]1- |
| C11457 | Phenylalanine metabolism | 456.86413[M+(NaCl)5+H]1+, 167.07032[M+H]1+, 189.05231[M+Na]1+, 165.05567[M-H]1- |
| C12455 | Lysine degradation | 140.04721[M+K]1+, 102.09134[M+H]1+, 124.07332[M+Na]1+, 146.08222[M-H+FA]1- |
| C12621 | Phenylalanine metabolism | 182.08121[M+NH4]1+, 165.05462[M+H]1+, 203.01014[M+K]1+, 187.03667[M+Na]1+, 163.04012[M-H]1- |
| C12622 | Phenylalanine metabolism | 205.04725[M+Na]1+, 181.05069[M-H]1- |
| C12623 | Phenylalanine metabolism | 180.04207[M1+.]1+, 203.0318[M+Na]1+, 179.03499[M-H]1- |
| C16074 | Styrene degradation | 140.04721[M+Na]1+ |
| C16075 | Styrene degradation | 136.07563[M+H]1+, 158.05766[M+Na]1+, 174.03156[M+K]1+, 180.06664[M-H+FA]1- |
| C20782 | Styrene degradation | 138.09137[M+NH4]1+, 143.04681[M+Na]1+, 121.06477[M+H]1+, 165.05567[M-H+FA]1- |

###
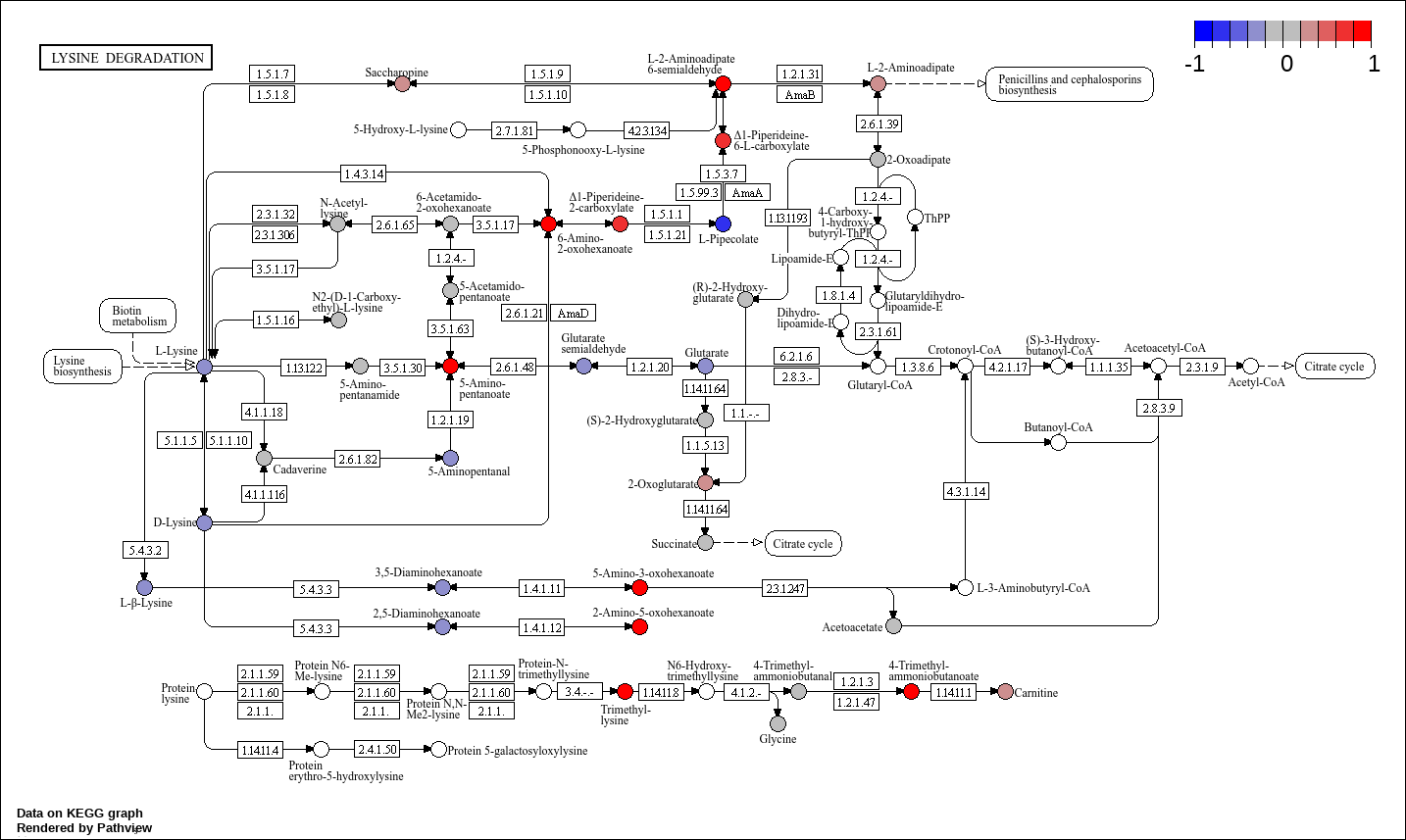


**Figure S8:** V-score projection onto KEGG *microbial metabolism* pathway. Red indicates increased relative V-score in PSC-IBD patients relative to IBD-only. Blue indicates a decrease relative to IBD-only. Grey nodes were found in the dataset but not in the PSC m/z signature.


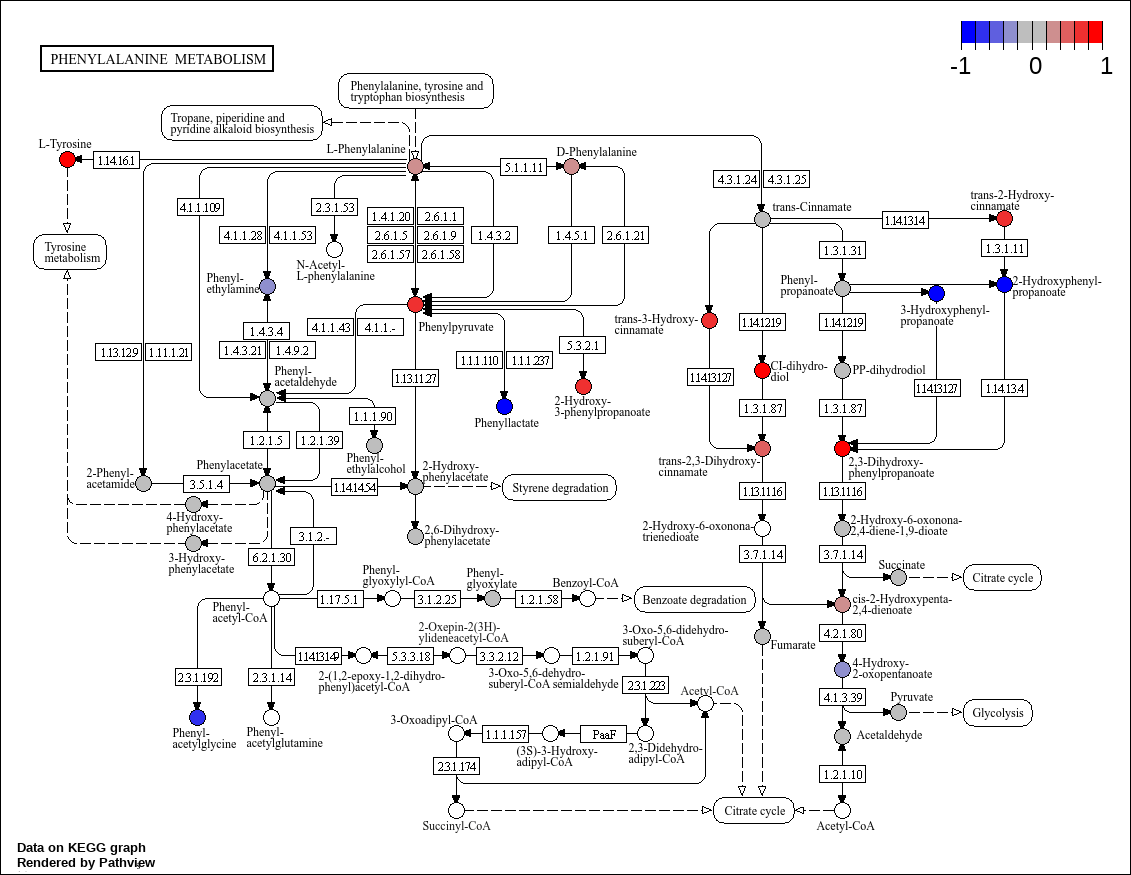


**Figure S9:** V-score projection onto KEGG *microbial metabolism* pathway. Red indicates increased relative V-score in PSC-IBD patients relative to IBD-only. Blue indicates a decrease relative to IBD-only. Grey nodes were found in the dataset but not in the PSC m/z signature.

###
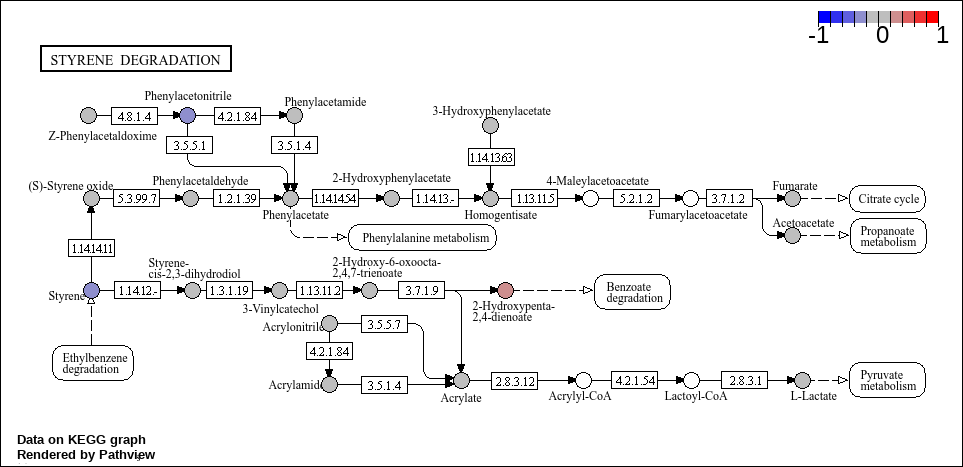


**Figure S10**: V-score projection onto KEGG *microbial metabolism* pathway. Red indicates increased relative V-score in PSC-IBD patients relative to IBD-only. Blue indicates a decrease relative to IBD-only. Grey nodes were found in the dataset but not in the PSC m/z signature.

### Table S8 – GUTSY Atlas V > 0 mummichog enrichment results

|  | Pathway total | Hits.total | Hits.sig | Expected | FET | EASE | Gamma | Emp.Hits |
| --- | --- | --- | --- | --- | --- | --- | --- | --- |
| Dialister pneumosintes MGS:1496 | 76 | 55 | 37 | 24.4770 | 0.00041961 | 0.0010196 | 0.00088278 | 0 |
| Eubacteriales sp. MGS:1167 | 74 | 59 | 39 | 23.8330 | 0.00049889 | 0.0011554 | 0.00088323 | 0 |
| Streptococcus oralis subsp. oralis MGS:0705 | 178 | 125 | 72 | 57.3270 | 0.00082396 | 0.0013536 | 0.00088388 | 0 |
| Clostridium sp. TM06-18 MGS:0048 | 153 | 112 | 65 | 49.2760 | 0.00131860 | 0.0022077 | 0.00088671 | 0 |
| Anaerostipes caccae MGS:0747 | 133 | 93 | 55 | 42.8350 | 0.00200990 | 0.0035228 | 0.00089109 | 0 |
| Eggerthella lenta MGS:0225 | 121 | 81 | 49 | 38.9700 | 0.00191780 | 0.0035485 | 0.00089118 | 0 |
| Bacteroides ovatus MGS:0031 | 68 | 54 | 35 | 21.9000 | 0.00183010 | 0.0040239 | 0.00089276 | 0 |
| Eubacteriales sp. MGS:0085 | 87 | 68 | 42 | 28.0200 | 0.00250520 | 0.0048802 | 0.00089563 | 0 |
| Bacteria sp. MGS:0575 | 35 | 20 | 16 | 11.2720 | 0.00142800 | 0.0058605 | 0.00089893 | 0 |
| Eubacteriales sp. MGS:0084 | 91 | 67 | 41 | 29.3080 | 0.00372440 | 0.0070997 | 0.00090311 | 0 |
| Lachnospiraceae sp. MGS:0389 | 88 | 69 | 42 | 28.3420 | 0.00381180 | 0.0071739 | 0.00090336 | 0 |
| Lachnospiraceae sp. MGS:1271 | 74 | 48 | 31 | 23.8330 | 0.00386100 | 0.0084138 | 0.00090757 | 0 |
| Butyricicoccus pullicaecorum MGS:1374 | 83 | 56 | 35 | 26.7310 | 0.00472540 | 0.0094905 | 0.00091124 | 0 |
| Desulfovibrio piger MGS:0955 | 95 | 62 | 38 | 30.5960 | 0.00521700 | 0.0099932 | 0.00091296 | 0 |
| Actinomyces sp. ICM58 MGS:0410 | 102 | 80 | 47 | 32.8510 | 0.00593300 | 0.0102810 | 0.00091394 | 0 |
| Bacteroides sp. MGS:0030 | 149 | 116 | 64 | 47.9880 | 0.00939320 | 0.0141280 | 0.00092722 | 0 |
| Fusobacterium nucleatum subsp. animalis MGS:1418 | 145 | 106 | 59 | 46.6990 | 0.01031200 | 0.0158310 | 0.00093317 | 0 |
| Pseudoflavonifractor sp. BIOML-A3 MGS:0735 | 39 | 25 | 18 | 12.5600 | 0.00549230 | 0.0159420 | 0.00093355 | 0 |
| Eubacteriales sp. MGS:0260 | 55 | 40 | 26 | 17.7140 | 0.00749890 | 0.0166080 | 0.00093589 | 0 |
| Intestinibacillus sp. Marseille-P4005 MGS:0168 | 189 | 123 | 67 | 60.8700 | 0.01162200 | 0.0170110 | 0.00093730 | 0 |
| Slackia isoflavoniconvertens MGS:0160 | 63 | 42 | 27 | 20.2900 | 0.00800530 | 0.0172480 | 0.00093814 | 0 |
| Desulfovibrionales sp. MGS:0462 | 59 | 42 | 27 | 19.0020 | 0.00800530 | 0.0172480 | 0.00093814 | 0 |
| Dorea formicigenerans MGS:0006 | 175 | 119 | 65 | 56.3610 | 0.01217200 | 0.0179320 | 0.00094055 | 0 |
| Streptococcus gordonii MGS:0713 | 126 | 80 | 46 | 40.5800 | 0.01176300 | 0.0194670 | 0.00094599 | 0 |
| Lachnospiraceae sp. MGS:1209 | 67 | 56 | 34 | 21.5780 | 0.01059300 | 0.0198890 | 0.00094749 | 0 |
| Enterocloster clostridioformis MGS:0686 | 118 | 74 | 43 | 38.0040 | 0.01177200 | 0.0199610 | 0.00094774 | 0 |
| Bifidobacterium catenulatum subsp. kashiwanohense MGS:0292 | 93 | 66 | 39 | 29.9520 | 0.01150000 | 0.0202700 | 0.00094885 | 0 |
| Enterocloster aldenensis MGS:0362 | 151 | 101 | 56 | 48.6320 | 0.01445900 | 0.0220600 | 0.00095525 | 0 |
| Ruminococcus callidus MGS:0247 | 48 | 37 | 24 | 15.4590 | 0.01075300 | 0.0236860 | 0.00096110 | 2 |
| Scardovia wiggsiae MGS:1737 | 170 | 116 | 63 | 54.7510 | 0.01665600 | 0.0242870 | 0.00096327 | 0 |
| Megamonas funiformis MGS:0684 | 64 | 47 | 29 | 20.6120 | 0.01376100 | 0.0268750 | 0.00097269 | 0 |
| Oscillospiraceae sp. MGS:0074 | 64 | 47 | 29 | 20.6120 | 0.01376100 | 0.0268750 | 0.00097269 | 0 |
| Streptococcus mutans MGS:0677 | 202 | 125 | 67 | 65.0570 | 0.01961400 | 0.0278110 | 0.00097612 | 0 |
| Eubacteriales sp. MGS:0103 | 80 | 55 | 33 | 25.7650 | 0.01526200 | 0.0279210 | 0.00097652 | 0 |
| Eubacteriales sp. MGS:0128 | 156 | 104 | 57 | 50.2420 | 0.01877400 | 0.0279710 | 0.00097671 | 0 |
| Clostridium sp. MGS:0050 | 157 | 104 | 57 | 50.5640 | 0.01877400 | 0.0279710 | 0.00097671 | 0 |
| Desulfovibrio piger MGS:0240 | 92 | 57 | 34 | 29.6300 | 0.01552700 | 0.0280230 | 0.00097690 | 0 |
| Anaerobutyricum hallii MGS:0012 | 154 | 100 | 55 | 49.5980 | 0.01943200 | 0.0291900 | 0.00098119 | 0 |
| Ruminococcus torques MGS:0034 | 172 | 117 | 63 | 55.3950 | 0.02157200 | 0.0309300 | 0.00098764 | 0 |
| Sutterella seckii MGS:0997 | 15 | 12 | 10 | 4.8310 | 0.00799780 | 0.0353090 | 0.00100410 | 0 |
| Gemmiger sp. MGS:0862 | 58 | 38 | 24 | 18.6800 | 0.01725500 | 0.0356390 | 0.00100530 | 0 |
| Parabacteroides johnsonii MGS:0469 | 65 | 40 | 25 | 20.9340 | 0.01806800 | 0.0364060 | 0.00100820 | 0 |
| Longibaculum muris MGS:1605 | 50 | 42 | 26 | 16.1030 | 0.01879900 | 0.0370390 | 0.00101060 | 0 |
| Eubacteriales sp. MGS:0671 | 91 | 66 | 38 | 29.3080 | 0.02260000 | 0.0377080 | 0.00101320 | 0 |
| Clostridia sp. MGS:0845 | 76 | 60 | 35 | 24.4770 | 0.02234900 | 0.0384140 | 0.00101590 | 0 |
| Clostridia sp. MGS:1035 | 64 | 50 | 30 | 20.6120 | 0.02098700 | 0.0384680 | 0.00101610 | 0 |
| Clostridium sp. AT4 MGS:0347 | 170 | 112 | 60 | 54.7510 | 0.02954600 | 0.0420260 | 0.00102980 | 0 |
| Oscillospiraceae sp. MGS:0256 | 169 | 110 | 59 | 54.4290 | 0.03018700 | 0.0430640 | 0.00103380 | 0 |
| Gemella morbillorum MGS:1782 | 117 | 85 | 47 | 37.6810 | 0.02866700 | 0.0437580 | 0.00103650 | 0 |
| Saccharomyces cerevisiae MGS:0782 | 51 | 29 | 19 | 16.4250 | 0.02044800 | 0.0460950 | 0.00104570 | 1 |
| Eubacteriales sp. MGS:0335 | 108 | 75 | 42 | 34.7830 | 0.03016600 | 0.0474070 | 0.00105090 | 0 |
| Porphyromonas sp. MGS:1016 | 52 | 31 | 20 | 16.7470 | 0.02193700 | 0.0476610 | 0.00105190 | 0 |
| Eubacteriales sp. MGS:1313 | 50 | 31 | 20 | 16.1030 | 0.02193700 | 0.0476610 | 0.00105190 | 0 |
| Megasphaera sp. DJF_B143 MGS:1121 | 27 | 16 | 12 | 8.6957 | 0.01509800 | 0.0480060 | 0.00105330 | 1 |
| Dorea sp. AF36-15AT MGS:0052 | 177 | 119 | 63 | 57.0050 | 0.03497700 | 0.0485530 | 0.00105550 | 0 |
| Sutterella sp. KLE1602 MGS:0228 | 101 | 71 | 40 | 32.5280 | 0.03058100 | 0.0487230 | 0.00105610 | 0 |
| Lachnospiraceae sp. MGS:0625 | 49 | 33 | 21 | 15.7810 | 0.02328200 | 0.0489690 | 0.00105710 | 0 |
| Bacteria sp. MGS:0459 | 96 | 69 | 39 | 30.9180 | 0.03073900 | 0.0493390 | 0.00105860 | 0 |
| Clostridium sp. TF06-15AC MGS:0032 | 143 | 98 | 53 | 46.0550 | 0.03395200 | 0.0494140 | 0.00105890 | 0 |

### Table S9 – GUTSY Atlas V > 0 mummichog enrichment matches

| Compound identifier | Pathway name | Matches [adduct] |
| --- | --- | --- |
| 1,5-anhydroglucitol (1,5-AG) | Bacteria sp. MGS:0575, Eubacteriales sp. MGS:0084, Lachnospiraceae sp. MGS:0389, Eubacteriales sp. MGS:0260, Streptococcus gordonii MGS:0713, Bifidobacterium catenulatum subsp. kashiwanohense MGS:0292, Ruminococcus callidus MGS:0247, Scardovia wiggsiae MGS:1737, Oscillospiraceae sp. MGS:0074, Streptococcus mutans MGS:0677, Eubacteriales sp. MGS:0103, Clostridium sp. MGS:0050, Ruminococcus torques MGS:0034, Saccharomyces cerevisiae MGS:0782, Porphyromonas sp. MGS:1016 | 164.06818[M1+.]1+, 187.05771[M+Na]1+, 203.0318[M+K]1+, 182.10234[M+NH4]1+, 199.03794[M+Cl]1-, 163.06124[M-H]1-, 209.06671[M-H+FA]1- |
| 1-arachidonoyl-GPE (20:4n6)* | Dialister pneumosintes MGS:1496, Streptococcus oralis subsp. oralis MGS:0705, Clostridium sp. TM06-18 MGS:0048, Anaerostipes caccae MGS:0747, Lachnospiraceae sp. MGS:1271, Desulfovibrio piger MGS:0955, Actinomyces sp. ICM58 MGS:0410, Fusobacterium nucleatum subsp. animalis MGS:1418, Intestinibacillus sp. Marseille-P4005 MGS:0168, Desulfovibrionales sp. MGS:0462, Dorea formicigenerans MGS:0006, Streptococcus gordonii MGS:0713, Enterocloster clostridioformis MGS:0686, Enterocloster aldenensis MGS:0362, Scardovia wiggsiae MGS:1737, Megamonas funiformis MGS:0684, Eubacteriales sp. MGS:0128, Clostridium sp. MGS:0050, Desulfovibrio piger MGS:0240, Anaerobutyricum hallii MGS:0012, Parabacteroides johnsonii MGS:0469, Clostridium sp. AT4 MGS:0347, Oscillospiraceae sp. MGS:0256, Gemella morbillorum MGS:1782, Eubacteriales sp. MGS:0335, Dorea sp. AF36-15AT MGS:0052, Sutterella sp. KLE1602 MGS:0228, Bacteria sp. MGS:0459, Clostridium sp. TF06-15AC MGS:0032 | 524.2743[M+Na]1+, 500.2782[M-H]1- |
| 1-linolenoylglycerol (18:3) | Clostridium sp. TM06-18 MGS:0048, Anaerostipes caccae MGS:0747, Eggerthella lenta MGS:0225, Desulfovibrio piger MGS:0955, Fusobacterium nucleatum subsp. animalis MGS:1418, Intestinibacillus sp. Marseille-P4005 MGS:0168, Dorea formicigenerans MGS:0006, Enterocloster clostridioformis MGS:0686, Scardovia wiggsiae MGS:1737, Anaerobutyricum hallii MGS:0012, Sutterella seckii MGS:0997, Clostridium sp. AT4 MGS:0347, Oscillospiraceae sp. MGS:0256, Saccharomyces cerevisiae MGS:0782, Eubacteriales sp. MGS:0335, Dorea sp. AF36-15AT MGS:0052, Clostridium sp. TF06-15AC MGS:0032 | 370.29509[M+NH4]1+ |
| 1-linoleoylglycerol (18:2) | Clostridium sp. TM06-18 MGS:0048, Scardovia wiggsiae MGS:1737, Clostridium sp. AT4 MGS:0347, Clostridium sp. TF06-15AC MGS:0032 | 372.31078[M+NH4]1+, 377.2661[M+Na]1+ |
| 1-oleoylglycerol (18:1) | Streptococcus oralis subsp. oralis MGS:0705, Clostridium sp. TM06-18 MGS:0048, Anaerostipes caccae MGS:0747, Eggerthella lenta MGS:0225, Fusobacterium nucleatum subsp. animalis MGS:1418, Intestinibacillus sp. Marseille-P4005 MGS:0168, Desulfovibrionales sp. MGS:0462, Dorea formicigenerans MGS:0006, Enterocloster clostridioformis MGS:0686, Enterocloster aldenensis MGS:0362, Scardovia wiggsiae MGS:1737, Megamonas funiformis MGS:0684, Streptococcus mutans MGS:0677, Eubacteriales sp. MGS:0128, Clostridium sp. MGS:0050, Anaerobutyricum hallii MGS:0012, Ruminococcus torques MGS:0034, Clostridium sp. AT4 MGS:0347, Oscillospiraceae sp. MGS:0256, Saccharomyces cerevisiae MGS:0782, Eubacteriales sp. MGS:0335, Dorea sp. AF36-15AT MGS:0052, Lachnospiraceae sp. MGS:0625, Clostridium sp. TF06-15AC MGS:0032 | 379.28178[M+Na]1+, 395.25572[M+K]1+, 391.26284[M+Cl]1- |
| 1-palmitoleoyl-GPC (16:1)* | Streptococcus oralis subsp. oralis MGS:0705, Clostridium sp. TM06-18 MGS:0048, Anaerostipes caccae MGS:0747, Eggerthella lenta MGS:0225, Butyricicoccus pullicaecorum MGS:1374, Actinomyces sp. ICM58 MGS:0410, Intestinibacillus sp. Marseille-P4005 MGS:0168, Slackia isoflavoniconvertens MGS:0160, Desulfovibrionales sp. MGS:0462, Dorea formicigenerans MGS:0006, Streptococcus gordonii MGS:0713, Bifidobacterium catenulatum subsp. kashiwanohense MGS:0292, Enterocloster aldenensis MGS:0362, Scardovia wiggsiae MGS:1737, Megamonas funiformis MGS:0684, Streptococcus mutans MGS:0677, Eubacteriales sp. MGS:0128, Clostridium sp. MGS:0050, Desulfovibrio piger MGS:0240, Anaerobutyricum hallii MGS:0012, Ruminococcus torques MGS:0034, Clostridium sp. AT4 MGS:0347, Oscillospiraceae sp. MGS:0256, Saccharomyces cerevisiae MGS:0782, Eubacteriales sp. MGS:0335, Dorea sp. AF36-15AT MGS:0052, Clostridium sp. TF06-15AC MGS:0032 | 516.3059[M+Na]1+, 532.2798[M+K]1+, 494.32409[M+H]1+ |
| 1-ribosyl-imidazoleacetate* | Eubacteriales sp. MGS:1167, Streptococcus oralis subsp. oralis MGS:0705, Bacteroides ovatus MGS:0031, Bacteria sp. MGS:0575, Eubacteriales sp. MGS:0084, Bacteroides sp. MGS:0030, Streptococcus gordonii MGS:0713, Lachnospiraceae sp. MGS:1209, Bifidobacterium catenulatum subsp. kashiwanohense MGS:0292, Scardovia wiggsiae MGS:1737, Streptococcus mutans MGS:0677, Eubacteriales sp. MGS:0671, Clostridia sp. MGS:0845, Clostridia sp. MGS:1035 | 276.11906[M+NH4]1+, 490.92608[M+(NaCl)4+H]1+ |
| 10-heptadecenoate (17:1n7) | Dialister pneumosintes MGS:1496, Streptococcus oralis subsp. oralis MGS:0705, Clostridium sp. TM06-18 MGS:0048, Anaerostipes caccae MGS:0747, Eggerthella lenta MGS:0225, Butyricicoccus pullicaecorum MGS:1374, Desulfovibrio piger MGS:0955, Actinomyces sp. ICM58 MGS:0410, Fusobacterium nucleatum subsp. animalis MGS:1418, Intestinibacillus sp. Marseille-P4005 MGS:0168, Desulfovibrionales sp. MGS:0462, Dorea formicigenerans MGS:0006, Scardovia wiggsiae MGS:1737, Megamonas funiformis MGS:0684, Eubacteriales sp. MGS:0128, Clostridium sp. MGS:0050, Desulfovibrio piger MGS:0240, Anaerobutyricum hallii MGS:0012, Ruminococcus torques MGS:0034, Clostridium sp. AT4 MGS:0347, Oscillospiraceae sp. MGS:0256, Gemella morbillorum MGS:1782, Eubacteriales sp. MGS:0335, Dorea sp. AF36-15AT MGS:0052, Sutterella sp. KLE1602 MGS:0228, Bacteria sp. MGS:0459, Clostridium sp. TF06-15AC MGS:0032 | 291.22951[M+Na]1+, 307.20332[M+K]1+, 303.20986[M+Cl]1- |
| 10-undecenoate (11:1n1) | Eubacteriales sp. MGS:0085, Eubacteriales sp. MGS:0084, Oscillospiraceae sp. MGS:0074, Sutterella seckii MGS:0997, Dorea sp. AF36-15AT MGS:0052 | 185.15363[M+H]1+, 207.13564[M+Na]1+, 202.18018[M+NH4]1+, 223.10945[M+K]1+, 229.14459[M-H+FA]1-, 183.13902[M-H]1- |
| 16-hydroxypalmitate | Dialister pneumosintes MGS:1496, Streptococcus oralis subsp. oralis MGS:0705, Clostridium sp. TM06-18 MGS:0048, Anaerostipes caccae MGS:0747, Eggerthella lenta MGS:0225, Butyricicoccus pullicaecorum MGS:1374, Bacteroides sp. MGS:0030, Fusobacterium nucleatum subsp. animalis MGS:1418, Intestinibacillus sp. Marseille-P4005 MGS:0168, Dorea formicigenerans MGS:0006, Streptococcus gordonii MGS:0713, Bifidobacterium catenulatum subsp. kashiwanohense MGS:0292, Scardovia wiggsiae MGS:1737, Megamonas funiformis MGS:0684, Clostridium sp. MGS:0050, Ruminococcus torques MGS:0034, Clostridium sp. AT4 MGS:0347, Oscillospiraceae sp. MGS:0256, Eubacteriales sp. MGS:0335, Dorea sp. AF36-15AT MGS:0052, Bacteria sp. MGS:0459 | 311.19816[M+K]1+, 295.22433[M+Na]1+, 271.22806[M-H]1-, 307.20488[M+Cl]1- |
| 2,3-dihydroxy-2-methylbutyrate | Eubacteriales sp. MGS:1167, Bacteroides ovatus MGS:0031, Lachnospiraceae sp. MGS:1209, Bacteria sp. MGS:0459 | 173.02106[M+K]1+, 135.06518[M+H]1+, 157.04718[M+Na]1+, 169.02755[M+Cl]1-, 133.05065[M-H]1-, 179.05615[M-H+FA]1- |
| 2,3-dihydroxyisovalerate | Eubacteriales sp. MGS:1167, Butyricicoccus pullicaecorum MGS:1374, Bacteroides sp. MGS:0030, Lachnospiraceae sp. MGS:1209, Parabacteroides johnsonii MGS:0469, Bacteria sp. MGS:0459 | 173.02106[M+K]1+, 135.06518[M+H]1+, 157.04718[M+Na]1+, 169.02755[M+Cl]1-, 133.05065[M-H]1-, 179.05615[M-H+FA]1- |
| 2-O-methylascorbic acid | Dialister pneumosintes MGS:1496, Streptococcus oralis subsp. oralis MGS:0705, Clostridium sp. TM06-18 MGS:0048, Anaerostipes caccae MGS:0747, Bacteroides ovatus MGS:0031, Lachnospiraceae sp. MGS:1271, Butyricicoccus pullicaecorum MGS:1374, Actinomyces sp. ICM58 MGS:0410, Bacteroides sp. MGS:0030, Fusobacterium nucleatum subsp. animalis MGS:1418, Intestinibacillus sp. Marseille-P4005 MGS:0168, Dorea formicigenerans MGS:0006, Streptococcus gordonii MGS:0713, Bifidobacterium catenulatum subsp. kashiwanohense MGS:0292, Enterocloster aldenensis MGS:0362, Scardovia wiggsiae MGS:1737, Megamonas funiformis MGS:0684, Streptococcus mutans MGS:0677, Eubacteriales sp. MGS:0128, Clostridium sp. MGS:0050, Anaerobutyricum hallii MGS:0012, Ruminococcus torques MGS:0034, Gemmiger sp. MGS:0862, Clostridium sp. AT4 MGS:0347, Oscillospiraceae sp. MGS:0256, Eubacteriales sp. MGS:0335, Dorea sp. AF36-15AT MGS:0052, Sutterella sp. KLE1602 MGS:0228, Clostridium sp. TF06-15AC MGS:0032 | 190.04747[M1+.]1+, 208.08154[M+NH4]1+, 189.0405[M-H]1- |
| 2-aminoadipate | Streptococcus oralis subsp. oralis MGS:0705, Clostridium sp. TM06-18 MGS:0048, Intestinibacillus sp. Marseille-P4005 MGS:0168, Slackia isoflavoniconvertens MGS:0160, Dorea formicigenerans MGS:0006, Ruminococcus callidus MGS:0247, Scardovia wiggsiae MGS:1737, Oscillospiraceae sp. MGS:0074, Streptococcus mutans MGS:0677, Eubacteriales sp. MGS:0128, Clostridium sp. MGS:0050, Anaerobutyricum hallii MGS:0012, Ruminococcus torques MGS:0034, Clostridium sp. AT4 MGS:0347, Gemella morbillorum MGS:1782, Eubacteriales sp. MGS:0335, Megasphaera sp. DJF_B143 MGS:1121, Dorea sp. AF36-15AT MGS:0052, Clostridium sp. TF06-15AC MGS:0032 | 184.05806[M+Na]1+, 200.03206[M+K]1+, 162.07609[M+H]1+, 160.06156[M-H]1- |
| 2-aminoheptanoate | Eubacteriales sp. MGS:1167, Bacteroides ovatus MGS:0031, Eubacteriales sp. MGS:0085, Bacteria sp. MGS:0575, Eubacteriales sp. MGS:0084, Eubacteriales sp. MGS:0260, Bifidobacterium catenulatum subsp. kashiwanohense MGS:0292, Ruminococcus callidus MGS:0247, Scardovia wiggsiae MGS:1737, Oscillospiraceae sp. MGS:0074, Streptococcus mutans MGS:0677, Eubacteriales sp. MGS:0103, Eubacteriales sp. MGS:0671, Clostridia sp. MGS:0845 | 146.11758[M+H]1+, 168.09951[M+Na]1+, 184.07337[M+K]1+ |
| 2-hydroxy-3-methylvalerate | Dialister pneumosintes MGS:1496, Streptococcus oralis subsp. oralis MGS:0705, Clostridium sp. TM06-18 MGS:0048, Anaerostipes caccae MGS:0747, Bacteroides ovatus MGS:0031, Lachnospiraceae sp. MGS:1271, Desulfovibrio piger MGS:0955, Actinomyces sp. ICM58 MGS:0410, Bacteroides sp. MGS:0030, Fusobacterium nucleatum subsp. animalis MGS:1418, Intestinibacillus sp. Marseille-P4005 MGS:0168, Desulfovibrionales sp. MGS:0462, Dorea formicigenerans MGS:0006, Enterocloster clostridioformis MGS:0686, Enterocloster aldenensis MGS:0362, Eubacteriales sp. MGS:0128, Clostridium sp. MGS:0050, Desulfovibrio piger MGS:0240, Ruminococcus torques MGS:0034, Gemmiger sp. MGS:0862, Parabacteroides johnsonii MGS:0469, Longibaculum muris MGS:1605, Clostridium sp. AT4 MGS:0347, Oscillospiraceae sp. MGS:0256, Gemella morbillorum MGS:1782, Eubacteriales sp. MGS:0335, Dorea sp. AF36-15AT MGS:0052, Sutterella sp. KLE1602 MGS:0228, Clostridium sp. TF06-15AC MGS:0032 | 191.04453[M+NaCl+H]1+, 150.11252[M+NH4]1+, 171.04171[M+K]1+, 155.06786[M+Na]1+, 133.08593[M+H]1+, 167.04798[M+Cl]1-, 131.07138[M-H]1-, 177.07683[M-H+FA]1- |
| 2-hydroxydecanoate | Dialister pneumosintes MGS:1496, Eubacteriales sp. MGS:1167, Bacteroides ovatus MGS:0031, Bacteroides sp. MGS:0030, Fusobacterium nucleatum subsp. animalis MGS:1418, Pseudoflavonifractor sp. BIOML-A3 MGS:0735, Enterocloster clostridioformis MGS:0686, Enterocloster aldenensis MGS:0362, Eubacteriales sp. MGS:0671, Clostridia sp. MGS:0845, Clostridia sp. MGS:1035, Bacteria sp. MGS:0459 | 211.13042[M+Na]1+, 227.10425[M+K]1+, 187.13398[M-H]1- |
| 2-hydroxyglutarate | Anaerostipes caccae MGS:0747, Intestinibacillus sp. Marseille-P4005 MGS:0168, Enterocloster clostridioformis MGS:0686, Enterocloster aldenensis MGS:0362, Parabacteroides johnsonii MGS:0469, Bacteria sp. MGS:0459 | 171.02645[M+Na]1+, 148.03692[M1+.]1+, 193.03537[M-H+FA]1-, 147.02988[M-H]1- |
| 2-hydroxypalmitate | Eggerthella lenta MGS:0225, Butyricicoccus pullicaecorum MGS:1374, Bacteroides sp. MGS:0030, Fusobacterium nucleatum subsp. animalis MGS:1418, Enterocloster aldenensis MGS:0362 | 311.19816[M+K]1+, 295.22433[M+Na]1+, 271.22806[M-H]1-, 307.20488[M+Cl]1- |
| 2-hydroxyphenylacetate | Eubacteriales sp. MGS:1167, Clostridium sp. TM06-18 MGS:0048, Anaerostipes caccae MGS:0747, Enterocloster clostridioformis MGS:0686 | 153.05463[M+H]1+, 175.03661[M+Na]1+, 151.04002[M-H]1- |
| 2-hydroxystearate | Eggerthella lenta MGS:0225, Pseudoflavonifractor sp. BIOML-A3 MGS:0735 | 323.25564[M+Na]1+, 339.22939[M+K]1+, 299.25946[M-H]1-, 335.23649[M+Cl]1- |
| 2-linoleoylglycerol (18:2) | Eubacteriales sp. MGS:0103 | 372.31078[M+NH4]1+, 377.2661[M+Na]1+ |
| 2-oleoylglycerol (18:1) | Clostridium sp. TM06-18 MGS:0048, Anaerostipes caccae MGS:0747, Lachnospiraceae sp. MGS:1271, Butyricicoccus pullicaecorum MGS:1374, Desulfovibrio piger MGS:0955, Fusobacterium nucleatum subsp. animalis MGS:1418, Intestinibacillus sp. Marseille-P4005 MGS:0168, Desulfovibrionales sp. MGS:0462, Dorea formicigenerans MGS:0006, Enterocloster clostridioformis MGS:0686, Enterocloster aldenensis MGS:0362, Scardovia wiggsiae MGS:1737, Streptococcus mutans MGS:0677, Eubacteriales sp. MGS:0128, Clostridium sp. MGS:0050, Anaerobutyricum hallii MGS:0012, Ruminococcus torques MGS:0034, Clostridium sp. AT4 MGS:0347, Oscillospiraceae sp. MGS:0256, Eubacteriales sp. MGS:0335, Lachnospiraceae sp. MGS:0625, Clostridium sp. TF06-15AC MGS:0032 | 379.28178[M+Na]1+, 395.25572[M+K]1+, 391.26284[M+Cl]1- |
| 2-piperidinone | Dialister pneumosintes MGS:1496, Streptococcus oralis subsp. oralis MGS:0705, Clostridium sp. TM06-18 MGS:0048, Eubacteriales sp. MGS:0084, Lachnospiraceae sp. MGS:0389, Actinomyces sp. ICM58 MGS:0410, Fusobacterium nucleatum subsp. animalis MGS:1418, Slackia isoflavoniconvertens MGS:0160, Desulfovibrionales sp. MGS:0462, Dorea formicigenerans MGS:0006, Streptococcus gordonii MGS:0713, Bifidobacterium catenulatum subsp. kashiwanohense MGS:0292, Ruminococcus callidus MGS:0247, Oscillospiraceae sp. MGS:0074, Streptococcus mutans MGS:0677, Eubacteriales sp. MGS:0103, Eubacteriales sp. MGS:0128, Clostridium sp. MGS:0050, Desulfovibrio piger MGS:0240, Anaerobutyricum hallii MGS:0012, Ruminococcus torques MGS:0034, Clostridia sp. MGS:1035, Gemella morbillorum MGS:1782, Megasphaera sp. DJF_B143 MGS:1121, Sutterella sp. KLE1602 MGS:0228, Clostridium sp. TF06-15AC MGS:0032 | 138.03162[M+K]1+, 117.10223[M+NH4]1+, 100.07566[M+H]1+, 122.05762[M+Na]1+, 144.06662[M-H+FA]1- |
| 2R,3R-dihydroxybutyrate | Streptococcus oralis subsp. oralis MGS:0705, Clostridium sp. TM06-18 MGS:0048, Anaerostipes caccae MGS:0747, Eggerthella lenta MGS:0225, Lachnospiraceae sp. MGS:1271, Butyricicoccus pullicaecorum MGS:1374, Intestinibacillus sp. Marseille-P4005 MGS:0168, Dorea formicigenerans MGS:0006, Streptococcus gordonii MGS:0713, Enterocloster aldenensis MGS:0362, Streptococcus mutans MGS:0677, Eubacteriales sp. MGS:0128, Clostridium sp. MGS:0050, Anaerobutyricum hallii MGS:0012, Ruminococcus torques MGS:0034, Parabacteroides johnsonii MGS:0469, Longibaculum muris MGS:1605, Clostridium sp. AT4 MGS:0347, Oscillospiraceae sp. MGS:0256, Dorea sp. AF36-15AT MGS:0052, Clostridium sp. TF06-15AC MGS:0032 | 143.03153[M+Na]1+, 159.00544[M+K]1+, 119.03496[M-H]1-, 155.01164[M+Cl]1-, 165.04037[M-H+FA]1-, 176.99351[M+NaCl-H]1- |
| 2S,3R-dihydroxybutyrate | Clostridium sp. TM06-18 MGS:0048, Bacteroides ovatus MGS:0031, Lachnospiraceae sp. MGS:1271, Desulfovibrio piger MGS:0955, Actinomyces sp. ICM58 MGS:0410, Bacteroides sp. MGS:0030, Intestinibacillus sp. Marseille-P4005 MGS:0168, Dorea formicigenerans MGS:0006, Enterocloster aldenensis MGS:0362, Eubacteriales sp. MGS:0128, Clostridium sp. MGS:0050, Ruminococcus torques MGS:0034, Parabacteroides johnsonii MGS:0469, Longibaculum muris MGS:1605, Clostridium sp. AT4 MGS:0347, Oscillospiraceae sp. MGS:0256, Gemella morbillorum MGS:1782, Eubacteriales sp. MGS:0335, Dorea sp. AF36-15AT MGS:0052, Lachnospiraceae sp. MGS:0625, Bacteria sp. MGS:0459 | 143.03153[M+Na]1+, 159.00544[M+K]1+, 119.03496[M-H]1-, 155.01164[M+Cl]1-, 165.04037[M-H+FA]1-, 176.99351[M+NaCl-H]1- |
| 3,4-dihydroxybutyrate | Streptococcus oralis subsp. oralis MGS:0705, Anaerostipes caccae MGS:0747, Eggerthella lenta MGS:0225, Desulfovibrio piger MGS:0955, Bacteroides sp. MGS:0030, Fusobacterium nucleatum subsp. animalis MGS:1418, Intestinibacillus sp. Marseille-P4005 MGS:0168, Slackia isoflavoniconvertens MGS:0160, Dorea formicigenerans MGS:0006, Streptococcus gordonii MGS:0713, Enterocloster clostridioformis MGS:0686, Bifidobacterium catenulatum subsp. kashiwanohense MGS:0292, Enterocloster aldenensis MGS:0362, Scardovia wiggsiae MGS:1737, Streptococcus mutans MGS:0677, Eubacteriales sp. MGS:0128, Desulfovibrio piger MGS:0240, Ruminococcus torques MGS:0034, Parabacteroides johnsonii MGS:0469, Clostridium sp. AT4 MGS:0347, Oscillospiraceae sp. MGS:0256, Gemella morbillorum MGS:1782, Eubacteriales sp. MGS:0335, Dorea sp. AF36-15AT MGS:0052, Lachnospiraceae sp. MGS:0625 | 143.03153[M+Na]1+, 159.00544[M+K]1+, 119.03496[M-H]1-, 155.01164[M+Cl]1-, 165.04037[M-H+FA]1-, 176.99351[M+NaCl-H]1- |
| 3-(3-hydroxyphenyl)propionate | Eggerthella lenta MGS:0225, Bacteroides ovatus MGS:0031, Lachnospiraceae sp. MGS:0389, Streptococcus gordonii MGS:0713, Bifidobacterium catenulatum subsp. kashiwanohense MGS:0292, Oscillospiraceae sp. MGS:0074, Anaerobutyricum hallii MGS:0012, Saccharomyces cerevisiae MGS:0782 | 456.86413[M+(NaCl)5+H]1+, 167.07032[M+H]1+, 189.05231[M+Na]1+, 165.05567[M-H]1- |
| 3-(4-hydroxyphenyl)lactate | Streptococcus oralis subsp. oralis MGS:0705, Clostridium sp. TM06-18 MGS:0048, Bacteroides ovatus MGS:0031, Eubacteriales sp. MGS:0085, Lachnospiraceae sp. MGS:0389, Actinomyces sp. ICM58 MGS:0410, Bacteroides sp. MGS:0030, Lachnospiraceae sp. MGS:1209, Bifidobacterium catenulatum subsp. kashiwanohense MGS:0292, Ruminococcus callidus MGS:0247, Clostridium sp. MGS:0050, Anaerobutyricum hallii MGS:0012, Ruminococcus torques MGS:0034, Longibaculum muris MGS:1605, Gemella morbillorum MGS:1782, Clostridium sp. TF06-15AC MGS:0032 | 205.04725[M+Na]1+, 181.05069[M-H]1- |
| 3-hydroxy-2-ethylpropionate | Dialister pneumosintes MGS:1496, Eggerthella lenta MGS:0225, Desulfovibrio piger MGS:0955, Bacteroides sp. MGS:0030, Fusobacterium nucleatum subsp. animalis MGS:1418, Pseudoflavonifractor sp. BIOML-A3 MGS:0735, Enterocloster aldenensis MGS:0362, Desulfovibrio piger MGS:0240, Longibaculum muris MGS:1605, Clostridium sp. AT4 MGS:0347, Eubacteriales sp. MGS:0335, Dorea sp. AF36-15AT MGS:0052, Lachnospiraceae sp. MGS:0625, Bacteria sp. MGS:0459 | 119.07029[M+H]1+, 141.0522[M+Na]1+, 157.02605[M+K]1+, 117.05576[M-H]1-, 175.01426[M+NaCl-H]1-, 153.03241[M+Cl]1-, 163.06124[M-H+FA]1- |
| 3-hydroxy-3-methylglutarate | Streptococcus oralis subsp. oralis MGS:0705, Anaerostipes caccae MGS:0747, Eggerthella lenta MGS:0225, Bacteroides sp. MGS:0030, Fusobacterium nucleatum subsp. animalis MGS:1418, Intestinibacillus sp. Marseille-P4005 MGS:0168, Streptococcus gordonii MGS:0713, Enterocloster clostridioformis MGS:0686, Enterocloster aldenensis MGS:0362, Scardovia wiggsiae MGS:1737, Streptococcus mutans MGS:0677, Dorea sp. AF36-15AT MGS:0052, Lachnospiraceae sp. MGS:0625, Bacteria sp. MGS:0459 | 185.04207[M+Na]1+, 201.01601[M+K]1+, 162.05248[M1+.]1+, 180.08656[M+NH4]1+, 163.06011[M+H]1+, 197.0223[M+Cl]1-, 207.0511[M-H+FA]1-, 161.04555[M-H]1- |
| 3-hydroxyadipate | Streptococcus oralis subsp. oralis MGS:0705, Anaerostipes caccae MGS:0747, Eggerthella lenta MGS:0225, Desulfovibrio piger MGS:0955, Bacteroides sp. MGS:0030, Fusobacterium nucleatum subsp. animalis MGS:1418, Pseudoflavonifractor sp. BIOML-A3 MGS:0735, Intestinibacillus sp. Marseille-P4005 MGS:0168, Streptococcus gordonii MGS:0713, Enterocloster aldenensis MGS:0362, Scardovia wiggsiae MGS:1737, Ruminococcus torques MGS:0034, Oscillospiraceae sp. MGS:0256 | 185.04207[M+Na]1+, 201.01601[M+K]1+, 162.05248[M1+.]1+, 180.08656[M+NH4]1+, 163.06011[M+H]1+, 197.0223[M+Cl]1-, 207.0511[M-H+FA]1-, 161.04555[M-H]1- |
| 3-hydroxydecanoate | Bacteroides sp. MGS:0030, Bifidobacterium catenulatum subsp. kashiwanohense MGS:0292 | 211.13042[M+Na]1+, 227.10425[M+K]1+, 187.13398[M-H]1- |
| 3-hydroxydecanoylcarnitine | Bacteroides sp. MGS:0030, Bifidobacterium catenulatum subsp. kashiwanohense MGS:0292, Eubacteriales sp. MGS:0671 | 332.24309[M+H]1+ |
| 3-indoxyl sulfate | Dialister pneumosintes MGS:1496, Streptococcus oralis subsp. oralis MGS:0705, Eggerthella lenta MGS:0225, Bacteroides ovatus MGS:0031, Eubacteriales sp. MGS:0085, Eubacteriales sp. MGS:0084, Actinomyces sp. ICM58 MGS:0410, Bacteroides sp. MGS:0030, Fusobacterium nucleatum subsp. animalis MGS:1418, Pseudoflavonifractor sp. BIOML-A3 MGS:0735, Intestinibacillus sp. Marseille-P4005 MGS:0168, Desulfovibrionales sp. MGS:0462, Dorea formicigenerans MGS:0006, Streptococcus gordonii MGS:0713, Enterocloster clostridioformis MGS:0686, Enterocloster aldenensis MGS:0362, Scardovia wiggsiae MGS:1737, Megamonas funiformis MGS:0684, Streptococcus mutans MGS:0677, Eubacteriales sp. MGS:0128, Parabacteroides johnsonii MGS:0469, Clostridium sp. AT4 MGS:0347, Oscillospiraceae sp. MGS:0256, Gemella morbillorum MGS:1782, Eubacteriales sp. MGS:0335, Porphyromonas sp. MGS:1016, Dorea sp. AF36-15AT MGS:0052, Sutterella sp. KLE1602 MGS:0228, Lachnospiraceae sp. MGS:0625, Clostridium sp. TF06-15AC MGS:0032 | 212.00244[M-H]1- |
| 3-methyl-2-oxovalerate | Clostridium sp. TM06-18 MGS:0048, Bacteroides ovatus MGS:0031, Lachnospiraceae sp. MGS:1271, Desulfovibrio piger MGS:0955, Actinomyces sp. ICM58 MGS:0410, Bacteroides sp. MGS:0030, Intestinibacillus sp. Marseille-P4005 MGS:0168, Desulfovibrionales sp. MGS:0462, Dorea formicigenerans MGS:0006, Enterocloster clostridioformis MGS:0686, Enterocloster aldenensis MGS:0362, Megamonas funiformis MGS:0684, Eubacteriales sp. MGS:0128, Clostridium sp. MGS:0050, Desulfovibrio piger MGS:0240, Anaerobutyricum hallii MGS:0012, Ruminococcus torques MGS:0034, Longibaculum muris MGS:1605, Clostridium sp. AT4 MGS:0347, Oscillospiraceae sp. MGS:0256, Gemella morbillorum MGS:1782, Eubacteriales sp. MGS:0335, Dorea sp. AF36-15AT MGS:0052, Clostridium sp. TF06-15AC MGS:0032 | 148.09679[M+NH4]1+, 131.07032[M+H]1+, 153.05223[M+Na]1+, 169.02609[M+K]1+, 302.93176[M+(NaCl)3-H]1-, 244.97303[M+(NaCl)2-H]1-, 129.05583[M-H]1-, 187.01434[M+NaCl-H]1-, 175.06119[M-H+FA]1-, 165.03234[M+Cl]1- |
| 3-ureidopropionate | Eubacteriales sp. MGS:0085, Eubacteriales sp. MGS:0084, Lachnospiraceae sp. MGS:0389, Eubacteriales sp. MGS:0260, Lachnospiraceae sp. MGS:1209, Gemmiger sp. MGS:0862, Eubacteriales sp. MGS:0671, Clostridia sp. MGS:0845, Clostridia sp. MGS:1035, Eubacteriales sp. MGS:1313, Megasphaera sp. DJF_B143 MGS:1121 | 132.05294[M1+.]1+, 155.0427[M+Na]1+, 171.01662[M+K]1+, 133.0608[M+H]1+, 150.08738[M+NH4]1+, 246.96343[M+(NaCl)2-H]1-, 131.04622[M-H]1-, 189.00492[M+NaCl-H]1- |
| 4-acetamidobutanoate | Dialister pneumosintes MGS:1496, Streptococcus oralis subsp. oralis MGS:0705, Anaerostipes caccae MGS:0747, Lachnospiraceae sp. MGS:1271, Bacteroides sp. MGS:0030, Fusobacterium nucleatum subsp. animalis MGS:1418, Streptococcus gordonii MGS:0713, Lachnospiraceae sp. MGS:1209, Enterocloster clostridioformis MGS:0686, Enterocloster aldenensis MGS:0362, Scardovia wiggsiae MGS:1737, Streptococcus mutans MGS:0677, Eubacteriales sp. MGS:0128, Ruminococcus torques MGS:0034, Clostridium sp. AT4 MGS:0347, Oscillospiraceae sp. MGS:0256, Gemella morbillorum MGS:1782, Lachnospiraceae sp. MGS:0625 | 146.08117[M+H]1+, 168.06313[M+Na]1+, 184.03708[M+K]1+, 144.06662[M-H]1- |
| 4-guanidinobutanoate | Eubacteriales sp. MGS:0084, Lachnospiraceae sp. MGS:0389, Eubacteriales sp. MGS:0260, Slackia isoflavoniconvertens MGS:0160, Streptococcus gordonii MGS:0713, Lachnospiraceae sp. MGS:1209, Bifidobacterium catenulatum subsp. kashiwanohense MGS:0292, Streptococcus mutans MGS:0677, Eubacteriales sp. MGS:0671, Clostridia sp. MGS:0845, Clostridia sp. MGS:1035 | 146.09244[M+H]1+, 163.11865[M+NH4]1+, 168.07437[M+Na]1+ |
| 4-hydroxyphenylacetate | Dialister pneumosintes MGS:1496, Streptococcus oralis subsp. oralis MGS:0705, Eubacteriales sp. MGS:0084, Lachnospiraceae sp. MGS:0389, Actinomyces sp. ICM58 MGS:0410, Fusobacterium nucleatum subsp. animalis MGS:1418, Streptococcus gordonii MGS:0713, Scardovia wiggsiae MGS:1737, Oscillospiraceae sp. MGS:0074, Streptococcus mutans MGS:0677 | 153.05463[M+H]1+, 175.03661[M+Na]1+, 151.04002[M-H]1- |
| 4-hydroxyphenylpyruvate | Eubacteriales sp. MGS:1167, Clostridium sp. TM06-18 MGS:0048, Slackia isoflavoniconvertens MGS:0160, Dorea formicigenerans MGS:0006, Streptococcus gordonii MGS:0713, Bifidobacterium catenulatum subsp. kashiwanohense MGS:0292, Streptococcus mutans MGS:0677, Clostridium sp. MGS:0050, Clostridia sp. MGS:1035, Porphyromonas sp. MGS:1016 | 180.04207[M1+.]1+, 203.0318[M+Na]1+, 179.03499[M-H]1- |
| 4-methyl-2-oxopentanoate | Clostridium sp. TM06-18 MGS:0048, Bacteroides ovatus MGS:0031, Lachnospiraceae sp. MGS:1271, Desulfovibrio piger MGS:0955, Actinomyces sp. ICM58 MGS:0410, Bacteroides sp. MGS:0030, Intestinibacillus sp. Marseille-P4005 MGS:0168, Dorea formicigenerans MGS:0006, Enterocloster aldenensis MGS:0362, Megamonas funiformis MGS:0684, Eubacteriales sp. MGS:0128, Clostridium sp. MGS:0050, Anaerobutyricum hallii MGS:0012, Ruminococcus torques MGS:0034, Longibaculum muris MGS:1605, Clostridium sp. AT4 MGS:0347, Oscillospiraceae sp. MGS:0256, Gemella morbillorum MGS:1782, Eubacteriales sp. MGS:0335, Dorea sp. AF36-15AT MGS:0052, Sutterella sp. KLE1602 MGS:0228, Clostridium sp. TF06-15AC MGS:0032 | 148.09679[M+NH4]1+, 131.07032[M+H]1+, 153.05223[M+Na]1+, 169.02609[M+K]1+, 302.93176[M+(NaCl)3-H]1-, 244.97303[M+(NaCl)2-H]1-, 129.05583[M-H]1-, 187.01434[M+NaCl-H]1-, 175.06119[M-H+FA]1-, 165.03234[M+Cl]1- |
| 4-vinylphenol sulfate | Bacteria sp. MGS:0575, Lachnospiraceae sp. MGS:0389, Streptococcus mutans MGS:0677, Sutterella seckii MGS:0997, Bacteria sp. MGS:0459 | 258.98013[M+NaCl+H]1+, 316.9387[M+(NaCl)2+H]1+, 199.00713[M-H]1- |
| 5,6-dihydrothymine | Eubacteriales sp. MGS:0084, Lachnospiraceae sp. MGS:0389, Lachnospiraceae sp. MGS:1209, Gemmiger sp. MGS:0862, Eubacteriales sp. MGS:0671 | 151.0478[M+Na]1+, 129.0658[M+H]1+, 146.09244[M+NH4]1+, 127.05134[M-H]1-, 173.05672[M-H+FA]1- |
| 5,6-dihydrouridine | Streptococcus oralis subsp. oralis MGS:0705, Clostridium sp. TM06-18 MGS:0048, Eggerthella lenta MGS:0225, Fusobacterium nucleatum subsp. animalis MGS:1418, Intestinibacillus sp. Marseille-P4005 MGS:0168, Slackia isoflavoniconvertens MGS:0160, Streptococcus gordonii MGS:0713, Enterocloster aldenensis MGS:0362, Scardovia wiggsiae MGS:1737, Streptococcus mutans MGS:0677, Eubacteriales sp. MGS:0128, Ruminococcus torques MGS:0034, Clostridium sp. AT4 MGS:0347, Oscillospiraceae sp. MGS:0256, Dorea sp. AF36-15AT MGS:0052 | 269.07442[M+Na]1+, 281.05475[M+Cl]1-, 291.08363[M-H+FA]1- |
| 5-dodecenoate (12:1n7) | Streptococcus oralis subsp. oralis MGS:0705, Anaerostipes caccae MGS:0747, Eggerthella lenta MGS:0225, Butyricicoccus pullicaecorum MGS:1374, Bacteroides sp. MGS:0030, Fusobacterium nucleatum subsp. animalis MGS:1418, Intestinibacillus sp. Marseille-P4005 MGS:0168, Bifidobacterium catenulatum subsp. kashiwanohense MGS:0292, Scardovia wiggsiae MGS:1737, Gemella morbillorum MGS:1782, Saccharomyces cerevisiae MGS:0782, Dorea sp. AF36-15AT MGS:0052, Sutterella sp. KLE1602 MGS:0228, Bacteria sp. MGS:0459 | 199.1693[M+H]1+, 216.19589[M+NH4]1+, 221.15125[M+Na]1+, 197.15478[M-H]1-, 243.16009[M-H+FA]1- |
| 5-methyluridine (ribothymidine) | Eubacteriales sp. MGS:1167, Bacteria sp. MGS:0459 | 276.11906[M+NH4]1+, 490.92608[M+(NaCl)4+H]1+ |
| 5-oxoproline | Lachnospiraceae sp. MGS:0389, Ruminococcus callidus MGS:0247, Streptococcus mutans MGS:0677, Eubacteriales sp. MGS:0671 | 152.03183[M+Na]1+, 168.00575[M+K]1+, 130.04993[M+H]1+, 147.07644[M+NH4]1+, 128.03537[M-H]1-, 185.99394[M+NaCl-H]1-, 174.04077[M-H+FA]1-, 243.95258[M+(NaCl)2-H]1- |
| 6-hydroxyindole sulfate | Dialister pneumosintes MGS:1496, Bacteroides ovatus MGS:0031, Eubacteriales sp. MGS:0085, Eubacteriales sp. MGS:0084, Actinomyces sp. ICM58 MGS:0410, Bacteroides sp. MGS:0030, Fusobacterium nucleatum subsp. animalis MGS:1418, Pseudoflavonifractor sp. BIOML-A3 MGS:0735, Intestinibacillus sp. Marseille-P4005 MGS:0168, Desulfovibrionales sp. MGS:0462, Dorea formicigenerans MGS:0006, Enterocloster clostridioformis MGS:0686, Enterocloster aldenensis MGS:0362, Scardovia wiggsiae MGS:1737, Megamonas funiformis MGS:0684, Streptococcus mutans MGS:0677, Eubacteriales sp. MGS:0128, Parabacteroides johnsonii MGS:0469, Clostridium sp. AT4 MGS:0347, Oscillospiraceae sp. MGS:0256, Eubacteriales sp. MGS:0335, Porphyromonas sp. MGS:1016, Dorea sp. AF36-15AT MGS:0052, Sutterella sp. KLE1602 MGS:0228, Clostridium sp. TF06-15AC MGS:0032 | 212.00244[M-H]1- |
| 7-methylguanine | Streptococcus oralis subsp. oralis MGS:0705, Clostridium sp. TM06-18 MGS:0048, Anaerostipes caccae MGS:0747, Intestinibacillus sp. Marseille-P4005 MGS:0168, Streptococcus gordonii MGS:0713, Bifidobacterium catenulatum subsp. kashiwanohense MGS:0292, Scardovia wiggsiae MGS:1737, Oscillospiraceae sp. MGS:0074, Streptococcus mutans MGS:0677, Eubacteriales sp. MGS:0128, Clostridium sp. MGS:0050, Anaerobutyricum hallii MGS:0012, Ruminococcus torques MGS:0034, Parabacteroides johnsonii MGS:0469, Oscillospiraceae sp. MGS:0256, Gemella morbillorum MGS:1782, Dorea sp. AF36-15AT MGS:0052 | 166.07243[M+H]1+, 183.09922[M+NH4]1+ |
| 9-hydroxystearate | Streptococcus oralis subsp. oralis MGS:0705, Clostridium sp. TM06-18 MGS:0048, Anaerostipes caccae MGS:0747, Butyricicoccus pullicaecorum MGS:1374, Desulfovibrio piger MGS:0955, Actinomyces sp. ICM58 MGS:0410, Intestinibacillus sp. Marseille-P4005 MGS:0168, Dorea formicigenerans MGS:0006, Streptococcus gordonii MGS:0713, Scardovia wiggsiae MGS:1737, Streptococcus mutans MGS:0677, Eubacteriales sp. MGS:0128, Clostridium sp. MGS:0050, Anaerobutyricum hallii MGS:0012, Ruminococcus torques MGS:0034, Clostridium sp. AT4 MGS:0347, Oscillospiraceae sp. MGS:0256, Gemella morbillorum MGS:1782, Saccharomyces cerevisiae MGS:0782, Eubacteriales sp. MGS:0335, Dorea sp. AF36-15AT MGS:0052, Sutterella sp. KLE1602 MGS:0228 | 323.25564[M+Na]1+, 339.22939[M+K]1+, 299.25946[M-H]1-, 335.23649[M+Cl]1- |
| N,N,N-trimethyl-alanylproline betaine (TMAP) | Eubacteriales sp. MGS:1167, Eubacteriales sp. MGS:0085, Eubacteriales sp. MGS:0084, Lachnospiraceae sp. MGS:1209, Bifidobacterium catenulatum subsp. kashiwanohense MGS:0292, Oscillospiraceae sp. MGS:0074, Eubacteriales sp. MGS:0103, Eubacteriales sp. MGS:0671, Clostridia sp. MGS:1035, Porphyromonas sp. MGS:1016 | 267.11049[M+K]1+, 251.13655[M+Na]1+, 229.15453[M+H]1+ |
| N-acetyl-beta-alanine | Streptococcus oralis subsp. oralis MGS:0705, Anaerostipes caccae MGS:0747, Intestinibacillus sp. Marseille-P4005 MGS:0168, Desulfovibrionales sp. MGS:0462, Lachnospiraceae sp. MGS:1209, Eubacteriales sp. MGS:0128, Anaerobutyricum hallii MGS:0012, Ruminococcus torques MGS:0034, Gemmiger sp. MGS:0862, Parabacteroides johnsonii MGS:0469, Oscillospiraceae sp. MGS:0256, Gemella morbillorum MGS:1782, Dorea sp. AF36-15AT MGS:0052, Sutterella sp. KLE1602 MGS:0228, Clostridium sp. TF06-15AC MGS:0032 | 154.04746[M+Na]1+, 170.02135[M+K]1+, 132.06556[M+H]1+, 166.02763[M+Cl]1-, 130.05096[M-H]1-, 176.05641[M-H+FA]1- |
| N-acetylalanine | Streptococcus oralis subsp. oralis MGS:0705, Anaerostipes caccae MGS:0747, Eggerthella lenta MGS:0225, Fusobacterium nucleatum subsp. animalis MGS:1418, Dorea formicigenerans MGS:0006, Streptococcus gordonii MGS:0713, Enterocloster clostridioformis MGS:0686, Enterocloster aldenensis MGS:0362, Scardovia wiggsiae MGS:1737, Streptococcus mutans MGS:0677, Ruminococcus torques MGS:0034, Longibaculum muris MGS:1605, Oscillospiraceae sp. MGS:0256, Gemella morbillorum MGS:1782, Saccharomyces cerevisiae MGS:0782, Porphyromonas sp. MGS:1016, Dorea sp. AF36-15AT MGS:0052 | 154.04746[M+Na]1+, 170.02135[M+K]1+, 132.06556[M+H]1+, 166.02763[M+Cl]1-, 130.05096[M-H]1-, 176.05641[M-H+FA]1- |
| N-acetylneuraminate | Streptococcus oralis subsp. oralis MGS:0705, Anaerostipes caccae MGS:0747, Eggerthella lenta MGS:0225, Bacteroides sp. MGS:0030, Fusobacterium nucleatum subsp. animalis MGS:1418, Intestinibacillus sp. Marseille-P4005 MGS:0168, Dorea formicigenerans MGS:0006, Enterocloster clostridioformis MGS:0686, Enterocloster aldenensis MGS:0362, Scardovia wiggsiae MGS:1737, Streptococcus mutans MGS:0677, Eubacteriales sp. MGS:0128, Clostridium sp. MGS:0050, Anaerobutyricum hallii MGS:0012, Clostridium sp. AT4 MGS:0347, Oscillospiraceae sp. MGS:0256, Gemella morbillorum MGS:1782, Saccharomyces cerevisiae MGS:0782, Dorea sp. AF36-15AT MGS:0052 | 332.09515[M+Na]1+, 308.09916[M-H]1-, 344.07554[M+Cl]1- |
| N-acetylthreonine | Dialister pneumosintes MGS:1496, Streptococcus oralis subsp. oralis MGS:0705, Streptococcus gordonii MGS:0713, Lachnospiraceae sp. MGS:1209, Bifidobacterium catenulatum subsp. kashiwanohense MGS:0292, Ruminococcus callidus MGS:0247, Scardovia wiggsiae MGS:1737, Streptococcus mutans MGS:0677, Eubacteriales sp. MGS:0103, Porphyromonas sp. MGS:1016 | 184.05806[M+Na]1+, 200.03206[M+K]1+, 162.07609[M+H]1+, 160.06156[M-H]1- |
| N-acetylvaline | Dialister pneumosintes MGS:1496, Streptococcus oralis subsp. oralis MGS:0705, Anaerostipes caccae MGS:0747, Eggerthella lenta MGS:0225, Bacteroides sp. MGS:0030, Fusobacterium nucleatum subsp. animalis MGS:1418, Intestinibacillus sp. Marseille-P4005 MGS:0168, Dorea formicigenerans MGS:0006, Enterocloster aldenensis MGS:0362, Scardovia wiggsiae MGS:1737, Streptococcus mutans MGS:0677, Eubacteriales sp. MGS:0128, Ruminococcus torques MGS:0034, Longibaculum muris MGS:1605, Clostridium sp. AT4 MGS:0347, Oscillospiraceae sp. MGS:0256, Dorea sp. AF36-15AT MGS:0052, Clostridium sp. TF06-15AC MGS:0032 | 159.08931[M1+.]1+, 160.09684[M+H]1+, 198.05264[M+K]1+, 158.08219[M-H]1- |
| N-delta-acetylornithine | Eubacteriales sp. MGS:1167, Bacteroides ovatus MGS:0031, Eubacteriales sp. MGS:0085, Eubacteriales sp. MGS:0084, Lachnospiraceae sp. MGS:0389, Eubacteriales sp. MGS:0260, Lachnospiraceae sp. MGS:1209, Eubacteriales sp. MGS:0671 | 192.13422[M+NH4]1+, 197.08973[M+Na]1+, 173.09315[M-H]1- |
| N-lactoyl leucine | Streptococcus oralis subsp. oralis MGS:0705, Clostridium sp. TM06-18 MGS:0048, Anaerostipes caccae MGS:0747, Eggerthella lenta MGS:0225, Actinomyces sp. ICM58 MGS:0410, Bacteroides sp. MGS:0030, Fusobacterium nucleatum subsp. animalis MGS:1418, Intestinibacillus sp. Marseille-P4005 MGS:0168, Dorea formicigenerans MGS:0006, Enterocloster clostridioformis MGS:0686, Enterocloster aldenensis MGS:0362, Megamonas funiformis MGS:0684, Streptococcus mutans MGS:0677, Eubacteriales sp. MGS:0128, Clostridium sp. MGS:0050, Anaerobutyricum hallii MGS:0012, Ruminococcus torques MGS:0034, Longibaculum muris MGS:1605, Clostridium sp. AT4 MGS:0347, Oscillospiraceae sp. MGS:0256, Gemella morbillorum MGS:1782, Eubacteriales sp. MGS:0335, Dorea sp. AF36-15AT MGS:0052, Clostridium sp. TF06-15AC MGS:0032 | 242.0788[M+K]1+, 226.10492[M+Na]1+, 204.12306[M+H]1+ |
| N-lactoyl valine | Streptococcus oralis subsp. oralis MGS:0705, Clostridium sp. TM06-18 MGS:0048, Anaerostipes caccae MGS:0747, Fusobacterium nucleatum subsp. animalis MGS:1418, Intestinibacillus sp. Marseille-P4005 MGS:0168, Dorea formicigenerans MGS:0006, Streptococcus gordonii MGS:0713, Enterocloster clostridioformis MGS:0686, Enterocloster aldenensis MGS:0362, Scardovia wiggsiae MGS:1737, Streptococcus mutans MGS:0677, Eubacteriales sp. MGS:0128, Clostridium sp. MGS:0050, Anaerobutyricum hallii MGS:0012, Ruminococcus torques MGS:0034, Longibaculum muris MGS:1605, Clostridium sp. AT4 MGS:0347, Oscillospiraceae sp. MGS:0256, Gemella morbillorum MGS:1782, Dorea sp. AF36-15AT MGS:0052, Sutterella sp. KLE1602 MGS:0228, Clostridium sp. TF06-15AC MGS:0032 | 228.06314[M+K]1+, 190.10754[M+H]1+, 212.08931[M+Na]1+ |
| N6,N6,N6-trimethyllysine | Dialister pneumosintes MGS:1496, Eubacteriales sp. MGS:1167, Streptococcus oralis subsp. oralis MGS:0705, Lachnospiraceae sp. MGS:0389, Pseudoflavonifractor sp. BIOML-A3 MGS:0735, Streptococcus gordonii MGS:0713, Scardovia wiggsiae MGS:1737, Streptococcus mutans MGS:0677, Eubacteriales sp. MGS:0103, Ruminococcus torques MGS:0034, Gemella morbillorum MGS:1782 | 189.15974[M+H]1+, 211.14166[M+Na]1+ |
| N6,N6-dimethyllysine | Lachnospiraceae sp. MGS:1271, Scardovia wiggsiae MGS:1737, Streptococcus mutans MGS:0677 | 175.14409[M+H]1+, 197.12616[M+Na]1+ |
| S-1-pyrroline-5-carboxylate | Eubacteriales sp. MGS:0085, Bifidobacterium catenulatum subsp. kashiwanohense MGS:0292, Clostridia sp. MGS:0845 | 136.03701[M+Na]1+, 158.04586[M-H+FA]1-, 112.04042[M-H]1- |
| S-methylcysteine sulfoxide | Bacteroides ovatus MGS:0031, Eubacteriales sp. MGS:0085, Eubacteriales sp. MGS:0084, Lachnospiraceae sp. MGS:0389, Bacteroides sp. MGS:0030, Lachnospiraceae sp. MGS:1209, Clostridia sp. MGS:0845 | 209.9962[M+NaCl+H]1+, 174.01951[M+Na]1+, 152.03757[M+H]1+ |
| acetoacetate | Butyricicoccus pullicaecorum MGS:1374, Bacteroides sp. MGS:0030, Fusobacterium nucleatum subsp. animalis MGS:1418 | 103.03896[M+H]1+, 140.99485[M+K]1+, 125.02096[M+Na]1+, 120.06552[M+NH4]1+, 137.00098[M+Cl]1-, 147.02988[M-H+FA]1-, 158.98303[M+NaCl-H]1-, 101.02439[M-H]1- |
| acisoga | Dialister pneumosintes MGS:1496, Clostridium sp. TM06-18 MGS:0048, Bacteroides ovatus MGS:0031, Bacteroides sp. MGS:0030, Enterocloster aldenensis MGS:0362, Desulfovibrio piger MGS:0240, Clostridia sp. MGS:0845, Bacteria sp. MGS:0459, Clostridium sp. TF06-15AC MGS:0032 | 185.12845[M+H]1+, 207.11043[M+Na]1+ |
| allantoin | Eubacteriales sp. MGS:0085, Eubacteriales sp. MGS:0084, Longibaculum muris MGS:1605, Clostridia sp. MGS:1035, Eubacteriales sp. MGS:1313 | 181.03319[M+Na]1+, 157.03662[M-H]1- |
| alpha-hydroxyisovalerate | Dialister pneumosintes MGS:1496, Streptococcus oralis subsp. oralis MGS:0705, Clostridium sp. TM06-18 MGS:0048, Anaerostipes caccae MGS:0747, Bacteroides ovatus MGS:0031, Lachnospiraceae sp. MGS:1271, Butyricicoccus pullicaecorum MGS:1374, Desulfovibrio piger MGS:0955, Actinomyces sp. ICM58 MGS:0410, Bacteroides sp. MGS:0030, Intestinibacillus sp. Marseille-P4005 MGS:0168, Slackia isoflavoniconvertens MGS:0160, Desulfovibrionales sp. MGS:0462, Dorea formicigenerans MGS:0006, Enterocloster clostridioformis MGS:0686, Enterocloster aldenensis MGS:0362, Megamonas funiformis MGS:0684, Eubacteriales sp. MGS:0128, Clostridium sp. MGS:0050, Desulfovibrio piger MGS:0240, Ruminococcus torques MGS:0034, Gemmiger sp. MGS:0862, Parabacteroides johnsonii MGS:0469, Longibaculum muris MGS:1605, Clostridium sp. AT4 MGS:0347, Gemella morbillorum MGS:1782, Eubacteriales sp. MGS:0335, Dorea sp. AF36-15AT MGS:0052, Sutterella sp. KLE1602 MGS:0228, Bacteria sp. MGS:0459, Clostridium sp. TF06-15AC MGS:0032 | 119.07029[M+H]1+, 141.0522[M+Na]1+, 157.02605[M+K]1+, 117.05576[M-H]1-, 175.01426[M+NaCl-H]1-, 153.03241[M+Cl]1-, 163.06124[M-H+FA]1- |
| alpha-ketobutyrate | Clostridium sp. TM06-18 MGS:0048, Lachnospiraceae sp. MGS:1271, Butyricicoccus pullicaecorum MGS:1374, Desulfovibrio piger MGS:0955, Actinomyces sp. ICM58 MGS:0410, Bacteroides sp. MGS:0030, Intestinibacillus sp. Marseille-P4005 MGS:0168, Dorea formicigenerans MGS:0006, Enterocloster aldenensis MGS:0362, Eubacteriales sp. MGS:0128, Clostridium sp. MGS:0050, Anaerobutyricum hallii MGS:0012, Ruminococcus torques MGS:0034, Longibaculum muris MGS:1605, Clostridium sp. AT4 MGS:0347, Oscillospiraceae sp. MGS:0256, Gemella morbillorum MGS:1782, Eubacteriales sp. MGS:0335, Dorea sp. AF36-15AT MGS:0052, Sutterella sp. KLE1602 MGS:0228, Lachnospiraceae sp. MGS:0625, Clostridium sp. TF06-15AC MGS:0032 | 103.03896[M+H]1+, 140.99485[M+K]1+, 125.02096[M+Na]1+, 120.06552[M+NH4]1+, 137.00098[M+Cl]1-, 147.02988[M-H+FA]1-, 158.98303[M+NaCl-H]1-, 101.02439[M-H]1- |
| alpha-ketoglutaramate* | Eubacteriales sp. MGS:1167, Eubacteriales sp. MGS:0085, Eubacteriales sp. MGS:0260, Lachnospiraceae sp. MGS:1209, Eubacteriales sp. MGS:0671, Clostridia sp. MGS:0845, Clostridia sp. MGS:1035 | 144.03026[M-H]1-, 190.03578[M-H+FA]1- |
| alpha-ketoglutarate | Dialister pneumosintes MGS:1496, Streptococcus oralis subsp. oralis MGS:0705, Clostridium sp. TM06-18 MGS:0048, Anaerostipes caccae MGS:0747, Desulfovibrio piger MGS:0955, Actinomyces sp. ICM58 MGS:0410, Bacteroides sp. MGS:0030, Fusobacterium nucleatum subsp. animalis MGS:1418, Intestinibacillus sp. Marseille-P4005 MGS:0168, Dorea formicigenerans MGS:0006, Streptococcus gordonii MGS:0713, Enterocloster clostridioformis MGS:0686, Enterocloster aldenensis MGS:0362, Scardovia wiggsiae MGS:1737, Streptococcus mutans MGS:0677, Eubacteriales sp. MGS:0128, Clostridium sp. MGS:0050, Desulfovibrio piger MGS:0240, Anaerobutyricum hallii MGS:0012, Ruminococcus torques MGS:0034, Parabacteroides johnsonii MGS:0469, Longibaculum muris MGS:1605, Clostridium sp. AT4 MGS:0347, Oscillospiraceae sp. MGS:0256, Gemella morbillorum MGS:1782, Eubacteriales sp. MGS:0335, Dorea sp. AF36-15AT MGS:0052, Sutterella sp. KLE1602 MGS:0228, Lachnospiraceae sp. MGS:0625, Clostridium sp. TF06-15AC MGS:0032 | 262.94591[M+(NaCl)2+H]1+, 320.90454[M+(NaCl)3+H]1+, 378.86309[M+(NaCl)4+H]1+, 436.82163[M+(NaCl)5+H]1+, 145.01427[M-H]1-, 191.01981[M-H+FA]1- |
| arachidonate (20:4n6) | Clostridium sp. TM06-18 MGS:0048, Anaerostipes caccae MGS:0747, Eggerthella lenta MGS:0225, Lachnospiraceae sp. MGS:1271, Desulfovibrio piger MGS:0955, Actinomyces sp. ICM58 MGS:0410, Bacteroides sp. MGS:0030, Intestinibacillus sp. Marseille-P4005 MGS:0168, Dorea formicigenerans MGS:0006, Enterocloster clostridioformis MGS:0686, Enterocloster aldenensis MGS:0362, Megamonas funiformis MGS:0684, Eubacteriales sp. MGS:0128, Desulfovibrio piger MGS:0240, Anaerobutyricum hallii MGS:0012, Clostridium sp. AT4 MGS:0347, Oscillospiraceae sp. MGS:0256, Eubacteriales sp. MGS:0335, Dorea sp. AF36-15AT MGS:0052, Lachnospiraceae sp. MGS:0625, Bacteria sp. MGS:0459, Clostridium sp. TF06-15AC MGS:0032 | 303.23333[M-H]1-, 339.20992[M+Cl]1- |
| argininate* | Eubacteriales sp. MGS:1167, Bacteroides ovatus MGS:0031, Eubacteriales sp. MGS:0085, Bacteria sp. MGS:0575, Eubacteriales sp. MGS:0084, Lachnospiraceae sp. MGS:0389, Eubacteriales sp. MGS:0260, Lachnospiraceae sp. MGS:1209, Ruminococcus callidus MGS:0247, Gemmiger sp. MGS:0862, Eubacteriales sp. MGS:0671, Clostridia sp. MGS:0845, Clostridia sp. MGS:1035, Clostridium sp. TF06-15AC MGS:0032 | 198.08497[M+Na]1+, 176.10295[M+H]1+, 349.9795[M+(NaCl)3+H]1+, 292.02074[M+(NaCl)2+H]1+, 214.05884[M+K]1+, 174.08838[M-H]1-, 290.0059[M+(NaCl)2-H]1-, 232.04696[M+NaCl-H]1-, 210.06513[M+Cl]1- |
| asparagine | Eubacteriales sp. MGS:1167, Eubacteriales sp. MGS:0671, Clostridia sp. MGS:0845, Clostridia sp. MGS:1035, Eubacteriales sp. MGS:1313 | 132.05294[M1+.]1+, 155.0427[M+Na]1+, 171.01662[M+K]1+, 133.0608[M+H]1+, 150.08738[M+NH4]1+, 246.96343[M+(NaCl)2-H]1-, 131.04622[M-H]1-, 189.00492[M+NaCl-H]1- |
| benzoate | Eubacteriales sp. MGS:0085, Eubacteriales sp. MGS:0084 | 123.04394[M+H]1+, 145.02617[M+Na]1+, 167.03489[M-H+FA]1-, 157.00621[M+Cl]1-, 121.02952[M-H]1- |
| beta-hydroxyisovalerate | Streptococcus oralis subsp. oralis MGS:0705, Clostridium sp. TM06-18 MGS:0048, Anaerostipes caccae MGS:0747, Eggerthella lenta MGS:0225, Lachnospiraceae sp. MGS:1271, Butyricicoccus pullicaecorum MGS:1374, Bacteroides sp. MGS:0030, Intestinibacillus sp. Marseille-P4005 MGS:0168, Dorea formicigenerans MGS:0006, Enterocloster clostridioformis MGS:0686, Enterocloster aldenensis MGS:0362, Eubacteriales sp. MGS:0128, Clostridium sp. MGS:0050, Anaerobutyricum hallii MGS:0012, Ruminococcus torques MGS:0034, Gemmiger sp. MGS:0862, Parabacteroides johnsonii MGS:0469, Clostridium sp. AT4 MGS:0347, Oscillospiraceae sp. MGS:0256, Gemella morbillorum MGS:1782, Eubacteriales sp. MGS:0335, Dorea sp. AF36-15AT MGS:0052, Clostridium sp. TF06-15AC MGS:0032 | 119.07029[M+H]1+, 141.0522[M+Na]1+, 157.02605[M+K]1+, 117.05576[M-H]1-, 175.01426[M+NaCl-H]1-, 153.03241[M+Cl]1-, 163.06124[M-H+FA]1- |
| butyrate/isobutyrate (4:0) | Bacteroides ovatus MGS:0031, Eubacteriales sp. MGS:0085, Bacteria sp. MGS:0575, Eubacteriales sp. MGS:0084, Lachnospiraceae sp. MGS:0389, Oscillospiraceae sp. MGS:0074, Streptococcus mutans MGS:0677, Eubacteriales sp. MGS:0103 | 106.08625[M+NH4]1+, 147.01836[M+NaCl+H]1+, 127.01562[M+K]1+, 89.05968[M+H]1+, 111.04165[M+Na]1+, 133.05065[M-H+FA]1-, 87.04515[M-H]1- |
| butyrylcarnitine (C4) | Streptococcus oralis subsp. oralis MGS:0705, Clostridium sp. TM06-18 MGS:0048, Anaerostipes caccae MGS:0747, Desulfovibrio piger MGS:0955, Actinomyces sp. ICM58 MGS:0410, Bacteroides sp. MGS:0030, Fusobacterium nucleatum subsp. animalis MGS:1418, Intestinibacillus sp. Marseille-P4005 MGS:0168, Slackia isoflavoniconvertens MGS:0160, Dorea formicigenerans MGS:0006, Streptococcus gordonii MGS:0713, Enterocloster clostridioformis MGS:0686, Scardovia wiggsiae MGS:1737, Megamonas funiformis MGS:0684, Streptococcus mutans MGS:0677, Eubacteriales sp. MGS:0128, Clostridium sp. MGS:0050, Desulfovibrio piger MGS:0240, Anaerobutyricum hallii MGS:0012, Ruminococcus torques MGS:0034, Clostridium sp. AT4 MGS:0347, Oscillospiraceae sp. MGS:0256, Gemella morbillorum MGS:1782, Dorea sp. AF36-15AT MGS:0052, Sutterella sp. KLE1602 MGS:0228, Clostridium sp. TF06-15AC MGS:0032 | 232.15431[M+H]1+, 254.13625[M+Na]1+ |
| carnitine | Streptococcus oralis subsp. oralis MGS:0705, Clostridium sp. TM06-18 MGS:0048, Anaerostipes caccae MGS:0747, Actinomyces sp. ICM58 MGS:0410, Fusobacterium nucleatum subsp. animalis MGS:1418, Intestinibacillus sp. Marseille-P4005 MGS:0168, Slackia isoflavoniconvertens MGS:0160, Desulfovibrionales sp. MGS:0462, Dorea formicigenerans MGS:0006, Streptococcus mutans MGS:0677, Eubacteriales sp. MGS:0128, Clostridium sp. MGS:0050, Desulfovibrio piger MGS:0240, Anaerobutyricum hallii MGS:0012, Ruminococcus torques MGS:0034, Gemmiger sp. MGS:0862, Clostridium sp. AT4 MGS:0347, Oscillospiraceae sp. MGS:0256, Saccharomyces cerevisiae MGS:0782, Eubacteriales sp. MGS:0335, Dorea sp. AF36-15AT MGS:0052, Sutterella sp. KLE1602 MGS:0228, Clostridium sp. TF06-15AC MGS:0032 | 162.11244[M1+.]1+ |
| chenodeoxycholate | Dialister pneumosintes MGS:1496, Streptococcus oralis subsp. oralis MGS:0705, Bacteria sp. MGS:0575, Lachnospiraceae sp. MGS:0389, Lachnospiraceae sp. MGS:1271, Butyricicoccus pullicaecorum MGS:1374, Actinomyces sp. ICM58 MGS:0410, Fusobacterium nucleatum subsp. animalis MGS:1418, Eubacteriales sp. MGS:0260, Slackia isoflavoniconvertens MGS:0160, Streptococcus gordonii MGS:0713, Bifidobacterium catenulatum subsp. kashiwanohense MGS:0292, Ruminococcus callidus MGS:0247, Scardovia wiggsiae MGS:1737, Oscillospiraceae sp. MGS:0074, Streptococcus mutans MGS:0677, Eubacteriales sp. MGS:0103, Gemmiger sp. MGS:0862, Eubacteriales sp. MGS:0671, Clostridia sp. MGS:1035, Gemella morbillorum MGS:1782, Eubacteriales sp. MGS:1313, Megasphaera sp. DJF_B143 MGS:1121 | 415.28176[M+Na]1+ |
| cinnamoylglycine | Eubacteriales sp. MGS:1167, Eubacteriales sp. MGS:0085, Bacteria sp. MGS:0575, Eubacteriales sp. MGS:0084, Lachnospiraceae sp. MGS:0389, Pseudoflavonifractor sp. BIOML-A3 MGS:0735, Eubacteriales sp. MGS:0260, Slackia isoflavoniconvertens MGS:0160, Desulfovibrionales sp. MGS:0462, Oscillospiraceae sp. MGS:0074, Eubacteriales sp. MGS:0103, Desulfovibrio piger MGS:0240, Eubacteriales sp. MGS:0671, Clostridia sp. MGS:0845, Clostridia sp. MGS:1035, Eubacteriales sp. MGS:1313 | 228.06314[M+Na]1+, 204.06658[M-H]1- |
| cis-4-decenoate (10:1n6)* | Lachnospiraceae sp. MGS:0389, Clostridia sp. MGS:0845 | 229.09654[M+NaCl+H]1+, 171.13798[M+H]1+, 188.1645[M+NH4]1+, 209.09393[M+K]1+, 193.11999[M+Na]1+, 169.12338[M-H]1-, 215.12872[M-H+FA]1- |
| cis-4-decenoylcarnitine (C10:1) | Lachnospiraceae sp. MGS:0389, Clostridia sp. MGS:0845 | 314.23257[M+H]1+ |
| citramalate | Streptococcus oralis subsp. oralis MGS:0705, Clostridium sp. TM06-18 MGS:0048, Desulfovibrio piger MGS:0955, Actinomyces sp. ICM58 MGS:0410, Intestinibacillus sp. Marseille-P4005 MGS:0168, Eubacteriales sp. MGS:0128, Desulfovibrio piger MGS:0240, Anaerobutyricum hallii MGS:0012, Oscillospiraceae sp. MGS:0256, Dorea sp. AF36-15AT MGS:0052, Bacteria sp. MGS:0459 | 171.02645[M+Na]1+, 148.03692[M1+.]1+, 193.03537[M-H+FA]1-, 147.02988[M-H]1- |
| citrate | Eubacteriales sp. MGS:1167, Bacteroides sp. MGS:0030, Pseudoflavonifractor sp. BIOML-A3 MGS:0735, Lachnospiraceae sp. MGS:1209, Enterocloster aldenensis MGS:0362, Eubacteriales sp. MGS:0671, Clostridia sp. MGS:0845, Porphyromonas sp. MGS:1016, Bacteria sp. MGS:0459 | 230.99018[M+K]1+, 215.01617[M+Na]1+, 191.01981[M-H]1-, 248.97839[M+NaCl-H]1-, 364.89598[M+(NaCl)3-H]1-, 306.93734[M+(NaCl)2-H]1- |
| citrulline | Eubacteriales sp. MGS:0085, Clostridia sp. MGS:1035, Bacteria sp. MGS:0459 | 198.08497[M+Na]1+, 176.10295[M+H]1+, 349.9795[M+(NaCl)3+H]1+, 292.02074[M+(NaCl)2+H]1+, 214.05884[M+K]1+, 174.08838[M-H]1-, 290.0059[M+(NaCl)2-H]1-, 232.04696[M+NaCl-H]1-, 210.06513[M+Cl]1- |
| cotinine | Streptococcus oralis subsp. oralis MGS:0705, Anaerostipes caccae MGS:0747, Eggerthella lenta MGS:0225, Desulfovibrio piger MGS:0955, Intestinibacillus sp. Marseille-P4005 MGS:0168, Dorea formicigenerans MGS:0006, Enterocloster clostridioformis MGS:0686, Scardovia wiggsiae MGS:1737, Megamonas funiformis MGS:0684, Streptococcus mutans MGS:0677, Eubacteriales sp. MGS:0128, Clostridium sp. MGS:0050, Desulfovibrio piger MGS:0240, Anaerobutyricum hallii MGS:0012, Ruminococcus torques MGS:0034, Parabacteroides johnsonii MGS:0469, Clostridium sp. AT4 MGS:0347, Oscillospiraceae sp. MGS:0256, Eubacteriales sp. MGS:0335, Megasphaera sp. DJF_B143 MGS:1121, Dorea sp. AF36-15AT MGS:0052, Sutterella sp. KLE1602 MGS:0228, Bacteria sp. MGS:0459 | 177.10216[M+H]1+ |
| creatinine | Pseudoflavonifractor sp. BIOML-A3 MGS:0735, Eubacteriales sp. MGS:0103, Porphyromonas sp. MGS:1016 | 114.06622[M+H]1+, 152.02209[M+K]1+, 136.04819[M+Na]1+, 148.02829[M+Cl]1-, 112.05166[M-H]1- |
| cysteine s-sulfate | Dialister pneumosintes MGS:1496, Streptococcus oralis subsp. oralis MGS:0705, Desulfovibrio piger MGS:0955, Actinomyces sp. ICM58 MGS:0410, Fusobacterium nucleatum subsp. animalis MGS:1418, Intestinibacillus sp. Marseille-P4005 MGS:0168, Slackia isoflavoniconvertens MGS:0160, Desulfovibrionales sp. MGS:0462, Dorea formicigenerans MGS:0006, Streptococcus gordonii MGS:0713, Enterocloster clostridioformis MGS:0686, Enterocloster aldenensis MGS:0362, Scardovia wiggsiae MGS:1737, Streptococcus mutans MGS:0677, Eubacteriales sp. MGS:0128, Clostridium sp. MGS:0050, Desulfovibrio piger MGS:0240, Sutterella seckii MGS:0997, Clostridium sp. AT4 MGS:0347, Oscillospiraceae sp. MGS:0256, Gemella morbillorum MGS:1782, Eubacteriales sp. MGS:0335, Eubacteriales sp. MGS:1313, Sutterella sp. KLE1602 MGS:0228, Clostridium sp. TF06-15AC MGS:0032 | 199.96934[M-H]1- |
| cysteinylglycine disulfide* | Streptococcus oralis subsp. oralis MGS:0705, Clostridium sp. TM06-18 MGS:0048, Anaerostipes caccae MGS:0747, Eggerthella lenta MGS:0225, Actinomyces sp. ICM58 MGS:0410, Intestinibacillus sp. Marseille-P4005 MGS:0168, Slackia isoflavoniconvertens MGS:0160, Dorea formicigenerans MGS:0006, Streptococcus gordonii MGS:0713, Scardovia wiggsiae MGS:1737, Streptococcus mutans MGS:0677, Eubacteriales sp. MGS:0128, Clostridium sp. MGS:0050, Anaerobutyricum hallii MGS:0012, Ruminococcus torques MGS:0034, Clostridium sp. AT4 MGS:0347, Oscillospiraceae sp. MGS:0256, Eubacteriales sp. MGS:0335, Porphyromonas sp. MGS:1016, Dorea sp. AF36-15AT MGS:0052, Sutterella sp. KLE1602 MGS:0228, Clostridium sp. TF06-15AC MGS:0032 | 298.05258[M+H]1+, 320.03451[M+Na]1+, 296.03815[M-H]1- |
| decanoylcarnitine (C10) | Eggerthella lenta MGS:0225, Bacteroides sp. MGS:0030 | 316.24821[M+H]1+ |
| deoxycarnitine | Eubacteriales sp. MGS:1167, Eubacteriales sp. MGS:0084, Lachnospiraceae sp. MGS:0389, Bacteroides sp. MGS:0030, Oscillospiraceae sp. MGS:0074, Eubacteriales sp. MGS:0103, Eubacteriales sp. MGS:1313 | 146.11758[M+H]1+, 168.09951[M+Na]1+, 184.07337[M+K]1+ |
| deoxycholate | Clostridium sp. TM06-18 MGS:0048, Anaerostipes caccae MGS:0747, Eggerthella lenta MGS:0225, Desulfovibrio piger MGS:0955, Actinomyces sp. ICM58 MGS:0410, Fusobacterium nucleatum subsp. animalis MGS:1418, Pseudoflavonifractor sp. BIOML-A3 MGS:0735, Intestinibacillus sp. Marseille-P4005 MGS:0168, Slackia isoflavoniconvertens MGS:0160, Desulfovibrionales sp. MGS:0462, Dorea formicigenerans MGS:0006, Enterocloster clostridioformis MGS:0686, Enterocloster aldenensis MGS:0362, Megamonas funiformis MGS:0684, Streptococcus mutans MGS:0677, Eubacteriales sp. MGS:0103, Eubacteriales sp. MGS:0128, Clostridium sp. MGS:0050, Desulfovibrio piger MGS:0240, Anaerobutyricum hallii MGS:0012, Ruminococcus torques MGS:0034, Clostridium sp. AT4 MGS:0347, Oscillospiraceae sp. MGS:0256, Eubacteriales sp. MGS:0335, Porphyromonas sp. MGS:1016, Megasphaera sp. DJF_B143 MGS:1121, Dorea sp. AF36-15AT MGS:0052, Sutterella sp. KLE1602 MGS:0228, Lachnospiraceae sp. MGS:0625, Bacteria sp. MGS:0459, Clostridium sp. TF06-15AC MGS:0032 | 415.28176[M+Na]1+ |
| dihomo-linoleate (20:2n6) | Dialister pneumosintes MGS:1496, Streptococcus oralis subsp. oralis MGS:0705, Clostridium sp. TM06-18 MGS:0048, Anaerostipes caccae MGS:0747, Bacteroides ovatus MGS:0031, Lachnospiraceae sp. MGS:1271, Butyricicoccus pullicaecorum MGS:1374, Desulfovibrio piger MGS:0955, Actinomyces sp. ICM58 MGS:0410, Bacteroides sp. MGS:0030, Intestinibacillus sp. Marseille-P4005 MGS:0168, Dorea formicigenerans MGS:0006, Megamonas funiformis MGS:0684, Clostridium sp. MGS:0050, Anaerobutyricum hallii MGS:0012, Clostridium sp. AT4 MGS:0347, Eubacteriales sp. MGS:0335, Dorea sp. AF36-15AT MGS:0052, Bacteria sp. MGS:0459 | 343.24152[M+Cl]1- |
| dihomo-linolenate (20:3n3 or n6) | Streptococcus oralis subsp. oralis MGS:0705, Clostridium sp. TM06-18 MGS:0048, Anaerostipes caccae MGS:0747, Eggerthella lenta MGS:0225, Bacteroides ovatus MGS:0031, Desulfovibrio piger MGS:0955, Actinomyces sp. ICM58 MGS:0410, Bacteroides sp. MGS:0030, Fusobacterium nucleatum subsp. animalis MGS:1418, Intestinibacillus sp. Marseille-P4005 MGS:0168, Dorea formicigenerans MGS:0006, Enterocloster clostridioformis MGS:0686, Enterocloster aldenensis MGS:0362, Megamonas funiformis MGS:0684, Eubacteriales sp. MGS:0128, Clostridium sp. MGS:0050, Anaerobutyricum hallii MGS:0012, Clostridium sp. AT4 MGS:0347, Oscillospiraceae sp. MGS:0256, Eubacteriales sp. MGS:0335, Dorea sp. AF36-15AT MGS:0052, Clostridium sp. TF06-15AC MGS:0032 | 305.24902[M-H]1- |
| dimethyl sulfone | Eubacteriales sp. MGS:0085, Eubacteriales sp. MGS:0084, Fusobacterium nucleatum subsp. animalis MGS:1418, Eubacteriales sp. MGS:0260, Slackia isoflavoniconvertens MGS:0160, Scardovia wiggsiae MGS:1737, Streptococcus mutans MGS:0677, Eubacteriales sp. MGS:0103, Desulfovibrio piger MGS:0240, Clostridia sp. MGS:0845, Porphyromonas sp. MGS:1016, Lachnospiraceae sp. MGS:0625 | 132.97205[M+K]1+, 95.0161[M+H]1+, 152.975[M+NaCl+H]1+, 116.99805[M+Na]1+ |
| dodecanedioate (C12-DC) | Eubacteriales sp. MGS:1167, Eggerthella lenta MGS:0225, Bacteroides sp. MGS:0030, Pseudoflavonifractor sp. BIOML-A3 MGS:0735, Bifidobacterium catenulatum subsp. kashiwanohense MGS:0292, Scardovia wiggsiae MGS:1737, Clostridia sp. MGS:0845, Porphyromonas sp. MGS:1016, Bacteria sp. MGS:0459 | 253.14099[M+Na]1+, 269.11484[M+K]1+, 229.14459[M-H]1- |
| dodecenedioate (C12:1-DC)* | Bacteroides ovatus MGS:0031, Bacteroides sp. MGS:0030, Bifidobacterium catenulatum subsp. kashiwanohense MGS:0292, Clostridia sp. MGS:0845 | 345.06062[M+(NaCl)2+H]1+, 267.09926[M+K]1+, 246.16994[M+NH4]1+, 251.12533[M+Na]1+, 227.12889[M-H]1- |
| erythritol | Dialister pneumosintes MGS:1496, Eubacteriales sp. MGS:1167, Clostridium sp. TM06-18 MGS:0048, Anaerostipes caccae MGS:0747, Eggerthella lenta MGS:0225, Lachnospiraceae sp. MGS:1271, Butyricicoccus pullicaecorum MGS:1374, Bacteroides sp. MGS:0030, Fusobacterium nucleatum subsp. animalis MGS:1418, Intestinibacillus sp. Marseille-P4005 MGS:0168, Dorea formicigenerans MGS:0006, Lachnospiraceae sp. MGS:1209, Enterocloster clostridioformis MGS:0686, Scardovia wiggsiae MGS:1737, Streptococcus mutans MGS:0677, Anaerobutyricum hallii MGS:0012, Dorea sp. AF36-15AT MGS:0052 | 145.04716[M+Na]1+, 123.06522[M+H]1+, 161.02098[M+K]1+, 157.02726[M+Cl]1- |
| erythronate* | Eubacteriales sp. MGS:1167, Streptococcus oralis subsp. oralis MGS:0705, Clostridium sp. TM06-18 MGS:0048, Anaerostipes caccae MGS:0747, Bacteroides sp. MGS:0030, Fusobacterium nucleatum subsp. animalis MGS:1418, Dorea formicigenerans MGS:0006, Streptococcus gordonii MGS:0713, Enterocloster clostridioformis MGS:0686, Bifidobacterium catenulatum subsp. kashiwanohense MGS:0292, Enterocloster aldenensis MGS:0362, Scardovia wiggsiae MGS:1737, Streptococcus mutans MGS:0677, Anaerobutyricum hallii MGS:0012, Longibaculum muris MGS:1605, Dorea sp. AF36-15AT MGS:0052 | 195.00309[M+NaCl+H]1+, 192.98853[M+NaCl-H]1-, 135.02995[M-H]1- |
| ethyl alpha-glucopyranoside | Dialister pneumosintes MGS:1496, Streptococcus oralis subsp. oralis MGS:0705, Clostridium sp. TM06-18 MGS:0048, Anaerostipes caccae MGS:0747, Lachnospiraceae sp. MGS:1271, Butyricicoccus pullicaecorum MGS:1374, Desulfovibrio piger MGS:0955, Actinomyces sp. ICM58 MGS:0410, Bacteroides sp. MGS:0030, Intestinibacillus sp. Marseille-P4005 MGS:0168, Slackia isoflavoniconvertens MGS:0160, Dorea formicigenerans MGS:0006, Eubacteriales sp. MGS:0128, Clostridium sp. MGS:0050, Ruminococcus torques MGS:0034, Parabacteroides johnsonii MGS:0469, Clostridium sp. AT4 MGS:0347, Oscillospiraceae sp. MGS:0256, Eubacteriales sp. MGS:0335, Dorea sp. AF36-15AT MGS:0052, Sutterella sp. KLE1602 MGS:0228, Bacteria sp. MGS:0459 | 231.0839[M+Na]1+, 247.05784[M+K]1+, 226.12838[M+NH4]1+, 208.09445[M1+.]1+, 253.09304[M-H+FA]1-, 207.08749[M-H]1- |
| ethyl beta-glucopyranoside | Clostridium sp. TM06-18 MGS:0048, Bacteroides ovatus MGS:0031, Butyricicoccus pullicaecorum MGS:1374, Bacteroides sp. MGS:0030, Lachnospiraceae sp. MGS:1209, Parabacteroides johnsonii MGS:0469, Bacteria sp. MGS:0459 | 231.0839[M+Na]1+, 247.05784[M+K]1+, 226.12838[M+NH4]1+, 208.09445[M1+.]1+, 253.09304[M-H+FA]1-, 207.08749[M-H]1- |
| ethyl glucuronide | Clostridium sp. TM06-18 MGS:0048, Anaerostipes caccae MGS:0747, Eggerthella lenta MGS:0225, Lachnospiraceae sp. MGS:1271, Butyricicoccus pullicaecorum MGS:1374, Desulfovibrio piger MGS:0955, Actinomyces sp. ICM58 MGS:0410, Bacteroides sp. MGS:0030, Intestinibacillus sp. Marseille-P4005 MGS:0168, Slackia isoflavoniconvertens MGS:0160, Dorea formicigenerans MGS:0006, Eubacteriales sp. MGS:0128, Clostridium sp. MGS:0050, Desulfovibrio piger MGS:0240, Ruminococcus torques MGS:0034, Gemmiger sp. MGS:0862, Parabacteroides johnsonii MGS:0469, Clostridium sp. AT4 MGS:0347, Oscillospiraceae sp. MGS:0256, Eubacteriales sp. MGS:0335, Dorea sp. AF36-15AT MGS:0052, Sutterella sp. KLE1602 MGS:0228, Bacteria sp. MGS:0459 | 222.07354[M1+.]1+, 245.06319[M+Na]1+, 221.0666[M-H]1-, 267.07228[M-H+FA]1- |
| fumarate | Streptococcus oralis subsp. oralis MGS:0705, Anaerostipes caccae MGS:0747, Bacteroides ovatus MGS:0031, Desulfovibrio piger MGS:0955, Actinomyces sp. ICM58 MGS:0410, Bacteroides sp. MGS:0030, Fusobacterium nucleatum subsp. animalis MGS:1418, Enterocloster clostridioformis MGS:0686, Enterocloster aldenensis MGS:0362, Longibaculum muris MGS:1605, Lachnospiraceae sp. MGS:0625, Bacteria sp. MGS:0459 | 134.0448[M+NH4]1+, 232.9354[M+(NaCl)2+H]1+, 115.00368[M-H]1-, 161.00918[M-H+FA]1- |
| galactonate | Anaerostipes caccae MGS:0747, Streptococcus gordonii MGS:0713, Lachnospiraceae sp. MGS:1209, Bifidobacterium catenulatum subsp. kashiwanohense MGS:0292, Scardovia wiggsiae MGS:1737, Streptococcus mutans MGS:0677, Saccharomyces cerevisiae MGS:0782 | 219.04749[M+Na]1+, 196.0581[M1+.]1+, 241.0565[M-H+FA]1-, 195.05102[M-H]1- |
| gamma-glutamylglutamine | Eubacteriales sp. MGS:1167, Eubacteriales sp. MGS:0085, Bacteria sp. MGS:0575, Lachnospiraceae sp. MGS:0389, Lachnospiraceae sp. MGS:1209, Ruminococcus callidus MGS:0247, Eubacteriales sp. MGS:0103, Eubacteriales sp. MGS:0671, Clostridia sp. MGS:0845 | 298.10095[M+Na]1+, 276.11906[M+H]1+, 274.10463[M-H]1- |
| gamma-glutamylvaline | Streptococcus oralis subsp. oralis MGS:0705, Clostridium sp. TM06-18 MGS:0048, Eggerthella lenta MGS:0225, Lachnospiraceae sp. MGS:0389, Intestinibacillus sp. Marseille-P4005 MGS:0168, Dorea formicigenerans MGS:0006, Enterocloster clostridioformis MGS:0686, Enterocloster aldenensis MGS:0362, Ruminococcus callidus MGS:0247, Scardovia wiggsiae MGS:1737, Streptococcus mutans MGS:0677, Eubacteriales sp. MGS:0128, Clostridium sp. MGS:0050, Anaerobutyricum hallii MGS:0012, Ruminococcus torques MGS:0034, Clostridium sp. AT4 MGS:0347, Oscillospiraceae sp. MGS:0256, Gemella morbillorum MGS:1782, Dorea sp. AF36-15AT MGS:0052, Sutterella sp. KLE1602 MGS:0228, Clostridium sp. TF06-15AC MGS:0032 | 269.11073[M+Na]1+ |
| gentisate | Eubacteriales sp. MGS:0085, Eubacteriales sp. MGS:0084, Lachnospiraceae sp. MGS:0389, Eubacteriales sp. MGS:0260, Lachnospiraceae sp. MGS:1209, Gemmiger sp. MGS:0862, Eubacteriales sp. MGS:0671, Clostridia sp. MGS:0845, Clostridia sp. MGS:1035 | 153.01931[M-H]1- |
| gluconate | Eggerthella lenta MGS:0225, Eubacteriales sp. MGS:0260, Enterocloster clostridioformis MGS:0686, Bifidobacterium catenulatum subsp. kashiwanohense MGS:0292, Enterocloster aldenensis MGS:0362, Scardovia wiggsiae MGS:1737, Oscillospiraceae sp. MGS:0074, Eubacteriales sp. MGS:0103, Eubacteriales sp. MGS:1313 | 219.04749[M+Na]1+, 196.0581[M1+.]1+, 241.0565[M-H+FA]1-, 195.05102[M-H]1- |
| glucuronate | Streptococcus oralis subsp. oralis MGS:0705, Anaerostipes caccae MGS:0747, Eggerthella lenta MGS:0225, Butyricicoccus pullicaecorum MGS:1374, Fusobacterium nucleatum subsp. animalis MGS:1418, Enterocloster clostridioformis MGS:0686, Enterocloster aldenensis MGS:0362, Scardovia wiggsiae MGS:1737, Streptococcus mutans MGS:0677, Parabacteroides johnsonii MGS:0469, Oscillospiraceae sp. MGS:0256 | 217.03196[M+Na]1+, 212.07639[M+NH4]1+, 193.03537[M-H]1-, 229.012[M+Cl]1- |
| glutamine | Eubacteriales sp. MGS:1167, Eubacteriales sp. MGS:0085, Lachnospiraceae sp. MGS:0389, Eubacteriales sp. MGS:0260, Bifidobacterium catenulatum subsp. kashiwanohense MGS:0292, Ruminococcus callidus MGS:0247, Eubacteriales sp. MGS:0103, Eubacteriales sp. MGS:0671, Clostridia sp. MGS:0845, Clostridia sp. MGS:1035, Eubacteriales sp. MGS:1313 | 169.05837[M+Na]1+, 147.07644[M+H]1+, 185.03233[M+K]1+, 376.89704[M+(NaCl)4-H]1-, 434.85492[M+(NaCl)5-H]1-, 181.03859[M+Cl]1-, 203.02058[M+NaCl-H]1-, 145.06183[M-H]1-, 318.9382[M+(NaCl)3-H]1- |
| glutarylcarnitine (C5-DC) | Bacteroides ovatus MGS:0031, Lachnospiraceae sp. MGS:1209, Porphyromonas sp. MGS:1016 | 276.14418[M1+.]1+, 335.11021[M+NaCl+H]1+ |
| glycerol | Dialister pneumosintes MGS:1496, Streptococcus oralis subsp. oralis MGS:0705, Clostridium sp. TM06-18 MGS:0048, Anaerostipes caccae MGS:0747, Eggerthella lenta MGS:0225, Lachnospiraceae sp. MGS:1271, Butyricicoccus pullicaecorum MGS:1374, Desulfovibrio piger MGS:0955, Actinomyces sp. ICM58 MGS:0410, Fusobacterium nucleatum subsp. animalis MGS:1418, Intestinibacillus sp. Marseille-P4005 MGS:0168, Dorea formicigenerans MGS:0006, Enterocloster clostridioformis MGS:0686, Enterocloster aldenensis MGS:0362, Scardovia wiggsiae MGS:1737, Megamonas funiformis MGS:0684, Eubacteriales sp. MGS:0128, Clostridium sp. MGS:0050, Desulfovibrio piger MGS:0240, Anaerobutyricum hallii MGS:0012, Ruminococcus torques MGS:0034, Clostridium sp. AT4 MGS:0347, Oscillospiraceae sp. MGS:0256, Gemella morbillorum MGS:1782, Eubacteriales sp. MGS:0335, Dorea sp. AF36-15AT MGS:0052, Sutterella sp. KLE1602 MGS:0228, Bacteria sp. MGS:0459, Clostridium sp. TF06-15AC MGS:0032 | 93.05464[M+H]1+, 131.01052[M+K]1+, 115.03658[M+Na]1+, 151.0131[M+NaCl+H]1+, 137.04554[M-H+FA]1-, 127.01679[M+Cl]1- |
| glycocholate | Dialister pneumosintes MGS:1496, Streptococcus oralis subsp. oralis MGS:0705, Bacteroides sp. MGS:0030, Fusobacterium nucleatum subsp. animalis MGS:1418, Streptococcus gordonii MGS:0713, Scardovia wiggsiae MGS:1737, Oscillospiraceae sp. MGS:0074, Streptococcus mutans MGS:0677, Eubacteriales sp. MGS:0671 | 524.2743[M+NaCl+H]1+, 500.2782[M+Cl]1- |
| glycohyocholate | Lachnospiraceae sp. MGS:1271, Bacteroides sp. MGS:0030, Fusobacterium nucleatum subsp. animalis MGS:1418, Lachnospiraceae sp. MGS:1209, Oscillospiraceae sp. MGS:0074, Parabacteroides johnsonii MGS:0469, Eubacteriales sp. MGS:0671 | 524.2743[M+NaCl+H]1+, 500.2782[M+Cl]1- |
| guanidinoacetate | Bacteria sp. MGS:0459 | 140.04307[M+Na]1+, 118.06109[M+H]1+ |
| gulonate* | Streptococcus oralis subsp. oralis MGS:0705, Anaerostipes caccae MGS:0747, Eggerthella lenta MGS:0225, Intestinibacillus sp. Marseille-P4005 MGS:0168, Streptococcus gordonii MGS:0713, Bifidobacterium catenulatum subsp. kashiwanohense MGS:0292, Scardovia wiggsiae MGS:1737, Streptococcus mutans MGS:0677, Eubacteriales sp. MGS:0103, Anaerobutyricum hallii MGS:0012, Oscillospiraceae sp. MGS:0256, Saccharomyces cerevisiae MGS:0782 | 219.04749[M+Na]1+, 196.0581[M1+.]1+, 241.0565[M-H+FA]1-, 195.05102[M-H]1- |
| hexanoylcarnitine (C6) | Streptococcus oralis subsp. oralis MGS:0705, Clostridium sp. TM06-18 MGS:0048, Anaerostipes caccae MGS:0747, Eggerthella lenta MGS:0225, Desulfovibrio piger MGS:0955, Actinomyces sp. ICM58 MGS:0410, Bacteroides sp. MGS:0030, Fusobacterium nucleatum subsp. animalis MGS:1418, Intestinibacillus sp. Marseille-P4005 MGS:0168, Desulfovibrionales sp. MGS:0462, Dorea formicigenerans MGS:0006, Eubacteriales sp. MGS:0128, Clostridium sp. MGS:0050, Desulfovibrio piger MGS:0240, Anaerobutyricum hallii MGS:0012, Ruminococcus torques MGS:0034, Clostridium sp. AT4 MGS:0347, Oscillospiraceae sp. MGS:0256, Dorea sp. AF36-15AT MGS:0052, Sutterella sp. KLE1602 MGS:0228, Clostridium sp. TF06-15AC MGS:0032 | 260.18564[M1+.]1+ |
| hippurate | Eubacteriales sp. MGS:0085, Bacteria sp. MGS:0575, Eubacteriales sp. MGS:0084, Lachnospiraceae sp. MGS:0389, Eubacteriales sp. MGS:0260, Lachnospiraceae sp. MGS:1209, Ruminococcus callidus MGS:0247, Oscillospiraceae sp. MGS:0074, Eubacteriales sp. MGS:0103, Gemmiger sp. MGS:0862, Eubacteriales sp. MGS:0671, Clostridia sp. MGS:0845, Clostridia sp. MGS:1035, Eubacteriales sp. MGS:1313 | 202.0476[M+Na]1+, 178.05097[M-H]1- |
| histidine | Eubacteriales sp. MGS:1167, Bacteria sp. MGS:0575, Ruminococcus callidus MGS:0247, Eubacteriales sp. MGS:0103, Sutterella seckii MGS:0997, Eubacteriales sp. MGS:0671, Clostridia sp. MGS:0845, Clostridia sp. MGS:1035, Eubacteriales sp. MGS:1313 | 173.10342[M+NH4]1+, 156.07679[M+H]1+, 178.05867[M+Na]1+, 194.03269[M+K]1+, 190.03891[M+Cl]1-, 212.02085[M+NaCl-H]1-, 154.0622[M-H]1- |
| homostachydrine* | Eubacteriales sp. MGS:1167, Bacteria sp. MGS:0575, Eubacteriales sp. MGS:0084, Lachnospiraceae sp. MGS:0389, Eubacteriales sp. MGS:0260, Slackia isoflavoniconvertens MGS:0160, Lachnospiraceae sp. MGS:1209, Parabacteroides johnsonii MGS:0469, Eubacteriales sp. MGS:0671, Clostridia sp. MGS:0845, Clostridia sp. MGS:1035 | 175.14409[M+NH4]1+, 158.11757[M+H]1+, 180.09947[M+Na]1+ |
| homovanillate (HVA) | Clostridium sp. TM06-18 MGS:0048, Eggerthella lenta MGS:0225, Bacteroides ovatus MGS:0031, Bacteroides sp. MGS:0030, Lachnospiraceae sp. MGS:1209, Bifidobacterium catenulatum subsp. kashiwanohense MGS:0292, Eubacteriales sp. MGS:0128, Clostridium sp. MGS:0050, Bacteria sp. MGS:0459, Clostridium sp. TF06-15AC MGS:0032 | 205.04725[M+Na]1+, 181.05069[M-H]1- |
| hypoxanthine | Clostridium sp. TM06-18 MGS:0048, Fusobacterium nucleatum subsp. animalis MGS:1418, Dorea formicigenerans MGS:0006, Streptococcus mutans MGS:0677, Clostridium sp. MGS:0050, Anaerobutyricum hallii MGS:0012, Ruminococcus torques MGS:0034, Gemmiger sp. MGS:0862, Clostridium sp. AT4 MGS:0347, Clostridium sp. TF06-15AC MGS:0032 | 175.00169[M+K]1+, 159.02781[M+Na]1+ |
| ibuprofen | Eggerthella lenta MGS:0225, Butyricicoccus pullicaecorum MGS:1374, Intestinibacillus sp. Marseille-P4005 MGS:0168, Streptococcus mutans MGS:0677, Parabacteroides johnsonii MGS:0469, Oscillospiraceae sp. MGS:0256 | 206.12981[M1+.]1+, 229.1201[M+Na]1+, 438.97168[M+(NaCl)4+H]1+, 381.01309[M+(NaCl)3+H]1+ |
| ibuprofen acyl glucuronide | Butyricicoccus pullicaecorum MGS:1374, Intestinibacillus sp. Marseille-P4005 MGS:0168, Enterocloster clostridioformis MGS:0686, Enterocloster aldenensis MGS:0362, Streptococcus mutans MGS:0677, Parabacteroides johnsonii MGS:0469, Oscillospiraceae sp. MGS:0256 | 383.16979[M+H]1+ |
| imidazole lactate | Bacteroides ovatus MGS:0031, Butyricicoccus pullicaecorum MGS:1374, Bacteroides sp. MGS:0030, Lachnospiraceae sp. MGS:1209, Bacteria sp. MGS:0459 | 157.06086[M+H]1+, 179.04265[M+Na]1+, 155.04625[M-H]1- |
| indole-3-carboxylate | Eubacteriales sp. MGS:1167, Bacteria sp. MGS:0575, Pseudoflavonifractor sp. BIOML-A3 MGS:0735, Eubacteriales sp. MGS:0260, Slackia isoflavoniconvertens MGS:0160, Desulfovibrionales sp. MGS:0462, Eubacteriales sp. MGS:0103, Desulfovibrio piger MGS:0240, Sutterella seckii MGS:0997, Gemmiger sp. MGS:0862, Eubacteriales sp. MGS:0671, Clostridia sp. MGS:0845, Saccharomyces cerevisiae MGS:0782, Porphyromonas sp. MGS:1016, Eubacteriales sp. MGS:1313, Megasphaera sp. DJF_B143 MGS:1121, Lachnospiraceae sp. MGS:0625 | 184.03708[M+Na]1+ |
| indolelactate | Eubacteriales sp. MGS:1167, Clostridium sp. TM06-18 MGS:0048, Bacteroides ovatus MGS:0031, Bacteroides sp. MGS:0030, Lachnospiraceae sp. MGS:1209, Longibaculum muris MGS:1605 | 228.06314[M+Na]1+, 204.06658[M-H]1- |
| isobutyrylcarnitine (C4) | Dialister pneumosintes MGS:1496, Eubacteriales sp. MGS:1167, Eubacteriales sp. MGS:0085, Eubacteriales sp. MGS:0084, Lachnospiraceae sp. MGS:0389, Pseudoflavonifractor sp. BIOML-A3 MGS:0735, Eubacteriales sp. MGS:0260, Eubacteriales sp. MGS:0103, Eubacteriales sp. MGS:0671, Clostridia sp. MGS:0845, Clostridia sp. MGS:1035, Eubacteriales sp. MGS:1313 | 232.15431[M1+.]1+, 291.12028[M+NaCl+H]1+, 267.1239[M+Cl]1- |
| isobutyrylglycine | Eubacteriales sp. MGS:1167, Eubacteriales sp. MGS:0085, Eubacteriales sp. MGS:0084, Pseudoflavonifractor sp. BIOML-A3 MGS:0735, Megamonas funiformis MGS:0684, Longibaculum muris MGS:1605, Bacteria sp. MGS:0459 | 146.08117[M+H]1+, 168.06313[M+Na]1+, 184.03708[M+K]1+, 144.06662[M-H]1- |
| isoursodeoxycholate | Streptococcus oralis subsp. oralis MGS:0705, Clostridium sp. TM06-18 MGS:0048, Anaerostipes caccae MGS:0747, Eggerthella lenta MGS:0225, Bacteroides ovatus MGS:0031, Lachnospiraceae sp. MGS:1271, Butyricicoccus pullicaecorum MGS:1374, Actinomyces sp. ICM58 MGS:0410, Bacteroides sp. MGS:0030, Fusobacterium nucleatum subsp. animalis MGS:1418, Intestinibacillus sp. Marseille-P4005 MGS:0168, Dorea formicigenerans MGS:0006, Enterocloster clostridioformis MGS:0686, Enterocloster aldenensis MGS:0362, Megamonas funiformis MGS:0684, Eubacteriales sp. MGS:0128, Clostridium sp. MGS:0050, Anaerobutyricum hallii MGS:0012, Ruminococcus torques MGS:0034, Longibaculum muris MGS:1605, Clostridium sp. AT4 MGS:0347, Oscillospiraceae sp. MGS:0256, Gemella morbillorum MGS:1782, Saccharomyces cerevisiae MGS:0782, Eubacteriales sp. MGS:0335, Dorea sp. AF36-15AT MGS:0052, Sutterella sp. KLE1602 MGS:0228, Lachnospiraceae sp. MGS:0625, Bacteria sp. MGS:0459, Clostridium sp. TF06-15AC MGS:0032 | 415.28176[M+Na]1+ |
| kynurenine | Clostridium sp. TM06-18 MGS:0048, Eubacteriales sp. MGS:0085, Eubacteriales sp. MGS:0084, Dorea formicigenerans MGS:0006, Streptococcus gordonii MGS:0713, Lachnospiraceae sp. MGS:1209, Bifidobacterium catenulatum subsp. kashiwanohense MGS:0292, Ruminococcus callidus MGS:0247, Scardovia wiggsiae MGS:1737, Oscillospiraceae sp. MGS:0074, Streptococcus mutans MGS:0677, Eubacteriales sp. MGS:0103, Eubacteriales sp. MGS:0128, Clostridium sp. MGS:0050, Anaerobutyricum hallii MGS:0012, Ruminococcus torques MGS:0034, Clostridia sp. MGS:1035, Gemella morbillorum MGS:1782, Porphyromonas sp. MGS:1016, Clostridium sp. TF06-15AC MGS:0032 | 231.07398[M+Na]1+ |
| lactate | Streptococcus oralis subsp. oralis MGS:0705, Clostridium sp. TM06-18 MGS:0048, Anaerostipes caccae MGS:0747, Eggerthella lenta MGS:0225, Lachnospiraceae sp. MGS:1271, Desulfovibrio piger MGS:0955, Actinomyces sp. ICM58 MGS:0410, Bacteroides sp. MGS:0030, Fusobacterium nucleatum subsp. animalis MGS:1418, Intestinibacillus sp. Marseille-P4005 MGS:0168, Dorea formicigenerans MGS:0006, Enterocloster clostridioformis MGS:0686, Enterocloster aldenensis MGS:0362, Scardovia wiggsiae MGS:1737, Megamonas funiformis MGS:0684, Streptococcus mutans MGS:0677, Eubacteriales sp. MGS:0128, Clostridium sp. MGS:0050, Desulfovibrio piger MGS:0240, Anaerobutyricum hallii MGS:0012, Ruminococcus torques MGS:0034, Sutterella seckii MGS:0997, Longibaculum muris MGS:1605, Clostridium sp. AT4 MGS:0347, Oscillospiraceae sp. MGS:0256, Gemella morbillorum MGS:1782, Eubacteriales sp. MGS:0335, Dorea sp. AF36-15AT MGS:0052, Sutterella sp. KLE1602 MGS:0228, Bacteria sp. MGS:0459, Clostridium sp. TF06-15AC MGS:0032 | 128.99486[M+K]1+, 91.03893[M+H]1+, 113.02087[M+Na]1+, 125.00114[M+Cl]1-, 146.98312[M+NaCl-H]1-, 89.0245[M-H]1-, 204.94161[M+(NaCl)2-H]1-, 320.8594[M+(NaCl)4-H]1-, 378.81825[M+(NaCl)5-H]1-, 135.02995[M-H+FA]1-, 262.90035[M+(NaCl)3-H]1- |
| lidocaine | Streptococcus oralis subsp. oralis MGS:0705, Lachnospiraceae sp. MGS:0389, Intestinibacillus sp. Marseille-P4005 MGS:0168, Dorea formicigenerans MGS:0006, Streptococcus gordonii MGS:0713, Anaerobutyricum hallii MGS:0012, Dorea sp. AF36-15AT MGS:0052 | 409.05707[M+(NaCl)3+H]1+ |
| linoleoylcarnitine (C18:2)* | Bacteroides ovatus MGS:0031, Eubacteriales sp. MGS:0085, Eubacteriales sp. MGS:0084, Lachnospiraceae sp. MGS:0389, Eubacteriales sp. MGS:0260, Lachnospiraceae sp. MGS:1209, Gemmiger sp. MGS:0862, Eubacteriales sp. MGS:0671, Clostridia sp. MGS:0845 | 424.34204[M1+.]1+ |
| maleate | Streptococcus oralis subsp. oralis MGS:0705, Anaerostipes caccae MGS:0747, Eggerthella lenta MGS:0225, Butyricicoccus pullicaecorum MGS:1374, Fusobacterium nucleatum subsp. animalis MGS:1418, Intestinibacillus sp. Marseille-P4005 MGS:0168, Streptococcus gordonii MGS:0713, Enterocloster clostridioformis MGS:0686, Enterocloster aldenensis MGS:0362, Scardovia wiggsiae MGS:1737, Streptococcus mutans MGS:0677, Clostridium sp. AT4 MGS:0347, Oscillospiraceae sp. MGS:0256 | 134.0448[M+NH4]1+, 232.9354[M+(NaCl)2+H]1+, 115.00368[M-H]1-, 161.00918[M-H+FA]1- |
| methionine | Eubacteriales sp. MGS:1167, Eubacteriales sp. MGS:0085, Lachnospiraceae sp. MGS:0389, Slackia isoflavoniconvertens MGS:0160, Streptococcus gordonii MGS:0713, Bifidobacterium catenulatum subsp. kashiwanohense MGS:0292, Ruminococcus callidus MGS:0247, Eubacteriales sp. MGS:0103, Eubacteriales sp. MGS:0671, Clostridia sp. MGS:1035, Eubacteriales sp. MGS:1313 | 172.04022[M+Na]1+, 150.05837[M+H]1+, 188.01428[M+K]1+, 184.02024[M+Cl]1-, 148.04382[M-H]1-, 321.91935[M+(NaCl)3-H]1- |
| methyl glucopyranoside (alpha + beta) | Bacteroides ovatus MGS:0031, Eubacteriales sp. MGS:0085, Eubacteriales sp. MGS:0084, Butyricicoccus pullicaecorum MGS:1374, Lachnospiraceae sp. MGS:1209, Eubacteriales sp. MGS:0671, Clostridia sp. MGS:1035, Bacteria sp. MGS:0459 | 194.07886[M1+.]1+, 229.0483[M+Cl]1-, 239.07737[M-H+FA]1-, 193.07173[M-H]1- |
| myristate (14:0) | Dialister pneumosintes MGS:1496, Streptococcus oralis subsp. oralis MGS:0705, Clostridium sp. TM06-18 MGS:0048, Eggerthella lenta MGS:0225, Butyricicoccus pullicaecorum MGS:1374, Fusobacterium nucleatum subsp. animalis MGS:1418, Intestinibacillus sp. Marseille-P4005 MGS:0168, Dorea formicigenerans MGS:0006, Anaerobutyricum hallii MGS:0012, Ruminococcus torques MGS:0034, Clostridium sp. AT4 MGS:0347, Gemella morbillorum MGS:1782, Saccharomyces cerevisiae MGS:0782, Dorea sp. AF36-15AT MGS:0052 | 287.17478[M+NaCl+H]1+, 251.19805[M+Na]1+, 267.17192[M+K]1+, 273.20725[M-H+FA]1-, 263.17842[M+Cl]1-, 227.20173[M-H]1- |
| myristoleate (14:1n5) | Dialister pneumosintes MGS:1496, Streptococcus oralis subsp. oralis MGS:0705, Clostridium sp. TM06-18 MGS:0048, Anaerostipes caccae MGS:0747, Eggerthella lenta MGS:0225, Butyricicoccus pullicaecorum MGS:1374, Desulfovibrio piger MGS:0955, Actinomyces sp. ICM58 MGS:0410, Fusobacterium nucleatum subsp. animalis MGS:1418, Intestinibacillus sp. Marseille-P4005 MGS:0168, Desulfovibrionales sp. MGS:0462, Dorea formicigenerans MGS:0006, Bifidobacterium catenulatum subsp. kashiwanohense MGS:0292, Scardovia wiggsiae MGS:1737, Desulfovibrio piger MGS:0240, Ruminococcus torques MGS:0034, Clostridium sp. AT4 MGS:0347, Gemella morbillorum MGS:1782, Saccharomyces cerevisiae MGS:0782, Dorea sp. AF36-15AT MGS:0052, Sutterella sp. KLE1602 MGS:0228, Bacteria sp. MGS:0459 | 265.15633[M+K]1+, 227.20052[M+H]1+, 249.18245[M+Na]1+, 225.18604[M-H]1- |
| myristoleoylcarnitine (C14:1)* | Anaerostipes caccae MGS:0747, Bacteroides sp. MGS:0030 | 370.29509[M1+.]1+ |
| octanoylcarnitine (C8) | Eggerthella lenta MGS:0225, Bacteroides sp. MGS:0030 | 288.21687[M1+.]1+ |
| oleoylcarnitine (C18:1) | Dialister pneumosintes MGS:1496, Streptococcus oralis subsp. oralis MGS:0705, Clostridium sp. TM06-18 MGS:0048, Lachnospiraceae sp. MGS:1271, Desulfovibrio piger MGS:0955, Actinomyces sp. ICM58 MGS:0410, Bacteroides sp. MGS:0030, Fusobacterium nucleatum subsp. animalis MGS:1418, Intestinibacillus sp. Marseille-P4005 MGS:0168, Enterocloster aldenensis MGS:0362, Megamonas funiformis MGS:0684, Eubacteriales sp. MGS:0128, Clostridium sp. MGS:0050, Desulfovibrio piger MGS:0240, Ruminococcus torques MGS:0034, Parabacteroides johnsonii MGS:0469, Clostridium sp. AT4 MGS:0347, Eubacteriales sp. MGS:0335, Sutterella sp. KLE1602 MGS:0228 | 426.35765[M1+.]1+ |
| ornithine | Lachnospiraceae sp. MGS:0389, Streptococcus mutans MGS:0677, Eubacteriales sp. MGS:1313 | 171.05306[M+K]1+, 249.01483[M+(NaCl)2+H]1+, 133.09717[M+H]1+, 155.07905[M+Na]1+, 131.08261[M-H]1-, 167.05927[M+Cl]1- |
| palmitoleate (16:1n7) | Dialister pneumosintes MGS:1496, Streptococcus oralis subsp. oralis MGS:0705, Clostridium sp. TM06-18 MGS:0048, Anaerostipes caccae MGS:0747, Eggerthella lenta MGS:0225, Lachnospiraceae sp. MGS:1271, Butyricicoccus pullicaecorum MGS:1374, Desulfovibrio piger MGS:0955, Actinomyces sp. ICM58 MGS:0410, Bacteroides sp. MGS:0030, Fusobacterium nucleatum subsp. animalis MGS:1418, Intestinibacillus sp. Marseille-P4005 MGS:0168, Desulfovibrionales sp. MGS:0462, Dorea formicigenerans MGS:0006, Streptococcus gordonii MGS:0713, Enterocloster clostridioformis MGS:0686, Enterocloster aldenensis MGS:0362, Scardovia wiggsiae MGS:1737, Megamonas funiformis MGS:0684, Eubacteriales sp. MGS:0128, Clostridium sp. MGS:0050, Desulfovibrio piger MGS:0240, Anaerobutyricum hallii MGS:0012, Ruminococcus torques MGS:0034, Clostridium sp. AT4 MGS:0347, Oscillospiraceae sp. MGS:0256, Gemella morbillorum MGS:1782, Saccharomyces cerevisiae MGS:0782, Eubacteriales sp. MGS:0335, Dorea sp. AF36-15AT MGS:0052, Sutterella sp. KLE1602 MGS:0228, Bacteria sp. MGS:0459 | 293.18759[M+K]1+, 277.21377[M+Na]1+, 255.23138[M+H]1+, 289.19433[M+Cl]1-, 253.21743[M-H]1-, 299.22298[M-H+FA]1- |
| palmitoylcarnitine (C16) | Streptococcus oralis subsp. oralis MGS:0705, Clostridium sp. TM06-18 MGS:0048, Desulfovibrio piger MGS:0955, Actinomyces sp. ICM58 MGS:0410, Fusobacterium nucleatum subsp. animalis MGS:1418, Intestinibacillus sp. Marseille-P4005 MGS:0168, Desulfovibrionales sp. MGS:0462, Dorea formicigenerans MGS:0006, Enterocloster aldenensis MGS:0362, Megamonas funiformis MGS:0684, Eubacteriales sp. MGS:0128, Clostridium sp. MGS:0050, Desulfovibrio piger MGS:0240, Anaerobutyricum hallii MGS:0012, Ruminococcus torques MGS:0034, Parabacteroides johnsonii MGS:0469, Longibaculum muris MGS:1605, Clostridium sp. AT4 MGS:0347, Oscillospiraceae sp. MGS:0256, Gemella morbillorum MGS:1782, Dorea sp. AF36-15AT MGS:0052, Sutterella sp. KLE1602 MGS:0228 | 400.34211[M1+.]1+ |
| phenylacetate | Dialister pneumosintes MGS:1496, Eubacteriales sp. MGS:1167, Eubacteriales sp. MGS:0085, Eubacteriales sp. MGS:0084, Desulfovibrio piger MGS:0955, Fusobacterium nucleatum subsp. animalis MGS:1418, Pseudoflavonifractor sp. BIOML-A3 MGS:0735, Eubacteriales sp. MGS:0260, Desulfovibrionales sp. MGS:0462, Enterocloster clostridioformis MGS:0686, Enterocloster aldenensis MGS:0362, Ruminococcus callidus MGS:0247, Streptococcus mutans MGS:0677, Eubacteriales sp. MGS:0103, Desulfovibrio piger MGS:0240, Eubacteriales sp. MGS:0671, Clostridia sp. MGS:0845, Clostridia sp. MGS:1035, Porphyromonas sp. MGS:1016, Eubacteriales sp. MGS:1313, Lachnospiraceae sp. MGS:0625 | 159.04174[M+Na]1+, 181.05069[M-H+FA]1-, 135.04523[M-H]1- |
| phenylalanine | Streptococcus oralis subsp. oralis MGS:0705, Eubacteriales sp. MGS:0085, Eubacteriales sp. MGS:0084, Lachnospiraceae sp. MGS:0389, Actinomyces sp. ICM58 MGS:0410, Dorea formicigenerans MGS:0006, Streptococcus gordonii MGS:0713, Bifidobacterium catenulatum subsp. kashiwanohense MGS:0292, Ruminococcus callidus MGS:0247, Scardovia wiggsiae MGS:1737, Oscillospiraceae sp. MGS:0074, Streptococcus mutans MGS:0677, Clostridium sp. MGS:0050, Anaerobutyricum hallii MGS:0012, Ruminococcus torques MGS:0034, Eubacteriales sp. MGS:0671, Gemella morbillorum MGS:1782, Sutterella sp. KLE1602 MGS:0228, Clostridium sp. TF06-15AC MGS:0032 | 204.04223[M+K]1+, 188.06823[M+Na]1+, 166.08624[M+H]1+, 164.0716[M-H]1-, 200.04843[M+Cl]1-, 279.98909[M+(NaCl)2-H]1-, 222.03025[M+NaCl-H]1- |
| phenylpyruvate | Eubacteriales sp. MGS:1167, Eubacteriales sp. MGS:0085, Lachnospiraceae sp. MGS:0389, Lachnospiraceae sp. MGS:1209, Clostridium sp. MGS:0050, Clostridium sp. TF06-15AC MGS:0032 | 182.08121[M+NH4]1+, 165.05462[M+H]1+, 203.01014[M+K]1+, 187.03667[M+Na]1+, 163.04012[M-H]1- |
| picolinate | Lachnospiraceae sp. MGS:0389, Eubacteriales sp. MGS:0260, Ruminococcus callidus MGS:0247, Eubacteriales sp. MGS:0671 | 122.0248[M-H]1-, 168.03019[M-H+FA]1- |
| pro-hydroxy-pro | Dialister pneumosintes MGS:1496, Eubacteriales sp. MGS:0260, Oscillospiraceae sp. MGS:0074, Streptococcus mutans MGS:0677, Eubacteriales sp. MGS:0103, Sutterella seckii MGS:0997, Eubacteriales sp. MGS:1313 | 251.10023[M+Na]1+ |
| propionylcarnitine (C3) | Dialister pneumosintes MGS:1496, Eubacteriales sp. MGS:1167, Streptococcus oralis subsp. oralis MGS:0705, Clostridium sp. TM06-18 MGS:0048, Eubacteriales sp. MGS:0085, Bacteria sp. MGS:0575, Eubacteriales sp. MGS:0084, Lachnospiraceae sp. MGS:0389, Desulfovibrio piger MGS:0955, Fusobacterium nucleatum subsp. animalis MGS:1418, Eubacteriales sp. MGS:0260, Slackia isoflavoniconvertens MGS:0160, Desulfovibrionales sp. MGS:0462, Dorea formicigenerans MGS:0006, Ruminococcus callidus MGS:0247, Scardovia wiggsiae MGS:1737, Megamonas funiformis MGS:0684, Oscillospiraceae sp. MGS:0074, Streptococcus mutans MGS:0677, Clostridium sp. MGS:0050, Desulfovibrio piger MGS:0240, Ruminococcus torques MGS:0034, Sutterella seckii MGS:0997, Gemmiger sp. MGS:0862, Clostridia sp. MGS:0845, Clostridia sp. MGS:1035, Eubacteriales sp. MGS:1313, Megasphaera sp. DJF_B143 MGS:1121, Sutterella sp. KLE1602 MGS:0228, Clostridium sp. TF06-15AC MGS:0032 | 218.13868[M1+.]1+, 277.10478[M+NaCl+H]1+ |
| propionylglycine | Eubacteriales sp. MGS:1167, Eubacteriales sp. MGS:0085, Bacteria sp. MGS:0575, Eubacteriales sp. MGS:0084, Eubacteriales sp. MGS:0260, Slackia isoflavoniconvertens MGS:0160, Desulfovibrionales sp. MGS:0462, Megamonas funiformis MGS:0684, Desulfovibrio piger MGS:0240, Gemmiger sp. MGS:0862, Eubacteriales sp. MGS:0671, Clostridia sp. MGS:0845, Clostridia sp. MGS:1035, Megasphaera sp. DJF_B143 MGS:1121 | 154.04746[M+Na]1+, 170.02135[M+K]1+, 132.06556[M+H]1+, 166.02763[M+Cl]1-, 130.05096[M-H]1-, 176.05641[M-H+FA]1- |
| pyridoxate | Eubacteriales sp. MGS:1167, Lachnospiraceae sp. MGS:1271, Butyricicoccus pullicaecorum MGS:1374, Bacteroides sp. MGS:0030, Enterocloster aldenensis MGS:0362, Longibaculum muris MGS:1605, Lachnospiraceae sp. MGS:0625 | 242.01891[M+NaCl+H]1+, 182.04584[M-H]1- |
| pyrraline | Eubacteriales sp. MGS:1167, Eubacteriales sp. MGS:0084, Lachnospiraceae sp. MGS:0389, Bifidobacterium catenulatum subsp. kashiwanohense MGS:0292, Oscillospiraceae sp. MGS:0074, Eubacteriales sp. MGS:0103, Clostridia sp. MGS:1035 | 486.96881[M+(NaCl)4+H]1+, 544.9275[M+(NaCl)5+H]1+ |
| pyruvate | Streptococcus oralis subsp. oralis MGS:0705, Clostridium sp. TM06-18 MGS:0048, Anaerostipes caccae MGS:0747, Eggerthella lenta MGS:0225, Lachnospiraceae sp. MGS:1271, Desulfovibrio piger MGS:0955, Actinomyces sp. ICM58 MGS:0410, Fusobacterium nucleatum subsp. animalis MGS:1418, Intestinibacillus sp. Marseille-P4005 MGS:0168, Dorea formicigenerans MGS:0006, Streptococcus gordonii MGS:0713, Enterocloster clostridioformis MGS:0686, Enterocloster aldenensis MGS:0362, Scardovia wiggsiae MGS:1737, Streptococcus mutans MGS:0677, Eubacteriales sp. MGS:0128, Clostridium sp. MGS:0050, Desulfovibrio piger MGS:0240, Anaerobutyricum hallii MGS:0012, Ruminococcus torques MGS:0034, Clostridium sp. AT4 MGS:0347, Oscillospiraceae sp. MGS:0256, Gemella morbillorum MGS:1782, Eubacteriales sp. MGS:0335, Dorea sp. AF36-15AT MGS:0052, Sutterella sp. KLE1602 MGS:0228, Clostridium sp. TF06-15AC MGS:0032 | 111.00526[M+Na]1+, 106.04987[M+NH4]1+, 133.01427[M-H+FA]1-, 144.96737[M+NaCl-H]1-, 202.92616[M+(NaCl)2-H]1-, 87.0088[M-H]1-, 122.98551[M+Cl]1- |
| quinolinate | Dialister pneumosintes MGS:1496, Streptococcus oralis subsp. oralis MGS:0705, Eubacteriales sp. MGS:0085, Eubacteriales sp. MGS:0084, Eubacteriales sp. MGS:0260, Slackia isoflavoniconvertens MGS:0160, Dorea formicigenerans MGS:0006, Streptococcus gordonii MGS:0713, Bifidobacterium catenulatum subsp. kashiwanohense MGS:0292, Scardovia wiggsiae MGS:1737, Oscillospiraceae sp. MGS:0074, Streptococcus mutans MGS:0677, Eubacteriales sp. MGS:0103, Eubacteriales sp. MGS:0128, Clostridium sp. MGS:0050, Anaerobutyricum hallii MGS:0012, Ruminococcus torques MGS:0034, Gemmiger sp. MGS:0862, Gemella morbillorum MGS:1782, Porphyromonas sp. MGS:1016, Eubacteriales sp. MGS:1313, Sutterella sp. KLE1602 MGS:0228, Clostridium sp. TF06-15AC MGS:0032 | 166.01454[M-H]1- |
| serotonin | Eubacteriales sp. MGS:1167, Eubacteriales sp. MGS:0103, Eubacteriales sp. MGS:1313 | 177.10216[M+H]1+ |
| spermidine | Eubacteriales sp. MGS:1167, Lachnospiraceae sp. MGS:0625 | 146.16522[M+H]1+ |
| stearate (18:0) | Streptococcus oralis subsp. oralis MGS:0705, Clostridium sp. TM06-18 MGS:0048, Eggerthella lenta MGS:0225, Lachnospiraceae sp. MGS:1271, Butyricicoccus pullicaecorum MGS:1374, Bacteroides sp. MGS:0030, Intestinibacillus sp. Marseille-P4005 MGS:0168, Dorea formicigenerans MGS:0006, Enterocloster clostridioformis MGS:0686, Enterocloster aldenensis MGS:0362, Eubacteriales sp. MGS:0128, Anaerobutyricum hallii MGS:0012, Ruminococcus torques MGS:0034, Clostridium sp. AT4 MGS:0347, Oscillospiraceae sp. MGS:0256, Eubacteriales sp. MGS:0335, Dorea sp. AF36-15AT MGS:0052, Bacteria sp. MGS:0459 | 307.26075[M+Na]1+, 517.1131[M+(NaCl)4+H]1+, 323.23457[M+K]1+, 515.0987[M+(NaCl)4-H]1-, 283.26443[M-H]1-, 329.27014[M-H+FA]1-, 319.24133[M+Cl]1- |
| stearoylcarnitine (C18) | Lachnospiraceae sp. MGS:1271, Intestinibacillus sp. Marseille-P4005 MGS:0168, Ruminococcus torques MGS:0034, Clostridium sp. AT4 MGS:0347, Oscillospiraceae sp. MGS:0256, Lachnospiraceae sp. MGS:0625 | 428.37321[M1+.]1+ |
| succinate | Streptococcus oralis subsp. oralis MGS:0705, Scardovia wiggsiae MGS:1737, Gemella morbillorum MGS:1782 | 141.01584[M+Na]1+, 156.98982[M+K]1+, 176.99247[M+NaCl+H]1+, 117.01937[M-H]1-, 163.02485[M-H+FA]1-, 174.97791[M+NaCl-H]1-, 152.99597[M+Cl]1- |
| tartronate (hydroxymalonate) | Eubacteriales sp. MGS:0085, Eubacteriales sp. MGS:0084, Lachnospiraceae sp. MGS:1209, Eubacteriales sp. MGS:0671, Clostridia sp. MGS:0845, Clostridia sp. MGS:1035 | 178.97173[M+NaCl+H]1+, 118.9986[M-H]1- |
| tetradecanedioate (C14-DC) | Streptococcus oralis subsp. oralis MGS:0705, Bacteroides sp. MGS:0030, Fusobacterium nucleatum subsp. animalis MGS:1418, Bifidobacterium catenulatum subsp. kashiwanohense MGS:0292, Scardovia wiggsiae MGS:1737, Dorea sp. AF36-15AT MGS:0052 | 297.14623[M+K]1+, 281.1723[M+Na]1+, 257.17598[M-H]1- |
| theanine | Eubacteriales sp. MGS:1167, Eubacteriales sp. MGS:0085, Eubacteriales sp. MGS:0084, Pseudoflavonifractor sp. BIOML-A3 MGS:0735, Lachnospiraceae sp. MGS:1209, Ruminococcus callidus MGS:0247, Eubacteriales sp. MGS:0671, Clostridia sp. MGS:0845, Clostridia sp. MGS:1035 | 192.13422[M+NH4]1+, 197.08973[M+Na]1+, 173.09315[M-H]1- |
| trans-4-hydroxyproline | Streptococcus oralis subsp. oralis MGS:0705, Anaerostipes caccae MGS:0747, Desulfovibrio piger MGS:0955, Actinomyces sp. ICM58 MGS:0410, Intestinibacillus sp. Marseille-P4005 MGS:0168, Desulfovibrionales sp. MGS:0462, Dorea formicigenerans MGS:0006, Streptococcus gordonii MGS:0713, Enterocloster aldenensis MGS:0362, Scardovia wiggsiae MGS:1737, Oscillospiraceae sp. MGS:0074, Streptococcus mutans MGS:0677, Eubacteriales sp. MGS:0128, Ruminococcus torques MGS:0034, Clostridium sp. AT4 MGS:0347, Oscillospiraceae sp. MGS:0256, Gemella morbillorum MGS:1782, Megasphaera sp. DJF_B143 MGS:1121, Sutterella sp. KLE1602 MGS:0228, Clostridium sp. TF06-15AC MGS:0032 | 154.04746[M+Na]1+, 170.02135[M+K]1+, 132.06556[M+H]1+, 166.02763[M+Cl]1-, 130.05096[M-H]1-, 176.05641[M-H+FA]1- |
| trigonelline (N'-methylnicotinate) | Lachnospiraceae sp. MGS:0389, Sutterella seckii MGS:0997, Bacteria sp. MGS:0459 | 155.08125[M+NH4]1+, 176.01081[M+K]1+, 160.03687[M+Na]1+, 136.0404[M-H]1-, 182.04584[M-H+FA]1- |
| trimethylamine N-oxide | Dialister pneumosintes MGS:1496, Eubacteriales sp. MGS:1167, Eubacteriales sp. MGS:0085, Eubacteriales sp. MGS:0084, Pseudoflavonifractor sp. BIOML-A3 MGS:0735, Eubacteriales sp. MGS:0260, Slackia isoflavoniconvertens MGS:0160, Desulfovibrionales sp. MGS:0462, Oscillospiraceae sp. MGS:0074, Eubacteriales sp. MGS:0103, Gemmiger sp. MGS:0862, Eubacteriales sp. MGS:0671, Clostridia sp. MGS:0845 | 76.07568[M+H]1+, 114.03158[M+K]1+, 98.05763[M+Na]1+ |
| tryptophan betaine | Clostridium sp. TM06-18 MGS:0048, Bacteroides ovatus MGS:0031, Eubacteriales sp. MGS:0085, Eubacteriales sp. MGS:0084, Lachnospiraceae sp. MGS:0389, Bacteroides sp. MGS:0030, Eubacteriales sp. MGS:0671, Clostridia sp. MGS:0845, Clostridia sp. MGS:1035 | 285.1[M+K]1+, 247.14407[M+H]1+, 269.12594[M+Na]1+ |
| tyrosine | Streptococcus oralis subsp. oralis MGS:0705, Clostridium sp. TM06-18 MGS:0048, Eubacteriales sp. MGS:0085, Eubacteriales sp. MGS:0084, Lachnospiraceae sp. MGS:0389, Actinomyces sp. ICM58 MGS:0410, Slackia isoflavoniconvertens MGS:0160, Dorea formicigenerans MGS:0006, Streptococcus gordonii MGS:0713, Bifidobacterium catenulatum subsp. kashiwanohense MGS:0292, Ruminococcus callidus MGS:0247, Scardovia wiggsiae MGS:1737, Oscillospiraceae sp. MGS:0074, Streptococcus mutans MGS:0677, Eubacteriales sp. MGS:0103, Clostridium sp. MGS:0050, Anaerobutyricum hallii MGS:0012, Ruminococcus torques MGS:0034, Gemmiger sp. MGS:0862, Gemella morbillorum MGS:1782, Saccharomyces cerevisiae MGS:0782, Porphyromonas sp. MGS:1016, Megasphaera sp. DJF_B143 MGS:1121, Clostridium sp. TF06-15AC MGS:0032 | 204.06309[M+Na]1+, 220.03699[M+K]1+, 182.08121[M+H]1+, 226.07223[M-H+FA]1-, 180.06664[M-H]1-, 295.98419[M+(NaCl)2-H]1-, 216.04299[M+Cl]1-, 238.02531[M+NaCl-H]1- |
| urate | Dialister pneumosintes MGS:1496, Streptococcus oralis subsp. oralis MGS:0705, Clostridium sp. TM06-18 MGS:0048, Eggerthella lenta MGS:0225, Bacteroides ovatus MGS:0031, Lachnospiraceae sp. MGS:1271, Desulfovibrio piger MGS:0955, Actinomyces sp. ICM58 MGS:0410, Bacteroides sp. MGS:0030, Fusobacterium nucleatum subsp. animalis MGS:1418, Intestinibacillus sp. Marseille-P4005 MGS:0168, Dorea formicigenerans MGS:0006, Enterocloster aldenensis MGS:0362, Megamonas funiformis MGS:0684, Eubacteriales sp. MGS:0128, Clostridium sp. MGS:0050, Desulfovibrio piger MGS:0240, Anaerobutyricum hallii MGS:0012, Ruminococcus torques MGS:0034, Longibaculum muris MGS:1605, Clostridium sp. AT4 MGS:0347, Oscillospiraceae sp. MGS:0256, Gemella morbillorum MGS:1782, Eubacteriales sp. MGS:0335, Dorea sp. AF36-15AT MGS:0052, Sutterella sp. KLE1602 MGS:0228, Clostridium sp. TF06-15AC MGS:0032 | 169.03566[M+H]1+, 191.01757[M+Na]1+, 167.02109[M-H]1-, 202.99784[M+Cl]1-, 224.97979[M+NaCl-H]1-, 282.93848[M+(NaCl)2-H]1- |
| urea | Dialister pneumosintes MGS:1496, Streptococcus oralis subsp. oralis MGS:0705, Eubacteriales sp. MGS:0085, Butyricicoccus pullicaecorum MGS:1374, Actinomyces sp. ICM58 MGS:0410, Fusobacterium nucleatum subsp. animalis MGS:1418, Streptococcus gordonii MGS:0713, Oscillospiraceae sp. MGS:0074, Streptococcus mutans MGS:0677, Eubacteriales sp. MGS:0103, Ruminococcus torques MGS:0034, Longibaculum muris MGS:1605, Gemella morbillorum MGS:1782, Porphyromonas sp. MGS:1016, Sutterella sp. KLE1602 MGS:0228 | 98.99551[M+K]1+, 83.02155[M+Na]1+, 78.06619[M+NH4]1+, 95.00173[M+Cl]1- |
| ursodeoxycholate | Streptococcus oralis subsp. oralis MGS:0705, Clostridium sp. TM06-18 MGS:0048, Anaerostipes caccae MGS:0747, Eggerthella lenta MGS:0225, Bacteroides ovatus MGS:0031, Lachnospiraceae sp. MGS:0389, Lachnospiraceae sp. MGS:1271, Butyricicoccus pullicaecorum MGS:1374, Actinomyces sp. ICM58 MGS:0410, Fusobacterium nucleatum subsp. animalis MGS:1418, Intestinibacillus sp. Marseille-P4005 MGS:0168, Slackia isoflavoniconvertens MGS:0160, Desulfovibrionales sp. MGS:0462, Dorea formicigenerans MGS:0006, Streptococcus gordonii MGS:0713, Enterocloster clostridioformis MGS:0686, Bifidobacterium catenulatum subsp. kashiwanohense MGS:0292, Enterocloster aldenensis MGS:0362, Scardovia wiggsiae MGS:1737, Oscillospiraceae sp. MGS:0074, Streptococcus mutans MGS:0677, Eubacteriales sp. MGS:0128, Clostridium sp. MGS:0050, Anaerobutyricum hallii MGS:0012, Ruminococcus torques MGS:0034, Gemmiger sp. MGS:0862, Clostridium sp. AT4 MGS:0347, Oscillospiraceae sp. MGS:0256, Gemella morbillorum MGS:1782, Saccharomyces cerevisiae MGS:0782, Eubacteriales sp. MGS:0335, Megasphaera sp. DJF_B143 MGS:1121, Clostridium sp. TF06-15AC MGS:0032 | 415.28176[M+Na]1+ |
| valine | Clostridium sp. TM06-18 MGS:0048, Lachnospiraceae sp. MGS:0389, Dorea formicigenerans MGS:0006, Ruminococcus callidus MGS:0247, Megamonas funiformis MGS:0684, Eubacteriales sp. MGS:0128, Clostridium sp. MGS:0050, Anaerobutyricum hallii MGS:0012, Ruminococcus torques MGS:0034, Gemmiger sp. MGS:0862, Longibaculum muris MGS:1605, Clostridium sp. AT4 MGS:0347, Dorea sp. AF36-15AT MGS:0052, Sutterella sp. KLE1602 MGS:0228, Clostridium sp. TF06-15AC MGS:0032 | 156.04211[M+K]1+, 140.0682[M+Na]1+, 118.0862[M+H]1+, 162.07723[M-H+FA]1-, 152.04834[M+Cl]1-, 231.989[M+(NaCl)2-H]1-, 174.0303[M+NaCl-H]1-, 116.07172[M-H]1- |
| vanillic alcohol sulfate | Eubacteriales sp. MGS:0085, Bacteria sp. MGS:0575, Eubacteriales sp. MGS:0084, Lachnospiraceae sp. MGS:0389, Eubacteriales sp. MGS:0260, Bifidobacterium catenulatum subsp. kashiwanohense MGS:0292, Ruminococcus callidus MGS:0247, Oscillospiraceae sp. MGS:0074, Streptococcus mutans MGS:0677, Eubacteriales sp. MGS:0103, Gemmiger sp. MGS:0862, Eubacteriales sp. MGS:0671, Clostridia sp. MGS:0845 | 422.88189[M+(NaCl)3+H]1+ |
| xanthine | Clostridium sp. TM06-18 MGS:0048, Eubacteriales sp. MGS:0085, Dorea formicigenerans MGS:0006, Streptococcus gordonii MGS:0713, Bifidobacterium catenulatum subsp. kashiwanohense MGS:0292, Scardovia wiggsiae MGS:1737, Streptococcus mutans MGS:0677, Clostridium sp. MGS:0050, Anaerobutyricum hallii MGS:0012, Ruminococcus torques MGS:0034, Gemella morbillorum MGS:1782, Dorea sp. AF36-15AT MGS:0052, Clostridium sp. TF06-15AC MGS:0032 | 151.02616[M-H]1-, 197.03166[M-H+FA]1- |

### Table S10 – GUTSY Atlas V < 0 mummichog enrichment results

|  |  |  |  |  |  |  |  |  |
| --- | --- | --- | --- | --- | --- | --- | --- | --- |
|  | Pathway total | Hits.total | Hits.sig | Expected | FET | EASE | Gamma | Emp.Hits |
| Eubacteriales sp. MGS:0543 | 89 | 66 | 34 | 21.379 | 0.0010156 | 0.0021146 | 0.0011283 | 0 |
| Lactobacillus delbrueckii subsp. bulgaricus MGS:0465 | 63 | 52 | 28 | 15.133 | 0.0012856 | 0.002924 | 0.0011316 | 0 |
| Eubacteriales sp. MGS:0493 | 74 | 57 | 30 | 17.776 | 0.0013805 | 0.0030001 | 0.0011319 | 0 |
| Ruminococcus sp. AM36-2AA MGS:0096 | 75 | 59 | 30 | 18.016 | 0.0029114 | 0.005925 | 0.0011436 | 0 |
| Eubacteriales sp. MGS:0646 | 43 | 30 | 18 | 10.329 | 0.0022058 | 0.0062789 | 0.0011451 | 0 |
| Lachnoclostridium sp. An14 MGS:1235 | 40 | 22 | 14 | 9.6085 | 0.0033794 | 0.010903 | 0.001164 | 0 |
| Lactococcus lactis subsp. lactis MGS:0318 | 50 | 41 | 22 | 12.011 | 0.0050288 | 0.011377 | 0.0011659 | 0 |
| Eubacterium ramulus MGS:0068 | 134 | 104 | 46 | 32.189 | 0.0077881 | 0.012287 | 0.0011697 | 0 |
| Paraprevotella xylaniphila MGS:0995 | 57 | 44 | 23 | 13.692 | 0.0063509 | 0.013662 | 0.0011754 | 0 |
| Faecalibacterium sp. MGS:0073 | 124 | 94 | 42 | 29.786 | 0.0093515 | 0.015045 | 0.0011812 | 0 |
| Oscillospiraceae sp. MGS:0057 | 121 | 92 | 41 | 29.066 | 0.01102 | 0.017667 | 0.0011922 | 0 |
| Eubacteriales sp. MGS:0589 | 108 | 87 | 39 | 25.943 | 0.01196 | 0.019392 | 0.0011995 | 0 |
| Negativibacillus massiliensis MGS:0356 | 87 | 58 | 28 | 20.899 | 0.01084 | 0.020117 | 0.0012026 | 0 |
| Eubacteriales sp. MGS:0137 | 103 | 74 | 34 | 24.742 | 0.012573 | 0.021329 | 0.0012078 | 0 |
| Oscillospiraceae sp. MGS:0146 | 126 | 104 | 45 | 30.267 | 0.014536 | 0.022146 | 0.0012113 | 0 |
| Peptoniphilus harei MGS:1800 | 34 | 26 | 15 | 8.1673 | 0.0090293 | 0.023564 | 0.0012174 | 0 |
| Bacteroidales sp. MGS:1002 | 81 | 64 | 30 | 19.457 | 0.013976 | 0.024613 | 0.001222 | 0 |
| Eubacterium ramulus MGS:0387 | 98 | 80 | 36 | 23.541 | 0.015143 | 0.024774 | 0.0012227 | 0 |
| Desulfovibrionaceae sp. MGS:0833 | 20 | 17 | 11 | 4.8043 | 0.0081662 | 0.027001 | 0.0012324 | 0 |
| Clostridium sp. OF03-18AA MGS:0119 | 131 | 105 | 45 | 31.468 | 0.018042 | 0.027073 | 0.0012327 | 1 |
| Ruminococcus sp. AM28-41 MGS:0631 | 70 | 54 | 26 | 16.815 | 0.014943 | 0.027656 | 0.0012353 | 0 |
| Eubacteriales sp. MGS:0322 | 127 | 97 | 42 | 30.507 | 0.018702 | 0.028596 | 0.0012394 | 0 |
| Eubacteriales sp. MGS:0701 | 61 | 44 | 22 | 14.653 | 0.015086 | 0.029939 | 0.0012453 | 1 |
| Lachnospiraceae sp. MGS:0246 | 92 | 73 | 33 | 22.1 | 0.018994 | 0.031397 | 0.0012518 | 0 |
| Anaerotignum faecicola MGS:0090 | 115 | 92 | 40 | 27.625 | 0.020466 | 0.031556 | 0.0012525 | 0 |
| Eubacteriales sp. MGS:0123 | 149 | 109 | 46 | 35.792 | 0.023379 | 0.034216 | 0.0012644 | 0 |
| Eggerthellales sp. MGS:0991 | 20 | 13 | 9 | 4.8043 | 0.0091476 | 0.034894 | 0.0012675 | 1 |
| Ruminococcus sp. MGS:0131 | 138 | 112 | 47 | 33.149 | 0.024532 | 0.03556 | 0.0012705 | 0 |
| Clostridium celatum MGS:0808 | 88 | 63 | 29 | 21.139 | 0.021385 | 0.036572 | 0.0012751 | 0 |
| Lachnospira pectinoschiza MGS:0099 | 86 | 63 | 29 | 20.658 | 0.021385 | 0.036572 | 0.0012751 | 0 |
| Eubacteriales sp. MGS:0856 | 117 | 90 | 39 | 28.105 | 0.02384 | 0.036599 | 0.0012752 | 0 |
| Eubacteriales sp. MGS:0881 | 51 | 40 | 20 | 12.251 | 0.02065 | 0.041057 | 0.0012956 | 0 |
| Prevotella bivia MGS:0936 | 14 | 9 | 7 | 3.363 | 0.0086744 | 0.043195 | 0.0013055 | 0 |
| Firmicutes sp. MGS:0526 | 71 | 56 | 26 | 17.055 | 0.026196 | 0.045598 | 0.0013167 | 0 |
| Eubacteriales sp. MGS:0472 | 104 | 83 | 36 | 24.982 | 0.030137 | 0.046477 | 0.0013209 | 0 |
| Ruminococcus sp. AF21-42 MGS:0111 | 143 | 111 | 46 | 34.351 | 0.034389 | 0.048944 | 0.0013326 | 1 |
| Eubacteriales sp. MGS:1289 | 48 | 33 | 17 | 11.53 | 0.023358 | 0.049096 | 0.0013333 | 0 |
| Veillonella tobetsuensis MGS:1344 | 100 | 78 | 34 | 24.021 | 0.032658 | 0.050871 | 0.0013418 | 0 |
| Lachnospiraceae sp. MGS:0252 | 107 | 78 | 34 | 25.703 | 0.032658 | 0.050871 | 0.0013418 | 0 |
| Erysipelotrichales sp. MGS:0283 | 127 | 89 | 38 | 30.507 | 0.0341 | 0.051206 | 0.0013434 | 0 |
| Clostridia sp. MGS:0011 | 114 | 81 | 35 | 27.384 | 0.034916 | 0.053589 | 0.0013548 | 0 |
| Ruminococcus sp. MGS:0126 | 93 | 73 | 32 | 22.34 | 0.03525 | 0.055527 | 0.0013643 | 0 |
| Firmicutes sp. MGS:1178 | 32 | 21 | 12 | 7.6868 | 0.021823 | 0.055643 | 0.0013648 | 0 |
| Ruminococcus sp. MGS:0487 | 71 | 49 | 23 | 17.055 | 0.03177 | 0.056522 | 0.0013691 | 2 |
| Eubacterium sp. MGS:0248 | 122 | 98 | 41 | 29.306 | 0.03993 | 0.057938 | 0.0013761 | 0 |
| Coprococcus sp. AM27-12LB MGS:0687 | 131 | 98 | 41 | 31.468 | 0.03993 | 0.057938 | 0.0013761 | 0 |
| Eubacteriales sp. MGS:1093 | 27 | 14 | 9 | 6.4858 | 0.018199 | 0.058406 | 0.0013784 | 1 |
| Coprococcus eutactus MGS:0155 | 109 | 79 | 34 | 26.183 | 0.040366 | 0.061643 | 0.0013944 | 1 |
| Holdemania sp. Marseille-P2844 MGS:0201 | 53 | 39 | 19 | 12.731 | 0.033118 | 0.063035 | 0.0014014 | 1 |
| Lachnospiraceae sp. MGS:0233 | 112 | 82 | 35 | 26.904 | 0.042894 | 0.064598 | 0.0014092 | 0 |
| Blautia sp. AF19-10LB MGS:0157 | 151 | 124 | 50 | 36.272 | 0.047492 | 0.064644 | 0.0014095 | 0 |
| Eubacteriales sp. MGS:0298 | 135 | 113 | 46 | 32.429 | 0.049158 | 0.068116 | 0.0014271 | 1 |
| Bacteroides finegoldii MGS:0224 | 48 | 37 | 18 | 11.53 | 0.038644 | 0.073474 | 0.0014548 | 0 |
| Eubacteriales sp. MGS:0093 | 80 | 53 | 24 | 19.217 | 0.045707 | 0.076616 | 0.0014713 | 0 |

### Table S11 – GUTSY Atlas V < 0 mummichog enrichment matches

| Compound identifier | Pathway name | Matches [adduct] |
| --- | --- | --- |
| (14 or 15)-methylpalmitate (a17:0 or i17:0) | Lactobacillus delbrueckii subsp. bulgaricus MGS:0465, Eubacteriales sp. MGS:0646, Lactococcus lactis subsp. lactis MGS:0318, Eubacterium ramulus MGS:0068, Faecalibacterium sp. MGS:0073, Eubacteriales sp. MGS:0589, Negativibacillus massiliensis MGS:0356, Clostridium sp. OF03-18AA MGS:0119, Lachnospiraceae sp. MGS:0246, Anaerotignum faecicola MGS:0090, Eubacteriales sp. MGS:0123, Clostridium celatum MGS:0808, Firmicutes sp. MGS:0526, Eubacteriales sp. MGS:0472, Eubacteriales sp. MGS:1289 | 288.28966[M+NH4]1+, 271.26311[M+H]1+, 293.24506[M+Na]1+, 309.21886[M+K]1+, 305.22558[M+Cl]1-, 269.24861[M-H]1-, 315.25445[M-H+FA]1- |
| (16 or 17)-methylstearate (a19:0 or i19:0) | Lactobacillus delbrueckii subsp. bulgaricus MGS:0465, Eubacteriales sp. MGS:0646, Lactococcus lactis subsp. lactis MGS:0318, Eubacterium ramulus MGS:0068, Faecalibacterium sp. MGS:0073, Eubacteriales sp. MGS:0589, Negativibacillus massiliensis MGS:0356, Eubacteriales sp. MGS:0137, Clostridium sp. OF03-18AA MGS:0119, Lachnospiraceae sp. MGS:0246, Anaerotignum faecicola MGS:0090, Eubacteriales sp. MGS:0123, Clostridium celatum MGS:0808, Firmicutes sp. MGS:0526, Eubacteriales sp. MGS:0472, Eubacteriales sp. MGS:1289 | 316.32098[M+NH4]1+, 321.27636[M+Na]1+ |
| 1-(1-enyl-palmitoyl)-GPC (P-16:0)* | Eubacteriales sp. MGS:0543, Eubacteriales sp. MGS:0493, Eubacteriales sp. MGS:0646, Eubacteriales sp. MGS:0589, Negativibacillus massiliensis MGS:0356, Eubacteriales sp. MGS:0137, Eubacteriales sp. MGS:0322, Eubacteriales sp. MGS:0701, Clostridium celatum MGS:0808, Eubacteriales sp. MGS:0856 | 502.3267[M+Na]1+ |
| 1-arachidonoyl-GPC (20:4n6)* | Lachnoclostridium sp. An14 MGS:1235, Eubacteriales sp. MGS:0589, Peptoniphilus harei MGS:1800, Eubacteriales sp. MGS:0123 | 566.3213[M+Na]1+, 582.2951[M+K]1+, 578.3017[M+Cl]1- |
| 1-arachidonoyl-GPE (20:4n6)* | Eubacteriales sp. MGS:0123, Eggerthellales sp. MGS:0991 | 524.2743[M+Na]1+, 500.2782[M-H]1- |
| 1-linoleoyl-GPC (18:2) | Eubacteriales sp. MGS:0543, Eubacteriales sp. MGS:0493, Eubacteriales sp. MGS:0646, Lachnoclostridium sp. An14 MGS:1235, Eubacteriales sp. MGS:0589, Eubacteriales sp. MGS:0137, Bacteroidales sp. MGS:1002, Clostridium sp. OF03-18AA MGS:0119, Eubacteriales sp. MGS:0322, Eubacteriales sp. MGS:0701, Clostridium celatum MGS:0808, Eubacteriales sp. MGS:0856, Firmicutes sp. MGS:0526, Eubacteriales sp. MGS:0472 | 520.3396[M+H]1+, 558.2951[M+K]1+, 542.3217[M+Na]1+, 554.3018[M+Cl]1- |
| 1-linoleoyl-GPE (18:2)* | Eubacteriales sp. MGS:0543, Clostridium sp. OF03-18AA MGS:0119, Eubacteriales sp. MGS:0856 | 500.2746[M+Na]1+, 476.27818[M-H]1- |
| 1-methylnicotinamide | Lactococcus lactis subsp. lactis MGS:0318, Faecalibacterium sp. MGS:0073, Oscillospiraceae sp. MGS:0057, Negativibacillus massiliensis MGS:0356, Eubacteriales sp. MGS:0123, Lachnospira pectinoschiza MGS:0099 | 160.06041[M+Na]1+, 137.07099[M1+.]1+ |
| 1-palmitoyl-GPC (16:0) | Eubacteriales sp. MGS:0543, Lactobacillus delbrueckii subsp. bulgaricus MGS:0465, Eubacteriales sp. MGS:0646, Eggerthellales sp. MGS:0991 | 496.3397[M+H]1+, 534.2956[M+K]1+, 518.3217[M+Na]1+, 530.3016[M+Cl]1-, 540.3304[M-H+FA]1- |
| 1-ribosyl-imidazoleacetate* | Eubacteriales sp. MGS:0543, Eubacteriales sp. MGS:0493, Eubacteriales sp. MGS:0589, Oscillospiraceae sp. MGS:0146, Eubacterium ramulus MGS:0387, Clostridium sp. OF03-18AA MGS:0119, Eubacteriales sp. MGS:0322, Lachnospiraceae sp. MGS:0246, Ruminococcus sp. MGS:0131, Eubacteriales sp. MGS:0856, Eubacteriales sp. MGS:0472 | 490.92608[M+(NaCl)4+H]1+, 276.11906[M+NH4]1+ |
| 1-stearoyl-GPC (18:0) | Eubacteriales sp. MGS:0543, Eubacteriales sp. MGS:0493, Eubacteriales sp. MGS:0137, Eubacteriales sp. MGS:0322, Eggerthellales sp. MGS:0991, Eubacteriales sp. MGS:0856 | 524.371[M+H]1+, 562.3265[M+K]1+, 546.353[M+Na]1+, 558.333[M+Cl]1-, 568.3616[M-H+FA]1- |
| 1-stearoyl-GPE (18:0) | Eubacteriales sp. MGS:0646 | 504.3059[M+Na]1+, 520.2799[M+K]1+, 480.30906[M-H]1- |
| 10-undecenoate (11:1n1) | Ruminococcus sp. AM36-2AA MGS:0096, Lactococcus lactis subsp. lactis MGS:0318, Eubacterium ramulus MGS:0068, Faecalibacterium sp. MGS:0073, Oscillospiraceae sp. MGS:0057, Eubacteriales sp. MGS:0589, Eubacteriales sp. MGS:0137, Oscillospiraceae sp. MGS:0146, Bacteroidales sp. MGS:1002, Clostridium sp. OF03-18AA MGS:0119, Ruminococcus sp. AM28-41 MGS:0631, Eubacteriales sp. MGS:0322, Lachnospiraceae sp. MGS:0246, Anaerotignum faecicola MGS:0090, Ruminococcus sp. MGS:0131, Clostridium celatum MGS:0808, Lachnospira pectinoschiza MGS:0099, Firmicutes sp. MGS:0526, Eubacteriales sp. MGS:0472, Ruminococcus sp. AF21-42 MGS:0111 | 185.15363[M+H]1+, 207.13564[M+Na]1+, 202.18018[M+NH4]1+, 223.10945[M+K]1+, 229.14459[M-H+FA]1-, 183.13902[M-H]1- |
| 2-hydroxyhippurate (salicylurate) | Lachnoclostridium sp. An14 MGS:1235, Eubacteriales sp. MGS:0322, Clostridium celatum MGS:0808, Eubacteriales sp. MGS:0856 | 218.04242[M+Na]1+, 194.04584[M-H]1- |
| 2-hydroxyibuprofen | Paraprevotella xylaniphila MGS:0995 | 261.08864[M+K]1+, 245.11488[M+Na]1+, 267.1239[M-H+FA]1- |
| 2-piperidinone | Ruminococcus sp. AM36-2AA MGS:0096, Eubacterium ramulus MGS:0068, Oscillospiraceae sp. MGS:0146, Peptoniphilus harei MGS:1800, Eubacterium ramulus MGS:0387, Lachnospiraceae sp. MGS:0246, Eggerthellales sp. MGS:0991, Clostridium celatum MGS:0808, Lachnospira pectinoschiza MGS:0099, Prevotella bivia MGS:0936, Ruminococcus sp. AF21-42 MGS:0111 | 138.03162[M+K]1+, 117.10223[M+NH4]1+, 100.07566[M+H]1+, 122.05762[M+Na]1+, 144.06662[M-H+FA]1- |
| 2-stearoyl-GPE (18:0)* | Eubacteriales sp. MGS:0646, Eubacteriales sp. MGS:0589 | 504.3059[M+Na]1+, 520.2799[M+K]1+, 480.30906[M-H]1- |
| 2R,3R-dihydroxybutyrate | Lactobacillus delbrueckii subsp. bulgaricus MGS:0465, Eubacterium ramulus MGS:0068, Oscillospiraceae sp. MGS:0057, Eubacteriales sp. MGS:0123 | 143.03153[M+Na]1+, 159.00544[M+K]1+, 155.01164[M+Cl]1-, 165.04037[M-H+FA]1-, 119.03496[M-H]1-, 176.99351[M+NaCl-H]1- |
| 2S,3R-dihydroxybutyrate | Lactobacillus delbrueckii subsp. bulgaricus MGS:0465, Lachnoclostridium sp. An14 MGS:1235, Lactococcus lactis subsp. lactis MGS:0318, Eubacterium ramulus MGS:0068, Paraprevotella xylaniphila MGS:0995, Faecalibacterium sp. MGS:0073, Oscillospiraceae sp. MGS:0057, Negativibacillus massiliensis MGS:0356, Oscillospiraceae sp. MGS:0146, Eubacterium ramulus MGS:0387, Clostridium sp. OF03-18AA MGS:0119, Ruminococcus sp. AM28-41 MGS:0631, Anaerotignum faecicola MGS:0090, Eubacteriales sp. MGS:0123, Ruminococcus sp. MGS:0131, Lachnospira pectinoschiza MGS:0099, Prevotella bivia MGS:0936, Eubacteriales sp. MGS:0472, Ruminococcus sp. AF21-42 MGS:0111 | 143.03153[M+Na]1+, 159.00544[M+K]1+, 155.01164[M+Cl]1-, 165.04037[M-H+FA]1-, 119.03496[M-H]1-, 176.99351[M+NaCl-H]1- |
| 3,4-dihydroxybutyrate | Paraprevotella xylaniphila MGS:0995 | 143.03153[M+Na]1+, 159.00544[M+K]1+, 155.01164[M+Cl]1-, 165.04037[M-H+FA]1-, 119.03496[M-H]1-, 176.99351[M+NaCl-H]1- |
| 3-(3-hydroxyphenyl)propionate | Eubacteriales sp. MGS:0543, Ruminococcus sp. AM36-2AA MGS:0096, Oscillospiraceae sp. MGS:0146, Peptoniphilus harei MGS:1800, Eubacterium ramulus MGS:0387, Ruminococcus sp. AM28-41 MGS:0631, Eubacteriales sp. MGS:0322, Clostridium celatum MGS:0808, Lachnospira pectinoschiza MGS:0099, Ruminococcus sp. AF21-42 MGS:0111 | 456.86413[M+(NaCl)5+H]1+, 167.07032[M+H]1+, 189.05231[M+Na]1+, 165.05567[M-H]1- |
| 3-carboxy-4-methyl-5-pentyl-2-furanpropionate (3-CMPFP)** | Eubacteriales sp. MGS:0543, Lactobacillus delbrueckii subsp. bulgaricus MGS:0465, Eubacteriales sp. MGS:0493, Ruminococcus sp. AM36-2AA MGS:0096, Lactococcus lactis subsp. lactis MGS:0318, Eubacterium ramulus MGS:0068, Faecalibacterium sp. MGS:0073, Oscillospiraceae sp. MGS:0057, Negativibacillus massiliensis MGS:0356, Eubacteriales sp. MGS:0137, Oscillospiraceae sp. MGS:0146, Bacteroidales sp. MGS:1002, Eubacterium ramulus MGS:0387, Clostridium sp. OF03-18AA MGS:0119, Ruminococcus sp. AM28-41 MGS:0631, Eubacteriales sp. MGS:0322, Eubacteriales sp. MGS:0701, Lachnospiraceae sp. MGS:0246, Anaerotignum faecicola MGS:0090, Eubacteriales sp. MGS:0123, Ruminococcus sp. MGS:0131, Clostridium celatum MGS:0808, Lachnospira pectinoschiza MGS:0099, Eubacteriales sp. MGS:0856, Eubacteriales sp. MGS:0881, Firmicutes sp. MGS:0526, Eubacteriales sp. MGS:0472, Ruminococcus sp. AF21-42 MGS:0111, Eubacteriales sp. MGS:1289 | 307.09418[M+K]1+, 291.12028[M+Na]1+, 267.1239[M-H]1- |
| 3-carboxy-4-methyl-5-propyl-2-furanpropanoate (CMPF) | Eubacteriales sp. MGS:0543, Lactobacillus delbrueckii subsp. bulgaricus MGS:0465, Eubacteriales sp. MGS:0493, Ruminococcus sp. AM36-2AA MGS:0096, Lachnoclostridium sp. An14 MGS:1235, Eubacterium ramulus MGS:0068, Faecalibacterium sp. MGS:0073, Oscillospiraceae sp. MGS:0057, Negativibacillus massiliensis MGS:0356, Eubacteriales sp. MGS:0137, Oscillospiraceae sp. MGS:0146, Bacteroidales sp. MGS:1002, Eubacterium ramulus MGS:0387, Clostridium sp. OF03-18AA MGS:0119, Ruminococcus sp. AM28-41 MGS:0631, Eubacteriales sp. MGS:0322, Eubacteriales sp. MGS:0701, Lachnospiraceae sp. MGS:0246, Anaerotignum faecicola MGS:0090, Eubacteriales sp. MGS:0123, Ruminococcus sp. MGS:0131, Clostridium celatum MGS:0808, Eubacteriales sp. MGS:0856, Eubacteriales sp. MGS:0881, Eubacteriales sp. MGS:0472, Ruminococcus sp. AF21-42 MGS:0111 | 279.06292[M+K]1+, 263.08899[M+Na]1+, 239.09254[M-H]1- |
| 3-hydroxyadipate | Paraprevotella xylaniphila MGS:0995 | 162.05248[M1+.]1+, 185.04207[M+Na]1+, 201.01601[M+K]1+, 180.08656[M+NH4]1+, 163.06011[M+H]1+, 197.0223[M+Cl]1-, 207.0511[M-H+FA]1-, 161.04555[M-H]1- |
| 3-hydroxyhippurate | Eubacteriales sp. MGS:0543, Ruminococcus sp. AM36-2AA MGS:0096, Oscillospiraceae sp. MGS:0057, Oscillospiraceae sp. MGS:0146, Eubacterium ramulus MGS:0387, Clostridium sp. OF03-18AA MGS:0119, Ruminococcus sp. AM28-41 MGS:0631, Eubacteriales sp. MGS:0322, Lachnospiraceae sp. MGS:0246, Clostridium celatum MGS:0808, Lachnospira pectinoschiza MGS:0099, Ruminococcus sp. AF21-42 MGS:0111 | 218.04242[M+Na]1+, 194.04584[M-H]1- |
| 3-hydroxyquinine | Bacteroidales sp. MGS:1002, Desulfovibrionaceae sp. MGS:0833, Anaerotignum faecicola MGS:0090, Prevotella bivia MGS:0936 | 340.17797[M1+.]1+, 341.18536[M+H]1+ |
| 3-methyl-2-oxobutyrate | Ruminococcus sp. AM36-2AA MGS:0096, Lactococcus lactis subsp. lactis MGS:0318, Eubacterium ramulus MGS:0068, Oscillospiraceae sp. MGS:0057, Oscillospiraceae sp. MGS:0146, Eubacterium ramulus MGS:0387, Ruminococcus sp. AM28-41 MGS:0631, Anaerotignum faecicola MGS:0090, Eubacteriales sp. MGS:0123, Ruminococcus sp. MGS:0131, Ruminococcus sp. AF21-42 MGS:0111 | 134.08116[M+NH4]1+, 175.01313[M+NaCl+H]1+, 155.01048[M+K]1+, 117.05462[M+H]1+, 139.03657[M+Na]1+, 151.01669[M+Cl]1-, 115.0401[M-H]1-, 172.99862[M+NaCl-H]1-, 161.04555[M-H+FA]1- |
| 3-methyl-2-oxovalerate | Ruminococcus sp. AM36-2AA MGS:0096, Lactococcus lactis subsp. lactis MGS:0318, Eubacterium ramulus MGS:0068, Oscillospiraceae sp. MGS:0057, Ruminococcus sp. AM28-41 MGS:0631, Anaerotignum faecicola MGS:0090, Eubacteriales sp. MGS:0123, Ruminococcus sp. MGS:0131, Ruminococcus sp. AF21-42 MGS:0111 | 148.09679[M+NH4]1+, 131.07032[M+H]1+, 153.05223[M+Na]1+, 169.02609[M+K]1+, 302.93176[M+(NaCl)3-H]1-, 244.97303[M+(NaCl)2-H]1-, 129.05583[M-H]1-, 187.01434[M+NaCl-H]1-, 175.06119[M-H+FA]1-, 165.03234[M+Cl]1- |
| 3-methyladipate | Eubacteriales sp. MGS:0543, Lactobacillus delbrueckii subsp. bulgaricus MGS:0465, Eubacteriales sp. MGS:0493, Faecalibacterium sp. MGS:0073, Eubacteriales sp. MGS:0589, Negativibacillus massiliensis MGS:0356, Eubacteriales sp. MGS:0137, Oscillospiraceae sp. MGS:0146, Bacteroidales sp. MGS:1002, Eubacterium ramulus MGS:0387, Clostridium sp. OF03-18AA MGS:0119, Eubacteriales sp. MGS:0322, Eubacteriales sp. MGS:0701, Lachnospiraceae sp. MGS:0246, Ruminococcus sp. MGS:0131, Clostridium celatum MGS:0808, Eubacteriales sp. MGS:0856, Eubacteriales sp. MGS:0881, Firmicutes sp. MGS:0526, Eubacteriales sp. MGS:0472 | 178.10742[M+NH4]1+, 199.03677[M+K]1+, 161.08087[M+H]1+, 450.87306[M+(NaCl)5+H]1+, 183.06283[M+Na]1+, 195.04294[M+Cl]1-, 159.06625[M-H]1-, 205.07185[M-H+FA]1- |
| 3-methylhistidine | Lachnoclostridium sp. An14 MGS:1235, Faecalibacterium sp. MGS:0073, Oscillospiraceae sp. MGS:0057, Negativibacillus massiliensis MGS:0356, Eubacteriales sp. MGS:0137, Oscillospiraceae sp. MGS:0146, Bacteroidales sp. MGS:1002, Eubacterium ramulus MGS:0387, Clostridium sp. OF03-18AA MGS:0119, Eubacteriales sp. MGS:0322, Eubacteriales sp. MGS:0701, Anaerotignum faecicola MGS:0090, Eubacteriales sp. MGS:0123, Ruminococcus sp. MGS:0131, Eubacteriales sp. MGS:0472 | 187.11903[M+NH4]1+, 170.0924[M+H]1+, 208.04828[M+K]1+, 192.07433[M+Na]1+ |
| 4-hydroxy-2-oxoglutaric acid | Lactobacillus delbrueckii subsp. bulgaricus MGS:0465, Ruminococcus sp. AM36-2AA MGS:0096, Eubacterium ramulus MGS:0068, Faecalibacterium sp. MGS:0073, Eubacteriales sp. MGS:0137, Oscillospiraceae sp. MGS:0146, Eubacteriales sp. MGS:0322, Anaerotignum faecicola MGS:0090, Ruminococcus sp. MGS:0131, Lachnospira pectinoschiza MGS:0099, Ruminococcus sp. AF21-42 MGS:0111 | 207.01466[M-H+FA]1-, 161.00918[M-H]1- |
| 4-hydroxyhippurate | Eubacteriales sp. MGS:0543, Peptoniphilus harei MGS:1800, Eubacterium ramulus MGS:0387, Clostridium celatum MGS:0808, Eubacteriales sp. MGS:0856 | 218.04242[M+Na]1+, 194.04584[M-H]1- |
| 4-hydroxyphenylpyruvate | Ruminococcus sp. AM36-2AA MGS:0096, Oscillospiraceae sp. MGS:0146, Ruminococcus sp. MGS:0131 | 180.04207[M1+.]1+, 203.0318[M+Na]1+, 179.03499[M-H]1- |
| 4-methyl-2-oxopentanoate | Lactobacillus delbrueckii subsp. bulgaricus MGS:0465, Ruminococcus sp. AM36-2AA MGS:0096, Lactococcus lactis subsp. lactis MGS:0318, Eubacterium ramulus MGS:0068, Oscillospiraceae sp. MGS:0057, Oscillospiraceae sp. MGS:0146, Eubacterium ramulus MGS:0387, Ruminococcus sp. AM28-41 MGS:0631, Anaerotignum faecicola MGS:0090, Eubacteriales sp. MGS:0123, Ruminococcus sp. MGS:0131, Ruminococcus sp. AF21-42 MGS:0111, Eubacteriales sp. MGS:1289 | 148.09679[M+NH4]1+, 131.07032[M+H]1+, 153.05223[M+Na]1+, 169.02609[M+K]1+, 302.93176[M+(NaCl)3-H]1-, 244.97303[M+(NaCl)2-H]1-, 129.05583[M-H]1-, 187.01434[M+NaCl-H]1-, 175.06119[M-H+FA]1-, 165.03234[M+Cl]1- |
| 5-dodecenoate (12:1n7) | Eubacteriales sp. MGS:0589 | 199.1693[M+H]1+, 216.19589[M+NH4]1+, 221.15125[M+Na]1+, 197.15478[M-H]1-, 243.16009[M-H+FA]1- |
| 5-methyluridine (ribothymidine) | Eubacteriales sp. MGS:0543, Eubacteriales sp. MGS:0589, Eubacteriales sp. MGS:0137, Clostridium sp. OF03-18AA MGS:0119, Eubacteriales sp. MGS:0322, Clostridium celatum MGS:0808, Eubacteriales sp. MGS:0856 | 490.92608[M+(NaCl)4+H]1+, 276.11906[M+NH4]1+ |
| N-acetyl-beta-alanine | Faecalibacterium sp. MGS:0073, Oscillospiraceae sp. MGS:0057, Peptoniphilus harei MGS:1800, Desulfovibrionaceae sp. MGS:0833, Eubacteriales sp. MGS:0123 | 154.04746[M+Na]1+, 170.02135[M+K]1+, 132.06556[M+H]1+, 176.05641[M-H+FA]1-, 166.02763[M+Cl]1-, 130.05096[M-H]1- |
| N-acetylalanine | Peptoniphilus harei MGS:1800, Prevotella bivia MGS:0936 | 154.04746[M+Na]1+, 170.02135[M+K]1+, 132.06556[M+H]1+, 176.05641[M-H+FA]1-, 166.02763[M+Cl]1-, 130.05096[M-H]1- |
| N-acetylglycine | Lactobacillus delbrueckii subsp. bulgaricus MGS:0465, Eubacteriales sp. MGS:0646, Paraprevotella xylaniphila MGS:0995, Faecalibacterium sp. MGS:0073, Eubacteriales sp. MGS:0589, Negativibacillus massiliensis MGS:0356, Eubacteriales sp. MGS:0137, Desulfovibrionaceae sp. MGS:0833, Clostridium sp. OF03-18AA MGS:0119, Eubacteriales sp. MGS:0322, Eubacteriales sp. MGS:0701, Anaerotignum faecicola MGS:0090, Eubacteriales sp. MGS:0123, Ruminococcus sp. MGS:0131, Eubacteriales sp. MGS:0856, Eubacteriales sp. MGS:0881, Prevotella bivia MGS:0936, Firmicutes sp. MGS:0526, Eubacteriales sp. MGS:0472 | 156.00574[M+K]1+, 140.03182[M+Na]1+, 176.00847[M+NaCl+H]1+, 118.0499[M+H]1+, 116.03533[M-H]1-, 152.01196[M+Cl]1- |
| N-acetylserine | Eubacteriales sp. MGS:0493, Peptoniphilus harei MGS:1800 | 170.04238[M+Na]1+, 186.01634[M+K]1+, 148.06044[M+H]1+, 204.00448[M+NaCl-H]1-, 319.9221[M+(NaCl)3-H]1-, 182.02254[M+Cl]1-, 261.96322[M+(NaCl)2-H]1-, 146.0459[M-H]1- |
| N-acetyltaurine | Eubacteriales sp. MGS:0493, Peptoniphilus harei MGS:1800 | 168.0325[M+H]1+, 190.01446[M+Na]1+, 166.01788[M-H]1- |
| N-formylphenylalanine | Lactobacillus delbrueckii subsp. bulgaricus MGS:0465, Ruminococcus sp. AM36-2AA MGS:0096, Lachnoclostridium sp. An14 MGS:1235, Eubacterium ramulus MGS:0068, Faecalibacterium sp. MGS:0073, Oscillospiraceae sp. MGS:0057, Eubacteriales sp. MGS:0137, Oscillospiraceae sp. MGS:0146, Bacteroidales sp. MGS:1002, Eubacterium ramulus MGS:0387, Clostridium sp. OF03-18AA MGS:0119, Ruminococcus sp. AM28-41 MGS:0631, Eubacteriales sp. MGS:0322, Lachnospiraceae sp. MGS:0246, Anaerotignum faecicola MGS:0090, Ruminococcus sp. MGS:0131, Lachnospira pectinoschiza MGS:0099, Ruminococcus sp. AF21-42 MGS:0111 | 216.06323[M+Na]1+ |
| N-lactoyl isoleucine | Eubacterium ramulus MGS:0068, Anaerotignum faecicola MGS:0090, Eubacteriales sp. MGS:0123 | 225.10971[M+Na]1+, 241.08351[M+K]1+, 201.1133[M-H]1-, 118.04113[M-H+Cl]2-, 237.09004[M+Cl]1- |
| N-methylproline | Eubacteriales sp. MGS:0543, Eubacteriales sp. MGS:0493, Ruminococcus sp. AM36-2AA MGS:0096, Eubacteriales sp. MGS:0646, Eubacterium ramulus MGS:0068, Faecalibacterium sp. MGS:0073, Eubacteriales sp. MGS:0589, Eubacteriales sp. MGS:0137, Oscillospiraceae sp. MGS:0146, Bacteroidales sp. MGS:1002, Eubacterium ramulus MGS:0387, Clostridium sp. OF03-18AA MGS:0119, Eubacteriales sp. MGS:0322, Lachnospiraceae sp. MGS:0246, Ruminococcus sp. MGS:0131, Eubacteriales sp. MGS:0856, Eubacteriales sp. MGS:0881, Firmicutes sp. MGS:0526, Eubacteriales sp. MGS:0472, Ruminococcus sp. AF21-42 MGS:0111 | 152.06821[M+Na]1+, 147.11281[M+NH4]1+, 168.04216[M+K]1+, 130.08631[M+H]1+ |
| N1-Methyl-2-pyridone-5-carboxamide | Eubacterium ramulus MGS:0068, Faecalibacterium sp. MGS:0073, Oscillospiraceae sp. MGS:0057, Negativibacillus massiliensis MGS:0356, Oscillospiraceae sp. MGS:0146, Eubacteriales sp. MGS:0123, Ruminococcus sp. MGS:0131 | 153.06581[M+H]1+, 170.0924[M+NH4]1+, 175.04782[M+Na]1+, 151.05123[M-H]1-, 187.02799[M+Cl]1-, 197.05676[M-H+FA]1- |
| N1-methyladenosine | Eubacteriales sp. MGS:0123 | 299.14646[M+NH4]1+, 282.11962[M+H]1+ |
| N6-methyladenosine | Eubacteriales sp. MGS:0123 | 299.14646[M+NH4]1+, 282.11962[M+H]1+ |
| S-allylcysteine | Eubacteriales sp. MGS:0493, Faecalibacterium sp. MGS:0073, Oscillospiraceae sp. MGS:0057, Negativibacillus massiliensis MGS:0356, Eubacteriales sp. MGS:0137, Oscillospiraceae sp. MGS:0146, Bacteroidales sp. MGS:1002, Eubacterium ramulus MGS:0387, Clostridium sp. OF03-18AA MGS:0119, Eubacteriales sp. MGS:0322, Lachnospiraceae sp. MGS:0246, Ruminococcus sp. MGS:0131, Eubacteriales sp. MGS:0881, Eubacteriales sp. MGS:0472 | 184.04031[M+Na]1+, 162.05831[M+H]1+, 179.08461[M+NH4]1+, 160.04377[M-H]1- |
| S-methylcysteine | Eubacteriales sp. MGS:0543, Eubacteriales sp. MGS:0493, Paraprevotella xylaniphila MGS:0995, Faecalibacterium sp. MGS:0073, Eubacteriales sp. MGS:0589, Eubacteriales sp. MGS:0137, Oscillospiraceae sp. MGS:0146, Bacteroidales sp. MGS:1002, Eubacterium ramulus MGS:0387, Clostridium sp. OF03-18AA MGS:0119, Eubacteriales sp. MGS:0322, Eubacteriales sp. MGS:0701, Lachnospiraceae sp. MGS:0246, Ruminococcus sp. MGS:0131, Clostridium celatum MGS:0808, Eubacteriales sp. MGS:0856, Eubacteriales sp. MGS:0881, Firmicutes sp. MGS:0526, Eubacteriales sp. MGS:0472, Eubacteriales sp. MGS:1289 | 158.02466[M+Na]1+, 173.9985[M+K]1+ |
| S-methylcysteine sulfoxide | Eubacteriales sp. MGS:0543, Eubacteriales sp. MGS:0493, Paraprevotella xylaniphila MGS:0995, Faecalibacterium sp. MGS:0073, Oscillospiraceae sp. MGS:0057, Eubacteriales sp. MGS:0589, Eubacteriales sp. MGS:0137, Oscillospiraceae sp. MGS:0146, Bacteroidales sp. MGS:1002, Eubacterium ramulus MGS:0387, Clostridium sp. OF03-18AA MGS:0119, Ruminococcus sp. AM28-41 MGS:0631, Eubacteriales sp. MGS:0322, Eubacteriales sp. MGS:0701, Lachnospiraceae sp. MGS:0246, Ruminococcus sp. MGS:0131, Clostridium celatum MGS:0808, Eubacteriales sp. MGS:0856, Eubacteriales sp. MGS:0881, Firmicutes sp. MGS:0526, Eubacteriales sp. MGS:0472, Ruminococcus sp. AF21-42 MGS:0111, Eubacteriales sp. MGS:1289 | 174.01951[M+Na]1+, 152.03757[M+H]1+, 209.9962[M+NaCl+H]1+ |
| acetylcarnitine (C2) | Paraprevotella xylaniphila MGS:0995, Eubacteriales sp. MGS:0589, Eubacteriales sp. MGS:0137, Eubacteriales sp. MGS:0123, Ruminococcus sp. MGS:0131 | 263.08899[M+NaCl+H]1+, 204.12306[M1+.]1+, 239.09254[M+Cl]1- |
| alanine | Eubacterium ramulus MGS:0068, Anaerotignum faecicola MGS:0090, Eubacteriales sp. MGS:0123, Lachnospira pectinoschiza MGS:0099, Ruminococcus sp. AF21-42 MGS:0111 | 128.01083[M+K]1+, 112.03688[M+Na]1+, 90.05497[M+H]1+, 134.04592[M-H+FA]1-, 124.01703[M+Cl]1-, 88.04043[M-H]1-, 145.99895[M+NaCl-H]1-, 203.95768[M+(NaCl)2-H]1- |
| andro steroid monosulfate C19H28O6S (1)* | Eubacterium ramulus MGS:0068, Paraprevotella xylaniphila MGS:0995, Oscillospiraceae sp. MGS:0057, Ruminococcus sp. AF21-42 MGS:0111 | 369.17469[M-H]1- |
| androstenediol (3beta,17beta) monosulfate (1) | Ruminococcus sp. AM36-2AA MGS:0096, Eubacterium ramulus MGS:0068, Faecalibacterium sp. MGS:0073, Oscillospiraceae sp. MGS:0057, Oscillospiraceae sp. MGS:0146, Ruminococcus sp. AM28-41 MGS:0631, Anaerotignum faecicola MGS:0090, Eubacteriales sp. MGS:0123, Lachnospira pectinoschiza MGS:0099, Ruminococcus sp. AF21-42 MGS:0111 | 369.17469[M-H]1- |
| androsterone sulfate | Eubacteriales sp. MGS:0543, Lachnoclostridium sp. An14 MGS:1235, Faecalibacterium sp. MGS:0073, Oscillospiraceae sp. MGS:0057, Eubacteriales sp. MGS:0137, Bacteroidales sp. MGS:1002, Eubacteriales sp. MGS:0123, Ruminococcus sp. MGS:0131, Lachnospira pectinoschiza MGS:0099 | 369.17469[M-H]1- |
| arabonate/xylonate | Eubacteriales sp. MGS:0543, Eubacteriales sp. MGS:0493, Oscillospiraceae sp. MGS:0146, Peptoniphilus harei MGS:1800, Clostridium sp. OF03-18AA MGS:0119, Eubacteriales sp. MGS:0322 | 184.08167[M+NH4]1+, 205.01127[M+K]1+, 166.04748[M1+.]1+, 165.04037[M-H]1-, 201.01754[M+Cl]1- |
| arachidate (20:0) | Eubacteriales sp. MGS:0646, Faecalibacterium sp. MGS:0073, Eubacteriales sp. MGS:0589, Negativibacillus massiliensis MGS:0356, Eubacteriales sp. MGS:0137, Bacteroidales sp. MGS:1002, Desulfovibrionaceae sp. MGS:0833, Clostridium sp. OF03-18AA MGS:0119, Eubacteriales sp. MGS:0881, Firmicutes sp. MGS:0526, Eubacteriales sp. MGS:0472 | 335.29197[M+Na]1+ |
| arachidonate (20:4n6) | Eubacterium ramulus MGS:0068, Eubacteriales sp. MGS:0589, Negativibacillus massiliensis MGS:0356, Anaerotignum faecicola MGS:0090, Eubacteriales sp. MGS:0123 | 339.20992[M+Cl]1-, 303.23333[M-H]1- |
| argininate* | Eubacteriales sp. MGS:0543, Ruminococcus sp. AM36-2AA MGS:0096, Faecalibacterium sp. MGS:0073, Eubacteriales sp. MGS:0589, Oscillospiraceae sp. MGS:0146, Bacteroidales sp. MGS:1002, Eubacterium ramulus MGS:0387, Clostridium sp. OF03-18AA MGS:0119, Ruminococcus sp. AM28-41 MGS:0631, Eubacteriales sp. MGS:0322, Eubacteriales sp. MGS:0701, Lachnospiraceae sp. MGS:0246, Ruminococcus sp. MGS:0131, Eubacteriales sp. MGS:0856, Eubacteriales sp. MGS:0881, Firmicutes sp. MGS:0526, Eubacteriales sp. MGS:0472, Ruminococcus sp. AF21-42 MGS:0111 | 349.9795[M+(NaCl)3+H]1+, 292.02074[M+(NaCl)2+H]1+, 198.08497[M+Na]1+, 176.10295[M+H]1+, 214.05884[M+K]1+, 174.08838[M-H]1-, 290.0059[M+(NaCl)2-H]1-, 232.04696[M+NaCl-H]1-, 210.06513[M+Cl]1- |
| arginine | Eubacteriales sp. MGS:0856, Eubacteriales sp. MGS:1289 | 197.10093[M+Na]1+, 175.11895[M+H]1+, 173.10437[M-H]1-, 209.08114[M+Cl]1- |
| benzoate | Lactobacillus delbrueckii subsp. bulgaricus MGS:0465, Lactococcus lactis subsp. lactis MGS:0318, Eubacterium ramulus MGS:0387, Eubacteriales sp. MGS:0322, Ruminococcus sp. MGS:0131, Clostridium celatum MGS:0808, Ruminococcus sp. AF21-42 MGS:0111 | 123.04394[M+H]1+, 145.02617[M+Na]1+, 167.03489[M-H+FA]1-, 157.00621[M+Cl]1-, 121.02952[M-H]1- |
| beta-alanine | Eubacterium ramulus MGS:0068, Oscillospiraceae sp. MGS:0057, Anaerotignum faecicola MGS:0090, Eubacteriales sp. MGS:0123, Ruminococcus sp. MGS:0131, Lachnospira pectinoschiza MGS:0099 | 128.01083[M+K]1+, 112.03688[M+Na]1+, 90.05497[M+H]1+, 134.04592[M-H+FA]1-, 124.01703[M+Cl]1-, 88.04043[M-H]1-, 145.99895[M+NaCl-H]1-, 203.95768[M+(NaCl)2-H]1- |
| butyrate/isobutyrate (4:0) | Eubacteriales sp. MGS:0493, Ruminococcus sp. AM36-2AA MGS:0096, Lactococcus lactis subsp. lactis MGS:0318, Oscillospiraceae sp. MGS:0146, Eubacterium ramulus MGS:0387, Clostridium sp. OF03-18AA MGS:0119, Eubacteriales sp. MGS:0322, Lachnospiraceae sp. MGS:0246, Clostridium celatum MGS:0808, Eubacteriales sp. MGS:0856, Ruminococcus sp. AF21-42 MGS:0111 | 106.08625[M+NH4]1+, 147.01836[M+NaCl+H]1+, 127.01562[M+K]1+, 89.05968[M+H]1+, 111.04165[M+Na]1+, 87.04515[M-H]1-, 133.05065[M-H+FA]1- |
| caprate (10:0) | Eubacteriales sp. MGS:0646, Paraprevotella xylaniphila MGS:0995, Faecalibacterium sp. MGS:0073, Eubacteriales sp. MGS:0589, Clostridium sp. OF03-18AA MGS:0119, Eubacteriales sp. MGS:0701, Clostridium celatum MGS:0808, Eubacteriales sp. MGS:0472 | 173.15362[M+H]1+, 211.10943[M+K]1+, 195.13561[M+Na]1+, 217.14454[M-H+FA]1-, 171.13907[M-H]1- |
| chiro-inositol | Eubacteriales sp. MGS:0543, Eubacteriales sp. MGS:0493, Ruminococcus sp. AM36-2AA MGS:0096, Paraprevotella xylaniphila MGS:0995, Faecalibacterium sp. MGS:0073, Eubacteriales sp. MGS:0589, Eubacteriales sp. MGS:0137, Oscillospiraceae sp. MGS:0146, Bacteroidales sp. MGS:1002, Eubacterium ramulus MGS:0387, Clostridium sp. OF03-18AA MGS:0119, Eubacteriales sp. MGS:0322, Eubacteriales sp. MGS:0701, Lachnospiraceae sp. MGS:0246, Ruminococcus sp. MGS:0131, Eubacteriales sp. MGS:0856, Eubacteriales sp. MGS:0881, Firmicutes sp. MGS:0526, Eubacteriales sp. MGS:0472 | 296.98805[M+(NaCl)2+H]1+, 180.06309[M1+.]1+, 198.09727[M+NH4]1+, 203.0526[M+Na]1+, 219.02652[M+K]1+, 225.06167[M-H+FA]1-, 215.03291[M+Cl]1-, 179.05615[M-H]1- |
| citrulline | Eggerthellales sp. MGS:0991, Eubacteriales sp. MGS:0856 | 349.9795[M+(NaCl)3+H]1+, 292.02074[M+(NaCl)2+H]1+, 198.08497[M+Na]1+, 176.10295[M+H]1+, 214.05884[M+K]1+, 174.08838[M-H]1-, 290.0059[M+(NaCl)2-H]1-, 232.04696[M+NaCl-H]1-, 210.06513[M+Cl]1- |
| creatine | Eubacterium ramulus MGS:0068, Negativibacillus massiliensis MGS:0356, Anaerotignum faecicola MGS:0090, Eubacteriales sp. MGS:0123, Lachnospira pectinoschiza MGS:0099 | 154.05869[M+Na]1+, 132.07679[M+H]1+, 170.03266[M+K]1+, 130.06214[M-H]1-, 166.03884[M+Cl]1- |
| cystine | Eubacteriales sp. MGS:0123, Eggerthellales sp. MGS:0991 | 278.98691[M+K]1+, 263.01303[M+Na]1+, 239.01669[M-H]1- |
| dehydroepiandrosterone sulfate (DHEA-S) | Eubacteriales sp. MGS:0543, Ruminococcus sp. AM36-2AA MGS:0096, Eubacterium ramulus MGS:0068, Faecalibacterium sp. MGS:0073, Oscillospiraceae sp. MGS:0057, Bacteroidales sp. MGS:1002, Eubacteriales sp. MGS:0123, Ruminococcus sp. MGS:0131, Lachnospira pectinoschiza MGS:0099, Ruminococcus sp. AF21-42 MGS:0111 | 367.15912[M-H]1- |
| epiandrosterone sulfate | Eubacteriales sp. MGS:0646, Lachnoclostridium sp. An14 MGS:1235, Faecalibacterium sp. MGS:0073, Oscillospiraceae sp. MGS:0057, Eubacteriales sp. MGS:0137, Bacteroidales sp. MGS:1002, Eubacteriales sp. MGS:0123, Ruminococcus sp. MGS:0131, Lachnospira pectinoschiza MGS:0099, Eubacteriales sp. MGS:0472 | 369.17469[M-H]1- |
| erythritol | Eubacteriales sp. MGS:0493, Peptoniphilus harei MGS:1800, Ruminococcus sp. MGS:0131 | 123.06522[M+H]1+, 145.04716[M+Na]1+, 161.02098[M+K]1+, 157.02726[M+Cl]1- |
| ethyl glucuronide | Lactobacillus delbrueckii subsp. bulgaricus MGS:0465, Lachnoclostridium sp. An14 MGS:1235, Eubacterium ramulus MGS:0068, Paraprevotella xylaniphila MGS:0995, Faecalibacterium sp. MGS:0073, Oscillospiraceae sp. MGS:0057, Negativibacillus massiliensis MGS:0356, Desulfovibrionaceae sp. MGS:0833, Ruminococcus sp. AM28-41 MGS:0631, Anaerotignum faecicola MGS:0090, Eubacteriales sp. MGS:0123, Ruminococcus sp. MGS:0131, Lachnospira pectinoschiza MGS:0099, Ruminococcus sp. AF21-42 MGS:0111 | 245.06319[M+Na]1+, 222.07354[M1+.]1+, 221.0666[M-H]1-, 267.07228[M-H+FA]1- |
| fructose | Paraprevotella xylaniphila MGS:0995 | 296.98805[M+(NaCl)2+H]1+, 180.06309[M1+.]1+, 198.09727[M+NH4]1+, 203.0526[M+Na]1+, 219.02652[M+K]1+, 225.06167[M-H+FA]1-, 215.03291[M+Cl]1-, 179.05615[M-H]1- |
| fumarate | Clostridium sp. OF03-18AA MGS:0119, Eubacteriales sp. MGS:0701, Anaerotignum faecicola MGS:0090, Ruminococcus sp. MGS:0131, Eubacteriales sp. MGS:0472 | 134.0448[M+NH4]1+, 232.9354[M+(NaCl)2+H]1+, 161.00918[M-H+FA]1-, 115.00368[M-H]1- |
| galactonate | Eubacteriales sp. MGS:0543, Eubacterium ramulus MGS:0068, Bacteroidales sp. MGS:1002, Desulfovibrionaceae sp. MGS:0833, Eubacteriales sp. MGS:0322, Lachnospiraceae sp. MGS:0246 | 219.04749[M+Na]1+, 196.0581[M1+.]1+, 241.0565[M-H+FA]1-, 195.05102[M-H]1- |
| glucose | Lactococcus lactis subsp. lactis MGS:0318, Eubacterium ramulus MGS:0068, Ruminococcus sp. AM28-41 MGS:0631, Eubacteriales sp. MGS:0123, Lachnospira pectinoschiza MGS:0099 | 296.98805[M+(NaCl)2+H]1+, 180.06309[M1+.]1+, 198.09727[M+NH4]1+, 203.0526[M+Na]1+, 219.02652[M+K]1+, 225.06167[M-H+FA]1-, 215.03291[M+Cl]1-, 179.05615[M-H]1- |
| glutamate | Ruminococcus sp. AM36-2AA MGS:0096, Eubacterium ramulus MGS:0068, Oscillospiraceae sp. MGS:0057, Anaerotignum faecicola MGS:0090, Eubacteriales sp. MGS:0123, Lachnospira pectinoschiza MGS:0099, Ruminococcus sp. AF21-42 MGS:0111 | 170.04238[M+Na]1+, 186.01634[M+K]1+, 148.06044[M+H]1+, 204.00448[M+NaCl-H]1-, 319.9221[M+(NaCl)3-H]1-, 182.02254[M+Cl]1-, 261.96322[M+(NaCl)2-H]1-, 146.0459[M-H]1- |
| glutamine | Eubacteriales sp. MGS:0856, Eubacteriales sp. MGS:1289 | 169.05837[M+Na]1+, 147.07644[M+H]1+, 185.03233[M+K]1+, 434.85492[M+(NaCl)5-H]1-, 376.89704[M+(NaCl)4-H]1-, 181.03859[M+Cl]1-, 203.02058[M+NaCl-H]1-, 145.06183[M-H]1-, 318.9382[M+(NaCl)3-H]1- |
| glutarate (C5-DC) | Ruminococcus sp. AM36-2AA MGS:0096, Eubacterium ramulus MGS:0068, Peptoniphilus harei MGS:1800, Eubacterium ramulus MGS:0387, Lachnospiraceae sp. MGS:0246, Clostridium celatum MGS:0808, Lachnospira pectinoschiza MGS:0099, Prevotella bivia MGS:0936, Ruminococcus sp. AF21-42 MGS:0111 | 191.00811[M+NaCl+H]1+, 248.96668[M+(NaCl)2+H]1+, 171.00542[M+K]1+, 150.07609[M+NH4]1+, 155.03145[M+Na]1+, 131.03498[M-H]1-, 177.04047[M-H+FA]1- |
| glutarylcarnitine (C5-DC) | Oscillospiraceae sp. MGS:0146, Peptoniphilus harei MGS:1800, Bacteroidales sp. MGS:1002, Clostridium sp. OF03-18AA MGS:0119, Lachnospiraceae sp. MGS:0246, Lachnospira pectinoschiza MGS:0099 | 335.11021[M+NaCl+H]1+, 276.14418[M1+.]1+ |
| glycerophosphoethanolamine | Eubacteriales sp. MGS:0543, Eubacteriales sp. MGS:0493, Lachnoclostridium sp. An14 MGS:1235 | 216.06323[M+H]1+, 214.04889[M-H]1- |
| glycerophosphorylcholine (GPC) | Eubacteriales sp. MGS:0543, Eubacteriales sp. MGS:0493, Eubacteriales sp. MGS:0137, Eubacteriales sp. MGS:0701 | 258.11002[M+H]1+, 280.09201[M+Na]1+, 431.98657[M+(NaCl)3+H]1+, 296.066[M+K]1+, 487.9304[M+(NaCl)4-H]1- |
| glycocholate | Paraprevotella xylaniphila MGS:0995, Faecalibacterium sp. MGS:0073, Oscillospiraceae sp. MGS:0146, Eubacterium ramulus MGS:0387, Clostridium sp. OF03-18AA MGS:0119, Eubacteriales sp. MGS:0322, Lachnospiraceae sp. MGS:0246, Clostridium celatum MGS:0808, Eubacteriales sp. MGS:0856 | 524.2743[M+NaCl+H]1+, 500.2782[M+Cl]1- |
| glycohyocholate | Eubacteriales sp. MGS:0543, Eubacteriales sp. MGS:0493, Lachnoclostridium sp. An14 MGS:1235, Faecalibacterium sp. MGS:0073, Eubacteriales sp. MGS:0589, Oscillospiraceae sp. MGS:0146, Clostridium sp. OF03-18AA MGS:0119, Eubacteriales sp. MGS:0322, Eubacteriales sp. MGS:0701, Eggerthellales sp. MGS:0991, Clostridium celatum MGS:0808, Eubacteriales sp. MGS:0856, Eubacteriales sp. MGS:0881, Firmicutes sp. MGS:0526, Eubacteriales sp. MGS:0472 | 524.2743[M+NaCl+H]1+, 500.2782[M+Cl]1- |
| heptanoate (7:0) | Eubacteriales sp. MGS:0493, Negativibacillus massiliensis MGS:0356, Eubacteriales sp. MGS:0137, Desulfovibrionaceae sp. MGS:0833, Clostridium sp. OF03-18AA MGS:0119, Eubacteriales sp. MGS:0322, Eubacteriales sp. MGS:0856, Eubacteriales sp. MGS:0472, Eubacteriales sp. MGS:1289 | 169.06238[M+K]1+, 131.10671[M+H]1+, 153.08859[M+Na]1+, 129.09216[M-H]1-, 175.09755[M-H+FA]1- |
| hexadecadienoate (16:2n6) | Lactobacillus delbrueckii subsp. bulgaricus MGS:0465, Ruminococcus sp. AM36-2AA MGS:0096, Faecalibacterium sp. MGS:0073, Eubacteriales sp. MGS:0589, Clostridium sp. OF03-18AA MGS:0119, Ruminococcus sp. AM28-41 MGS:0631, Anaerotignum faecicola MGS:0090, Ruminococcus sp. MGS:0131 | 275.19812[M+Na]1+ |
| hexadecanedioate (C16-DC) | Paraprevotella xylaniphila MGS:0995 | 287.22161[M+H]1+, 309.20359[M+Na]1+, 304.24824[M+NH4]1+, 325.17754[M+K]1+ |
| histidine | Eubacteriales sp. MGS:0137, Lachnospiraceae sp. MGS:0246, Eubacteriales sp. MGS:0856, Ruminococcus sp. AF21-42 MGS:0111, Eubacteriales sp. MGS:1289 | 173.10342[M+NH4]1+, 156.07679[M+H]1+, 178.05867[M+Na]1+, 194.03269[M+K]1+, 190.03891[M+Cl]1-, 212.02085[M+NaCl-H]1-, 154.0622[M-H]1- |
| ibuprofen | Paraprevotella xylaniphila MGS:0995 | 229.1201[M+Na]1+, 206.12981[M1+.]1+, 438.97168[M+(NaCl)4+H]1+, 381.01309[M+(NaCl)3+H]1+ |
| isobutyrylcarnitine (C4) | Eubacteriales sp. MGS:0543, Eubacteriales sp. MGS:0589, Negativibacillus massiliensis MGS:0356, Eubacteriales sp. MGS:0322, Lachnospiraceae sp. MGS:0246, Clostridium celatum MGS:0808, Eubacteriales sp. MGS:0856, Eubacteriales sp. MGS:0472 | 232.15431[M1+.]1+, 291.12028[M+NaCl+H]1+, 267.1239[M+Cl]1- |
| isoleucine | Ruminococcus sp. AM36-2AA MGS:0096, Eubacterium ramulus MGS:0068, Oscillospiraceae sp. MGS:0057, Oscillospiraceae sp. MGS:0146, Anaerotignum faecicola MGS:0090, Eubacteriales sp. MGS:0123, Ruminococcus sp. AF21-42 MGS:0111 | 154.08383[M+Na]1+, 132.10192[M+H]1+, 170.05777[M+K]1+, 303.96367[M+(NaCl)3-H]1-, 188.046[M+NaCl-H]1-, 166.06401[M+Cl]1-, 246.00463[M+(NaCl)2-H]1-, 130.08735[M-H]1-, 176.09282[M-H+FA]1- |
| isovalerate (i5:0) | Eubacteriales sp. MGS:0543, Lactococcus lactis subsp. lactis MGS:0318, Oscillospiraceae sp. MGS:0057, Anaerotignum faecicola MGS:0090, Eubacteriales sp. MGS:0123, Ruminococcus sp. AF21-42 MGS:0111 | 120.10193[M+NH4]1+, 141.03119[M+K]1+, 103.07534[M+H]1+, 125.05734[M+Na]1+, 147.06621[M-H+FA]1-, 101.06078[M-H]1- |
| leucine | Ruminococcus sp. AM36-2AA MGS:0096, Eubacterium ramulus MGS:0068, Oscillospiraceae sp. MGS:0057, Oscillospiraceae sp. MGS:0146, Anaerotignum faecicola MGS:0090, Eubacteriales sp. MGS:0123, Ruminococcus sp. MGS:0131, Lachnospira pectinoschiza MGS:0099, Ruminococcus sp. AF21-42 MGS:0111 | 154.08383[M+Na]1+, 132.10192[M+H]1+, 170.05777[M+K]1+, 303.96367[M+(NaCl)3-H]1-, 188.046[M+NaCl-H]1-, 166.06401[M+Cl]1-, 246.00463[M+(NaCl)2-H]1-, 130.08735[M-H]1-, 176.09282[M-H+FA]1- |
| lysine | Eubacterium ramulus MGS:0068, Anaerotignum faecicola MGS:0090, Eubacteriales sp. MGS:0123, Ruminococcus sp. AF21-42 MGS:0111 | 169.09475[M+Na]1+, 147.11281[M+H]1+, 185.06872[M+K]1+, 181.07497[M+Cl]1-, 145.0983[M-H]1- |
| mannose | Lactococcus lactis subsp. lactis MGS:0318, Eubacterium ramulus MGS:0068, Oscillospiraceae sp. MGS:0057, Negativibacillus massiliensis MGS:0356, Ruminococcus sp. AM28-41 MGS:0631, Eubacteriales sp. MGS:0123 | 296.98805[M+(NaCl)2+H]1+, 180.06309[M1+.]1+, 198.09727[M+NH4]1+, 203.0526[M+Na]1+, 219.02652[M+K]1+, 225.06167[M-H+FA]1-, 215.03291[M+Cl]1-, 179.05615[M-H]1- |
| margarate (17:0) | Lactobacillus delbrueckii subsp. bulgaricus MGS:0465, Lactococcus lactis subsp. lactis MGS:0318, Eubacterium ramulus MGS:0068, Eubacteriales sp. MGS:0589, Negativibacillus massiliensis MGS:0356, Ruminococcus sp. AM28-41 MGS:0631, Anaerotignum faecicola MGS:0090, Eubacteriales sp. MGS:0123, Firmicutes sp. MGS:0526, Eubacteriales sp. MGS:0472, Eubacteriales sp. MGS:1289 | 288.28966[M+NH4]1+, 271.26311[M+H]1+, 293.24506[M+Na]1+, 309.21886[M+K]1+, 305.22558[M+Cl]1-, 269.24861[M-H]1-, 315.25445[M-H+FA]1- |
| methionine | Oscillospiraceae sp. MGS:0146, Eubacterium ramulus MGS:0387, Clostridium sp. OF03-18AA MGS:0119, Lachnospiraceae sp. MGS:0246, Eubacteriales sp. MGS:0856, Ruminococcus sp. AF21-42 MGS:0111 | 172.04022[M+Na]1+, 150.05837[M+H]1+, 188.01428[M+K]1+, 184.02024[M+Cl]1-, 148.04382[M-H]1-, 321.91935[M+(NaCl)3-H]1- |
| methyl glucopyranoside (alpha + beta) | Eubacteriales sp. MGS:0543, Lactobacillus delbrueckii subsp. bulgaricus MGS:0465, Eubacteriales sp. MGS:0493, Ruminococcus sp. AM36-2AA MGS:0096, Eubacteriales sp. MGS:0646, Lachnoclostridium sp. An14 MGS:1235, Paraprevotella xylaniphila MGS:0995, Faecalibacterium sp. MGS:0073, Oscillospiraceae sp. MGS:0057, Eubacteriales sp. MGS:0589, Eubacteriales sp. MGS:0137, Oscillospiraceae sp. MGS:0146, Bacteroidales sp. MGS:1002, Eubacterium ramulus MGS:0387, Clostridium sp. OF03-18AA MGS:0119, Ruminococcus sp. AM28-41 MGS:0631, Eubacteriales sp. MGS:0322, Eubacteriales sp. MGS:0701, Lachnospiraceae sp. MGS:0246, Ruminococcus sp. MGS:0131, Lachnospira pectinoschiza MGS:0099, Eubacteriales sp. MGS:0856, Eubacteriales sp. MGS:0881, Firmicutes sp. MGS:0526, Eubacteriales sp. MGS:0472, Ruminococcus sp. AF21-42 MGS:0111, Eubacteriales sp. MGS:1289 | 194.07886[M1+.]1+, 239.07737[M-H+FA]1-, 229.0483[M+Cl]1-, 193.07173[M-H]1- |
| methylsuccinate | Eubacteriales sp. MGS:0701, Eubacteriales sp. MGS:0856, Firmicutes sp. MGS:0526 | 191.00811[M+NaCl+H]1+, 248.96668[M+(NaCl)2+H]1+, 171.00542[M+K]1+, 150.07609[M+NH4]1+, 155.03145[M+Na]1+, 131.03498[M-H]1-, 177.04047[M-H+FA]1- |
| myo-inositol | Eubacteriales sp. MGS:0493, Paraprevotella xylaniphila MGS:0995, Faecalibacterium sp. MGS:0073, Oscillospiraceae sp. MGS:0057, Eubacteriales sp. MGS:0137, Oscillospiraceae sp. MGS:0146, Bacteroidales sp. MGS:1002, Eubacterium ramulus MGS:0387, Clostridium sp. OF03-18AA MGS:0119, Eubacteriales sp. MGS:0322, Lachnospiraceae sp. MGS:0246, Ruminococcus sp. MGS:0131, Eubacteriales sp. MGS:0856, Eubacteriales sp. MGS:0472, Ruminococcus sp. AF21-42 MGS:0111 | 296.98805[M+(NaCl)2+H]1+, 180.06309[M1+.]1+, 198.09727[M+NH4]1+, 203.0526[M+Na]1+, 219.02652[M+K]1+, 225.06167[M-H+FA]1-, 215.03291[M+Cl]1-, 179.05615[M-H]1- |
| myristoleate (14:1n5) | Lactobacillus delbrueckii subsp. bulgaricus MGS:0465, Eubacteriales sp. MGS:0589 | 265.15633[M+K]1+, 227.20052[M+H]1+, 249.18245[M+Na]1+, 225.18604[M-H]1- |
| naproxen | Lactobacillus delbrueckii subsp. bulgaricus MGS:0465, Ruminococcus sp. AM28-41 MGS:0631, Clostridium celatum MGS:0808 | 253.0836[M+Na]1+, 460.92165[M+(NaCl)4-H]1-, 402.96338[M+(NaCl)3-H]1- |
| nonadecanoate (19:0) | Lactobacillus delbrueckii subsp. bulgaricus MGS:0465, Eubacteriales sp. MGS:0646, Lactococcus lactis subsp. lactis MGS:0318, Faecalibacterium sp. MGS:0073, Eubacteriales sp. MGS:0589, Negativibacillus massiliensis MGS:0356, Eubacteriales sp. MGS:0137, Bacteroidales sp. MGS:1002, Desulfovibrionaceae sp. MGS:0833, Clostridium sp. OF03-18AA MGS:0119, Eubacteriales sp. MGS:0322, Anaerotignum faecicola MGS:0090, Ruminococcus sp. MGS:0131, Clostridium celatum MGS:0808, Eubacteriales sp. MGS:0856, Eubacteriales sp. MGS:0881, Firmicutes sp. MGS:0526, Eubacteriales sp. MGS:0472, Eubacteriales sp. MGS:1289 | 316.32098[M+NH4]1+, 321.27636[M+Na]1+ |
| oxalate (ethanedioate) | Eubacteriales sp. MGS:0543, Lactobacillus delbrueckii subsp. bulgaricus MGS:0465, Eubacteriales sp. MGS:0493, Ruminococcus sp. AM36-2AA MGS:0096, Lactococcus lactis subsp. lactis MGS:0318, Eubacterium ramulus MGS:0068, Paraprevotella xylaniphila MGS:0995, Faecalibacterium sp. MGS:0073, Oscillospiraceae sp. MGS:0057, Eubacteriales sp. MGS:0589, Negativibacillus massiliensis MGS:0356, Eubacteriales sp. MGS:0137, Oscillospiraceae sp. MGS:0146, Bacteroidales sp. MGS:1002, Eubacterium ramulus MGS:0387, Desulfovibrionaceae sp. MGS:0833, Clostridium sp. OF03-18AA MGS:0119, Eubacteriales sp. MGS:0322, Eubacteriales sp. MGS:0701, Lachnospiraceae sp. MGS:0246, Ruminococcus sp. MGS:0131, Clostridium celatum MGS:0808, Lachnospira pectinoschiza MGS:0099, Eubacteriales sp. MGS:0856, Eubacteriales sp. MGS:0881, Firmicutes sp. MGS:0526, Eubacteriales sp. MGS:0472, Ruminococcus sp. AF21-42 MGS:0111, Eubacteriales sp. MGS:1289 | 206.91985[M+(NaCl)2+H]1+, 148.96118[M+NaCl+H]1+, 146.94667[M+NaCl-H]1-, 88.98811[M-H]1- |
| palmitate (16:0) | Eubacterium ramulus MGS:0068, Eubacteriales sp. MGS:0589, Ruminococcus sp. AM28-41 MGS:0631, Anaerotignum faecicola MGS:0090, Eubacteriales sp. MGS:0123 | 279.22936[M+Na]1+, 295.20328[M+K]1+, 257.24743[M+H]1+, 291.20978[M+Cl]1-, 301.23867[M-H+FA]1-, 255.23309[M-H]1- |
| pentadecanoate (15:0) | Lactobacillus delbrueckii subsp. bulgaricus MGS:0465, Eubacteriales sp. MGS:0646, Lactococcus lactis subsp. lactis MGS:0318, Eubacterium ramulus MGS:0068, Eubacteriales sp. MGS:0589, Clostridium sp. OF03-18AA MGS:0119, Anaerotignum faecicola MGS:0090, Ruminococcus sp. MGS:0131, Firmicutes sp. MGS:0526 | 281.18759[M+K]1+, 265.21376[M+Na]1+, 241.21734[M-H]1-, 287.22299[M-H+FA]1- |
| pentose acid* | Eubacteriales sp. MGS:0543, Lactobacillus delbrueckii subsp. bulgaricus MGS:0465, Eubacteriales sp. MGS:0493, Lachnoclostridium sp. An14 MGS:1235, Paraprevotella xylaniphila MGS:0995, Faecalibacterium sp. MGS:0073, Oscillospiraceae sp. MGS:0057, Eubacteriales sp. MGS:0589, Eubacteriales sp. MGS:0137, Oscillospiraceae sp. MGS:0146, Bacteroidales sp. MGS:1002, Eubacterium ramulus MGS:0387, Clostridium sp. OF03-18AA MGS:0119, Ruminococcus sp. AM28-41 MGS:0631, Eubacteriales sp. MGS:0322, Eubacteriales sp. MGS:0701, Lachnospiraceae sp. MGS:0246, Ruminococcus sp. MGS:0131, Lachnospira pectinoschiza MGS:0099, Eubacteriales sp. MGS:0856, Eubacteriales sp. MGS:0881, Firmicutes sp. MGS:0526, Eubacteriales sp. MGS:0472, Ruminococcus sp. AF21-42 MGS:0111 | 120.10193[M+NH4]1+, 141.03119[M+K]1+, 103.07534[M+H]1+, 125.05734[M+Na]1+, 147.06621[M-H+FA]1-, 101.06078[M-H]1- |
| phenol sulfate | Eubacterium ramulus MGS:0068, Faecalibacterium sp. MGS:0073, Oscillospiraceae sp. MGS:0146, Peptoniphilus harei MGS:1800, Bacteroidales sp. MGS:1002, Eubacterium ramulus MGS:0387, Desulfovibrionaceae sp. MGS:0833, Ruminococcus sp. AM28-41 MGS:0631, Anaerotignum faecicola MGS:0090, Eubacteriales sp. MGS:0123, Ruminococcus sp. MGS:0131, Eubacteriales sp. MGS:0881, Prevotella bivia MGS:0936 | 172.99136[M-H]1- |
| phosphate | Eubacteriales sp. MGS:0543, Bacteroidales sp. MGS:1002, Clostridium sp. OF03-18AA MGS:0119, Eubacteriales sp. MGS:0322, Ruminococcus sp. MGS:0131, Eubacteriales sp. MGS:0856 | 120.96616[M+Na]1+, 136.94014[M+K]1+, 98.98417[M+H]1+, 270.84561[M+(NaCl)3-H]1-, 96.96961[M-H]1-, 132.94632[M+Cl]1-, 328.80461[M+(NaCl)4-H]1-, 386.76345[M+(NaCl)5-H]1-, 142.97508[M-H+FA]1-, 154.92829[M+NaCl-H]1-, 212.88697[M+(NaCl)2-H]1- |
| propionylglycine | Eubacteriales sp. MGS:0543, Lactobacillus delbrueckii subsp. bulgaricus MGS:0465, Eubacteriales sp. MGS:0646, Eubacteriales sp. MGS:0589, Oscillospiraceae sp. MGS:0146, Eubacterium ramulus MGS:0387, Desulfovibrionaceae sp. MGS:0833, Eubacteriales sp. MGS:0322, Eubacteriales sp. MGS:0701, Lachnospiraceae sp. MGS:0246, Anaerotignum faecicola MGS:0090, Eggerthellales sp. MGS:0991, Eubacteriales sp. MGS:0881, Firmicutes sp. MGS:0526, Ruminococcus sp. AF21-42 MGS:0111 | 154.04746[M+Na]1+, 170.02135[M+K]1+, 132.06556[M+H]1+, 176.05641[M-H+FA]1-, 166.02763[M+Cl]1-, 130.05096[M-H]1- |
| pseudouridine | Peptoniphilus harei MGS:1800, Eubacteriales sp. MGS:1289 | 267.05883[M+Na]1+, 289.06815[M-H+FA]1-, 279.03907[M+Cl]1-, 243.0623[M-H]1- |
| quinate | Oscillospiraceae sp. MGS:0057, Negativibacillus massiliensis MGS:0356, Ruminococcus sp. AF21-42 MGS:0111 | 215.05262[M+Na]1+, 192.06307[M1+.]1+, 210.09718[M+NH4]1+, 191.05615[M-H]1- |
| ribonate | Eubacteriales sp. MGS:0493, Peptoniphilus harei MGS:1800 | 184.08167[M+NH4]1+, 205.01127[M+K]1+, 166.04748[M1+.]1+, 165.04037[M-H]1-, 201.01754[M+Cl]1- |
| sarcosine | Ruminococcus sp. AM28-41 MGS:0631 | 128.01083[M+K]1+, 112.03688[M+Na]1+, 90.05497[M+H]1+, 134.04592[M-H+FA]1-, 124.01703[M+Cl]1-, 88.04043[M-H]1-, 145.99895[M+NaCl-H]1-, 203.95768[M+(NaCl)2-H]1- |
| sebacate (C10-DC) | Eubacteriales sp. MGS:0589, Eubacteriales sp. MGS:0472 | 225.10971[M+Na]1+, 241.08351[M+K]1+, 201.1133[M-H]1-, 118.04113[M-H+Cl]2-, 237.09004[M+Cl]1- |
| serine | Eubacteriales sp. MGS:0322, Clostridium celatum MGS:0808, Eubacteriales sp. MGS:0856, Ruminococcus sp. AF21-42 MGS:0111 | 164.00833[M+NaCl+H]1+, 128.03181[M+Na]1+, 144.00582[M+K]1+, 106.04987[M+H]1+, 277.91141[M+(NaCl)3-H]1-, 219.95258[M+(NaCl)2-H]1-, 161.99397[M+NaCl-H]1-, 104.03534[M-H]1-, 140.012[M+Cl]1- |
| stachydrine | Eubacteriales sp. MGS:0543, Eubacteriales sp. MGS:0493, Ruminococcus sp. AM36-2AA MGS:0096, Eubacteriales sp. MGS:0646, Eubacterium ramulus MGS:0068, Paraprevotella xylaniphila MGS:0995, Faecalibacterium sp. MGS:0073, Oscillospiraceae sp. MGS:0057, Eubacteriales sp. MGS:0589, Eubacteriales sp. MGS:0137, Oscillospiraceae sp. MGS:0146, Bacteroidales sp. MGS:1002, Eubacterium ramulus MGS:0387, Clostridium sp. OF03-18AA MGS:0119, Eubacteriales sp. MGS:0322, Eubacteriales sp. MGS:0701, Lachnospiraceae sp. MGS:0246, Ruminococcus sp. MGS:0131, Eubacteriales sp. MGS:0856, Eubacteriales sp. MGS:0881, Firmicutes sp. MGS:0526, Eubacteriales sp. MGS:0472, Ruminococcus sp. AF21-42 MGS:0111 | 166.08396[M+Na]1+, 182.05784[M+K]1+, 144.10193[M+H]1+, 161.12847[M+NH4]1+, 178.06403[M+Cl]1- |
| stearate (18:0) | Eubacteriales sp. MGS:0646, Eubacterium ramulus MGS:0068, Eubacteriales sp. MGS:0589, Negativibacillus massiliensis MGS:0356, Ruminococcus sp. AM28-41 MGS:0631, Anaerotignum faecicola MGS:0090, Eubacteriales sp. MGS:0123, Eubacteriales sp. MGS:0472 | 307.26075[M+Na]1+, 517.1131[M+(NaCl)4+H]1+, 323.23457[M+K]1+, 283.26443[M-H]1-, 515.0987[M+(NaCl)4-H]1-, 329.27014[M-H+FA]1-, 319.24133[M+Cl]1- |
| tartronate (hydroxymalonate) | Eubacteriales sp. MGS:0543, Lactobacillus delbrueckii subsp. bulgaricus MGS:0465, Eubacteriales sp. MGS:0493, Ruminococcus sp. AM36-2AA MGS:0096, Paraprevotella xylaniphila MGS:0995, Faecalibacterium sp. MGS:0073, Oscillospiraceae sp. MGS:0057, Eubacteriales sp. MGS:0589, Eubacteriales sp. MGS:0137, Oscillospiraceae sp. MGS:0146, Bacteroidales sp. MGS:1002, Eubacterium ramulus MGS:0387, Clostridium sp. OF03-18AA MGS:0119, Eubacteriales sp. MGS:0322, Eubacteriales sp. MGS:0701, Lachnospiraceae sp. MGS:0246, Ruminococcus sp. MGS:0131, Clostridium celatum MGS:0808, Eubacteriales sp. MGS:0856, Eubacteriales sp. MGS:0881, Firmicutes sp. MGS:0526, Eubacteriales sp. MGS:0472, Ruminococcus sp. AF21-42 MGS:0111, Eubacteriales sp. MGS:1289 | 178.97173[M+NaCl+H]1+, 118.9986[M-H]1- |
| taurine | Lactobacillus delbrueckii subsp. bulgaricus MGS:0465, Eubacteriales sp. MGS:0493, Oscillospiraceae sp. MGS:0057, Ruminococcus sp. AF21-42 MGS:0111 | 126.02197[M+H]1+, 163.97778[M+K]1+, 148.00388[M+Na]1+, 297.88356[M+(NaCl)3-H]1-, 355.84248[M+(NaCl)4-H]1-, 239.92478[M+(NaCl)2-H]1-, 124.00738[M-H]1-, 181.96606[M+NaCl-H]1-, 159.98409[M+Cl]1- |
| threonine | Clostridium sp. OF03-18AA MGS:0119, Eggerthellales sp. MGS:0991 | 235.9827[M+(NaCl)2+H]1+, 293.94133[M+(NaCl)3+H]1+, 178.02408[M+NaCl+H]1+, 142.0475[M+Na]1+, 120.06552[M+H]1+, 158.02142[M+K]1+, 291.92654[M+(NaCl)3-H]1-, 233.96828[M+(NaCl)2-H]1-, 176.00959[M+NaCl-H]1-, 154.02766[M+Cl]1-, 118.05102[M-H]1- |
| tiglylcarnitine (C5:1-DC) | Lactobacillus delbrueckii subsp. bulgaricus MGS:0465, Lactococcus lactis subsp. lactis MGS:0318, Eubacterium ramulus MGS:0068, Faecalibacterium sp. MGS:0073, Oscillospiraceae sp. MGS:0057, Eubacteriales sp. MGS:0589, Negativibacillus massiliensis MGS:0356, Eubacteriales sp. MGS:0137, Oscillospiraceae sp. MGS:0146, Bacteroidales sp. MGS:1002, Clostridium sp. OF03-18AA MGS:0119, Eubacteriales sp. MGS:0322, Lachnospiraceae sp. MGS:0246, Eubacteriales sp. MGS:0123, Ruminococcus sp. MGS:0131, Clostridium celatum MGS:0808, Eubacteriales sp. MGS:0856, Firmicutes sp. MGS:0526, Eubacteriales sp. MGS:0472, Ruminococcus sp. AF21-42 MGS:0111 | 266.13616[M+Na]1+, 244.15435[M+H]1+ |
| trans-4-hydroxyproline | Eubacterium ramulus MGS:0068, Negativibacillus massiliensis MGS:0356, Eubacteriales sp. MGS:0123 | 154.04746[M+Na]1+, 170.02135[M+K]1+, 132.06556[M+H]1+, 176.05641[M-H+FA]1-, 166.02763[M+Cl]1-, 130.05096[M-H]1- |
| tryptophan | Lactobacillus delbrueckii subsp. bulgaricus MGS:0465, Ruminococcus sp. AM36-2AA MGS:0096, Lactococcus lactis subsp. lactis MGS:0318, Eubacterium ramulus MGS:0068, Oscillospiraceae sp. MGS:0057, Oscillospiraceae sp. MGS:0146, Eubacterium ramulus MGS:0387, Anaerotignum faecicola MGS:0090, Ruminococcus sp. MGS:0131, Lachnospira pectinoschiza MGS:0099, Ruminococcus sp. AF21-42 MGS:0111 | 263.05612[M+NaCl+H]1+, 227.07901[M+Na]1+, 243.05307[M+K]1+, 205.09717[M+H]1+, 261.04129[M+NaCl-H]1-, 203.08267[M-H]1-, 249.08825[M-H+FA]1-, 239.05939[M+Cl]1- |
| urate | Eubacterium ramulus MGS:0068, Paraprevotella xylaniphila MGS:0995, Faecalibacterium sp. MGS:0073, Oscillospiraceae sp. MGS:0057, Oscillospiraceae sp. MGS:0146, Ruminococcus sp. AM28-41 MGS:0631, Anaerotignum faecicola MGS:0090, Eubacteriales sp. MGS:0123, Ruminococcus sp. MGS:0131, Ruminococcus sp. AF21-42 MGS:0111 | 169.03566[M+H]1+, 191.01757[M+Na]1+, 224.97979[M+NaCl-H]1-, 167.02109[M-H]1-, 202.99784[M+Cl]1-, 282.93848[M+(NaCl)2-H]1- |
| uridine | Eubacteriales sp. MGS:0493, Ruminococcus sp. AM36-2AA MGS:0096, Lactococcus lactis subsp. lactis MGS:0318, Eubacterium ramulus MGS:0068, Faecalibacterium sp. MGS:0073, Oscillospiraceae sp. MGS:0057, Eubacteriales sp. MGS:0589, Negativibacillus massiliensis MGS:0356, Eubacteriales sp. MGS:0137, Oscillospiraceae sp. MGS:0146, Eubacterium ramulus MGS:0387, Clostridium sp. OF03-18AA MGS:0119, Eubacteriales sp. MGS:0322, Lachnospiraceae sp. MGS:0246, Anaerotignum faecicola MGS:0090, Eubacteriales sp. MGS:0123, Ruminococcus sp. MGS:0131, Lachnospira pectinoschiza MGS:0099, Eubacteriales sp. MGS:0856, Firmicutes sp. MGS:0526, Eubacteriales sp. MGS:0472, Ruminococcus sp. AF21-42 MGS:0111, Eubacteriales sp. MGS:1289 | 267.05883[M+Na]1+, 289.06815[M-H+FA]1-, 279.03907[M+Cl]1-, 243.0623[M-H]1- |
| valine | Lactococcus lactis subsp. lactis MGS:0318, Eubacterium ramulus MGS:0068, Oscillospiraceae sp. MGS:0057, Negativibacillus massiliensis MGS:0356, Oscillospiraceae sp. MGS:0146, Eubacterium ramulus MGS:0387, Anaerotignum faecicola MGS:0090, Eubacteriales sp. MGS:0123, Ruminococcus sp. MGS:0131, Lachnospira pectinoschiza MGS:0099, Ruminococcus sp. AF21-42 MGS:0111 | 156.04211[M+K]1+, 140.0682[M+Na]1+, 118.0862[M+H]1+, 231.989[M+(NaCl)2-H]1-, 162.07723[M-H+FA]1-, 152.04834[M+Cl]1-, 174.0303[M+NaCl-H]1-, 116.07172[M-H]1- |
| xanthine | Ruminococcus sp. AM36-2AA MGS:0096, Eubacterium ramulus MGS:0068, Faecalibacterium sp. MGS:0073, Oscillospiraceae sp. MGS:0146, Eubacterium ramulus MGS:0387, Clostridium sp. OF03-18AA MGS:0119, Anaerotignum faecicola MGS:0090, Lachnospira pectinoschiza MGS:0099, Ruminococcus sp. AF21-42 MGS:0111 | 197.03166[M-H+FA]1-, 151.02616[M-H]1- |


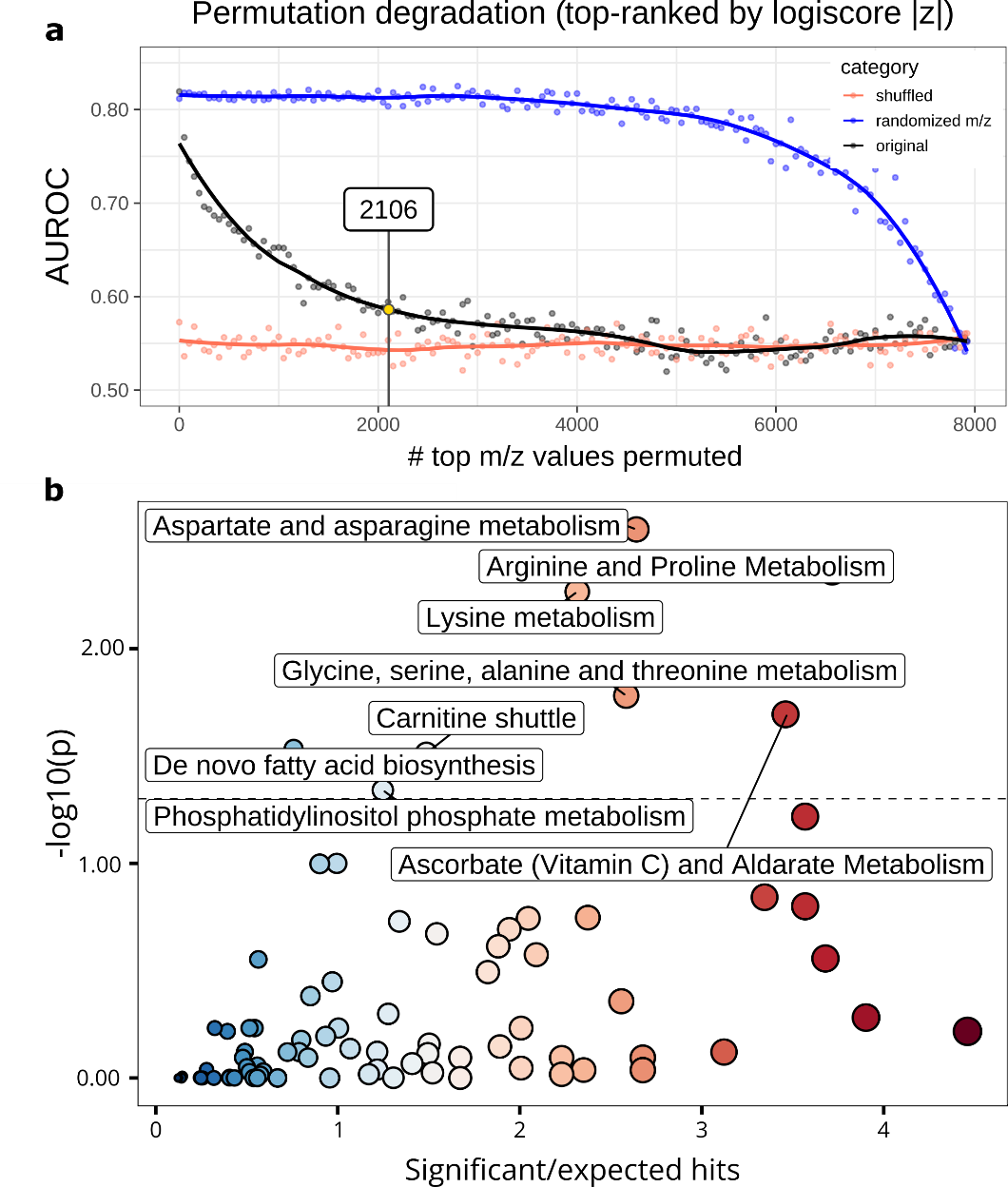


**Figure S11*:*** Logistic regression-based feature selection**,** signature threshold determination and enrichment analysis. **(a)** Signature determination in PSC. Label-shuffled negative control is represented by the red dashed line. Blue dotted line represents a second negative control built by removing random features rather than ranked. Black solid line represents the models built by removing ranked m/z values, this line is used for signature determination. **(b)** Enrichment results for MetaFishNet database. Dotted line represents an EASE p-value of 0.05.

### Table S12 – Logiscore MFN enrichment results

|  |  |  |  |  |  |  |  |  |
| --- | --- | --- | --- | --- | --- | --- | --- | --- |
|  | Pathway total | Hits.total | Hits.sig | Expected | FET | EASE | Gamma | Emp.Hits |
| Aspartate and asparagine metabolism | 114 | 60 | 45 | 17.039 | 0.0011844 | 0.0027796 | 0.0083937 | 0 |
| Arginine and Proline Metabolism | 45 | 30 | 25 | 6.726 | 0.0012316 | 0.0043582 | 0.0084179 | 0 |
| Lysine metabolism | 52 | 20 | 18 | 7.7723 | 0.0010524 | 0.0054226 | 0.0084343 | 0 |
| Glycine, serine, alanine and threonine metabolism | 88 | 46 | 34 | 13.153 | 0.0074441 | 0.016557 | 0.0086082 | 0 |
| Ascorbate (Vitamin C) and Aldarate Metabolism | 29 | 17 | 15 | 4.3346 | 0.004618 | 0.02021 | 0.0086661 | 0 |
| De novo fatty acid biosynthesis | 106 | 13 | 12 | 15.844 | 0.0054961 | 0.029246 | 0.0088114 | 0 |
| Carnitine shuttle | 72 | 19 | 16 | 10.762 | 0.0087331 | 0.030682 | 0.0088347 | 0 |
| Phosphatidylinositol phosphate metabolism | 59 | 12 | 11 | 8.8186 | 0.0092417 | 0.045581 | 0.0090812 | 0 |

### Table S13 – Logiscore MFN enrichment matches

| Compound identifier | Pathway name | Matches [adduct] |
| --- | --- | --- |

| (5-L-Glutamyl)-L-amino acid; L-gamma-Glutamyl-L-amino acid | Aspartate and asparagine metabolism | 241.07951[M+Na]1+ |
| --- | --- | --- |
| 1-Pyrroline-4-hydroxy-2-carboxylate | Aspartate and asparagine metabolism | 152.03183[M+Na]1+, 168.00575[M+K]1+ |
| 2-Hydroxyglutarate | Aspartate and asparagine metabolism | 171.02645[M+Na]1+ |
| 4-Guanidinobutanoate | Aspartate and asparagine metabolism | 146.09244[M+H]1+ |
| 5-Oxo-L-proline | Aspartate and asparagine metabolism | 152.03183[M+Na]1+, 168.00575[M+K]1+ |
| 5-Oxoproline; Pyroglutamic acid; 5-Pyrrolidone-2-carboxylic acid; Pyroglutamate; 5-Oxo-L-proline; L-Pyroglutamic acid; L-5-Pyrrolidone-2-carboxylic acid | Aspartate and asparagine metabolism | 152.03183[M+Na]1+, 168.00575[M+K]1+ |
| N-Acetylornithine; N2-Acetyl-L-ornithine | Aspartate and asparagine metabolism | 192.13422[M+NH4]1+ |
| N-Succinyl-L-glutamate 5-semialdehyde; (2S)-2-(3-Carboxypropanoylamino)-5-oxopentanoic acid | Aspartate and asparagine metabolism | 231.07398[M1+.]1+ |
| N1-Acetylspermidine | Aspartate and asparagine metabolism | 188.17576[M+H]1+ |
| N8-Acetylspermidine | Aspartate and asparagine metabolism | 188.17576[M+H]1+ |
| O-Acetylcarnitine; O-Acetyl-L-carnitine | Aspartate and asparagine metabolism | 263.08899[M+NaCl+H]1+ |
| Spermidine; N-(3-Aminopropyl)-1,4-butane-diamine | Aspartate and asparagine metabolism | 146.16522[M+H]1+ |
| Spermine; N,N'-Bis(3-aminopropyl)-1,4-butanediamine | Aspartate and asparagine metabolism | 203.22305[M+H]1+ |
| cis-4-Hydroxy-D-proline | Aspartate and asparagine metabolism | 170.02135[M+K]1+, 154.04746[M+Na]1+, 132.06556[M+H]1+ |
| dehydrospermidine | Aspartate and asparagine metabolism | 146.16522[M+H]1+ |
| spermic acid 2 | Aspartate and asparagine metabolism | 255.13159[M+Na]1+ |
| 2,3,4,5-Tetrahydropyridine-2-carboxylate; delta1-Piperideine-6-L-carboxylate | Lysine metabolism | 128.07058[M+H]1+ |
| 2-Oxoadipate; 2-Oxoadipic acid | Lysine metabolism | 183.02646[M+Na]1+ |
| 3-Dehydroxycarnitine | Lysine metabolism | 146.11758[M+H]1+, 168.09951[M+Na]1+ |
| 4-Trimethylammoniobutanoate | Lysine metabolism | 146.11758[M1+.]1+ |
| 6-Amino-2-oxohexanoate; 2-Oxo-6-aminocaproate | Lysine metabolism | 146.08117[M+H]1+, 168.06313[M+Na]1+, 184.03708[M+K]1+ |
| L-2-Aminoadipate 6-semialdehyde; 2-Aminoadipate 6-semialdehyde | Lysine metabolism | 146.08117[M+H]1+, 168.06313[M+Na]1+, 184.03708[M+K]1+ |
| L-2-Aminoadipate; L-alpha-Aminoadipate; L-alpha-Aminoadipic acid; L-2-Aminoadipic acid; L-2-Aminohexanedioate | Lysine metabolism | 184.05806[M+Na]1+ |
| L-Pipecolate; Pipecolinic acid; Pipecolic acid; 2-Piperidinecarboxylic acid | Lysine metabolism | 152.06821[M+Na]1+, 147.11281[M+NH4]1+ |
| N6,N6,N6-Trimethyl-L-lysine | Lysine metabolism | 189.15974[M+H]1+, 211.14166[M+Na]1+ |
| N6-(L-1,3-Dicarboxypropyl)-L-lysine; Saccharopine; L-Saccharopine; N-[(S)-5-Amino-5-carboxypentyl]-L-glutamic acid | Lysine metabolism | 299.12136[M+Na]1+ |
| Protein N6,N6-dimethyl-L-lysine | Lysine metabolism | 175.14409[M+H]1+ |
| delta1-Piperideine-2-carboxylate; 1,2-Didehydropiperidine-2-carboxylate | Lysine metabolism | 128.07058[M+H]1+ |
| (4R,5S)-4,5,6-Trihydroxy-2,3-dioxohexanoate | Ascorbate (Vitamin C) and Aldarate Metabolism | 230.99018[M+K]1+, 215.01617[M+Na]1+ |
| Ascorbate; Ascorbic acid; L-Ascorbate; L-Ascorbic acid; Vitamin C | Ascorbate (Vitamin C) and Aldarate Metabolism | 176.03177[M1+.]1+, 194.06598[M+NH4]1+ |
| D-glucurono-6,3-lactone | Ascorbate (Vitamin C) and Aldarate Metabolism | 176.03177[M1+.]1+, 194.06598[M+NH4]1+ |
| Dehydroascorbate | Ascorbate (Vitamin C) and Aldarate Metabolism | 212.97947[M+K]1+ |
| L-Gulonate; L-Gulonic acid; Gulonate; Gulonic acid | Ascorbate (Vitamin C) and Aldarate Metabolism | 219.04749[M+Na]1+ |
| L-Lyxonate; L-Lyxonic acid | Ascorbate (Vitamin C) and Aldarate Metabolism | 184.08167[M+NH4]1+ |
| L-Xylonate; L-Xylonic acid | Ascorbate (Vitamin C) and Aldarate Metabolism | 184.08167[M+NH4]1+ |
| Lipoate; Lipoic acid; alpha-Lipoic acid; Thioctic acid | Ascorbate (Vitamin C) and Aldarate Metabolism | 207.05068[M+H]1+, 265.00907[M+NaCl+H]1+ |
| Threonate | Ascorbate (Vitamin C) and Aldarate Metabolism | 195.00309[M+NaCl+H]1+ |
| monodehydroascorbate(1-) | Ascorbate (Vitamin C) and Aldarate Metabolism | 193.05838[M+NH4]1+, 176.03177[M+H]1+ |
| (9Z)-Octadecenoic acid; (Z)-Octadec-9-enoic acid; Oleate; Oleic acid | De novo fatty acid biosynthesis | 321.21899[M+K]1+ |
| 11-cis-eicosenoate | De novo fatty acid biosynthesis | 542.1209[M+(NaCl)4+H]1+ |
| Icosanoic acid; Eicosanoic acid; Arachidic acid | De novo fatty acid biosynthesis | 335.29197[M+Na]1+ |
| Linoleate; Linoleic acid; (9Z,12Z)-Octadecadienoic acid; 9-cis,12-cis-Octadecadienoate; 9-cis,12-cis-Octadecadienoic acid | De novo fatty acid biosynthesis | 319.20337[M+K]1+, 303.22943[M+Na]1+ |
| Elaidic carnitine | Carnitine shuttle | 426.35765[M+H]1+ |
| Hexadecenoyl carnitine | Carnitine shuttle | 398.32633[M+H]1+ |
| L-Palmitoylcarnitine | Carnitine shuttle | 400.34211[M+H]1+ |
| Linoelaidyl carnitine | Carnitine shuttle | 424.34204[M+H]1+ |
| Linoleyl carnitine | Carnitine shuttle | 424.34204[M+H]1+ |
| Vaccenyl carnitine | Carnitine shuttle | 426.35765[M+H]1+ |
| octadecenoyl carnitine | Carnitine shuttle | 426.35765[M+H]1+ |
| pendtadenoyl carnitine | Carnitine shuttle | 424.28234[M+K]1+ |
| propionyl-carnitine | Carnitine shuttle | 218.13868[M+H]1+, 240.12058[M+Na]1+ |
| stearoylcarnitine | Carnitine shuttle | 428.37321[M+H]1+ |
| tetradecanoyl carnitine | Carnitine shuttle | 372.31078[M+H]1+ |
| trans-Hexadec-2-enoyl carnitine | Carnitine shuttle | 398.32633[M+H]1+ |
| 1D-myo-Inositol 1,3,4,5-tetrakisphosphate; D-myo-Inositol 1,3,4,5-tetrakisphosphate; Inositol 1,3,4,5-tetrakisphosphate | Phosphatidylinositol phosphate metabolism | 500.9369[M+H]1+ |
| 1D-myo-Inositol 1,3,4,6-tetrakisphosphate; D-myo-Inositol 1,3,4,6-tetrakisphosphate; Inositol 1,3,4,6-tetrakisphosphate | Phosphatidylinositol phosphate metabolism | 500.9369[M+H]1+ |
| 1D-myo-Inositol 1,4,5,6-tetrakisphosphate; D-myo-Inositol 1,4,5,6-tetrakisphosphate; Inositol 1,4,5,6-tetrakisphosphate | Phosphatidylinositol phosphate metabolism | 500.9369[M+H]1+ |
| 1D-myo-Inositol 3,4,5,6-tetrakisphosphate; D-myo-Inositol 3,4,5,6-tetrakisphosphate; Inositol 3,4,5,6-tetrakisphosphate | Phosphatidylinositol phosphate metabolism | 500.9369[M+H]1+ |
| D-Galactose | Phosphatidylinositol phosphate metabolism | 296.98805[M+(NaCl)2+H]1+ |
| D-myo-Inositol 1,2-cyclic phosphate; 1D-myo-Inositol 1,2-cyclic phosphate | Phosphatidylinositol phosphate metabolism | 242.01891[M1+.]1+, 243.02693[M+H]1+ |
| Glycerol; Glycerin; 1,2,3-Trihydroxypropane; 1,2,3-Propanetriol | Phosphatidylinositol phosphate metabolism | 93.05464[M+H]1+ |
| myo-Inositol; D-myo-Inositol; 1D-myo-Inositol; L-myo-Inositol; 1L-myo-Inositol; meso-Inositol; Inositol; Dambose; Cyclohexitol; Meat sugar; Bios I | Phosphatidylinositol phosphate metabolism | 296.98805[M+(NaCl)2+H]1+ |
| L-Methionine; Methionine; L-2-Amino-4methylthiobutyric acid | Arginine and Proline Metabolism, Glycine, serine, alanine and threonine metabolism | 150.05837[M+H]1+, 172.04022[M+Na]1+, 188.01428[M+K]1+ |
| Pyruvate; Pyruvic acid; 2-Oxopropanoate; 2-Oxopropanoic acid; Pyroracemic acid | Arginine and Proline Metabolism, Glycine, serine, alanine and threonine metabolism | 111.00526[M+Na]1+ |
| D-Glucuronate; Glucuronic acid; Glucuronate | Ascorbate (Vitamin C) and Aldarate Metabolism, Phosphatidylinositol phosphate metabolism | 217.03196[M+Na]1+ |
| 4-Acetamidobutanoate; N4-Acetylaminobutanoate | Aspartate and asparagine metabolism, Arginine and Proline Metabolism | 146.08117[M+H]1+, 168.06313[M+Na]1+, 184.03708[M+K]1+ |
| 4-Aminobutanal; 4-Aminobutyraldehyde; Butyraldehyde, 4-amino- | Aspartate and asparagine metabolism, Arginine and Proline Metabolism | 88.07567[M+H]1+ |
| L-1-Pyrroline-3-hydroxy-5-carboxylate; 3-Hydroxy-L-1-pyrroline-5-carboxylate; (3R,5S)-1-Pyrroline-3-hydroxy-5-carboxylate | Aspartate and asparagine metabolism, Arginine and Proline Metabolism | 152.03183[M+Na]1+, 168.00575[M+K]1+ |
| L-Citrulline; 2-Amino-5-ureidovaleric acid; Citrulline | Aspartate and asparagine metabolism, Arginine and Proline Metabolism | 198.08497[M+Na]1+, 176.10295[M+H]1+, 292.02074[M+(NaCl)2+H]1+ |
| L-Glutamate 5-semialdehyde; L-Glutamate gamma-semialdehyde | Aspartate and asparagine metabolism, Arginine and Proline Metabolism | 170.02135[M+K]1+, 154.04746[M+Na]1+, 132.06556[M+H]1+ |
| N4-Acetylaminobutanal | Aspartate and asparagine metabolism, Arginine and Proline Metabolism | 152.06821[M+Na]1+, 147.11281[M+NH4]1+ |
| Putrescine; 1,4-Butanediamine; 1,4-Diaminobutane; Tetramethylenediamine | Aspartate and asparagine metabolism, Arginine and Proline Metabolism | 378.90016[M+(NaCl)5+H]1+ |
| Urea; Carbamide | Aspartate and asparagine metabolism, Arginine and Proline Metabolism | 98.99551[M+K]1+ |
| trans-4-Hydroxy-L-proline | Aspartate and asparagine metabolism, Arginine and Proline Metabolism | 170.02135[M+K]1+, 154.04746[M+Na]1+, 132.06556[M+H]1+ |
| L-Arginine; (S)-2-Amino-5-guanidinovaleric acid | Aspartate and asparagine metabolism, Arginine and Proline Metabolism, Glycine, serine, alanine and threonine metabolism | 175.11895[M+H]1+, 197.10093[M+Na]1+ |
| 2-Oxoglutarate; Oxoglutaric acid; 2-Ketoglutaric acid; alpha-Ketoglutaric acid | Aspartate and asparagine metabolism, Arginine and Proline Metabolism, Lysine metabolism, Glycine, serine, alanine and threonine metabolism | 262.94591[M+(NaCl)2+H]1+ |
| Carnitine; gamma-Trimethyl-hydroxybutyrobetaine; 3-Hydroxy-4-trimethylammoniobutanoate | Aspartate and asparagine metabolism, Lysine metabolism | 162.11244[M1+.]1+ |
| L-Lysine; Lysine acid; 2,6-Diaminohexanoic acid | Aspartate and asparagine metabolism, Lysine metabolism | 169.09475[M+Na]1+, 147.11281[M+H]1+ |
| Hexadecanoic acid; Hexadecanoate; Hexadecylic acid; Palmitic acid; Palmitate; Cetylic acid | De novo fatty acid biosynthesis, Phosphatidylinositol phosphate metabolism | 279.22936[M+Na]1+, 295.20328[M+K]1+ |
| 2-Phosphoglycolate; Phosphoglycolic acid | Glycine, serine, alanine and threonine metabolism | 178.97173[M+Na]1+ |
| 2-methylbutyrylglycine | Glycine, serine, alanine and threonine metabolism | 159.08931[M+H]1+ |
| 3-Phosphonooxypyruvate; 3-Phosphonooxypyruvic acid; 3-Phosphohydroxypyruvate; 3-Phosphohydroxypyruvic acid | Glycine, serine, alanine and threonine metabolism | 206.96664[M+Na]1+ |
| 3-methylcrotonoylglycine | Glycine, serine, alanine and threonine metabolism | 215.03157[M+NaCl+H]1+ |
| 5-Aminolevulinate; 5-Amino-4-oxopentanoate; 5-Amino-4-oxovaleric acid | Glycine, serine, alanine and threonine metabolism | 170.02135[M+K]1+, 154.04746[M+Na]1+, 132.06556[M+H]1+ |
| Aminoacetone; 1-Amino-2-propanone | Glycine, serine, alanine and threonine metabolism | 112.01589[M+K]1+, 96.04195[M+Na]1+ |
| Betaine; Trimethylaminoacetate; Glycine betaine; N,N,N-Trimethylglycine; Trimethylammonioacetate | Glycine, serine, alanine and threonine metabolism | 156.04211[M+K]1+ |
| Creatine; alpha-Methylguanidino acetic acid; Methylglycocyamine | Glycine, serine, alanine and threonine metabolism | 154.05869[M+Na]1+, 132.07679[M+H]1+, 170.03266[M+K]1+ |
| D-Glycerate; Glycerate; (R)-Glycerate; Glyceric acid | Glycine, serine, alanine and threonine metabolism | 129.01583[M+Na]1+ |
| Glycolate; Glycolic acid; Hydroxyacetic acid | Glycine, serine, alanine and threonine metabolism | 99.00527[M+Na]1+ |
| Guanidinoacetate; Guanidinoacetic acid; Glycocyamine; N-Amidinoglycine; Guanidoacetic acid | Glycine, serine, alanine and threonine metabolism | 140.04307[M+Na]1+ |
| L-Homocysteine; L-2-Amino-4-mercaptobutyric acid | Glycine, serine, alanine and threonine metabolism | 158.02466[M+Na]1+ |
| isovalerylglycine | Glycine, serine, alanine and threonine metabolism | 159.08931[M+H]1+ |
| L-Carnitine; L-gamma-Trimethyl-beta-hydroxybutyrobetaine; Vitamin BT; 3-Carboxy-2-hydroxy-N,N,N-trimethyl-1-propanaminium hydroxide, inner salt; Levocarnitine; (R)-Carnitine | Lysine metabolism, Carnitine shuttle | 162.11244[M+H]1+ |
